# Metathetical Exchange, Synthesis, and Carbon Dioxide Addition of Higher Homologues of Group 9 Metal Carbynes

**DOI:** 10.1002/anie.202503930

**Published:** 2025-05-23

**Authors:** Lennart G. Holzapfel, Johanna Manegold, Stefan F. Clewing, Hartmut Schubert, Klaus Eichele, Lars Wesemann

**Affiliations:** ^1^ Institut für Anorganische Chemie, Eberhard Karls Universität Tübingen Auf der Morgenstelle 18 72076 Tübingen Germany

**Keywords:** Cobalt, Iridium, Metathesis, Rhodium, Tetrylidyne

## Abstract

A one‐pot synthesis for previously unknown heavy homologues of Group 9 metal carbynes [(Me_3_P)_3_Co≡GeAr*] (**1**), [(Me_3_P)_3_Rh≡GeAr*] (**2**), [(Me_3_P)_3_Ir≡GeAr*] (**3**), [(Me_3_P)_3_Ir≡SnAr*] (**5**), and [(Et_3_P)_3_Ir≡PbAr*] (**7**) is presented [Ar* = C_6_H_3_‐2,6‐(Trip)_2_, Trip = 2,4,6‐C_6_H_2_ *i*Pr_3_]. During these preparations, the tetrylidynes [(Me_3_P)_3_Rh≡SnAr*] (**4**), and [(Me_3_P)_3_Rh≡PbAr*] (**6**), were also prepared in a one‐pot procedure. In a hitherto unknown metathetical exchange reaction, the transition metal plumbylidynes were converted into the respective stannylidynes in reaction with terphenyl stannylene chloride, and the germylidynes were formed from the corresponding stannylidynes in reaction with terphenyl germylene chloride. These metathesis reactions were tracked by ^31^P{^1^H} NMR spectroscopy, which shows complete exchange of the tetrel [EAr]‐fragment and the formation of an intermediate in the rhodium plumbylidyne reaction with stannylene chloride (Ar = Ar*, Tbb). Reactions of the tetrylidynes (**2**–**5**) with carbon dioxide yield the products of a redox reaction [(Me_3_P)_3_(CO)M‐E(*η* ^2^‐O_2_CO‐*κ* ^2^ *O*)Ar*] [E = Ge, M = Rh (**11**); M = Ir (**12**); E = Sn, M = Rh (**14**), M = Ir (**16**)] with a carbon monoxide coordinated at the transition metal and a carbonate coordinated at the Group 14 element. For tin the intermediates formed by mono CO_2_ addition [(Me_3_P)_3_M(*μ*,*η* ^2^‐CO_2_‐*κC*:*κO*)SnAr*] [M = Rh (**13**), M = Ir (**15**)] have been isolated.

## Graphical abstract

A one‐pot synthesis for previously unknown heavy carbynes of Group 9 metals is presented. Furthermore, in a hitherto unknown metathetical exchange reaction, the transition metal plumbylidynes were converted into the respective stannylidynes in reaction with terphenyl stannylene chloride, and the germylidynes were formed from the corresponding stannylidynes in reaction with terphenyl germylene chloride.

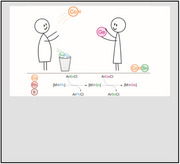

## Introduction

The higher homologues of the transition metal carbynes are called tetrylidynes. These compounds are characterized by a linear X–E–M unit with a short M–E bond length (E = Si, Ge, Sn, Pb; M = transition metal, X = substituent at E).^[^ ^1^, ^2^, ^3^, ^4^ ^]^ Almost thirty years ago, Power and coworkers synthesized the first example [Cp(CO)_2_Mo≡GeAr’] and later its chromium and tungsten analogues by treating Ar'GeCl with the respective anion [CpM(CO)_3_]^−^ (M = Cr, Mo, W) in a nucleophilic substitution at the germylene chloride followed by the elimination of CO [Ar’ = C_6_H_3_‐2,6‐(Mes)_2_, Mes = 2,4,6‐trimethylphenyl].^[^ ^5^ ^]^ The homologous chromium and tungsten compounds were also synthesized by nucleophilic substitution at the germylene chloride Ar'GeCl.^[^ ^6^ ^]^ Today, a large variety of compounds featuring a triple bond between a transition metal (M = Nb, Cr, Mo, W, Mn, Re, Fe, Ni, Pt) and a heavy Group 14 element (E = Si, Ge, Sn, Pb) have been reported by Filippou et al. using one of the following synthetic strategies:^[^ ^7^, ^8^, ^9^, ^10^, ^11^, ^12^, ^13^, ^14^, ^15^, ^16^, ^17^, ^18^, ^19^, ^20^, ^21^, ^22^, ^23^, ^24^, ^25^ ^]^ addition of dibromodisilene to anionic transition metal complexes Li[Cp*M(CO)_3_] (M = Cr–W),^[^ ^25^ ^]^ nucleophilic substitution at organotetrel halides by anionic transition metal complexes,^[^ ^15^, ^22^ ^]^ elimination of N_2_ or PMe_3_ ligands and oxidative addition of organotetrel halides at transition metal complexes.^[^ ^7^, ^8^, ^9^, ^10^, ^11^, ^12^, ^13^, ^17^, ^19^ ^]^ Hashimoto, Tobita and coworkers established a stepwise dehydrogenation route and a hydrogen transfer to mesityl isocyanate and nitriles to access the transition metal tetrylidynes [W≡Si] and [W≡Ge].^[^ ^26^, ^27^, ^28^, ^29^, ^30^, ^31^ ^]^ Hydride abstraction of [Os(H)═Si(H)] was successfully employed by Tilley et al. to synthesize an osmium complex featuring an osmium–silicon triple bond.^[^ ^32^ ^]^ After elimination of CO ligands from [CpMo(CO)_3_(GeN(Ar’’)SiMe_3_] (Ar’’ = C_6_H_2_{C(H)Ph)_2_}_2_Me‐2,4,6), Jones and coworkers characterized a molybdenum aminogermylidyne complex.^[^ ^33^ ^]^ Hadlington and coworkers treated tetrylene chlorides with Ni(COD)_2_ followed by chloride abstraction to yield the respective nickel aminotetrylidynes [Ni≡Ge] and [Ni≡Sn].^[^ ^34^ ^]^

**Scheme 1:**
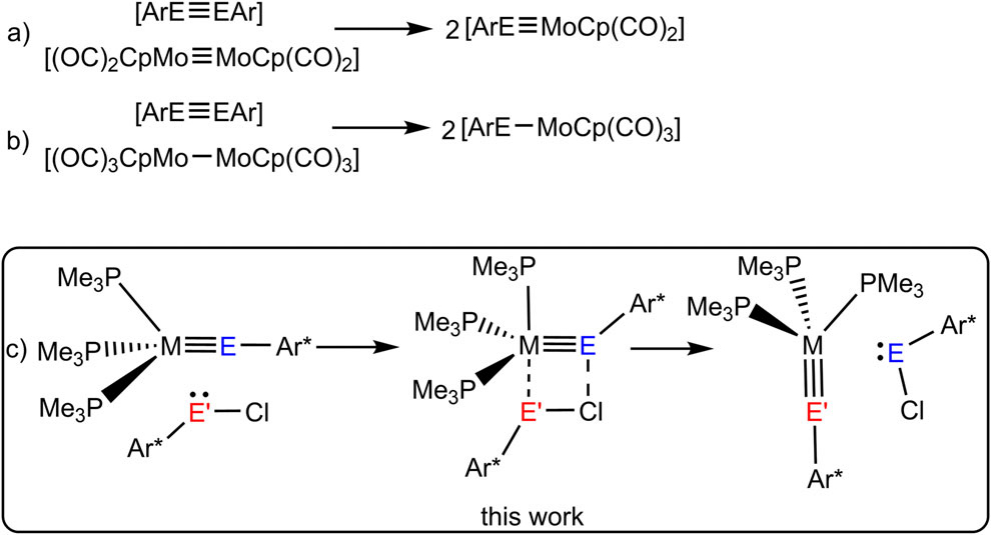
Metathetical exchange reactions of low valent Group 14 element compounds [a) E = Ge, Sn, Pb; b) E = Sn, Pb; a), and b) Ar = Ar* or C_6_H_3_‐2,6‐(C_6_H_3_‐2,6‐(*i*Pr)_2_; c) E = Sn, Pb; E’ = Ge, Sn, Ar* = C_6_H_3_‐2,6‐(Trip)_2_, Trip = 2,4,6‐C_6_H_2_ *i*Pr_3_].

Furthermore, Power and coworkers presented a metathetical exchange reaction between metal‐metal triple bonds of transition metal molybdenum and main group metals (E = Ge, Sn, and Pb) for the synthesis of carbyne homologues of molybdenum [Cp(CO)_2_Mo≡EAr*] (Scheme 1a).^[^ ^35^ ^]^ The carbonyl complex (CO)_2_CpMo≡MoCp(CO)_2_ was treated with the dimetallynes ArE≡EAr [E = Ge (80 °C), Sn (rt), or Pb (rt)] to yield tetrylidynes of molybdenum [Cp(CO)_2_Mo≡EAr]. However, in reaction of the dimetallynes ArE≡EAr with (CO)_3_CpMo–MoCp(CO)_3_ arylmolybdatetrylenes [Cp(CO)_3_Mo–EAr] (E = Sn, Pb) were obtained (Scheme 1b).^[^ ^35^, ^36^ ^]^

We are investigating the chemistry of novel higher homologues of Group 9 metal carbynes and recently reported complexes of cobalt [Co≡E] (E = Sn, Pb)^[^ ^37^ ^]^ and rhodium [Rh≡E] (E = Sn, Pb).^[^ ^38^ ^]^ The cobalt compounds were synthesized by nucleophilic substitution treating the anion [Co(PMe_3_)_4_]^−^ with low valent bromides of tin and lead [Li(thf)_2_][TbbEBr_2_] (E = Sn, Pb) (Tbb = 2,6‐[CH(SiMe_3_)_2_]_2_‐4‐(*t*Bu)C_6_H_2_).^[^ ^37^ ^]^ Rhodium tetrylidynes [(Me_3_P)_2_(Ph_3_P)Rh≡EAr*] (E = Sn, Pb) were synthesized from the hydride complexes [(Ph_3_P)_2_RhH_2_EAr*] (E = Sn, Pb) by hydrogen transfer to styrene and addition of PMe_3_.^[^ ^38^ ^]^

Quantum chemical investigations indicate that the metal‐element triple bonds consist of a σ‐bond, which results from donation of the main group element lone‐pair to the transition metal, and two π‐bonds derived from transition metal d‐orbital donation into empty p‐orbitals at the Group 14 element.^[^ ^7^, ^34^ ^]^

Recently, reactivity studies of the higher homologues of carbyne complexes have come into focus. Silylidyne and germylidyne complexes have been reported to show [2+2]‐cycloaddition reactions at the triple bond, activate CH‐bonds, add nucleophiles at the Si‐atom, and react with alcohols and α,β‐unsaturated ketones.^[^ ^32^, ^39^, ^40^, ^41^ ^]^ Hydrogen addition to rhodium element triple bonds of tin and lead was reported. The [Rh≡Sn] adds a second equivalent of hydrogen in a reversible reaction.^[^ ^38^ ^]^ Reaction of water with a [Co≡Sn] complex results in an addition of two equivalents to give a [(Me_3_P)_3_H_2_Co–Sn(OH)_2_Tbb] complex.^[^ ^37^ ^]^ The chromium silicon triple bond in [Cp*(CO)_2_Cr≡Si‐Eind] was shown to react with hydrogen or benzene under UV (365 nm) irradiation (Eind = 1,1,3,3,5,5,7,7‐octaethyl‐s‐hydrindacene‐4‐yl).^[^ ^42^ ^]^ Cationic tungsten complexes featuring a [W≡Ge] triple bond activate formyl CH‐bonds of benzaldehyde and ortho CH‐bonds of pyridine.^[^ ^43^ ^]^ The metal–silicon triple bonds in [Cp*(CO)_2_M≡Si‐Tbb] (M = Cr–W) add mesityl isocyanate, react in a ring opening of ethyloxirane, perform a double [2+1]‐cycloaddition of trimethylsilyldiazomethane and eliminate nitrogen in reaction with mesityl azide.^[^ ^25^ ^]^

**Scheme 2:**
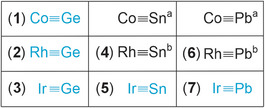
Tetrylidynes of transition metals Co, Rh, and Ir (blue: this publication) a,^[^ ^37^ ^]^ b.^[^ ^38^ ^]^

To set up a comparative reactivity study of higher homologues of Group 9 metal carbynes, we investigated the syntheses of so far unknown tetrylidynes (Scheme 2). In this publication, we present a straightforward one‐pot procedure for the synthesis of the first heavy carbynes of iridium [Ir≡Ge] (**3**), [Ir≡Sn] (**5**), [Ir≡Pb] (**7**), as well as previously unknown germylidynes of cobalt [Co≡Ge] (**1**) and rhodium [Rh≡Ge] (**2**) (Scheme 2). In addition, improved procedures for tetrylidynes of rhodium (**4**, **6**) have been elaborated. A previously unknown metathetical exchange has been developed to convert transition metal plumbylidynes to the respective stannylidynes, and stannylidynes to the corresponding germylidynes (Scheme 1c).^[^ ^35^, ^44^, ^45^, ^46^, ^47^ ^]^ Furthermore, the reactivity of the metal–element triple bond is studied with respect to carbon dioxide addition reactions.

## Results and Discussion

**Scheme 3:**
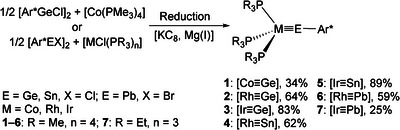
Syntheses of Group 9 tetrylidynes. For the syntheses of [Rh≡Pb] (**6**) and [Ir≡Pb] (**7**) Mg(I) = [^Mes^NacnacMg]_2_ was used as the reducing reagent.^[^ ^49^, ^50^ ^]^

**Figure 1:**
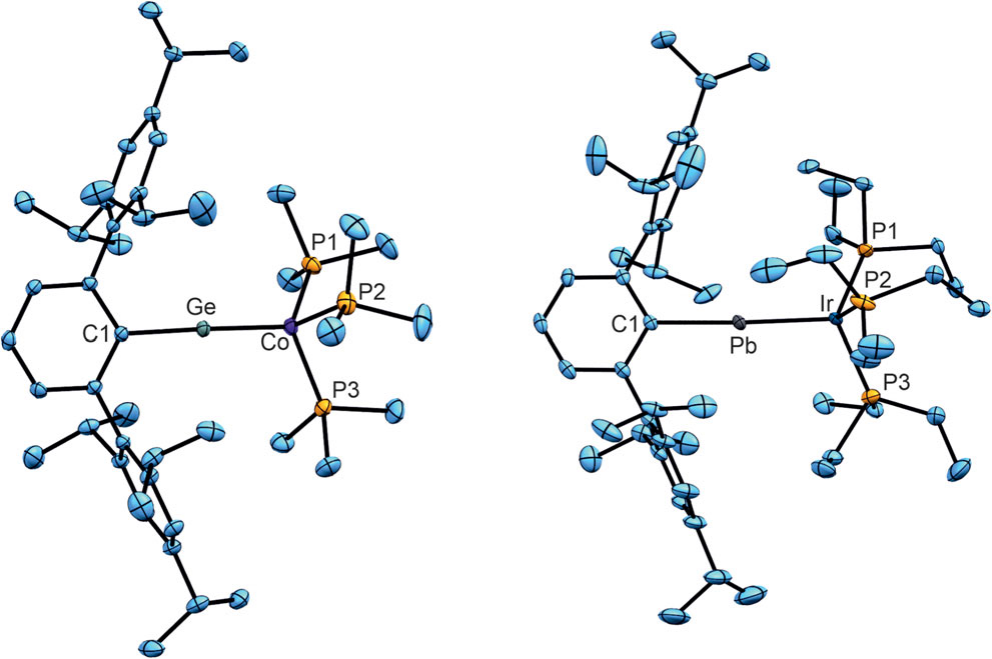
ORTEPs of molecular structures of tetrylidynes **1** (left) and **7** (right). Hydrogen atoms have been omitted. Ellipsoids set at 50% probability. Plots of the other molecular structures (**2**–**6**) have been placed in the Supporting Information.^[^ ^51^ ^]^

A hitherto unknown germylidyne of cobalt **1** was synthesized straightforwardly by reduction of a mixture of tetrakis(trimethylphosphine) cobalt [Co(PMe_3_)_4_] and terphenyl germanium chloride [Ar*GeCl]_2_ in toluene with one equivalent of KC_8_ at room temperature (Scheme 3).^[^ ^48^ ^]^ Dark green crystals of the germylidyne **1** were obtained from a concentrated *o*‐difluorobenzene solution at −40 °C. The molecular structure features a Co–Ge–C1 unit close to linearity [176.9(1)°] and a short Co–Ge bond [2.0999(2) Å] (Figure 1, Table 1). To the best of our knowledge, this Co–Ge bond is the shortest bond between these elements so far in comparison to the Co–Ge bond length observed in a hydridogermylene complex of cobalt {[(Me_3_P)_3_CoHGeAr*], Ge‐Co: 2.1918(4) Å}.^[^ ^37^ ^]^ The germylidynes of rhodium and iridium were obtained in moderate yield and on a relatively large scale (**2**: 487 mg, 64%; **3**: 758 mg, 83%) by treating a mixture of the respective tetrakis(trimethylphosphine) metal chloride [MCl(PMe_3_)_4_] (M = Rh, Ir) and terphenyl germylene chloride [Ar*GeCl]_2_ with two equivalents of the reducing reagent KC_8_ (Scheme 3).

**Table 1:** Selected interatomic distances (Å) and angles (°) in tetrylidynes **1**–**7**.

	M≡E	E–C1	M–E–C1
**1** [Co≡Ge]	2.0999(2)	1.988(1)	176.9(1)
**2** [Rh≡Ge]	2.1953(3)	1.999(3)	178.0(1)
**3** [Ir≡Ge]	2.2087(3)	1.985(2)	178.1(1)
**4** [Rh≡Sn]	2.3748(3)	2.197(3)	177.4(1)
**5** [Ir≡Sn]	2.3909(3)	2.178(4)	177.8(1)
**6** [Rh≡Pb]	2.4457(3)	2.299(3)	174.9(1)
**7** [Ir≡Pb]	2.4743(1)	2.277(2)	177.2(1)

As expected, the molecular structures of compounds **2** and **3** display a nearly linear C1–Ge–M unit [**2**: 178.0(1), **3**: 178.1(1)°] and short bond lengths (see Supporting Information for ORTEP). The length of the triple bonds in compounds **2** and **3** [**2** Rh–Ge: 2.1953(3), **3** Ir–Ge: 2.2087(3) Å] are the shortest described bonds between these elements. Short Rh–Ge bond lengths were found in a dinuclear rhodium complex featuring a [Rh–Ge–Rh] unit and Rh–Ge bond lengths of 2.3005(3) and 2.3088(3) Å.^[^ ^52^ ^]^ The Ir–Ge bond length can be compared with Ir–Ge interatomic distances observed in the germylene coordination compound [(Me_3_P)_3_HIr═GeBrTbb] [Ir–Ge: 2.2879(3) Å], and the cation [(Me_3_P)_3_HIr═GeAr*]^+^, [Ir–Ge: 2.225(2) Å].^[^ ^53^ ^]^ The stannylidyne of iridium **5** was also synthesized by KC_8_ reduction (Scheme 3) and isolated as a brown powder in high yield (89%). By X‐ray diffraction a very short Ir–Sn bond was determined in complex **5** [Ir–Sn: 2.3909(3) Å] (see Supporting Information for molecular structure, Table 1) comparable to the interatomic distance in the cationic iridium compound [(Me_3_P)_3_HIr═SnTbb]^+^ [Ir–Sn: 2.4135(5) Å].^[^ ^53^ ^]^ The heaviest homologue in the series of Group 9 metal tetrylidynes, the plumbylidyne of iridium **7** was synthesized from a mixture of tris(triethylphosphine) iridium chloride [IrCl(PEt_3_)_3_] and half of an equivalent of the plumbylene [Ar*PbBr]_2_. The PEt_3_ precursor was used due to problems of purification and crystallization with the PMe_3_ product. Since the plumbylidyne product was obtained unselectively in reduction with KC_8_, the Mg(I) reagent [^Mes^NacNacMg]_2_ was employed as a reducing agent.^[^ ^49^, ^50^ ^]^ Consistent with previous results, the Ir–Pb bond in the molecular structure of **7** (Figure 1, Table 1) [Ir–Pb: 2.4743(1) Å] is the shortest Ir–Pb bond so far. Complexes displaying an Ir–Pb bond are rare, but published interatomic distances vary in the range of 2.844(3)–3.117(1) Å.^[^ ^54^, ^55^ ^]^

**Figure 2:**
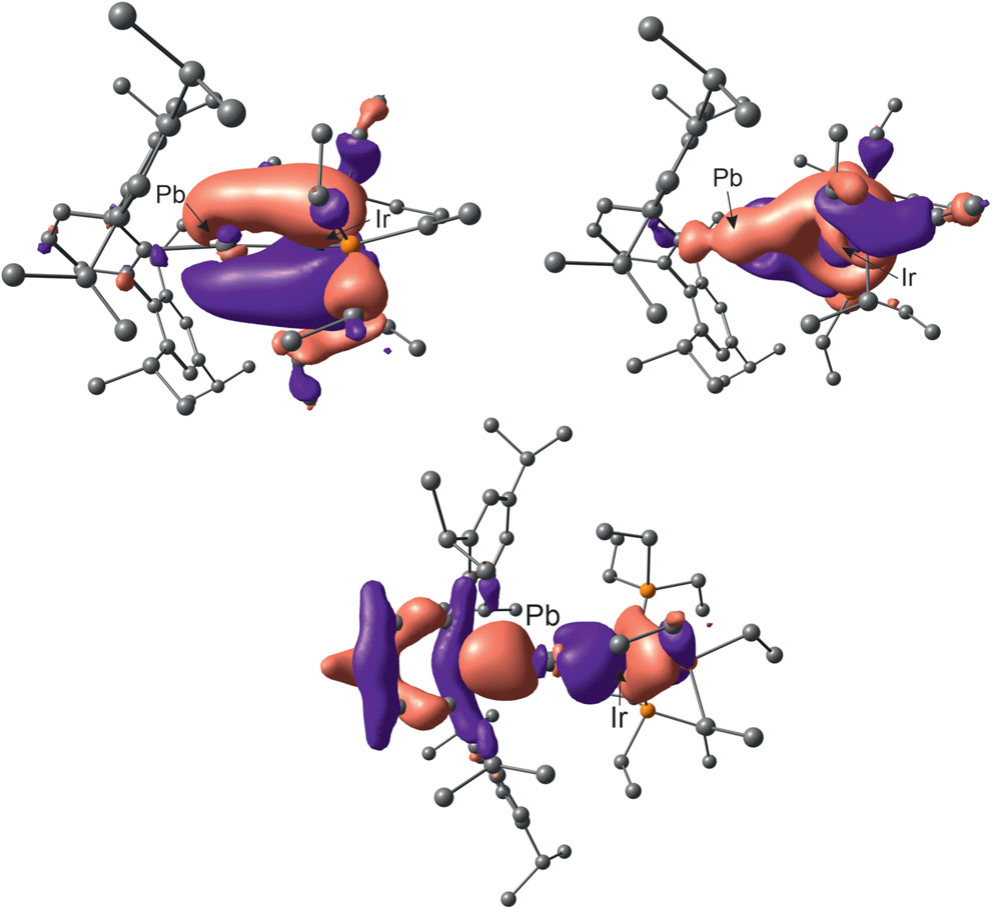
HOMO (top left), HOMO–1 (top right) and HOMO–11 (bottom) of **7** (contour value 0.03).

DFT calculations [BP86 D3(BJ)/def2SVP (Co, Rh, Ir, Ge, Sn, Pb: def2‐TZVP)]^[^ ^56^, ^57^, ^58^, ^59^, ^60^, ^61^, ^62^, ^63^, ^64^, ^65^, ^66^, ^67^, ^68^, ^69^, ^70^, ^71^, ^72^, ^73^, ^74^, ^75^ ^]^ together with NBO^[^ ^67^ ^]^ analyses were carried out for the tetrylidyne derivatives to evaluate the electronic structure of the triple bonds (see Table SI3). The short [M≡E] bonds and the almost linear C1–E–M units were reproduced by these calculations (E = Ge, Sn, Pb; M = Co, Rh, Ir). Figure 2 shows the HOMO, HOMO–1 and HOMO–11 of **7** [Ir≡Pb], which resemble the π‐bonds and σ‐bond. The Group 9 metal tetrylidynes exhibit almost to the same extent a high polarization of the σ‐bond to the Group 14 elements and a high polarization of the π‐bonds to the transition metals. The tungsten germylidyne complex [Cl(dppe)W≡Ge(*η* ^1^‐Cp*)]^[^ ^7^ ^]^ and plumbylidynes of molybdenum and tungsten [Br(Me_3_P)_4_M≡Pb‐Ar*]^[^ ^11^, ^12^ ^]^ show less polarized σ‐ and π‐bonds.

To evaluate the bond cleavage energies of the Group 9 metal tetrylidynes [(Me_3_P)_3_M≡EAr*] (M = Co, Rh, Ir, E = Ge, Sn, Pb) the molecules were divided into preferred fragments [(Me_3_P)_3 _M]^−^ and [EAr*]^+^ and analyzed by DFT (Orca, r^2^SCAN‐3c) calculations (see Table SI4).^[^ ^56^, ^76^, ^77^, ^78^, ^79^, ^80^ ^]^ The bond cleavage energies decrease in the order [M≡Ge] > [M≡Sn] > [M≡Pb] (M = Co, Rh, Ir). Concerning the transition metals, the order [Rh≡E] < [Ir≡E] ≤ [Co≡E] (E = Ge, Sn, Pb) was found.^[^ ^81^ ^]^

The observed ^119^Sn{^1^H} and ^207^Pb{^1^H} NMR data of complexes **4**–**7** (Table 2) are in the range of published tetrylidyne complexes: [Mo≡E] (Sn 1021, Pb 9660 ppm),^[^ ^35^ ^]^ [Co≡E] (Sn 1027, Pb 5022 ppm),^[^ ^37^ ^]^ [Rh≡E] (Sn 1149, Pb 5729 ppm).^[^ ^38^ ^]^ The ^103^Rh{^1^H} NMR signals of **2** and **4** were observed as quartets due to coupling with three equivalent phosphorus atoms. In the ^13^C{^1^H} NMR spectrum of **6** and **7** the signal for the Pb–C_ipso_ carbon atom was observed at high frequency (**6**: 272.1, **7**: 254.8 ppm), which can be rationalized as an effect of the lead atom on the adjacent carbon atom and is already known for other plumbylidynes (280.6, 279.1, 278.7, 248.1 ppm).^[^ ^11^, ^12^, ^37^, ^38^, ^82^ ^]^

**Table 2:** Selected NMR data [ppm] of compounds **1**–**7**.

	*δ* (^31^P)	*δ* (^103^Rh)	*δ* (^119^Sn/^207 ^Pb)
**1** [Co≡Ge]	14.4		
**2** [Rh≡Ge]	−0.4	−8354	
**3** [Ir≡Ge]	−37.1		
**4** [Rh≡Sn]	5.7	−8230	1113
**5** [Ir≡Sn]	−34.9		908
**6** [Rh≡Pb]	59.1		5470
**7** [Ir≡Pb]	43.1		4543

During the syntheses of compounds **2**–**6**, the formation of a solid after mixing the starting materials [MCl(PMe_3_)_4_] and [Ar*ECl]_2_ in toluene was observed. In three cases, these solids were characterized as the products of a chloride transfer from the transition metal to the tetrylene. The compounds [M(PMe_3_)_4_][Ar*ECl_2_] [E = Ge, M = Rh, (**8**); E = Sn, M = Rh (**9**), M = Ir (**10**)] were isolated and characterized (see Supporting Information for Scheme and molecular structures).^[^ ^83^, ^84^, ^85^ ^]^ Interestingly, although no interaction between the transition metal and the Group 14 element is formed, the transition metal tetrylidyne complexes have been synthesized straightforwardly by reduction of these intermediately formed salts.

**Scheme 4:**
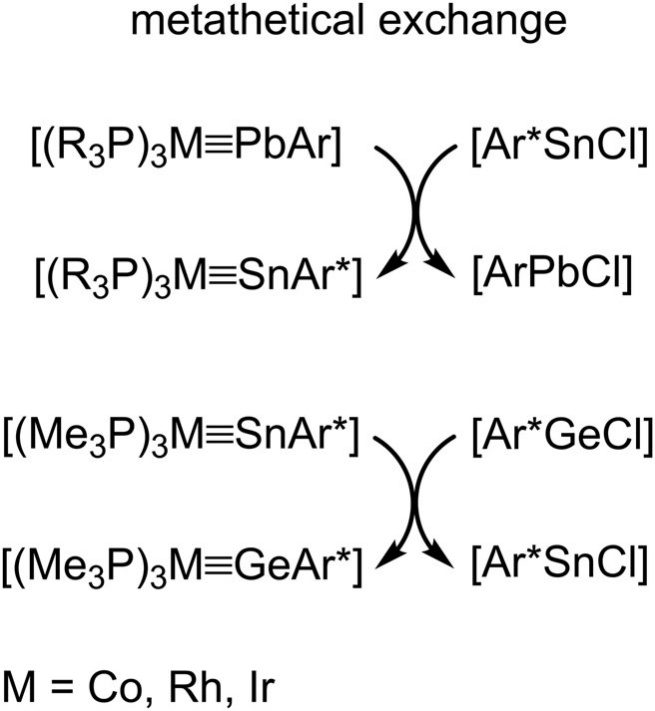
Metathetical exchange reactions of transition metal tetrylidynes [R = Me, Et; Ar = Ar*, Tbb]; [Ar*ECl] (E = Ge, Sn, Pb) are monomeric compounds in solution.^[^ ^86^, ^87^, ^88^ ^]^

**Figure 3:**
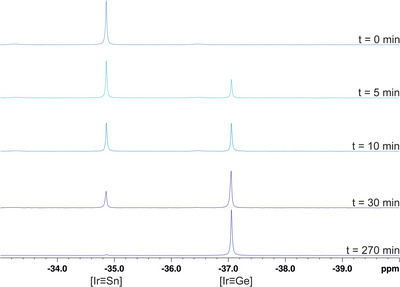
^31^P{^1^H} NMR spectra showing the progress of metathetical exchange of [Ir≡Sn] **5** with [Ar*GeCl] to yield [Ir≡Ge] **3** and [Ar*SnCl].

Treating the stannylidyne of iridium **5** [Ir≡Sn] with terphenyl germylene chloride [Ar*GeCl] leads to the formation of the homologous germylidyne **3** [Ir≡Ge] and [Ar*SnCl] (Scheme 4) as a product of a metathetical exchange. To evaluate this reaction with respect to the previously synthesized tetrylidynes of Co, Rh, and Ir, the plumbylidynes and stannylidynes of the metals Co, Rh, and Ir were treated with the lighter homologous terphenyl tetrylene chlorides (Scheme 4). In all cases, the formation of metathesis products was observed. To rationalize the thermodynamics of the metathesis reactions, these were calculated using DFT methods [Orca, BP86 D3(BJ)/def2SVP (Co, Rh, Ir, Ge, Sn, Pb: def2‐TZVP)]^[^ ^56^, ^57^, ^58^, ^59^, ^60^, ^61^, ^62^, ^63^, ^64^, ^65^, ^66^, ^67^, ^68^, ^69^ ^]^ and the results were listed in Table SI5 in the Supporting Information. To sum up the results, the metathesis from stannylidyne to germylidyne is more exergonic than the metathesis from plumbylidyne to stannylidyne. In the Group 9 metal series Co, Rh, and Ir, the free enthalpy also increases. The metathetical exchange from [Ir≡Sn] **5** → [Ir≡Ge] **3** was followed by ^31^P{^1^H} NMR spectroscopy over a period of 4.5 h (Figure 3). Based on the ^31^P{^1^H} NMR spectra, the formation of the metathesis product is a straightforward reaction.

**Figure 4:**
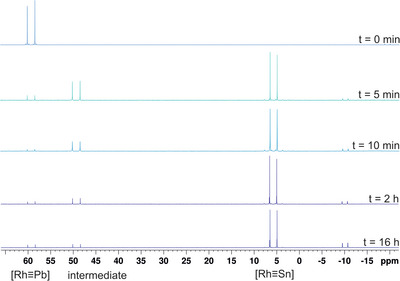
^31^P{^1^H} NMR spectra featuring the metathetical exchange of [Rh≡Pb] **6** with [Ar*SnCl] to give [Rh≡Sn] **4** and [Ar*PbCl], (−10.1 ppm = impurity).

**Scheme 5:**
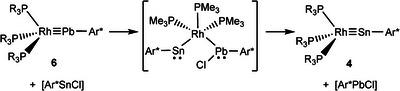
Metathetical exchange of plumbylidyne (**6**) to stannylidyne (**4**) with formation of an intermediate postulated on the basis of computations.

In the case of the rhodium plumbylidyne [Rh≡Pb] **6** (59.1 ppm, Figure 4) in reaction with chloro stannylene [Ar*SnCl], the formation of an intermediate (49.4 ppm, Figure 4) and stannylidyne [Rh≡Sn] **4** (5.7 ppm, Figure 4) was detected by ^31^P{^1^H} NMR spectroscopy. After 16 h, the formation of [Rh≡Sn] **4** was completed. However, the formation of an unknown impurity (−10.1 ppm, Figure 4, 16 h NMR spectrum) was also observed. Tin or lead satellites for the signal of the proposed intermediate were NMR spectroscopically not observed. Further indications of the possible composition of the intermediates in these reactions were derived from ^31^P{^1^H} NMR investigations on a mixture of [(Me_3_P)_3_Ir≡SnAr*] and three equivalents [Ar*SnCl] featuring a signal with ^119/117^Sn satellites whose integrals in relation to the integral of the main signal indicate the coordination of two Ar*Sn moieties at the iridium phosphine fragment (see Figure SI74 for ^31^P{^1^H} NMR spectrum, a ^119^Sn NMR signal was not found). In the presumed intermediate, the iridium atom is pentacoordinated: due to a dynamic interplay of the phosphine ligands, only one signal in the ^31^P{^1^H} NMR spectrum is found, as known from a series of pentacoordinated germylene and stannylene tris(trimethylphosphine)iridium complexes.^[^ ^53^ ^]^ The minimum energy path of the metathesis was investigated using DFT computations (Nudged elastic band method).^[^ ^56^, ^57^, ^58^, ^59^, ^60^, ^61^, ^62^, ^63^, ^64^, ^65^, ^66^, ^67^, ^68^, ^69^, ^89^ ^]^ The structure of an adduct between the plumbylidyne and chloro stannylene was found and optimized as a possible intermediate (Scheme 5).

To the best of our knowledge, the observed exchange reaction between tetrylidynes and lower homologue tetrylene chlorides is a previously unknown type of metathesis reaction. Power and coworkers presented a metathetical exchange between triple bonds to synthesize tetrylidynes of molybdenum (Scheme 1a).^[^ ^35^ ^]^

**Scheme 6:**
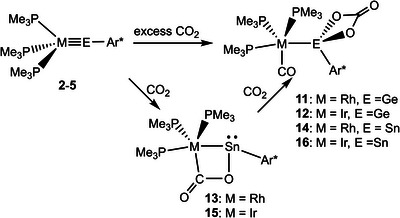
Reaction of tetrylidynes **2**–**5** with CO_2_.

The tetrylidynes **2**–**5** were treated in solution at room temperature with an excess of carbon dioxide. Two equivalents of carbon dioxide react with the tetrylidynes to yield carbonate complexes of oxidized Group 14 elements. As the product of the reduction, a CO_2_ molecule is converted to a carbon monoxide ligand, which is coordinated to the transition metal of complexes **11**, **12**, **14**, and **16** (Scheme 6).^[^ ^90^ ^]^

**Figure 5:**
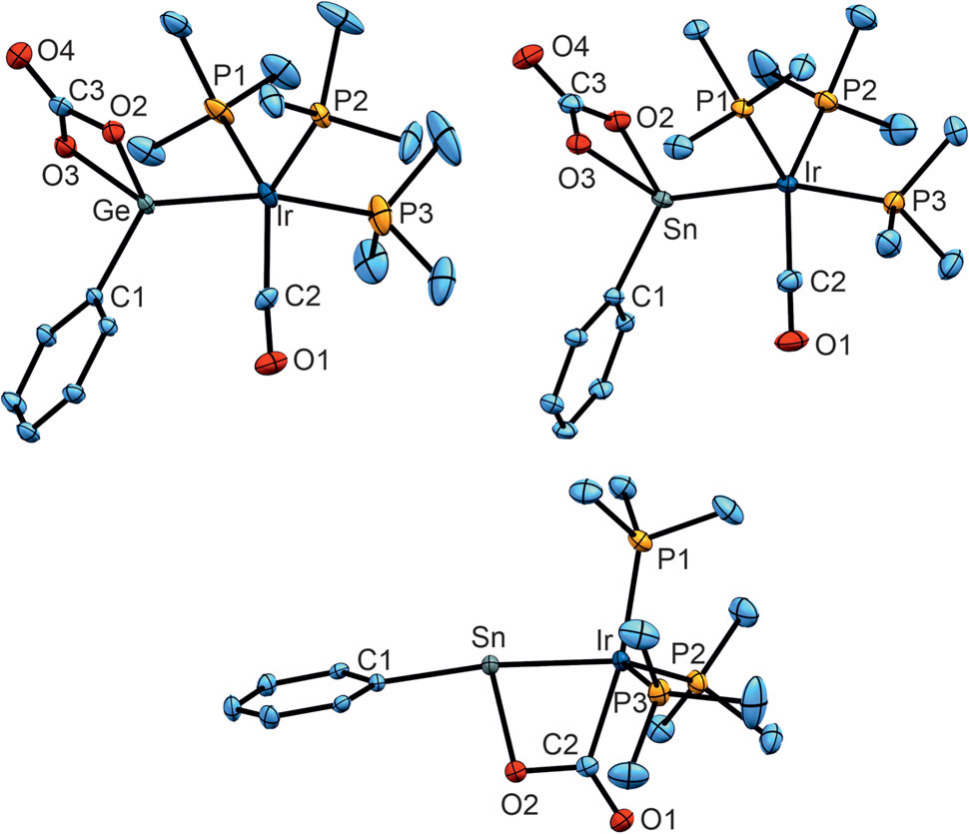
ORTEPs of the CO_2_ reaction products **12** (left), **16** (right), and **15** (middle). Ellipsoids set at 50% probability. Trip substituents and hydrogen atoms have been omitted. Interatomic distances (Å), angles (°) for **12**: Ir–Ge 2.4092(**4**), Ir–P1 2.3504(**11**), Ir–P2 2.3473(**10**), Ir–P3 2.3267(**11**), Ir–C2 1.865(**4**), C2–O1 1.156(**5**), Ge–O2 1.900(**3**), Ge‐O3 1.905(**3**), C3–O2 1.355(**5**), C3–O3 1.337(**5**), C3–O4 1.199(**5**), O2–Ge‐O3 69.8(**1**); **16**: Ir–Sn 2.5734(**2**), Ir–P1 2.3626(**5**), Ir–P2 2.3394(5), Ir–P3 2.3101(**5**), Ir–C2 1.872(**2**), C2–O1 1.160(**3**), Sn–O2 2.093(**1**), Sn–O3 2.090(**1**), C3–O2 1.341(**2**), C3–O3 1.335(**3**), C3–O4 1.218(**3**), O2–Sn‐O3 63.7(**1**); **15**: Ir–Sn 2.4896(**2**), Ir–C2 2.123(**3**), Ir–P1 2.3134(**8**), Ir–P2 2.2859(**8**), Ir–P3 2.2697(**8**), C2–O2 1.368(**3**), C2–O1 1.215(**3**), O2–C2‐O1 118.4(**3**), Ir–C2–O1 129.2(**2**), Ir–C2–O2 112.5(**2**), C1–Sn–Ir 170.0(**1**), P1–Ir–C2 171.0(**1**).

The molecular structures of the CO_2_ reaction products **12** and **16** are depicted in Figure 5 (see Supporting Information for the molecular structures of **11**, **12**, **14**). This type of reaction has already been observed for the homologous cobalt stannylidyne [(Me_3_P)_3_Co≡SnTbb].^[^ ^37^ ^]^ The carbonate–germanium coordination in **11** and **12** is a seldom case for such a structural motif and has only been documented in [(Eind)_2_GeO_2_CO] [Ge–O 1.8588(9), C–O 1.3563(13), C═O 1.193(2) Å], which shows almost similar interatomic distances for the germanium carbonate coordination (Eind: 1,1,3,3,5,5,7,7‐octaethyl‐s‐hydrindacen‐4‐yl).^[^ ^91^ ^]^ The Ir–Ge bond length in **12** of 2.4092(4) Å corresponds to a single bond between these elements.^[^ ^92^, ^93^ ^]^ The CO coordination in **12** was also characterized by IR spectroscopy and shows a signal at 1919 cm^−1^. The carbonate coordination at tin in **14** and **16** is comparable with compound [(Me_3_P)_3_(CO)Co‐Sn(O_2_CO)Tbb], exhibiting almost similar interatomic distances of the SnO_2_CO unit.^[^ ^37^ ^]^ Carbonate coordination at tin was also observed in [{2,6‐(Me_2_NCH_2_)_2_C_6_H_3_}‐(Ph)SnCO_3_] and [Ni_3_(*μ*‐PPh_2_CH_2_PPh_2_)_3_(*μ*_3_‐Cl)(*μ*_3_‐Sn(OH)(η^5^‐O_2_CO))].^[^ ^94^, ^95^ ^]^ As a presumed intermediate of this CO_2_ reaction, a product of a [2+2] cycloaddition was discussed.^[^ ^37^ ^]^ After optimization of the reaction conditions, the products of a single CO_2_ cycloaddition (**13**, **15**) have been isolated in the cases of the stannylidynes **4** and **5** (Scheme 6). This type of addition product has already been documented in the case of CO_2_ addition at germylene complex [(Et_3_P)_2_Pt═Ge{N(SiMe_3_)_2_}_2_] leading to a Pt(*μ*,*η* ^2^‐CO_2_)Ge ring structure [C–O 1.333(10), C═O 1.214(10) Å]. A similar structure was also observed in the case of a diarylketone addition to a tungsten‐silicon triple bond.^[^ ^41^, ^96^ ^]^ In complexes **13** (M = Rh) and **15** (M = Ir) four‐membered [M(*μ*,*η* ^2^‐CO_2_)Sn] cycles are formed exhibiting similar C–O interatomic distances in comparison to the PtGe literature example (vide supra) [**13**: C2–O2 1.359(3), C2–O1 1.218(3), **15**: C2–O2 1.368(3), C2–O1 1.215(3), Å (see Figure 5)]. The Ir–Sn [2.4896(2) Å] and Rh–Sn [2.4896(2) Å] interatomic distances are of the same length, which could be due to steric restraint in the four‐membered SnMCO‐ring (M = Rh, Ir). The Ir–Sn bond length of the addition product **15** indicates a very short single bond.^[^ ^53^, ^97^ ^]^ The Rh–Sn bond length in complex **13** is comparable with known single bonds between these elements.^[^ ^98^ ^]^

At room temperature, two signals for the repective *trans* and *cis* PMe_3_ ligands in relation to the tin atom were observed in the ^31^P{^1^H} NMR spectra of the CO_2_ addition products **13** and **15**. Due to ring formation, the pentacoordinate complexes **13** and **15** do not show chemical exchange of the PMe_3_ ligands at room temperature. The bonding situation in complex **13** was evaluated by DFT calculations and NBO analysis.^[^ ^56^, ^57^, ^58^, ^59^, ^60^, ^61^, ^62^, ^63^, ^64^, ^65^, ^66^, ^67^ ^]^ One electron pair is almost completely localized at the tin atom, and one of the filled d‐orbitals of the rhodium atom is slightly distributed (7%) into an unoccupied p‐orbital of the tin atom. These findings are congruent with the found interatomic distances between the transition metal and the tetrylene. The CO_2_ reaction was also studied for the plumbylidynes **6** and **7**, since an analogous oxidation of the lead would not be favorable. Due to the formation of at least two products in the case of compound **6**, the isolation of a reaction product was not straightforward. However, low‐quality crystals of a reaction product were isolated featuring a bridging carbonate between lead(II) and a square planar coordinated rhodium(I) [*trans*‐(Me_3_P)_2_(CO)Rh(*μ*‐OCO_2_)PbAr*]. Thus, instead of oxidizing the lead atom, the rhodium is oxidized from Rh(−I) to Rh(+I).

## Conclusions

In conclusion, a straightforward one‐pot procedure for synthesizing up to 1 g of Group 9 tetrylidynes has been presented. A hitherto unknown metathetical exchange to transfer plumbylidynes of Co, Rh, and Ir to the respective stannylidynes and the stannylidynes to the germylidynes is established. The stepwise reaction of tetrylidynes with carbon dioxide is discussed, and products of a [2+2] cycloaddition of CO_2_ are presented. The facile syntheses of tetrylidynes enable a systematic study of their chemical properties, which will be presented in the near future.

## Supporting Information

The authors have cited additional references within the Supporting Information.^[^ ^99^, ^100^, ^101^, ^102^, ^103^, ^104^, ^105^, ^106^, ^107^, ^108^, ^109^, ^110^, ^111^, ^112^ ^]^

## Conflict of Interests

The authors declare no conflict of interest.

## Supporting information



Supporting information

## Data Availability

The data that support the findings of this study are available in the Supporting Information of this article.
