# Supplementary material for: Metathetical Exchange, Synthesis, and Carbon Dioxide Addition of Higher Homologues of Group 9 Metal Carbynes
Source: Angew Chem Int Ed Engl. 2025 May 23;64(27):e202503930. doi: 10.1002/anie.202503930 (PMC12207358; doi:10.1002/anie.202503930)
Supplement: Supplementary file 1 — Supporting information [file ANIE-64-e202503930-s001.pdf]

Supporting Information

Lennart G. Holzapfel[‡], Johanna Manegold[‡], Stefan F. Clewing, Hartmut Schubert, Klaus Eichele, Lars Wesemann*

Metathetical Exchange, Synthesis and Carbon Dioxide Addition of Higher Homologues of Group 9 Metal Carbynes

Content

Experimental........2
Syntheses........4
Data of crystal structure determinations........12
NMR Spectroscopy........20
NMR spectra of Ar*Ge≡Co(PMe_3)$_3$ (**1**)........20
NMR spectra of Ar*Ge≡Rh(PMe_3)$_3$ (**2**)........23
NMR spectra of Ar*Ge≡Ir(PMe_3)$_3$ (**3**)........27
NMR spectra of Ar*Sn≡Rh(PMe_3)$_3$ (**4**)........30
NMR spectra of Ar*Sn≡Ir(PMe_3)$_3$ (**5**)........35
NMR spectra of Ar*Pb≡Rh(PMe_3)$_3$ (**6**)........39
NMR spectra of Ar*Pb≡Ir(PEt_3)$_3$ (**7**)........43
NMR spectra of [Rh(PMe_3)$_4$][Ar*$GeCl_2$] (**8**)........47
NMR spectra of [Rh(PMe_3)$_4$][Ar*$SnCl_2$] (**9**)........50
NMR spectra of [Ir(PMe_3)$_4$][Ar*$SnCl_2$] (**10**)........54
NMR spectra of [Ar*Ge(CO_3)Rh(CO)(PMe_3)$_3$] (**11**)........58
NMR spectra of [Ar*Ge(CO_3)Ir(CO)(PMe_3)$_3$] (**12**)........61
NMR spectra of [Ar*Sn(μ-CO_2)Rh(PMe_3)$_3$] (**13**)........64
NMR spectra of [Ar*Sn(CO_3)Rh(CO)(PMe_3)$_3$] (**14**)........68
NMR spectra of [Ar*Sn(μ-CO_2)Ir(PMe_3)$_3$] (**15**)........72
NMR spectra of [Ar*Sn(CO_3)Ir(CO)(PMe_3)$_3$] (**16**)........76
NMR spectra of the metathesis reactions........79
IR spectroscopy.........83
UV-Vis spectroscopy.........89
Quantum chemical calculations........96
Optimized structures........99
References........119

Experimental

General information. All manipulations were performed under argon (99.999 %) atmosphere using standard Schlenk techniques or an *MBraun* Glovebox. Additionally, the argon for Schlenk techniques is dried through SICAPENT®. All solvents were degassed via three “freeze-pump-thaw” cycles and dried with diverse methods. *n*-Hexane and *n*-pentane were obtained from an *MBraun* solvent purification system (SPS). Diethyl ether, tetrahydrofuran and toluene were obtained from an *MBraun* solvent purification system (SPS) and analogous to benzene-d_6 and toluene-d_8 distilled from a Na/K alloy. Benzene, benzene-d_6 and *o*-difluorobenzene were dried over activated aluminium oxide. $[Ar^*EX]_2$ (E = Ge, X = Cl; E = Sn, X = Cl; E = Pb, X = Br) (Ar* = $(2,6\text{-Trip})_2C_6H_3$, Trip = 2,4,6- *i*$Pr_3C_6H_2$),[1] $Co(PMe_3)_4$,[2] $Rh(PMe_3)_4Cl$,[3] $Ir(PMe_3)_4Cl$,[4] and $Ir(PEt_3)_4Cl$ were synthesized following the respective literature.[5] Commercially purchased chemicals (*Sigma Aldrich*, *ABCR, Fisher Scientific, TCI Chemicals*) were used as received. All air and moisture sensitive chemicals were stored under argon atmosphere.

NMR spectroscopy. The NMR spectra were recorded with one of the following spectrometers with the respective equipment:

- *Bruker* Avance III HD 300 NanoBay, 5 mm BBFO probe head operating at 121.49 (^{31}P), 111.92 (^{119}Sn) MHz.
- *Bruker* AVII+400, 5 mm QNP (quad nucleus probe) head (for ^{1}H, ^{13}C, ^{31}P spectra) or a 5 mm H-1,X+F-19 BBFO ATM head (Quad Systems), operating at 400.11 (^{1}H), 100.62 (^{13}C),161.97 (^{31}P), 149.20 (^{119}Sn) MHz.
- *Bruker* AVII+500, 10 mm low gamma BBO probe head (^{103}Rh), 500.13 (^{1}H), 15.94 (^{103}Rh) MHz.
- *Bruker* Avance III HDX 600, variable temperature set up, 5 mm Prodigy BBO Cryo probe head, operating at 600.13 (^{1}H), 150.92 (^{13}C), 242.94 (^{31}P), 223.79 (^{119}Sn), 126.26 (^{207}Pb) MHz.
- *Bruker* Avance III HDX 700, 5 mm Prodigy TCI Cryo probe head, operating at 700.21 (^{1}H), 176.07 (^{13}C) MHz.

The chemical shifts are reported as δ in ppm relative to the following external standards: tetramethylsilane (^{1}H, ^{13}C), 85% phosphoric acid (^{31}P), rhodium(III)acetylacetonate (^{103}Rh), tetramethyl tin (^{119}Sn) and tetramethyl lead (^{207}Pb).[6] The chemical shifts were referenced using the chemical shift of the solvents ^{2}H resonance frequency as followes: Ξ = 25.145020 % for ^{13}C, Ξ = 40.480742 for ^{31}P, Ξ = 3.186447 for ^{103}Rh, Ξ = 37.290632 % for ^{119}Sn and Ξ = 20.920599 % for ^{207}Pb.[6] The multiplicity of the signals is abbreviated as s = singlet, d = doublet, t = triplet, q = quartet, sept = septet and m = multiplet or br. = broad/unresolved. The proton and carbon signals were assigned via detailed analysis of ^{1}H, ^{13}C{^{1}H}-(UDEFT), ^{13}C{^{1}H}-DEPT135, ^{1}H-^{1}H-COSY, ^{1}H-^{13}C-HSQC and ^{1}H-^{13}C-HMBC NMR spectra. Selected 1D-NMR spectra of the compounds can be found in the Supporting Information.

IR spectroscopy. The IR spectra were recorded with a Bruker VERTEX 70 IR spectrometer using an ATR unit.

Crystal structure determination. X-ray data were collected with a *Bruker* Smart APEX II diffractometer with graphite monochromated Mo-K_α radiation. The used programs were Bruker APEX2 v2011.8-0 including SADABS for absorption correction, SAINT for data reduction, SHELXS for structure solution and SHELXLE or WinGX suite of programs v1.70.01 including SHELXL for

structure refinement.[7] All details of the structure refinement and solution can be found in the Supporting Information.

UV/Vis Spectroscopy. Visible UV/Vis absorption spectra were recorded on PerkinElmer Lambda 35 spectrophotometer in gas tight 1 cm quartz cuvettes sealed by Teflon stoppers or Teflon lined screw caps.

Elemental Analysis. The elemental analysis was performed at the Department for Inorganic Chemistry at the University of Tübingen using a vario MICRO CUBE or UNICUBE by *elementar*.

Syntheses

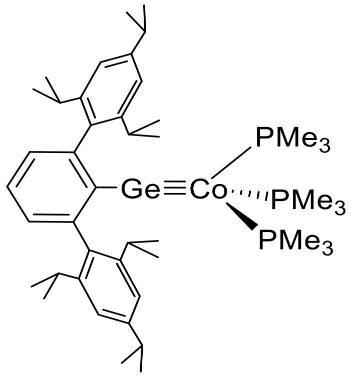

[Co≡Ge] **1**: $Co(PMe_3)_4$ (125 mg, 344 µmol, 1.00 equiv.) and $[Ar^*GeCl]_2$ (203 mg, 172 µmol, 0.50 equiv.) are weighed in together, suspended in toluene (4.00 ml) at room temperature and stirred for twenty minutes. Subsequently, KC_8 (51.2 mg, 378 µmol, 1.10 equiv.) is added to the green reaction mixture which turns darker green upon addition. After stirring for one hour the solvent is removed under reduced pressure and the solid is coevaporated with *n*-pentane (2.00 ml). The product is extracted with *n*-pentane (4.00 ml) which is removed under reduced pressure after filtration. The product is crystallized from a highly saturated *o*-difluorobenzene solution at −40 °C to yield $Ar^*Ge{\equiv}Co(PMe_3)_3$ (**1**) (97.4 mg, 116 mmol, 34%) as dark green crystals suitable for X-ray diffraction. **^{1}H-NMR** [400.11 MHz, 300.0 K, benzene-d_6]: δ (ppm) = 1.13–1.17 (m, 27H, P(C$\underline{H}_3$)$_3$), 1.19 (d, 12H, $^3J_{H\text{-}H}$ = 6.8 Hz, *o*-^{i}Pr-C$\underline{H}_3$), 1.35 (d, 12H, $^3J_{H\text{-}H}$ = 7.0 Hz, *p*-^{i}Pr-C$\underline{H}_3$), 1.51 (d, 12H, $^3J_{H\text{-}H}$ = 7.0 Hz, *o*-^{i}Pr-C$\underline{H}_3$), 2.91 (sept, 2H, $^3J_{H\text{-}H}$ = 6.9 Hz, *p*-^{i}Pr-C$\underline{H}$), 3.14 (sept, 4H, $^3J_{H\text{-}H}$ = 6.9 Hz, *o*-^{i}Pr-C$\underline{H}$), 6.91–6.97 (m, 2H, *m*-C$_6\underline{H}_3$), 7.18 (s, 4H, *m*-$\underline{H}_{trip}$), 7.26–7.33 (m, 1H, *p*-C$_6\underline{H}_3$). **^{13}C{^{1}H}-NMR** [100.62 MHz, 300.0 K, benzene-d_6]: δ (ppm) = 24.1 (s, *o*-^{i}Pr-$\underline{C}$H$_3$), 24.3 (s, *p*-^{i}Pr-$\underline{C}$H$_3$), 24.9 (s, *o*-^{i}Pr-$\underline{C}$H$_3$), 27.6–27.9 (m, P($\underline{C}$H$_3$)$_3$), 30.7 (s, *o*-^{i}Pr-$\underline{C}$H), 34.5 (s, *p*-^{i}Pr-$\underline{C}$H), 120.9 (s, *m*-$\underline{C}_{trip}$), 126.0 (s, *p*-$\underline{C}_6$H$_3$), 129.0 (s, *m*-$\underline{C}_6$H$_3$), 138.5 (s, *i*-$\underline{C}_{trip}$), 142.0 (s, *o*-$\underline{C}_6$H$_3$), 146.2 (s, *o*-$\underline{C}_{trip}$), 147.5 (s, *p*-$\underline{C}_{trip}$), 169.8 (s, *i*-$\underline{C}_6$H$_3$–Ge). **^{31}P{^{1}H}-NMR** [161.97 MHz, 300.0 K, benzene-d_6]: δ (ppm) = 14.4 (s, $\underline{P}$Me$_3$). **Elemental analysis** calculated for $C_{45}H_{76}CoGeP_3$ (%): C 64.23, H 9.10. Found: 63.87, H 8.99. **UV/Vis** (*n*-pentane, c = 0.048 mmol·L^{-1}): λ (nm) [ε (L·mol^{-1}·cm^{-1})]: 433 [5145], 323 [27675], 283 [26158].

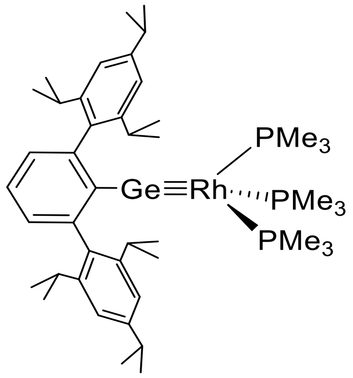

[Rh≡Ge] **2**: $[Ar^*GeCl]_2$ (504 mg, 42.8 µmol, 0.50 equiv.) and $Rh(PMe_3)_4Cl$ (378 mg, 85.8 µmol, 1.00 equiv.) are weighed in together, suspended in toluene (15.0 ml) and stirred for two hours at room temperature, upon which a red reaction mixture is obtained. Subsequently, KC_8 (254 mg, 1.88 mmol, 2.20 equiv.) is added and the now violet-brown reaction mixture is stirred for further two hours at room temperature. All volatile components are removed under reduced pressure and the solid is coevaporated with *n*-pentane (2.00 ml). The product is extracted with toluene (3x5.00 ml) and filtered. The highly concentrated filtrate is stored at –40 °C for crystallization to yield dark purple crystals of $Ar^*Ge{\equiv}Rh(PMe_3)_3$ (**2**) (487 mg, 55.0 µmol, 64%). The crystals are suitable for X-ray diffraction analysis. **^{1}H-NMR** [400.10 MHz, 298.0 K, benzene-d_6]: δ (ppm) = 1.17–1.25 (m, 39H, *o*-^{i}Pr-C$\underline{H}_3$ + P(C$\underline{H}_3$)$_3$), 1.36 (d, 12H, $^3J_{H\text{-}H}$ = 6.9 Hz, *p*-^{i}Pr-C$\underline{H}_3$), 1.54 (d, 12H, $^3J_{H\text{-}H}$ = 6.9 Hz, *o*-^{i}Pr-C$\underline{H}_3$), 2.92 (sept, 2H, $^3J_{H\text{-}H}$ = 6.9 Hz, *p*-^{i}Pr-C$\underline{H}$), 3.20 (sept, 4H, $^3J_{H\text{-}H}$ = 6.9 Hz, *o*-^{i}Pr-C$\underline{H}$), 7.00–7.04 (m, 2H, *m*-C$_6\underline{H}_3$), 7.18–7.25 (m, 5H, *m*-$\underline{H}_{trip}$ + *p*-C$_6\underline{H}_3$). **^{13}C{^{1}H}-NMR** [100.61 MHz, 298.0 K, benzene-d_6]: δ (ppm) = 24.7 (*p*-^{i}Pr-$\underline{C}$H$_3$), 24.9 (*o*-^{i}Pr-$\underline{C}$H$_3$), 25.3 (*o*-^{i}Pr-$\underline{C}$H$_3$), 29.3–29.8 (br. m, P($\underline{C}$H$_3$)$_3$), 31.2 (*o*-^{i}Pr-$\underline{C}$H), 34.8 (*p*-^{i}Pr-$\underline{C}$H), 121.1 (*m*-$\underline{C}_{trip}$), 127.2 (*p*-$\underline{C}_6$H$_3$), 129.2 (*m*-$\underline{C}_6$H$_3$), 137.5 (*i*-$\underline{C}_{trip}$), 142.4 (br. m, $^3J_{Rh\text{-}C}$ = 3.4 Hz, *o*-$\underline{C}_6$H$_3$), 147.2 (*o*-$\underline{C}_{trip}$), 148.0 (*p*-$\underline{C}_{trip}$), 169.9 (d, $^2J_{Rh\text{-}C}$ = 18.5 Hz, *i*-$\underline{C}_6$H$_3$). **^{31}P{^{1}H}-NMR** [161.97 MHz, 298.0 K, benzene-d_6]: δ (ppm) = −0.37 (d, $^1J_{Rh\text{-}P}$ = 176.7 Hz). **^{103}Rh{^{1}H}-NMR** [15.94 MHz, 299.2 K, benzene-d_6]: δ (ppm) = −8353.9 (q, $^1J_{Rh\text{-}P}$ = 177.0 Hz). **Elemental analysis** calculated for $C_{45}H_{76}GeP_3Rh$ + 0.5 C_7H_8 (%): C 62.53, H 8.66. Found: C 62.59, H 8.95. **UV/Vis** (*n*-pentane, c = 0.045 mmol·L^{-1}): λ (nm) [ε (L·mol^{-1}·cm^{-1})]: 548 [3525], 414 [14441].

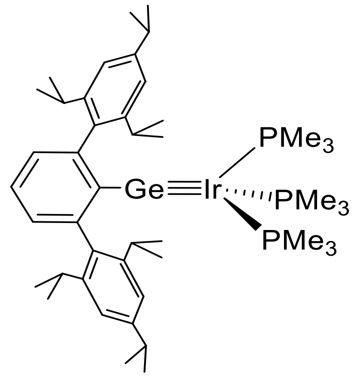

[Ir≡Ge] **3**: $Ir(PMe_3)_4Cl$ (500 mg, 0.94 mmol, 1.00 equiv.) and $[Ar^*GeCl]_2$ (554 mg, 0.47 mmol, 0.50 equiv.) are weighed in together, suspended in toluene (12.0 ml) at room temperature and stirred for one hour, upon which a dark orange reaction mixture is obtained. Subsequently, KC_8 (280 mg, 2.07 mmol, 2.20 equiv.) is added to the orange reaction mixture which turns dark violet upon addition. After stirring for three hours the solvent is removed under reduced pressure and the solid is coevaporated with *n*-pentane (3.00 ml). The product is extracted with *n*-pentane (6.00 ml) which is removed under reduced pressure after filtration. The product can be crystallized from a highly saturated toluene solution at −40 °C to yield Ar*Ge≡Ir$(PMe_3)_3$ (**3**) (758 mg, 0.78 mmol, 83%) as dark violet crystals suitable for X-ray diffraction. **^{1}H-NMR** [400.11 MHz, 300.0 K, benzene-*d*$_6$]: δ (ppm) = 1.21 (d, 12H, $^3J_{H\text{-}H}$ = 6.9 Hz, *o*-^{i}Pr-C$\underline{H}_3$), 1.34 (d, 12H, $^3J_{H\text{-}H}$ = 6.9 Hz, *p*-^{i}Pr-C$\underline{H}_3$), 1.38–1.45 (m, 27H, P(C$\underline{H}_3$)$_3$), 1.55 (d, 12H, $^3J_{H\text{-}H}$ = 6.9 Hz, *o*-^{i}Pr-C$\underline{H}_3$), 2.90 (sept, 2H, $^3J_{H\text{-}H}$ = 6.9 Hz, *p*-^{i}Pr-C$\underline{H}$), 3.17 (sept, 4H, $^3J_{H\text{-}H}$ = 6.9 Hz, *o*-^{i}Pr-C$\underline{H}$), 7.03–7.06 (m, 2H, *m*-C$_6\underline{H}_3$), 7.19 (s, 4H, *m*-$\underline{H}_{trip}$), 7.23–7.28 (m, 1H, *p*-C$_6\underline{H}_3$). **^{13}C{^{1}H}-NMR** [100.62 MHz, 300.0 K, benzene-*d*$_6$]: δ (ppm) = 24.2–24.6 (m, *o*-^{i}Pr-$\underline{C}$H$_3$ + *p*-^{i}Pr-$\underline{C}$H$_3$), 24.9 (s, *o*-^{i}Pr-$\underline{C}$H$_3$), 30.9 (s, *o*-^{i}Pr-$\underline{C}$H), 32.0–32.3 (m, P($\underline{C}$H$_3$)$_3$), 34.5 (s, *p*-^{i}Pr-$\underline{C}$H), 120.7 (s, *m*-$\underline{C}_{trip}$), 126.6 (s, *p*-$\underline{C}_6$H$_3$), 129.3 (s, *m*-$\underline{C}_6$H$_3$), 137.2 (s, *i*-$\underline{C}_{trip}$), 142.0 (s, *o*-$\underline{C}_6$H$_3$), 146.7 (s, *o*-$\underline{C}_{trip}$), 147.7 (s, *p*-$\underline{C}_{trip}$), 171.8 (s, *i*-$\underline{C}_6$H$_3$–Ge). **^{31}P{^{1}H}-NMR** [161.97 MHz, 300.0 K, benzene-*d*$_6$]: δ (ppm) = −37.1 (s, $\underline{P}$Me3). **Elemental analysis** calculated for $C_{45}H_{76}GeIrP_3$ (%): C 55.44, H 7.86. Found: C 55.71, H 7.75. **UV/Vis** (*n*-pentane, c = 0.041 mmol·L^{-1}): λ (nm) [ε (L·mol^{-1}·cm^{-1})]: 511 [5595], 380 [24220].

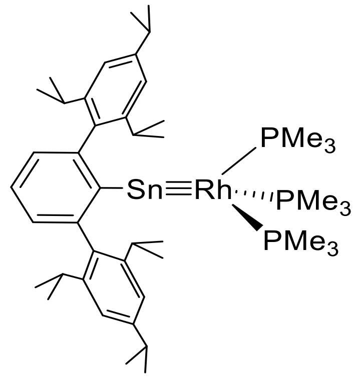

[Rh≡Sn] **4**: $[Ar^*SnCl]_2$ (560 mg, 44.1 µmol, 0.50 equiv.) and $Rh(PMe_3)_4Cl$ (390 mg, 88.1 µmol, 1.00 equiv.) are weighed together, suspended in toluene (15.0 ml) and stirred for two hours at room temperature upon which a violet reaction mixture is obtained. Subsequently, KC_8 (254 mg, 1.88 mmol, 2.20 equiv.) is added and the now green-brown reaction mixture is stirred at room temperature for two hours. All volatile components are removed under reduced pressure and the remaining solid is coevaporated with *n*-pentane (2.00 ml). The product is extracted with toluene (3x5.00 ml) and filtered. Dark green crystals of Ar*Sn≡Rh$(PMe_3)_3$ (**4**) (512 mg, 55.0 µmol, 62%) are obtained from the highly concentrated filtrate at –40 °C. The crystals are suitable for X-ray diffraction analysis. **^{1}H-NMR** [400.10 MHz, 300.0 K, benzene-*d*$_6$]: δ (ppm) = 1.22 (d, 12H, $^3J_{H\text{-}H}$ = 6.9 Hz, *o*-^{i}Pr-C$\underline{H}_3$), 1.30–1.33 (m, 27H, P(C$\underline{H}_3$)$_3$), 1.35 (d, 12H, $^3J_{H\text{-}H}$ = 6.9 Hz, *p*-^{i}Pr-C$\underline{H}_3$), 1.53 (d, 12H, $^3J_{H\text{-}H}$ = 6.9 Hz, *o*-^{i}Pr-C$\underline{H}_3$), 2.92 (sept, 2H, $^3J_{H\text{-}H}$ = 6.9 Hz, *p*-^{i}Pr-C$\underline{H}$), 3.24 (sept, 4H, $^3J_{H\text{-}H}$ = 6.9 Hz, *o*-^{i}Pr-C$\underline{H}_3$), 7.18 (m, 2H, *m*-C$_6\underline{H}_3$, overlapping with solvent signal), 7.20 (s, 4H, *m*-$\underline{H}_{trip}$), 7.27–7.33 (m, 1H, *p*-C$_6\underline{H}_3$). **^{13}C{^{1}H}-NMR** [100.61 MHz, 300.0 K, benzene-*d*$_6$]: δ (ppm) = 24.1–24.3 (br., *o*-^{i}Pr-$\underline{C}$H$_3$ + *p*-^{i}Pr-$\underline{C}$H$_3$), 25.0 (*o*-^{i}Pr-$\underline{C}$H$_3$), 30.6 (*o*-^{i}Pr-$\underline{C}$H), 30.8–31.1 (br. m, P($\underline{C}$H$_3$)$_3$), 34.4 (*p*-^{i}Pr-$\underline{C}$H), 120.9 (*m*-$\underline{C}_{trip}$), 126.4 (*p*-$\underline{C}_6$H$_3$), 129.1 (*m*-$\underline{C}_6$H$_3$), 137.5 (*i*-$\underline{C}_{trip}$), 143.0 (*o*-$\underline{C}_6$H$_3$), 146.9 (*o*-$\underline{C}_{trip}$), 147.9 (*p*-$\underline{C}_{trip}$), 187.9 (d, $^2J_{Rh\text{-}C}$ = 15.1 Hz, *i*-$\underline{C}_6$H$_3$). **^{31}P{^{1}H}-NMR** [161.97 MHz, 300.0 K, benzene-*d*$_6$]: δ (ppm) = 5.71 (d + sat., $^1J_{Rh\text{-}P}$ = 190.2 Hz, $^2J_{119/117Sn\text{-}P}$ = 295.4 Hz). **^{103}Rh{^{1}H}-NMR** [15.94 MHz, 300.0 K, benzene-*d*$_6$]: δ (ppm) = –8230.4 (q, $^1J_{Rh\text{-}P}$ = 189.4 Hz). **^{119}Sn{^{1}H}-NMR** [149.20 MHz, 300.0 K, benzene-*d*$_6$]: δ (ppm) = 1113.4 (dq, $^1J_{119Sn\text{-}Rh}$ = 906.4 Hz, $^2J_{119Sn\text{-}P}$ = 264.0 Hz). **Elemental analysis** calculated for $C_{45}H_{76}P_3RhSn$ + 0.5 C_7H_8 (%): C 59.58, H 8.25. Found: C 59.74, H 8.205. **UV/Vis** (*n*-pentane, c = 0.043 mmol·L^{-1}): λ (nm) [ε (L·mol^{-1}·cm^{-1})]: 441 [6168], 280 [16720].

[Ir≡Sn] **5**: $Ir(PMe_3)_4Cl$ (150 mg, 0.28 mmol, 1.00 equiv.) and $[Ar^*SnCl]_2$ (179 mg, 0.14 mmol, 0.50 equiv.) are weighed in together, suspended in toluene (5.00 ml) at room temperature and stirred for one hour, upon which a dark red reaction mixture is obtained. Subsequently, KC_8 (84.8 mg, 0.63 mmol, 2.20 equiv.) is added to the orange reaction mixture which turns dark brown upon addition. After stirring for two hours the solvent is removed under reduced pressure and the solid is coevaporated with *n*-pentane (1.00 ml). The product is extracted with *n*-pentane (2.00 ml) which is removed under reduced pressure after filtration to yield $Ar^*Sn{\equiv}Ir(PMe_3)_3$ (**5**) (256 mg, 0.25 mmol, 89%) as a brown powder. $Ar^*Sn{\equiv}Ir(PMe_3)_3$ can be crystallized from a highly saturated toluene solution at −40 °C to yield dark brown crystals suitable for X-ray diffraction. **^{1}H-NMR** [600.13 MHz, 298.0 K, benzene-d_6]: δ (ppm) = 1.21 (d, 12H, $^3J_{H\text{-}H}$ = 6.8 Hz, *o*-^{i}Pr-C$\underline{H}_3$), 1.35 (d, 12H, $^3J_{H\text{-}H}$ = 6.9 Hz, *p*-^{i}Pr-C$\underline{H}_3$), 1.49–1.53 (m, 27H, P(C$\underline{H}_3$)$_3$), 1.54 (d, 12H, $^3J_{H\text{-}H}$ = 6.9 Hz, *o*-^{i}Pr-C$\underline{H}_3$), 2.91 (sept, 2H, $^3J_{H\text{-}H}$ = 6.9 Hz, *p*-^{i}Pr-C$\underline{H}$), 3.21 (sept, 4H, *o*-^{i}Pr-C$\underline{H}$, $^3J_{H\text{-}H}$ = 6.9 Hz), 7.18–7.20 (m, 2H, *m*-C$_6\underline{H}_3$), 7.21 (s, 4H, *m*-$\underline{H}_{trip}$), 7.31–7.34 (m, 1H, *p*-C$_6\underline{H}_3$). **^{13}C{^{1}H}-NMR** [150.92 MHz, 298.0 K, benzene-d_6]: δ (ppm) = 23.3 (s, *o*-^{i}Pr-$\underline{C}$H$_3$), 23.5 (s, *p*-^{i}Pr-$\underline{C}$H$_3$), 24.3 (s, *o*-^{i}Pr-$\underline{C}$H$_3$), 29.8 (s, *o*-^{i}Pr-$\underline{C}$H), 33.3–33.6 (m, P($\underline{C}$H$_3$)$_3$ + *p*-^{i}Pr-$\underline{C}$H), 120.1 (s, *m*-$\underline{C}_{trip}$), 125.5 (s, *p*-$\underline{C}_6$H$_3$), 128.8 (s, *m*-$\underline{C}_6$H$_3$), 136.7–136.7 (m, *i*-$\underline{C}_{trip}$), 142.1 (s, *o*-$\underline{C}_6$H$_3$), 146.1 (s, *o*-$\underline{C}_{trip}$), 147.2 (s, *p*-$\underline{C}_{trip}$), 187.5 (q, *i*-$\underline{C}_6$H$_3$–Sn, $^2J_{P\text{-}C}$ = 5.0 Hz). **^{31}P{^{1}H}-NMR** [161.97 MHz, 300.0 K, benzene-d_6]: δ (ppm) = −34.9 (s + sat., $\underline{P}$Me$_3$, $^2J_{119/117Sn\text{-}P}$ = 520.7 Hz). **^{119}Sn{^{1}H}-NMR** [112.03 MHz, 300.0 K, benzene-d_6]: δ (ppm) = 907.5 (q, $^2J_{Sn\text{-}P}$ = 533.5 Hz). **Elemental analysis** calculated for $C_{45}H_{76}SnIrP_3$ + 0.5 C_7H_8 (%): C 54.59, H 7.56. Found: C 54.83, H 7.59. **UV/Vis** (*n*-pentane, c = 0.039 mmol·L^{-1}): λ (nm) [ε (L·mol^{-1}·cm^{-1})]: 414 [15926], 273 [34913].

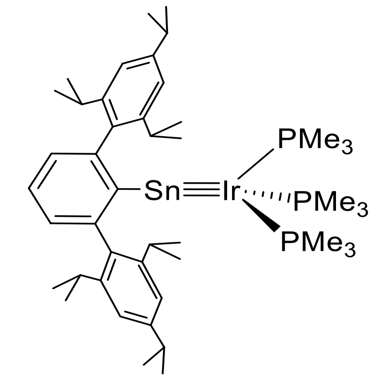

[Rh≡Pb] **6**: *Annotation: Due to the light sensibility of many lead compounds, this synthesis was performed in the dark.* A solution of $[Ar^*PbBr]_2$ (60.0 mg, 39.0 µmol, 0.50 equiv.) in cold toluene (0.5 ml, −35°C) is dripped into a suspension of $Rh(PMe_3)_3Cl$ (34.5 mg, 78.0 µmol, 1.00 equiv.) in cold toluene (0.5 ml, –35°C). After 15 minutes at room temperature, {(^{Mes}NacNac)Mg}$_2$ (57.4 mg, 80.0 µmol, 1.03 equiv.) is added to the red suspension upon which it turns intense brown. After stirring for a further 15 minutes the solvent is removed under reduced pressure and the resulting solid is coevaporated with *n*-pentane (2 x 1.5 ml). The product is extracted with cold *n*-pentane (4 x 1.0 ml) which is removed under reduced pressure after filtration. The crude product $Ar^*Pb{\equiv}Rh(PMe_3)_3$ (**6**) (46.7 mg, 45.8 µmol, 59%) is purified by crystallization from a highly concentrated toluene solution at –40 °C to yield dark brown crystals. The crystals are suitable for X-ray diffraction analysis. **^{1}H-NMR** [600.13 MHz, 273.0 K, toluene-d_8]: δ (ppm) = 1.21 (d, 12H, $^3J_{H\text{-}H}$ = 6.9 Hz, *o*-^{i}Pr-C$\underline{H}_3$), 1.35 (d, 12H, $^3J_{H\text{-}H}$ = 6.9 Hz, *p*-^{i}Pr-C$\underline{H}_3$), 1.48 (d, 12H, $^3J_{H\text{-}H}$ = 6.9 Hz, *o*-^{i}Pr-C$\underline{H}_3$), 1.67–1.70 (m, 27H, P(C$\underline{H}_3$)$_3$), 2.90 (sept, 2H, $^3J_{H\text{-}H}$ = 6.9 Hz, *p*-^{i}Pr-C$\underline{H}$), 3.34 (sept, 4H, $^3J_{H\text{-}H}$ = 6.9 Hz, *o*-^{i}Pr-C$\underline{H}_3$), 7.15 (s, 4H, *m*-$\underline{H}_{trip}$), 7.43 (t, 1H, *p*-C$_6\underline{H}_3$), 7.59 (d, 2H, *m*-C$_6\underline{H}_3$). **^{13}C{^{1}H}-NMR** [150.92 MHz, 273.0 K, toluene-d_8]: δ (ppm) = 23.9 (*o*-^{i}Pr-$\underline{C}$H$_3$), 24.7 (*p*-^{i}Pr-$\underline{C}$H$_3$), 25.2 (*o*-^{i}Pr-$\underline{C}$H$_3$), 30.7 (*o*-^{i}Pr-$\underline{C}$H), 34.6–34.9 (m, P($\underline{C}$H$_3$)$_3$ + *p*-^{i}Pr-$\underline{C}$H), 121.2 (*m*-$\underline{C}_{trip}$), 125.4 (*p*-$\underline{C}_6$H$_3$), 134.6 (*m*-$\underline{C}_6$H$_3$), 139.1 (*i*-$\underline{C}_{trip}$), 143.2 (*o*-$\underline{C}_6$H$_3$), 146.5 (*o*-$\underline{C}_{trip}$), 147.9 (*p*-$\underline{C}_{trip}$), 272.1 (d, $^2J_{Rh\text{-}C}$ = 14.9 Hz, *i*-$\underline{C}_6$H$_3$). **^{31}P{^{1}H}-NMR** [242.94 MHz, 298.0 K, toluene-d_8]: δ (ppm) = 59.1 (d + sat., $^1J_{Rh\text{-}P}$ = 203 Hz, $^2J_{Pb\text{-}P}$ = 81 Hz). **^{207}Pb{^{1}H}-NMR** [126.26 MHz, 298.0 K, toluene-d_8]: δ (ppm) = 5470 (br. d, $^1J_{Pb\text{-}Rh}$ = 1040 Hz). **Elemental analysis** calculated for $C_{45}H_{76}P_3PbRh$ + C_7H_8 (%): C 56.15, H 7.61. Found: C 56.14, H 7.11. **UV/Vis** (*n*-pentane, c = 0.047 mmol·L^{-1}): λ (nm) [ε (L·mol^{-1}·cm^{-1})]: 455 [13348], 370 [11115], 296 [42057].

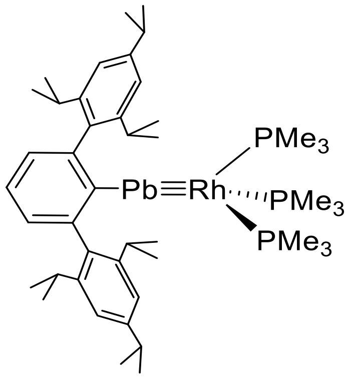

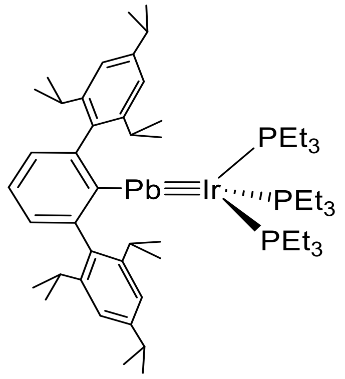

[Ir≡Pb] **7**: *Annotation: Due to the light sensibility of many lead compounds, this synthesis was performed in the dark.* $Ir(PEt_3)_3Cl$ (31.8 mg, 54.6 µmol, 1.00 equiv.) and $[Ar^*PbBr]_2$ (42.0 mg, 27.3 µmol, 0.50 equiv.) are weighed together, suspended in cold toluene (2.5 ml, −35 °C) at room temperature and stirred for 20 minutes to yield a red solution. Subsequently, $\{(^{Mes}NacNac)Mg\}_2$ (41.2 mg, 57.4 µmol, 1.05 equiv.) is added to the red solution which turns dark brown upon addition. After stirring for 30 minutes the solvent is removed under reduced pressure and the solid is coevaporated with *n*-pentane (2 x 1.0 ml). The product is extracted with *n*-pentane (3 x 1.5 ml) which is removed under reduced pressure after filtration. The brown crude product is purified by crystallization from a highly saturated toluene solution at –40 °C to yield $Ar^*Pb{\equiv}Ir(PEt_3)_3$ (**7**) (16.6 mg, 13.4 µmol, 25%) as brown crystals which are suitable for X-ray diffraction. **^{1}H-NMR** [600.13 MHz, 235.0 K, toluene-d_8]: δ (ppm) = 0.81–0.92 (br. m, 27H, $P(CH_2C\underline{H}_3)_3$), 1.27 (br. d, 12H, $^3J_{H\text{-}H}$ = 6.5 Hz, o-^{i}Pr-$C\underline{H}_3$), 1.41 (br. d, 12H, $^3J_{H\text{-}H}$ = 6.8 Hz, p-^{i}Pr-$C\underline{H}_3$), 1.59 (br. d, 12H, $^3J_{H\text{-}H}$ = 6.6 Hz, o-^{i}Pr-$C\underline{H}_3$), 1.65–1.74 (br. m, 18H, $P(C\underline{H}_2CH_3)_3$), 2.94 (br. sept, 2H, $^3J_{H\text{-}H}$ = 6.7 Hz, p-^{i}Pr-$C\underline{H}$), 3.40 (sept, 4H, o-^{i}Pr-$C\underline{H}$, $^3J_{H\text{-}H}$ = 6.7 Hz), 7.22 (br. s, 4H, m-$\underline{H}_{trip}$), 7.46–7.53 (br. m, 1H, p-$C_6\underline{H}_3$), 7.56–7.63 (br. m, 2H, m-$C_6\underline{H}_3$). **^{13}C{^{1}H}-NMR** [150.93 MHz, 234.9 K, toluene-d_8]: δ (ppm) = 10.3 (s, $P(CH_2\underline{C}H_3)_3$), 23.2 (s, o-^{i}Pr-$\underline{C}H_3$), 24.3 (s, p-^{i}Pr-$\underline{C}H_3$), 25.6 (s, o-^{i}Pr-$\underline{C}H_3$), 30.5 (s, o-^{i}Pr-$\underline{C}H$), 32.4–32.5 (m, $P(\underline{C}H_2CH_3)_3$), 34.6 (s, p-^{i}Pr-$\underline{C}H$), 121.0 (s, m-$\underline{C}_{trip}$), 124.1 (s, p-$\underline{C}_6H_3$), 134.9 (s, m-$\underline{C}_6H_3$), 139.5 (s, i-$\underline{C}_{trip}$), 143.2 (s, o-$\underline{C}_6H_3$), 146.1 (s, o-$\underline{C}_{trip}$), 147.2 (s, p-$\underline{C}_{trip}$), 254.8 (s, i-$\underline{C}_6H_3$–Pb). **^{31}P{^{1}H}-NMR** [121.49 MHz, 298.0 K, toluene-d_8]: δ (ppm) = 43.1 (s + sat., $^2J_{Pb\text{-}P}$ = 198.2 Hz, $\underline{P}Et_3$). **^{207}Pb{^{1}H}-NMR** [126.17 MHz, 298.0 K, toluene-d_8]: δ (ppm) = 4542.6 (br. q, $^2J_{Pb\text{-}P}$ = 197.6 Hz, FWHH = 200 Hz). **Elemental analysis** calculated for $C_{54}H_{94}IrP_3Pb$ (%): C 52.49, H 7.67. Found: C 53.05, H 7.44. **UV/Vis** (*n*-pentane, c = 0.032 mmol·L^{-1}): λ (nm) [ε (L·mol^{-1}·cm^{-1})]: 442 [12078], 294 [32988].

$$1/2\ [Ar^*ECl]_2 + [M(PMe_3)_4Cl] \longrightarrow [M(PMe_3)_4][Ar^*ECl_2]$$

8: E = Ge, M = Rh (52%)
9: E = Sn, M = Rh (69%)
10: E = Sn, M = Ir (82%)

Scheme S01: Synthesis of the salts **8**, **9**, **10**.

$[Rh(PMe_3)_4][Ar^*GeCl_2]$ (**8**): $[Ar^*GeCl]_2$ (40.0 mg, 33.9 µmol, 0.50 equiv.) and $Rh(PMe_3)_4Cl$ (30.0 mg, 67.8 µmol, 1.00 equiv.) are weighed together and suspended in toluene (2.00 ml). The intense red reaction mixture is stirred for two hours at room temperature. Subsequently, all volatile components are removed under reduced pressure and the red solid is coevaporated with *n*-pentane (2.00 ml). The product is extracted with Et_2O (3x2.00 ml) and filtered. Dark red crystals of $[Rh(PMe_3)_4][Ar^*GeCl_2]$ (**8**) (36.7 mg, 35.5 µmol, 52%) are obtained from the concentrated filtrate at –40 °C. The crystals are suitable for X-ray diffraction analysis. **^{1}H-NMR** [400.11 MHz, 299.2 K, benzene-d_6]: δ (ppm) = 0.98 (br. d, 36H, $P(C\underline{H}_3)_3$), 1.17 (br. d, 9H, $^3J_{H\text{-}H}$ = 6.9 Hz, o-^{i}Pr-$C\underline{H}_3$), 1.26–1.42 (m, 24H, o-^{i}Pr-$C\underline{H}_3$ + p-^{i}Pr-$C\underline{H}_3$), 1.50–1.63 (br, 9H, $P(C\underline{H}_3)_3$), 1.73 (br. d, 3H, o-^{i}Pr-$C\underline{H}_3$), 2.83–3.04 (m, 2H, p-^{i}Pr-$C\underline{H}$), 3.32–3.59 (br, 2H, o-^{i}Pr-$C\underline{H}$), 3.60–3.74 m, 2H, o-^{i}Pr-$C\underline{H}$), 7.18–7.33 (m, 7H, m-$C_6\underline{H}_3$ + p-$C_6\underline{H}_3$ + m-$\underline{H}_{trip}$). **^{13}C{^{1}H}-NMR** [100.61 MHz, 299.2 K, benzene-d_6]: δ (ppm) = 18.3–21.3 ($P(\underline{C}H_3)_3$), 23.9 ($P(\underline{C}H_3)_3$), 24.2 (o-^{i}Pr-$\underline{C}H_3$), 24.6 (2xp-^{i}Pr-$\underline{C}H_3$), 24.7 (o-^{i}Pr-$\underline{C}H_3$), 26.3 (2xo-^{i}Pr-$\underline{C}H_3$), 31.2 (o-^{i}Pr-$\underline{C}H$), 34.8 (p-^{i}Pr-$\underline{C}H$), 120.0 (m-$\underline{C}_{trip}$), 126.5 (m-/p-$\underline{C}_6H_3$), 130.5 (m-/p-$\underline{C}_6H_3$), 131.1 (i-$\underline{C}_{trip}$), 140.3 (o-$\underline{C}_6H_3$), 146.2 (o-$\underline{C}_{trip}$), 147.8 (o-$\underline{C}_{trip}$), 148.3 p-$\underline{C}_{trip}$),

162.6 (i-$\underline{C}_6H_3$, weak intensity, assignment due to 2D-spectra). **^{31}P{^{1}H}-NMR** [161.97 MHz, 299.2 K, benzene-d_6]: δ (ppm) = (–19.4)–(–14.4) (br). **Elemental analysis** calculated for $C_{48}H_{85}Cl_2GeP_4Rh$ (%): C 55.84, H 8.30. Found: C 57.67, H 8.16.

$[Rh(PMe_3)_4][Ar^*SnCl_2]$ (**9**): $[Ar^*SnCl]_2$ (20.7 mg, 13.6 µmol, 0.50 equiv.) and $Rh(PMe_3)_4Cl$ (14.4 mg, 32.5 µmol, 1.00 equiv.) are suspended in toluene (1.00 ml). The purple reaction mixture is stirred for two hours at room temperature. Subsequently, all volatile components are removed under reduced pressure and the red solid is coevaporated and washed with n-pentane (3.00 ml). The product is extracted with Et_2O (3x1.00 ml) and filtered. Red crystals of $[Rh(PMe_3)_4][Ar^*SnCl_2]$ (**9**) (24.2 mg, 22.4 µmol, 69%) are obtained from the concentrated filtrate. The crystals are suitable for X-ray diffraction analysis. **^{1}H-NMR** [400.10 MHz, 299.2 K, benzene-d_6]: δ (ppm) = 0.95(br, 36H, $P(C\underline{H}_3)_3$), 1.31 (br d, 12H, $^3J_{H-H}$ = 6.8 Hz, o-^{i}Pr-$C\underline{H}_3$), 1.39 (br d, 12H, $^3J_{H-H}$ = 6.8 Hz, p-^{i}Pr-$C\underline{H}_3$), 1.73 (br d, 12H, $^3J_{H-H}$ = 6.8 Hz, o-^{i}Pr-$C\underline{H}_3$), 2.91–3.06 (br sept, 2H, $^3J_{H-H}$ = 6.8 Hz, p-^{i}Pr-C$\underline{H}$), 3.56–3.78 (br, 4H, o-^{i}Pr-C$\underline{H}$), 7.31 (br, 7H, m-$C_6\underline{H}_3$+p-$C_6\underline{H}_3$+ m-$\underline{H}_{trip}$). **^{13}C{^{1}H}-NMR** [100.61 MHz, 299.2 K, benzene-d_6]: δ (ppm) = 20.4 (br, $P(\underline{C}H_3)_3$), 24.2 (o-^{i}Pr-$\underline{C}H_3$), 24.7 (p-^{i}Pr-$\underline{C}H_3$), 26.4 (o-^{i}Pr-$\underline{C}H_3$), 31.1 (o-^{i}Pr-$\underline{C}$H), 34.9 (p-^{i}Pr-$\underline{C}$H), 120.4 (m-$\underline{C}_{trip}$), 125.5 (m-/p-$\underline{C}_6H_3$), 130.4 (m-/p-$\underline{C}_6H_3$), 140.5 (i-$\underline{C}_{trip}$), 146.7 (p-$\underline{C}_{trip}$), 147.5 (o-$\underline{C}_{trip}$), 147.8 (o-$\underline{C}_6H_3$), 176.7 (i-$\underline{C}_6H_3$, weak intensity, assignment due to 2D-spectra). **^{31}P{^{1}H}-NMR** [161.97 MHz, 299.2 K, benzene-d_6]: δ (ppm) = –16.1 (br). **^{119}Sn{^{1}H}-NMR** [149.20 MHz, 299.2 K, benzene-d_6]: δ (ppm) = 192.6 (br). **Elemental analysis** calculated for $C_{48}H_{85}Cl_2P_4RhSn$ (%): C 53.45, H 7.94. Found C 53.61, H 8.26.

$[Ir(PMe_3)_4][Ar^*SnCl_2]$ (**10**): A solution of $[Ar^*SnCl]_2$ (39.4 mg, 31.0 µmol, 0.55 equiv.) in toluene (1.5 ml) is added to a suspension of $Ir(PMe_3)_4Cl$ (30.0 mg, 56.4 µmol, 1.00 equiv.) in toluene (0.3 ml) and stirred for one hour. The solvent is removed under reduced pressure, the remaining crude product is washed with n-pentane (3 x 1.5 ml) and dried under reduced pressure. $[Ir(PMe_3)_4][Ar^*SnCl_2]$ (54.1 mg, 46.3 µmol, 82%) is obtained as a red powder. $[Ir(PMe_3)_4][Ar^*SnCl_2]$ (**10**) can be crystallized from a highly saturated diethyl ether solution at room temperature to yield red crystals suitable for X-ray diffraction. **^{1}H-NMR** [600.13 MHz, 235.1 K, toluene-d_8]: δ (ppm) = 0.92–1.38 (br. m, 45H, 6 x p-^{i}Pr-$C\underline{H}_3$, 18 x o-^{i}Pr-$C\underline{H}_3$, 27 x $P(C\underline{H}_3)_3$), 1.38–1.54 (br. m, 21H, 6 x p-^{i}Pr-$C\underline{H}_3$, 6 x o-^{i}Pr-$C\underline{H}_3$, 9 x $P(C\underline{H}_3)_3$), 1.84 (br. d, 6H, $^3J_{H-H}$ = 4.6 Hz, o-^{i}Pr-$C\underline{H}_3$), 2.77–2.82 (br. m, 1H, p-^{i}Pr-C$\underline{H}$), 2.91–2.96 (br. m, 1H, o-^{i}Pr-C$\underline{H}$), 3.03–3.08 (br. m, 1H, p-^{i}Pr-C$\underline{H}$), 3.55–3.62 (br. m, 1H, o-^{i}Pr-C$\underline{H}$), 3.69–3.75 (br. m, 2H, o-^{i}Pr-C$\underline{H}$), 7.02 (br. s, 1H, m-$\underline{H}_{trip}$), 7.15 (br. s, 1H, m-$\underline{H}_{trip}$), 7.19–7.23 (br. m, 1H, p-$C_6\underline{H}_3$), 7.40 (br. s, 2H, m-$\underline{H}_{trip}$), 7.44 (br. s, 2H, m-$C_6\underline{H}_3$). **^{13}C{^{1}H}-NMR** [150.92 MHz, 235.0 K, toluene-d_8]: δ (ppm) = 21.6 (br. s, $P(\underline{C}H_3)$), 23.3 (s, o-^{i}Pr-$\underline{C}H_3$), 23.7 (s, $P(\underline{C}H_3)$), 24.0 (s, o-^{i}Pr-$\underline{C}H_3$), 24.3 (s, p-^{i}Pr-$\underline{C}H_3$), 24.6 (s, o-^{i}Pr-$\underline{C}H_3$), 24.8 (s, p-^{i}Pr-$\underline{C}H_3$), 25.1 (br. s, $P(\underline{C}H_3)$, 26.3 (s, o-^{i}Pr-$\underline{C}H_3$), 27.3 (s, o-^{i}Pr-$\underline{C}H_3$), 29.0 (br. s, $P(\underline{C}H_3)$, 30.5 (s, o-^{i}Pr-$\underline{C}$H), 30.7 (s, o-^{i}Pr-$\underline{C}$H), 30.9 (s, o-^{i}Pr-$\underline{C}$H), 34.6 (s, p-^{i}Pr-$\underline{C}$H), 34.9 (s, p-^{i}Pr-$\underline{C}$H), 120.0 (s, m-$\underline{C}_{trip}$), 120.8 (s, m-$\underline{C}_{trip}$), 122.1 (s, m-$\underline{C}_{trip}$), 125.5 (s, m-$\underline{C}_6H_3$), 129.7 (s, m-$\underline{C}_6H_3$), 132.2 (s, p-$\underline{C}_6H_3$), 140.1 (s, i-$\underline{C}_{trip}$), 142.4 (s, o-$\underline{C}_6H_3$), 146.4 (s, p-$\underline{C}_{trip}$), 147.4 (s, o-$\underline{C}_{trip}$), 147.5 (s, o-$\underline{C}_{trip}$), 148.6 (s, o-$\underline{C}_{trip}$), 149.8 (s, p-$\underline{C}_{trip}$), 175.3 (s, i-$\underline{C}_6H_3$–Sn). **^{31}P{^{1}H}-NMR** [242.92 MHz, 235.1 K, toluene-d_8]: δ (ppm) = −54.4 (br. t, 2P, $\underline{P}Me_3$), −50.4 (t + sat., 2P, $^2J_{119/117Sn-P}$ = 799.9 Hz, $^2J_{P-P}$ = 28.9 Hz, $\underline{P}Me_3$). **^{119}Sn{^{1}H}-NMR** [223.79 MHz, 235.0 K, toluene-d_8]: δ (ppm) = 190.8 (s). **Elemental analysis** calculated for $C_{48}H_{85}Cl_2IrP_4Sn$ (%): C 49.36, H 7.34. Found: C 49.53, H 7.01.

$Ar^*Ge(CO_3)Rh(CO)(PMe_3)_3$ (**11**): $Ar^*Ge{\equiv}Rh(PMe_3)_3$ (25.1 mg, 28.3 µmol, 1.00 equiv.) is dissolved in benzene (0.4 ml) and transferred into a *J. Young* NMR tube. Via a "freeze-pump-thaw" cycle, the argon atmosphere is removed and subsequently the NMR tube is flooded with carbon dioxide. After three days at room temperature all volatile components are removed under reduced pressure. The remaining solid is washed with *n*-pentane (3x1.0 ml) yielding the yellow powder of $Ar^*Ge(CO_3)Rh(CO)(PMe_3)_3$ (**11**) (21.7 mg, 22.3 µmol, 79%). Yellow crystals suitable for X-ray diffraction analysis are obtained from a concentrated *o*-difluorobenzene solution. **^{1}H-NMR** [700.21 MHz, 298.0 K, benzene-d_6]: δ (ppm) = 0.81 (d, 27H, $^3J_{H\text{-}H}$ = 6.6 Hz, P(C<u>H</u>$_3$)$_3$), 1.06–1.15 (br., 6H, *o*-^{i}Pr-C<u>H</u>$_3$), 1.28–1.34 (br., 6H, *o*-^{i}Pr-C<u>H</u>$_3$), 1.39–1.40 (d, 12H, $^3J_{H\text{-}H}$ = 6.8 Hz, *p*-^{i}Pr-C<u>H</u>$_3$), 1.54–1.62 (br., 6H, *o*-^{i}Pr-C<u>H</u>$_3$), 1.94–2.06 (br., 6H, *o*-^{i}Pr-C<u>H</u>$_3$), 2.97 (sept, 2H, $^3J_{H\text{-}H}$ = 6.8 Hz, *p*-^{i}Pr-C<u>H</u>), 3.31–3.50 (br. m, 4H, *o*-^{i}Pr-C<u>H</u>), 7.12–7.15 (m, 1H, *p*-C$_6$<u>H</u>$_3$), 7.18–7.22 (m, 4H, *m*-<u>H</u>$_{trip}$ + *m*-C$_6$<u>H</u>$_3$), 7.39 (br. 2H, *m*-<u>H</u>$_{trip}$). **^{13}C{^{1}H}-NMR** [176.07 MHz, 298.0 K, benzene-d_6]: δ (ppm) = 22.2 (*o*-^{i}Pr-<u>C</u>H$_3$), 23.3 (d, $^2J_{Rh\text{-}C}$ = 21.3 Hz, P(<u>C</u>H$_3$)$_3$), 24.6 (2 x *p*-^{i}Pr-<u>C</u>H$_3$), 25.0 (*o*-^{i}Pr-<u>C</u>H$_3$), 26.1 (*o*-^{i}Pr-<u>C</u>H$_3$), 27.2 (*o*-^{i}Pr-<u>C</u>H$_3$), 30.8 (*o*-^{i}Pr-<u>C</u>H), 32.4 (*o*-^{i}Pr-<u>C</u>H), 34.4 (*p*-^{i}Pr-<u>C</u>H), 120.2 (*m*-<u>C</u>$_{trip}$), 120.9 (*m*-<u>C</u>$_{trip}$), 127.3 (*p*-<u>C</u>$_6$H$_3$), 131.5 (*m*-<u>C</u>$_6$H$_3$), 138.1 (*i*-<u>C</u>$_{trip}$), 144.1 (*o*-<u>C</u>$_6$H$_3$), 146.6 (*o*-<u>C</u>$_{trip}$), 148.5 (*p*-<u>C</u>$_{trip}$), 149.2 (*o*-<u>C</u>$_{trip}$), 151.4 (*i*-<u>C</u>$_6$H$_3$), 157.1 (Ge-<u>C</u>O$_3$), 201.4 (br., Rh-<u>C</u>O). **^{31}P{^{1}H}-NMR** [161.97 MHz, 299.2 K, benzene-d_6]: δ (ppm) = –24.7 (d, $^1J_{Rh\text{-}P}$ = 111 Hz). **Elemental analysis** calculated for $C_{47}H_{76}GeO_4P_3Rh$ + 3 $C_6H_4F_2$ (%): C 59.33, H 6.74. Found: C 58.98, H 6.95. **IR** (cm^{-1}): 1714, 1932 (ν Rh–CO and Ge–CO$_3$).

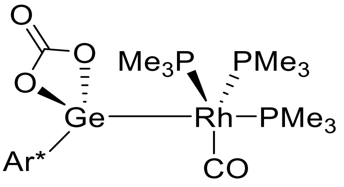

$Ar^*Ge(CO_3)Ir(CO)(PMe_3)_3$ (**12**): $Ar^*Ge{\equiv}Ir(PMe_3)_3$ (27.9 mg, 28.6 µmol, 1.00 equiv.) is dissolved in benzene (0.40 ml) and transferred into a *J. Young* NMR tube. The dark purple solution is degassed with one "freeze-pump-thaw" cycle after which the atmosphere is exchanged with carbon dioxide. After one hour at room temperature the yellow solution is transferred to a vial and the solvent is removed under reduced pressure. The crude product is dissolved in *o*-difluorobenzene and the concentrated solution is stored at –40 °C to yield $Ar^*Ge(CO_3)Ir(CO)(PMe_3)_3$ (**12**) as yellow crystals (26.1 mg, 22.2 µmol, 78%) suitable for X-ray diffraction. **^{1}H-NMR** [700.21 MHz, 298.0 K, benzene-d_6]: δ (ppm) = 0.94–0.99 (m, 27H, P(C<u>H</u>$_3$)$_3$), 1.09 (d, 6H, $^3J_{H\text{-}H}$ = 6.8 Hz, *o*-^{i}Pr-C<u>H</u>$_3$), 1.30 (d, 6H, $^3J_{H\text{-}H}$ = 6.7 Hz, *o*-^{i}Pr-C<u>H</u>$_3$), 1.36–1.40 (m, 12H, *p*-^{i}Pr-C<u>H</u>$_3$), 1.60 (d, 6H, $^3J_{H\text{-}H}$ = 6.7 Hz, *o*-^{i}Pr-C<u>H</u>$_3$), 2.00 (d, 6H, $^3J_{H\text{-}H}$ = 6.7 Hz, *o*-^{i}Pr-C<u>H</u>$_3$), 2.96 (sept, 2H, $^3J_{H\text{-}H}$ = 6.9 Hz, *p*-^{i}Pr-C<u>H</u>), 3.34 (sept, 2H, $^3J_{H\text{-}H}$ = 6.7 Hz, *o*-^{i}Pr-C<u>H</u>), 3.46 (sept, 2H, $^3J_{H\text{-}H}$ = 6.7 Hz, *o*-^{i}Pr-C<u>H</u>), 7.12–7.14 (m, 1H, *p*-C$_6$<u>H</u>$_3$), 7.19–7.23 (m, 4H, *m*-<u>H</u>$_{trip}$ + *m*-C$_6$<u>H</u>$_3$), 7.35–7.40 (m, 2H, *m*-<u>H</u>$_{trip}$). **^{13}C{^{1}H}-NMR** [176.08 MHz, 298.0 K, benzene-d_6]: δ (ppm) = 21.9 (s, *o*-^{i}Pr-<u>C</u>H$_3$), 24.0–24.2 (m, P(<u>C</u>H$_3$)$_3$ + *p*-^{i}Pr-<u>C</u>H$_3$), 24.4 (s, *o*-^{i}Pr-<u>C</u>H$_3$), 24.6 (s, *p*-^{i}Pr-<u>C</u>H$_3$), 25.8 (s, *o*-^{i}Pr-<u>C</u>H$_3$), 27.0 (s, *o*-^{i}Pr-<u>C</u>H$_3$), 30.4 (s, *o*-^{i}Pr-<u>C</u>H), 32.1 (s, *o*-^{i}Pr-<u>C</u>H), 34.7 (s, *p*-^{i}Pr-<u>C</u>H), 119.9 (s, *m*-<u>C</u>$_{trip}$), 120.5 (s, *m*-<u>C</u>$_{trip}$), 126.9 (s, *p*-<u>C</u>$_6$H$_3$), 131.2 (s, *m*-<u>C</u>$_6$H$_3$), 137.6 (s, *i*-<u>C</u>$_{trip}$), 144.1 (s, *o*-<u>C</u>$_6$H$_3$), 146.5 (s, *o*-<u>C</u>$_{trip}$), 147.3 (q, $^3J_{P\text{-}C}$ = 5.2 Hz, *i*-<u>C</u>$_6$H$_3$–Ge), 148.0 (s, *p*-<u>C</u>$_{trip}$), 150.9 (s, *o*-<u>C</u>$_{trip}$), 156.8 (s, <u>C</u>O$_3$), 187.5 (q, $^2J_{P\text{-}C}$ = 25.6 Hz, <u>C</u>O). **^{31}P{^{1}H}-NMR** [161.97 MHz, 299.2 K, benzene-d_6]: δ (ppm) = –60.4 (s, <u>P</u>Me$_3$). **Elemental analysis** calculated for $C_{47}H_{76}GeIrO_4P_3$ + $C_6H_4F_2$ (%): C 54.09, H 6.85. Found: C 54.46, H 6.43. **IR** (cm^{-1}): 1718, 1919 (*ν*(CO) and *ν*(CO$_3$)).

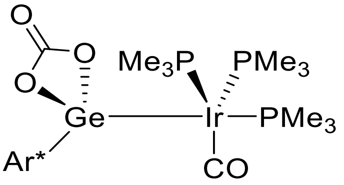

[$Ar^*Sn(\mu\text{-}CO_2)Rh(PMe_3)_3$] (**13**): $Ar^*Sn{\equiv}Rh(PMe_3)_3$ (24.8 mg, 26.6 µmol, 1.00 equiv.) is dissolved in benzene (0.40 ml) and transferred into a *J. Young* NMR tube. The argon atmosphere is removed via a "freeze-pump-thaw" cycle and subsequently the NMR tube is flooded with carbon dioxide. After one hour at room

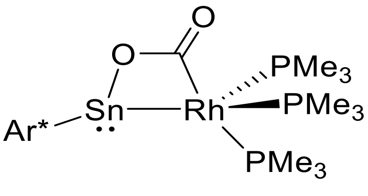

temperature all volatile components are removed under reduced pressure yielding the crude product. Red crystals of the stanna rhoda lactone **13** (12.7 mg, 13.0 µmol, 49%) suitable for X-ray diffraction are obtained from a concentrated *o*-difluorobenzene solution at –40 °C. **^{1}H-NMR** [600.13 MHz, 298.0 K, benzene-d_6]: δ (ppm) = 0.82 (d, 9H, $^3J_{H\text{-}H}$ = 6.0 Hz, $P(CH_3)_3$), 1.18 (d, 12H, $^3J_{H\text{-}H}$ = 6.9 Hz, *o*-^{i}Pr-CH_3), 1.22–1.26 (m, 18H, $P(CH_3)_3$), 1.31 (d, 12H, $^3J_{H\text{-}H}$ = 6.9 Hz, *p*-^{i}Pr-CH_3), 1.57 (d, 12H, $^3J_{H\text{-}H}$ = 6.9 Hz, *o*-^{i}Pr-CH_3), 2.88 (sept, 2H, $^3J_{H\text{-}H}$ = 6.9 Hz, *p*-^{i}Pr-CH), 3.29 (sept, 4H, $^3J_{H\text{-}H}$ = 6.9 Hz, *o*-^{i}Pr-CH), 7.19–7.30 (m, 7H, *m*-C_6H_3 + *p*-C_6H_3 + *m*-H_{trip}). **^{13}C{^{1}H}-NMR** [150.90 MHz, 298.0 K, benzene-d_6]: δ (ppm) = 23.9 (*o*-^{i}Pr-CH_3), 24.2–25.0 ($P(CH_3)_3$ + *p*-^{i}Pr-CH_3), 26.1 (*o*-^{i}Pr-CH_3), 31.1 (*o*-^{i}Pr-CH), 34.7 (*p*-^{i}Pr-CH), 121.3 (*m*-C_{trip}), 127.5 (*m*-/*p*-C_6H_3), 129.7 (*m*-/*p*-C_6H_3), 136.8 (*i*-C_{trip}), 144.9 (*o*-C_6H_3), 148.2 (*o*-C_{trip}), 148.7 (*p*-C_{trip}), 174.4 (*i*-C_6H_3), 193.9 (br, Rh-CO_2). **^{31}P{^{1}H}-NMR** [161.97 MHz, 300.2 K, benzene-d_6]: δ (ppm) = –16.9 (dt, $^1J_{Rh\text{-}P}$ = 100 Hz, $^2J_{P\text{-}P}$ = 36.3 Hz, *trans*-$P(CH_3)_3$), –9.3 (dd, $^1J_{Rh\text{-}P}$ = 187 Hz, $^2J_{P\text{-}P}$ = 36.6 Hz, *cis*-$P(CH_3)_3$). **^{119}Sn{^{1}H}-NMR** [223.79 MHz, 298.0 K, C_6D_6]: δ (ppm) = 736.8 (tdd, $^2J_{P\text{-}Ptrans}$ = 550 Hz, $^2J_{P\text{-}Pcis}$ = 330 Hz, $^1J_{Sn\text{-}Rh}$ = 80 Hz). **Elemental analysis** calculated for $C_{46}H_{76}O_2P_3RhSn$ (%): C 56.68, H 7.85. Found: C 57.86, H 7.01. **IR** (cm^{-1}): 1665, 1926(ν Rh–CO and Rh=CO).

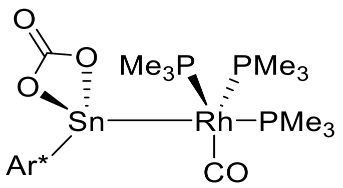

$Ar^*Sn(CO_3)Rh(CO)(PMe_3)_3$ (**14**): $Ar^*Sn{\equiv}Rh(PMe_3)_3$ (28.6 mg, 30.7 µmol, 1.00 equiv.) is dissolved in benzene (0.40 ml) and transferred into a *J. Young* NMR tube. The argon atmosphere is removed via a "freeze-pump-thaw" cycle and subsequently the NMR tube is flooded with carbon dioxide. After three days at room temperature all volatile components are removed under reduced pressure yielding the yellow crude product. Yellow crystals of $Ar^*Sn(CO_3)Rh(CO)(PMe_3)_3$ (**14**) (21.7 mg, 21.3 µmol, 69%) suitable for X-ray diffraction are obtained from a concentrated *o*-difluorobenzene solution at –40 °C. **^{1}H-NMR** [700.21 MHz, 299.2 K, benzene-d_6 + *o*-difluorobenzene]: δ (ppm) = 1.01 (br, 27H, $P(CH_3)_3$), 1.06 (d, 12H, $^3J_{H\text{-}H}$ = 6.9 Hz, *o*-^{i}Pr-CH_3), 1.29 (d, 12H, $^3J_{H\text{-}H}$ = 6.9 Hz, *p*-^{i}Pr-CH_3), 1.64 (d, 12H, $^3J_{H\text{-}H}$ = 6.9 Hz, *o*-^{i}Pr-CH_3), 2.87 (sept, 2H, $^3J_{H\text{-}H}$ = 6.9 Hz, *p*-^{i}Pr-CH), 3.32 (sept, 4H, $^3J_{H\text{-}H}$ = 6.9 Hz, *o*-^{i}Pr-CH), 7.17–7.30 (m, 7H, *m*-C_6H_3 + *p*-C_6H_3 + *m*-H_{trip}). **^{13}C{^{1}H}-NMR** [176.07 MHz, 298.0 K, benzene-d_6 + *o*-difluorobenzene]: δ (ppm) = 22.8–23.4 (br, $P(CH_3)_3$), 23.7 (*o*-^{i}Pr-CH_3), 24.4 (*p*-^{i}Pr-CH_3), 26.6 (*o*-^{i}Pr-CH_3), 31.5 (*o*-^{i}Pr-CH), 34.8 (*p*-^{i}Pr-CH), 121.2 (*m*-C_{trip}), 126.9 (*m*-/*p*-C_6H_3), 130.9 (*m*-/*p*-C_6H_3), 138.9 (*i*-C_{trip}), 146.0 (*o*-C_6H_3), 148.6 (*p*-C_{trip}), 150.7 (*o*-C_{trip}), 151.1 (*o*-C_{trip}), 161.5 (Sn–CO_3), 164.5 (*i*-C_6H_3), 200.2–201.4 (m, Rh–CO). **^{31}P{^{1}H}-NMR** [161.97 MHz, 299.2 K, benzene-d_6 + *o*-difluorobenzene]: δ (ppm) = –19.5 (d + satellites, $^1J_{Rh\text{-}P}$ = 108 Hz, $^2J_{Sn\text{-}P}$ = 278 Hz). **^{119}Sn{^{1}H}-NMR** [149.20 MHz, 299.2 K, benzene-d_6 + *o*-difluorobenzene]: δ (ppm) = 139.7–147.1 (m). **Elemental analysis** calculated for $C_{45}H_{76}O_4P_3RhSn$ + 3 $C_6H_4F_2$ (%): C 57.32, H 6.51. Found: C 57.86, H 7.014. **IR** (cm^{-1}): 1665, 1926 (ν Rh–CO and Sn–CO_3).

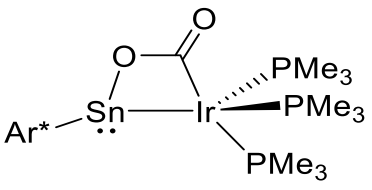

$[Ar^*Sn(\mu\text{-}CO_2)Ir(PMe_3)_3]$ (**15**): $Ar^*Sn{\equiv}Ir(PMe_3)_3$ (39.7 mg, 38.9 µmol, 1.00 equiv.) is dissolved in benzene (1.80 ml) and transferred into a *J. Young* NMR tube. The dark brown solution is degassed with one "freeze-pump-thaw" cycle after which the atmosphere is exchanged with carbon dioxide. After one hour at room temperature the dark red solution is transferred to a vial and the solvent is removed under reduced pressure. The red crude product is dissolved in *o*-difluorobenzene and the concentrated solution is stored at –40 °C to yield the stanna irida lactone **15** as red crystals (15.4 mg, 13.3 µmol, 34%) suitable for X-ray diffraction. **^{1}H-NMR** [400.11 MHz, 299.2 K, benzene-d_6]: δ (ppm) = 1.02 (d, 9H, $^2J_{P\text{-}H}$ = 7.4 Hz, *trans*-$P(CH_3)_3$), 1.16 (d, 12H, $^3J_{H\text{-}H}$ = 6.8 Hz, *o*-^{i}Pr-CH_3), 1.31 (d, 12H, $^3J_{H\text{-}H}$ = 6.9 Hz, *p*-^{i}Pr-CH_3), 1.38–1.45 (m, 18H, *cis*-$P(CH_3)_3$), 1.56 (d, 12H, $^3J_{H\text{-}H}$ = 6.9 Hz, *o*-^{i}Pr-CH_3), 2.88 (sept, 2H, $^3J_{H\text{-}H}$ = 6.9 Hz, *p*-^{i}Pr-CH), 3.24 (sept, 4H, $^3J_{H\text{-}H}$ = 6.8 Hz, *o*-^{i}Pr-CH), 7.19–7.25 (m, 7H, *m*-/*p*-C_6H_3 + *m*-H_{trip}). **^{13}C{^{1}H}-NMR**

[176.08 MHz, 298.0 K, benzene-d_6]: δ (ppm) = 22.8 (s, o-^{i}Pr-C̲H$_3$), 23.4 (s, p-^{i}Pr-C̲H$_3$), 24.9 (s, o-^{i}Pr-C̲H$_3$), 25.2–25.3 (m, N = $|^1J_{P\text{-}C} + {}^3J_{P\text{-}C}|$ = 29.9 Hz, $^3J'_{P\text{-}C}$ = 3.3 Hz, *cis*-P(C̲H$_3$)$_3$), 27.5 (dt, $^1J_{P\text{-}C}$ = 26.5 Hz, $^3J_{P\text{-}C}$ = 4.1 Hz, *trans*-P(C̲H$_3$)$_3$), 29.9 (s, o-^{i}Pr-C̲H), 33.6 (s, p-^{i}Pr-C̲H), 120.0 (s, m-C̲$_{trip}$), 126.6 (s, m-/p-C̲$_6$H$_3$), 128.3 (s, m-/p-C̲$_6$H$_3$), 135.8 (s, i-C̲$_{trip}$), 143.8 (s, o-C̲$_6$H$_3$), 147.0 (s, o-C̲$_{trip}$), 147.6 (s, p-C̲$_{trip}$), 165.4 (td, $^3J_{P(cis)\text{-}C}$ = 12.9 Hz, $^3J_{P(trans)\text{-}C}$ = 3.7 Hz, i-C̲$_6$H$_3$–Sn), 173.4 (dt, $^3J_{P(cis)\text{-}C}$ = 98.5 Hz, $^3J_{P(trans)\text{-}C}$ = 10.3 Hz, Ir–C̲O$_2$). **^{31}P{^{1}H}-NMR** [161.97 MHz, 299.2 K, benzene-d_6]: δ (ppm) = –51.6 (t, $^2J_{P\text{-}P}$ = 18.1 Hz, *trans*-P̲(CH$_3$)$_3$), –36.9 (d + sat., $^2J_{119/117Sn\text{-}P}$ = 695.3 Hz, $^2J_{P\text{-}P}$ = 18.1 Hz, *cis*-P̲(CH$_3$)$_3$). **^{119}Sn{^{1}H}-NMR** [111.94 MHz, 298.0 K, benzene-d_6]: δ (ppm) = 424.8 (dt, $^2J_{Sn\text{-}P(cis)}$ = 706.8 Hz, $^2J_{Sn\text{-}P(trans)}$ = 90.7 Hz). **Elemental analysis** calculated for $C_{46}H_{76}IrO_2P_3Sn$ (%): C 51.88, H 7.19. Found: C 52.05, H 7.12. **IR** (cm^{-1}): 1585 (ν(C=O)).

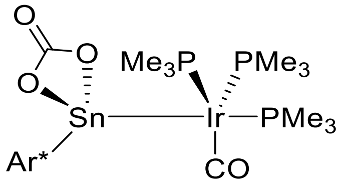

Ar*Sn(CO$_3$)Ir(CO)(PMe$_3$)$_3$ (**16**): Ar*Sn≡Ir(PMe$_3$)$_3$ (34.1 mg, 33.4 µmol, 1.00 equiv.) is dissolved in benzene (0.40 ml) and transferred into a *J. Young* NMR tube. The dark brown solution is degassed with one "freeze-pump-thaw" cycle after which the atmosphere is exchanged with carbon dioxide. After one hour at room temperature the pale-yellow solution is transferred to a vial and the solvent is removed under reduced pressure. The yellow crude product is dissolved in o-difluorobenzene and the concentrated solution is stored at –40 °C to yield Ar*Sn(CO$_3$)Ir(CO)(PMe$_3$)$_3$ (**16**) as yellow crystals (33.2 mg, 22.9 µmol, 69%) suitable for X-ray diffraction. **^{1}H-NMR** [400.11 MHz, 299.2 K, benzene-d_6]: δ (ppm) = 1.07–1.14 (m, 27H, P(CH̲$_3$)$_3$), 1.15 (d, 12H, $^3J_{H\text{-}H}$ = 6.7 Hz, o-^{i}Pr-CH̲$_3$), 1.32 (d, 12H, $^3J_{H\text{-}H}$ = 6.9 Hz, p-^{i}Pr-CH̲$_3$), 1.75 (br. m, 12H, o-^{i}Pr-CH̲$_3$), 2.89 (sept, 2H, $^3J_{H\text{-}H}$ = 6.8 Hz, p-^{i}Pr-CH̲), 3.30–3.50 (br. m, 4H, o-^{i}Pr-CH̲), 7.16–7.27 (m, 7H, m-/p-C$_6$H̲$_3$ + m-H̲$_{trip}$). **^{13}C{^{1}H}-NMR** [176.08 MHz, 298.0 K, benzene-d_6]: δ (ppm) = 23.2 (br. s, o-^{i}Pr-C̲H$_3$), 24.1–24.4 (m, p-^{i}Pr-C̲H$_3$ + P(C̲H$_3$)$_3$), 26.5 (s, o-^{i}Pr-C̲H$_3$), 31.1 (br. s, o-^{i}Pr-C̲H), 34.5 (s, p-^{i}Pr-C̲H), 120.7 (br. s, m-C̲$_{trip}$), 126.8 (s, m-/p-C̲$_6$H$_3$), 130.7 (s, m-/p-C̲$_6$H$_3$), 138.4 (s, i-C̲$_{trip}$), 145.7 (s, o-C̲$_6$H$_3$), 148.2 (s, p-C̲$_{trip}$), 148.6 (br. s, o-C̲$_{trip}$), 158.4 (q, $^3J_{P\text{-}C}$ = 4.3 Hz, i-C̲$_6$H$_3$–Sn), 160.4 (s, C̲O$_3$), 189.4 (q, $^2J_{P\text{-}C}$ = 19.7 Hz, C̲O). **^{31}P{^{1}H}-NMR** [161.97 MHz, 299.2 K, benzene-d_6]: δ (ppm) = –57.0 (s + sat. $^2J_{119/117Sn\text{-}P}$ = 200.2 Hz, P̲(CH$_3$)$_3$,). **^{119}Sn{^{1}H}-NMR** [149.20 MHz, 299.2 K, benzene-d_6]: δ (ppm) = –37.2 (q, $^2J_{Sn\text{-}P}$ = 220.2 Hz). **Elemental analysis** calculated $C_{47}H_{76}IrO_4P_3Sn + C_6H_4F_2$ (%): C 52.05, H 6.59. Found: C 52.48, H 6.82. **IR** (cm^{-1}): 1671, 1913 (ν(CO) and ν(CO$_3$)).

Data of crystal structure determinations

Table SI 1. Crystal structure refinement tables of compounds **1–8**.

	**1**	**2**	**3**	**4**	**5**	**6**	**7**	**8**
Empirical formula	$C_{45}H_{76}CoGeP_3$	2 $C_{45}H_{76}GeP_3Rh$ · C_7H_8	$C_{97}H_{160}Ge_2Ir_2P_6$	2 $C_{45}H_{76}P_3RhSn$ · C_7H_8	$C_{48.5}H_{80}IrP_3Sn$	2 $C_{45}H_{76}P_3PbRh$ · C_7H_8	$C_{54}H_{94}IrP_3Pb$	$C_{48}H_{85}Cl_2GeP_4Rh$ · 2 $C_4H_{10}O$
M_r / g mol^{-1}	841.48	1863.06	2041.64	1955.26	1066.92	2132.26	1235.59	1180.67
λ / Å	0.71073	0.71073	0.71073	0.71073	0.71073	0.71073	0.71073	0.71073
T / K	120(2)	120(2)	120(2)	120(2)	120(2)	100(2)	120(2)	120(2)
Crystal system	monoclinic	orthorhombic	orthorhombic	orthorhombic	orthorhombic	orthorhombic	triclinic	monoclinic
Space group	$P2_1/c$	$P2_12_12_1$	$P2_1\,2_1\,2_1$	$P2_12_12_1$	$P2_1\,2_1\,2_1$	$P2_1\,2_1\,2_1$	$P\bar{1}$	$P2_1$/n
Z	4	4	4	4	8	4	2	4
a / Å	15.9410(8)	19.9643(4)	19.9651(5)	20.3614(5)	20.3846(7)	20.4743(8)	11.1475(2)	21.5897(4)
b / Å	14.5984(7)	21.3584(4)	21.3495(6)	21.3649(5)	21.3450(8)	21.3395(9)	12.7527(2)	13.3991(3)
c / Å	21.2234(10)	24.1295(5)	24.1047(6)	23.9744(5)	23.9453(9)	23.8909(10)	20.8634(4)	24.1489(5)
α / °	90	90	90	90	90	90	103.7470(10)	90
β / °	103.734(2)	90	90	90	90	90	96.4630(10)	113.2960(10)
γ / °	90	90	90	90	90	90	102.1950(10)	90
V / Å^3	4797.8(4)	10289.0(4)	10274.5(5)	10429.3(4)	10418.8(7)	10438.2(7)	2774.30(9)	6416.3(2)
D_c / g cm^{-3}	1.165	1.203	1.320	1.245	1.360	1.357	1.479	1.222
μ / mm^{-1}	1.099	1.027	3.295	0.916	3.152	3.658	5.544	0.944
F(000)	1800	3944	4200	4088	4344	4344	1244	2512
Crystal size / mm	0.30 x 0.27 x 0.26	0.27 x 0.25 x 0.24	0.31 x 0.28 x 0.27	0.31 x 0.28 x 0.26	0.29 x 0.27 x 0.25	0.29 x 0.28 x 0.26	0.27 x 0.25 x 0.24	0.33 x 0.31 x 0.28
θ range / °	1.445 - 30.509	3.206 – 28.768	1.396 - 31.535	2.984 – 33.379	1.381 - 27.913	1.378 – 28.814	1.696 – 34.342	1.626 – 29.503
Limiting indices	–22 ≤ h ≤ 22<br>–20 ≤ k ≤ 20<br>–30 ≤ l ≤ 30	–27 ≤ h ≤ 26<br>–28 ≤ k ≤ 28<br>–32 ≤ l ≤ 32	–29 ≤ h ≤ 29<br>–31 ≤ k ≤ 31<br>–35 ≤ l ≤ 35	–31 ≤ h ≤ 31<br>–32 ≤ k ≤ 32<br>–37 ≤ l ≤ 36	–26 ≤ h ≤ 26<br>–28 ≤ k ≤ 28<br>–31 ≤ l ≤ 31	–27 ≤ h ≤ 27<br>–28 ≤ k ≤ 28<br>–32 ≤ l ≤ 32	–17 ≤ h ≤ 17<br>–20 ≤ k ≤ 20<br>–33 ≤ l ≤ 29	–29 ≤ h ≤ 29<br>–18 ≤ k ≤ 18<br>–33 ≤ l ≤ 32
Reflections collect.	169877	288310	448343	471644	318194	390346	133566	183411
Indepdnt. Reflections	14626	26672	34226	40094	24823	27176	22058	17870
R_{int}	0.0261	0.0487	0.0477	0.0470	0.0564	0.0443	0.0220	0.0402
Completeness	99.8%	99.5%	99.8%	99.6%	99.5%	99.7%	94.8%	99.9%
Absorp. Corr.	multi-scan	multi-scan	multi-scan	multi-scan	multi-scan	multi-scan	multi-scan	multi-scan
Trans. (max., min.)	0.7461, 0.6765	0.7458, 0.7081	0.7462, 0.6393	0.7466, 0.6990	0.7456, 0.6450	0.7458, 0.5000	0.7468, 0.4788	0.7459, 0.6907
Parameters/restraints	472 / 0	1072 / 357	1072 / 357	1007 / 0	1060 / 363	1007 / 0	629 / 0	671 / 151
R_1, ωR_2 [$I > 2\sigma(I)$]	0.0278, 0.0666	0.0284, 0.0530	0.0201, 0.0382	0.0345, 0.0601	0.0237, 0.0411	0.0213, 0.0439	0.0274, 0.0542	0.0327, 0.0729
R_1, ωR_2 (all data)	0.0391, 0.0713	0.0379, 0.0559	0.0264, 0.0399	0.0535, 0.0655	0.0333, 0.0434	0.0287, 0.0467	0.0372, 0.0562	0.0507, 0.0811
GooF on F^2	1.029	1.025	1.009	1.011	1.009	0.997	1.152	1.033
$\Delta\rho_{max,min}$ / e·Å^{-3}	0.482, –0.344	0.785, –0.396	1.022, –0.567	1.288, –0.753	0.999, –0.521	1.521, –0.701	2.747, –1.331	1.036, –0.877
CCDC	2421880	2421873	2421883	2421876	2421882	2421875	2421879	2421872

Table SI 2. Crystal structure refinement tables of compounds **9–16**.

	**9**	**10**	**11**	**12**	**13**	**14**	**15**	**16**
Empirical formula	$C_{48}H_{85}Cl_2P_4RhSn$<br>· $C_4H_{10}O$	$C_{52}H_{95}Cl_2IrOP_4Sn$	$C_{47}H_{76}GeO_4P_3Rh$<br>· 3 $C_6H_4F_2$	$C_{53}H_{80}F_2GeIrO_4P_3$	$C_{46}H_{76}O_2P_3RhSn$<br>· $C_6H_4F_2$	$C_{47}H_{76}O_4P_3RhSn$<br>· 3 $C_6H_4F_2$	$C_{52}H_{80}F_2IrO_2P_3Sn$	$C_{65}H_{88}F_6IrO_4P_3Sn$
M_r / g mol^{-1}	1152.65	1241.94	1315.76	1176.87	1089.67	1361.86	1178.96	1451.15
λ / Å	0.71073	0.71073	0.71073	0.71073	0.71073	0.71073	0.71073	0.71073
T / K	120(2)	120(2)	120(2)	120(2)	120(2)	120(2)	120(0)	120(0)
Crystal system	triclinic	triclinic	triclinic	monoclinic	monoclinic	triclinic	monoclinic	triclinic
Space group	$P\bar{1}$	$P\bar{1}$	$P\bar{1}$	$P2_1/c$	$P2_1$/n	$P\bar{1}$	$P2_1/c$	$P\bar{1}$
Z	4	4	2	4	4	2	4	2
a / Å	16.4705(3)	16.4664(4)	12.8001(2)	12.9228(3)	10.5118(4)	12.7949(3)	10.5138(3)	12.7836(2)
b / Å	20.8910(4)	20.9015(5)	14.5454(2)	24.0859(5)	23.1271(8)	14.6234(4)	23.0333(6)	14.6143(2)
c / Å	20.9216(3)	20.9081(5)	18.5995(3)	18.5327(4)	22.0872(7)	18.7987(5)	22.0824(5)	18.7711(3)
α / °	60.4500(10)	60.3800(10)	86.7332(8)	90	90	86.5350(10)	90	86.6660(10)
β / °	74.6970(10)	74.7730(10)	75.1649(7)	110.3040(10)	92.260(2)	74.9260(10)	92.5030(10)	74.9060(10)
γ / °	78.5750(10)	78.6280(10)	74.9348(9)	90	90	74.3760(10)	90	74.7140(10)
V / Å^3	6020.57(19)	6016.5(3)	3232.25(9)	5410.0(2)	5365.4(3)	3270.63(15)	5342.5(2)	3265.90(9)
D_c / g cm^{-3}	1.272	1.371	1.352	1.445	1.348	1.383	1.466	1.476
μ / mm^{-1}	0.916	2.852	0.856	3.150	0.905	0.769	3.089	2.553
F(000)	2416	2544	1372	2408	2260	1408	2392	1472
Crystal size / mm	0.34 x 0.32 x 0.27	0.26 x 0.24 x 0.22	0.40 x 0.36 x 0.27	0.33 x 0.29 x 0.27	0.29 x 0.26 x 0.25	0.33 x 0.29 x 0.27	0.41 x 0.17 x 0.16	0.33 x 0.29 x 0.27
θ range / °	1.286 – 30.551	1.286 - 29.186	1.450 – 32.093	1.445 - 29.142	1.761 – 30.550	1446 – 30.025	1.278 - 27.399	1.445 - 28.367
Limiting indices	$-23 \le h \le 23$<br>$-29 \le k \le 29$<br>$-29 \le l \le 29$	$-22 \le h \le 22$<br>$-28 \le k \le 28$<br>$-28 \le l \le 28$	$-19 \le h \le 18$<br>$-21 \le k \le 21$<br>$-27 \le l \le 27$	$-17 \le h \le 17$<br>$-32 \le k \le 32$<br>$-25 \le l \le 25$	$-15 \le h \le 14$<br>$-32 \le k \le 29$<br>$-31 \le l \le 31$	$-17 \le h \le 17$<br>$-20 \le k \le 20$<br>$-26 \le l \le 26$	$-13 \le h \le 13$<br>$-29 \le k \le 29$<br>$-28 \le l \le 28$	$-14 \le h \le 17$<br>$-19 \le k \le 19$<br>$-24 \le l \le 25$
Reflections collect.	307170	227394	84789	259912	135348	119564	118583	40951
Indepdnt. Reflections	36769	32398	22555	14540	16307	18997	12059	15945
R_{int}	0.0475	0.0506	0.0237	0.0322	0.0316	0.0204	0.0645	0.0203
Completeness	99.7%	99.5%	99.6%	100%	99.3%	99.3%	99.2%	97.6%
Absorp. Corr.	multi-scan	multi-scan	multi-scan	multi-scan	multi-scan	multi-scan	multi-scan	multi-scan
Trans. (max., min.)	0.7461, 0.6986	0.7458, 0.6409	0.8315, 0.7600	0.7458, 0.5945	0.7461, 0.6951	0.7460, 0.6980	0.7455, 0.6684	0.7457, 0.6242
Parameters/restraints	1224 / 371	1185 / 69	742 / 0	689 / 497	582 / 0	760 / 69	581 / 0	766 / 69
R_1, ωR_2 [$I > 2\sigma(I)$]	0.0360, 0.0757	0.0406, 0.0708	0.0256, 0.0635	0.0391, 0.0829	0.0354, 0.0833	0.0275, 0.0690	0.0306, 0.0525	0.0214, 0.0495
R_1, ωR_2 (all data)	0.0607, 0.0870	0.0768, 0.0806	0.0324, 0.0671	0.0412, 0.0836	0.0489, 0.0882	0.0327, 0.0745	0.0452, 0.0557	0.0252, 0.0513
GooF on F^2	1.025	1.000	0.993	1.392	1.068	0.977	1.018	1.044
$\Delta\rho_{max,min}$ / e·Å^{-3}	1.465, –1.118	4.099, –2.084	0.965, –0.604	1.209, –2.418	4.473, –0.724	1.125, –0.792	2.132, –1.173	1.191, –0.700
CCDC	2421874	2421884	2421869	2421881	2421871	2421870	2421878	2421877

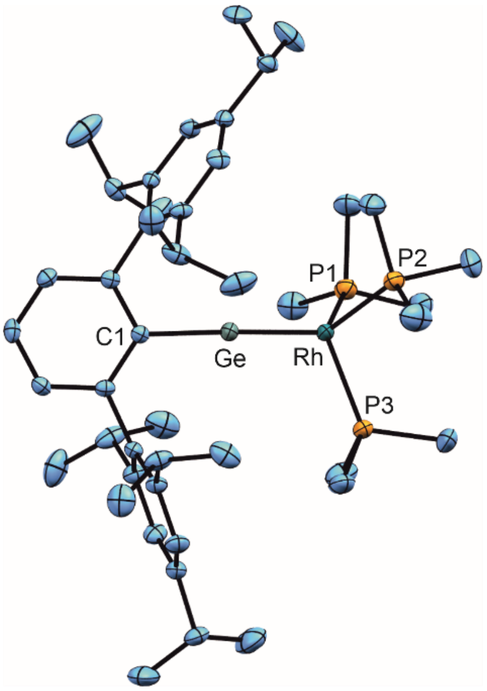

Figure SI01. ORTEP of the molecular structure of **2**. Ellipsoids set at 50% probability. Hydrogen atoms are omitted for clarity. Interatomic distances (Å) and angles (°): Rh-Ge 2.1953(3); C1-Ge 1.999(3), Rh-P1 2.2584(8), Rh-P2 2.2665(8), Rh-P3 2.2680(9), C1-Ge-Rh 178.0(1).

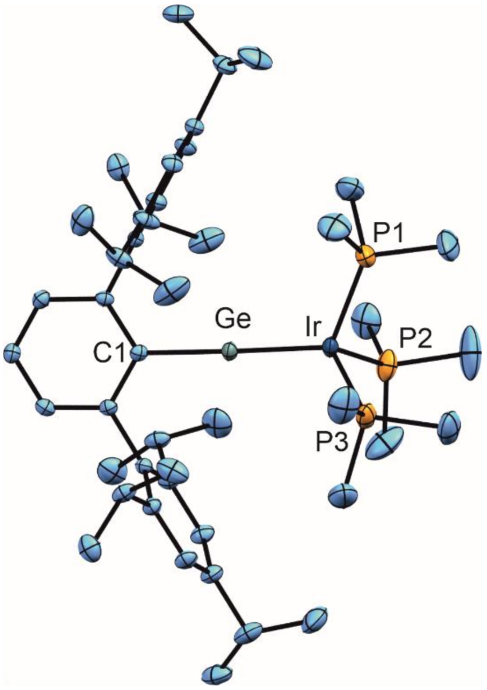

Figure SI02. ORTEP of the molecular structure of **3**. Ellipsoids set at 50% probability. Hydrogen atoms are omitted for clarity. Interatomic distances (Å) and angles (°): Ir-Ge 2.2087(3), C1-Ge 1.985(2), Ir-P1 2.2440(7), Ir-P2 2.2489(7), Ir-P3 2.2524(8), C1-Ge-Ir 178.1(1).

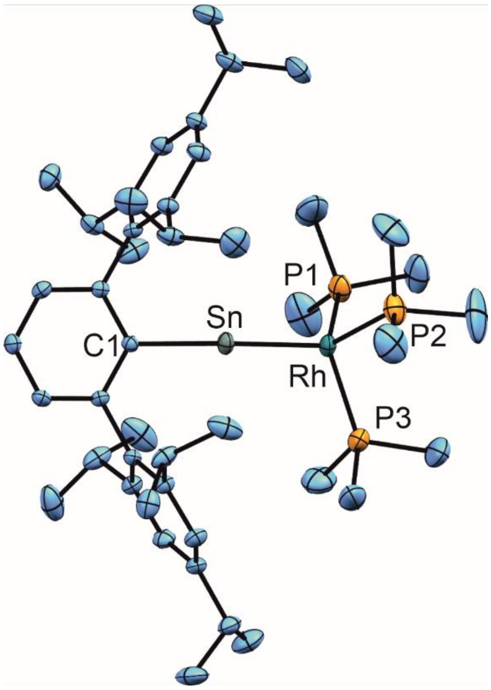

Figure SI03. ORTEP of the molecular structure of **4**. Ellipsoids set at 50% probability. Hydrogen atoms are omitted for clarity Interatomic distances (Å) and angles (°): Rh-Sn 2.3748(3), C1-Sn 2.197(3), Rh-P1 2.2500(9), Rh-P2 2.2586(9), Rh-P3 2.2588(9), C1-Sn-Rh 177.4(1).

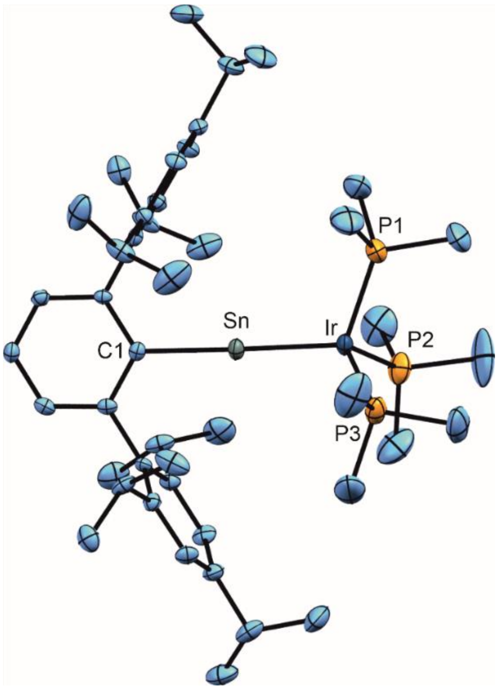

Figure SI04. ORTEP of the molecular structure of **5**. Ellipsoids set at 50% probability. Hydrogen atoms are omitted for clarity. Interatomic distances (Å) and angles (°): Ir-Sn 2.3909(3), C1-Sn 2.178(4), Ir-P1 2.236(1), Ir-P2 2.242(1), Ir-P3 2.244(1), C1-Sn-Ir 177.8(1).

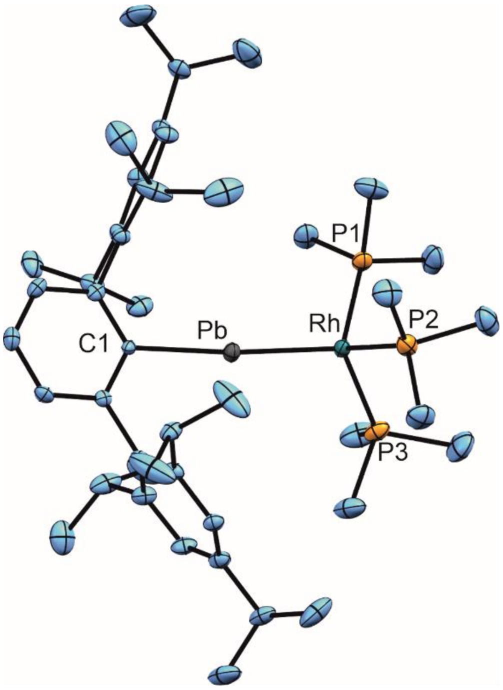

Figure SI05. ORTEP of the molecular structure of **6**. Ellipsoids set at 50% probability. Hydrogen atoms are omitted for clarity. Interatomic distances (Å) and angles (°): Rh-Pb 2.4457(3), C1-Pb 2.299(3), Rh-P1 2.254(1), Rh-P2 2.2615(9), Rh-P3 2.266(1), C1-Pb-Rh 174.9(1).

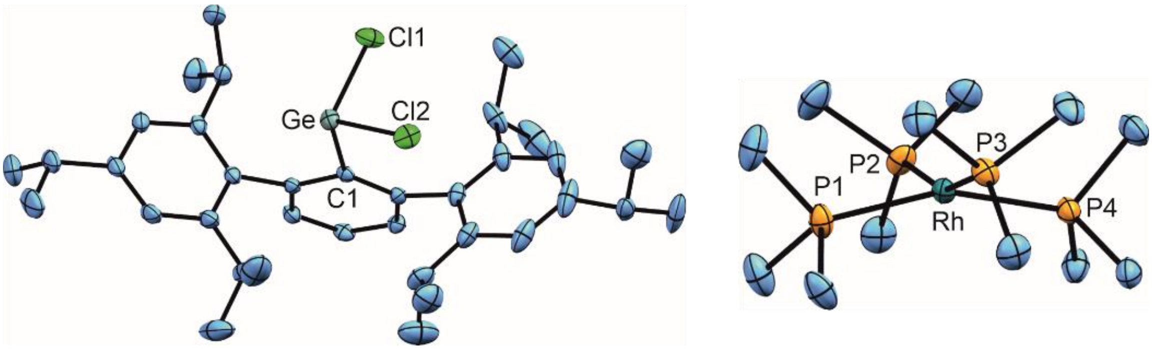

Figure SI06. ORTEPs of the cation and anion of **8**. Ellipsoids set at 50% probability. Hydrogen atoms are omitted for clarity. Interatomic distances (Å) and angles (°): Ge-C1 2.0460(17), Ge-Cl1 2.3053(5), Ge-Cl2 2.3338(5), Rh-P1 2.3102(5) Rh-P2 2.3030(5), Rh-P4 2.3080(5), Rh-P3 2.3095(5), P2-Rh-P3 158.3(1), P1-Rh-P4 158.7(1).

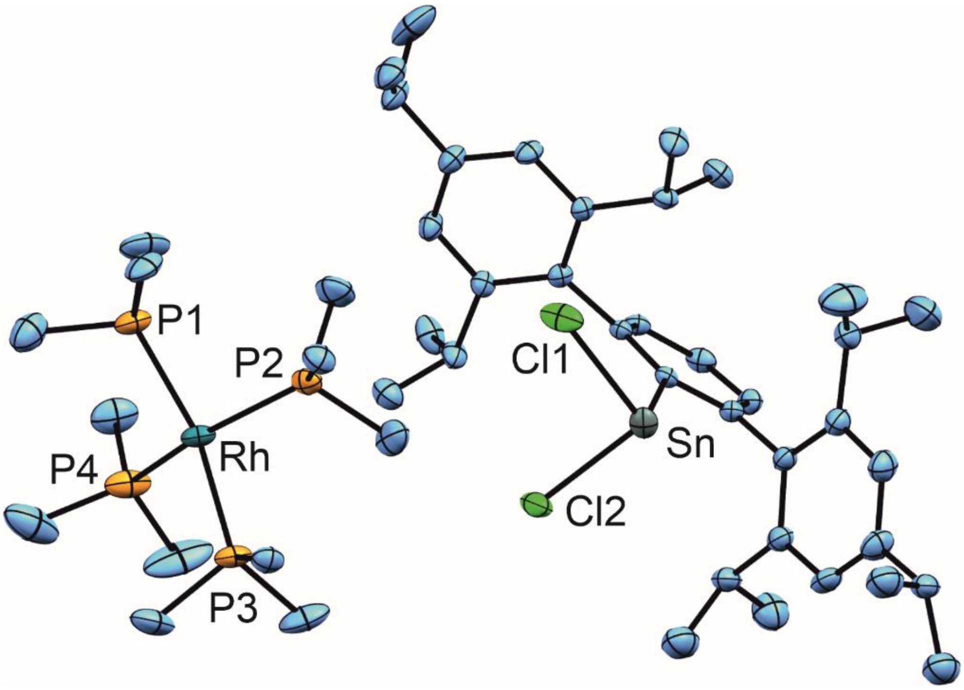

Figure SI07. ORTEP of the molecular structure of **9**. Ellipsoids set at 50% probability. Hydrogen atoms are omitted for clarity. Interatomic distances (Å) and angles (°): Sn-Cl1 2.5074(7), Sn-Cl2 2.5222(6), Rh-P1 2.2964(6), Rh-P2 2.322(4), Rh-P3 2.2856(6), Rh-P4 2.2737(6), P1-Rh-P3 147.47(2), P2-Rh-P4 146.52(7).

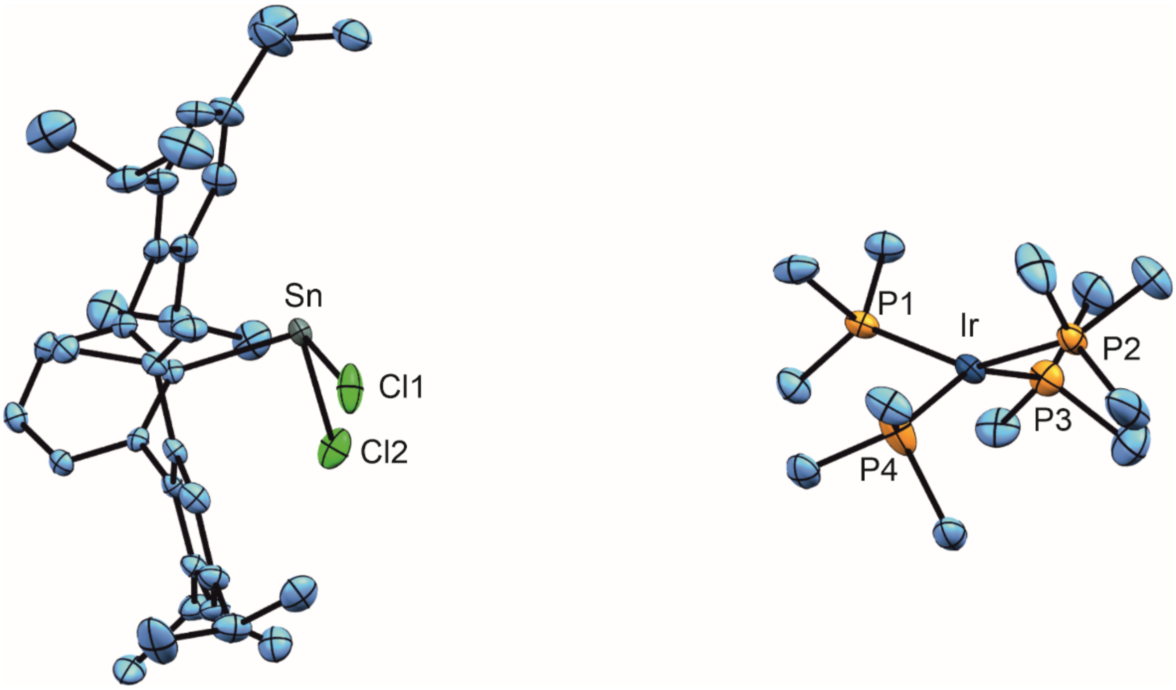

Figure SI08. ORTEP of the molecular structure of **10**. Ellipsoids set at 50% probability. Hydrogen atoms are omitted for clarity. Interatomic distances (Å) and angles (°): Sn-Cl1 2.5073(11), Sn-Cl2 2.5215(10), Ir-P1 2.2895(10), Ir-P2 2.2989(11), Ir-P3 2.2824(10), Ir-P4 2.2729(10), P1-Ir-P3 147.19(4), P2-Ir-P4 149.37(5).

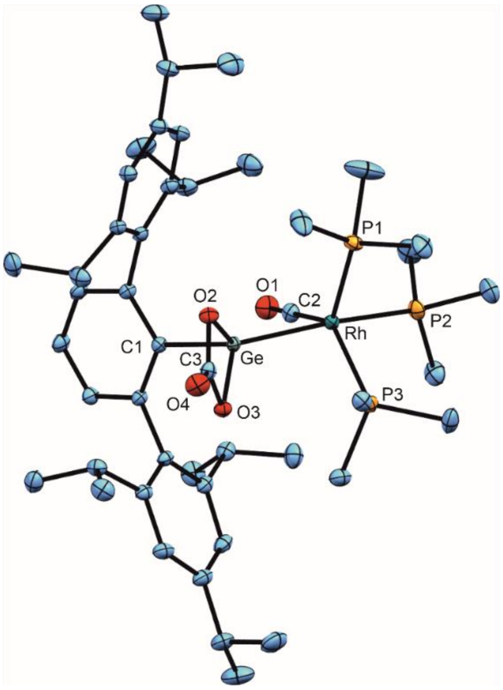

Figure SI09. ORTEP of the molecular structure of **11**. Ellipsoids set at 50% probability. Hydrogen atoms are omitted for clarity. Interatomic distances (Å) and angles (°): Rh-Ge 2.39360(15), Rh-P1 2.3859(3), Rh-P2 2.3304(3), Rh-P3 2.3870(3), Rh-C2 1.8659(12), C2-O1 1.1541(15), Ge-O2 1.9068(8), Ge-O3 1.9091(8), C3-O2 1.3384(14), C3-O3 1.3408(14), C3-O4 1.2124(14), O2-Ge-O3 69.6(1), C1-Ge-Rh 125.8(1).

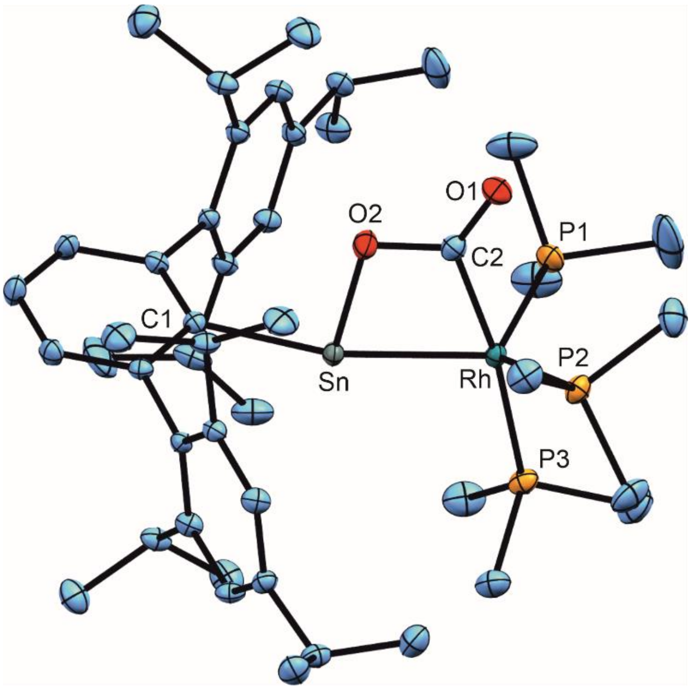

Figure SI10. ORTEP of the molecular structure of **13**. Ellipsoids set at 50% probability. Hydrogen atoms are omitted for clarity. Interatomic distances (Å) and angles (°): Rh-Sn 2.4896(2), Rh-C2 2.097(2), Rh-P1 2.2802(6), Rh-P2 2.3068(6), Rh-P3 2.3279(6), C2-O2 1.359(3), C2-O1 1.218(3), O2-C2-O1 119.1(2), Rh-C2-O1 129.1(2), Rh-C2-O2 111.8(2), C1-Sn-Ir 167.62(5), P1-Ir-C2 171.0(1).

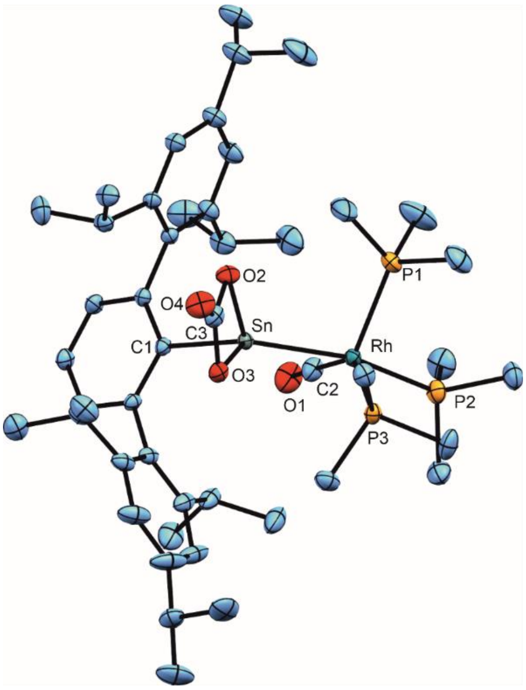

Figure SI11. ORTEP of the molecular structure of **14**. Ellipsoids set at 50% probability. Hydrogen atoms are omitted for clarity. Interatomic distances (Å) and angles (°): Rh-Sn 2.55119(17), Rh-P1 2.3754(5), Rh-P2 2.3077(5), Rh-P3 2.3781(4), Rh-C2 1.8716(18), C2-O1 1.154(2), Sn-O2 2.0983(12), Sn-O3 2.0939(12), C3-O2 1.336(2), C3-O3 1.338(2), C3-O4 1.219(2), O2-Sn-O3 63.5(1), C1-Sn-Rh 127.6(1).

NMR Spectroscopy

NMR spectra of Ar*Ge≡Co(PMe_3)$_3$ (**1**)

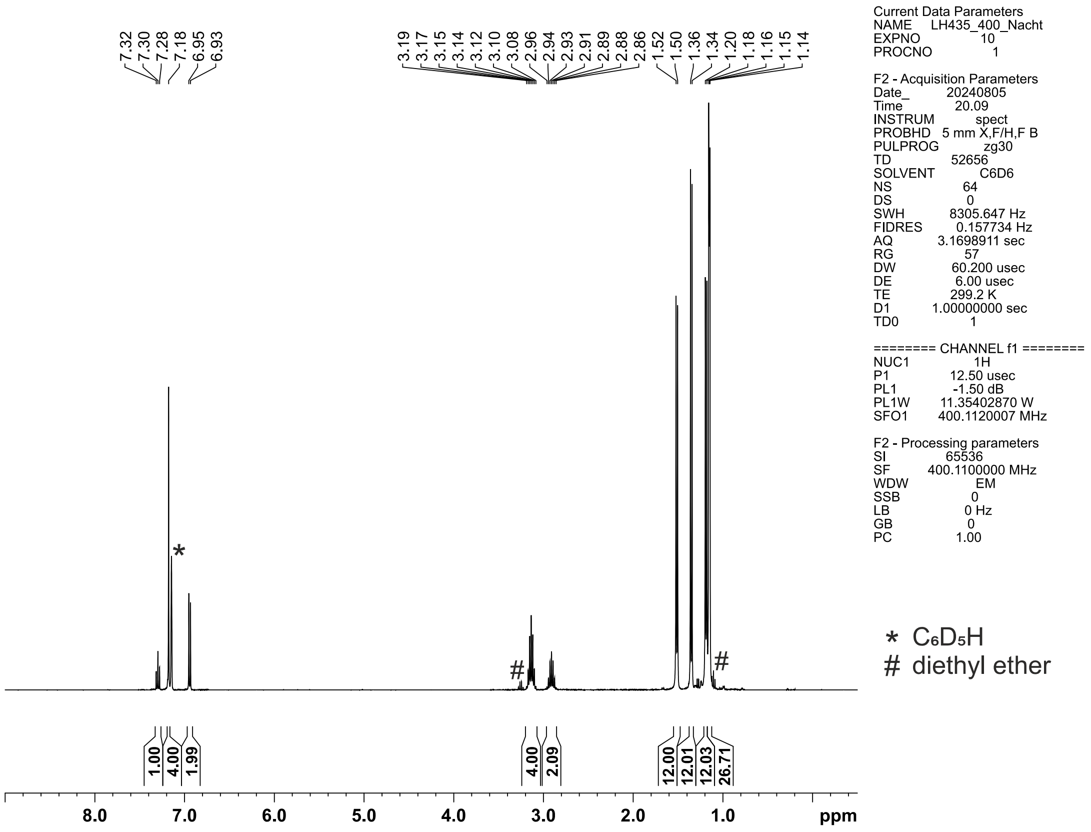

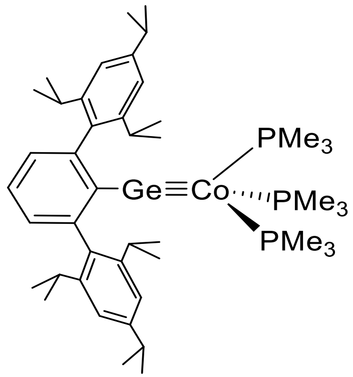

Figure SI12. ^{1}H NMR of compound **1** (0.5–9 ppm).

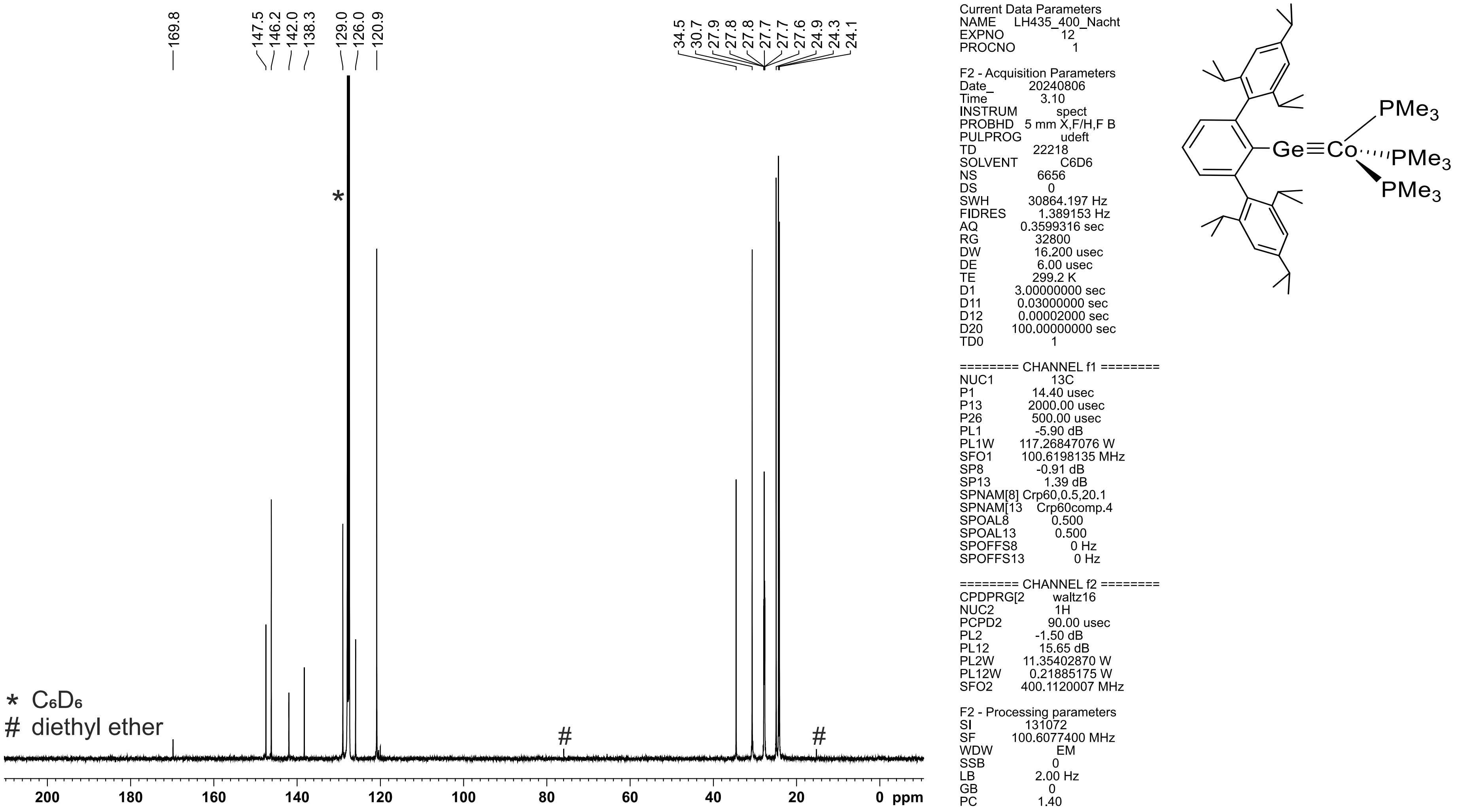

Figure SI13. $^{13}C\{^{1}H\}$ NMR of compound **1**.

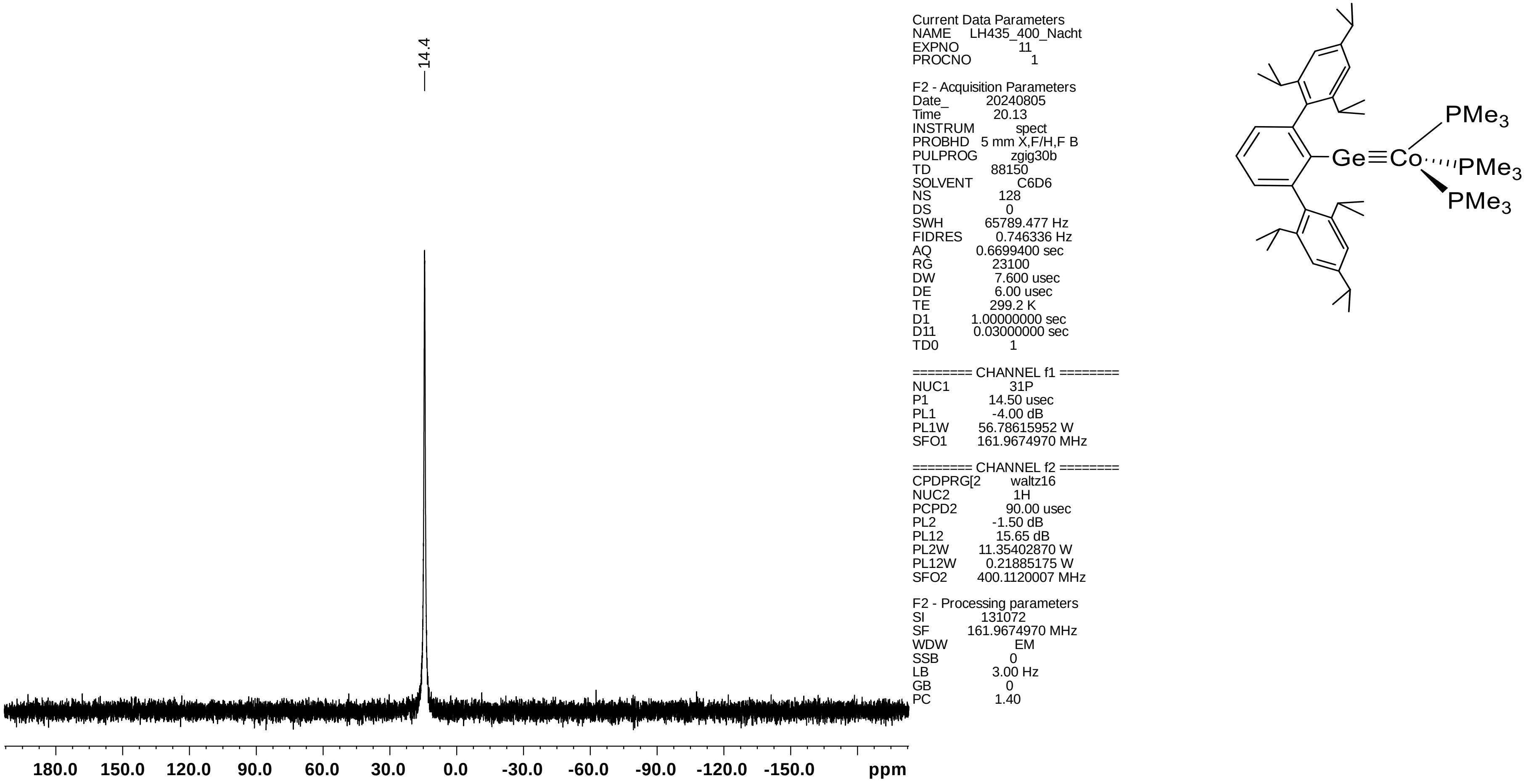

Figure SI14. $^{31}P\{^{1}H\}$ NMR of compound **1**.

NMR spectra of Ar*Ge≡Rh(PMe$_3$)$_3$ (**2**)

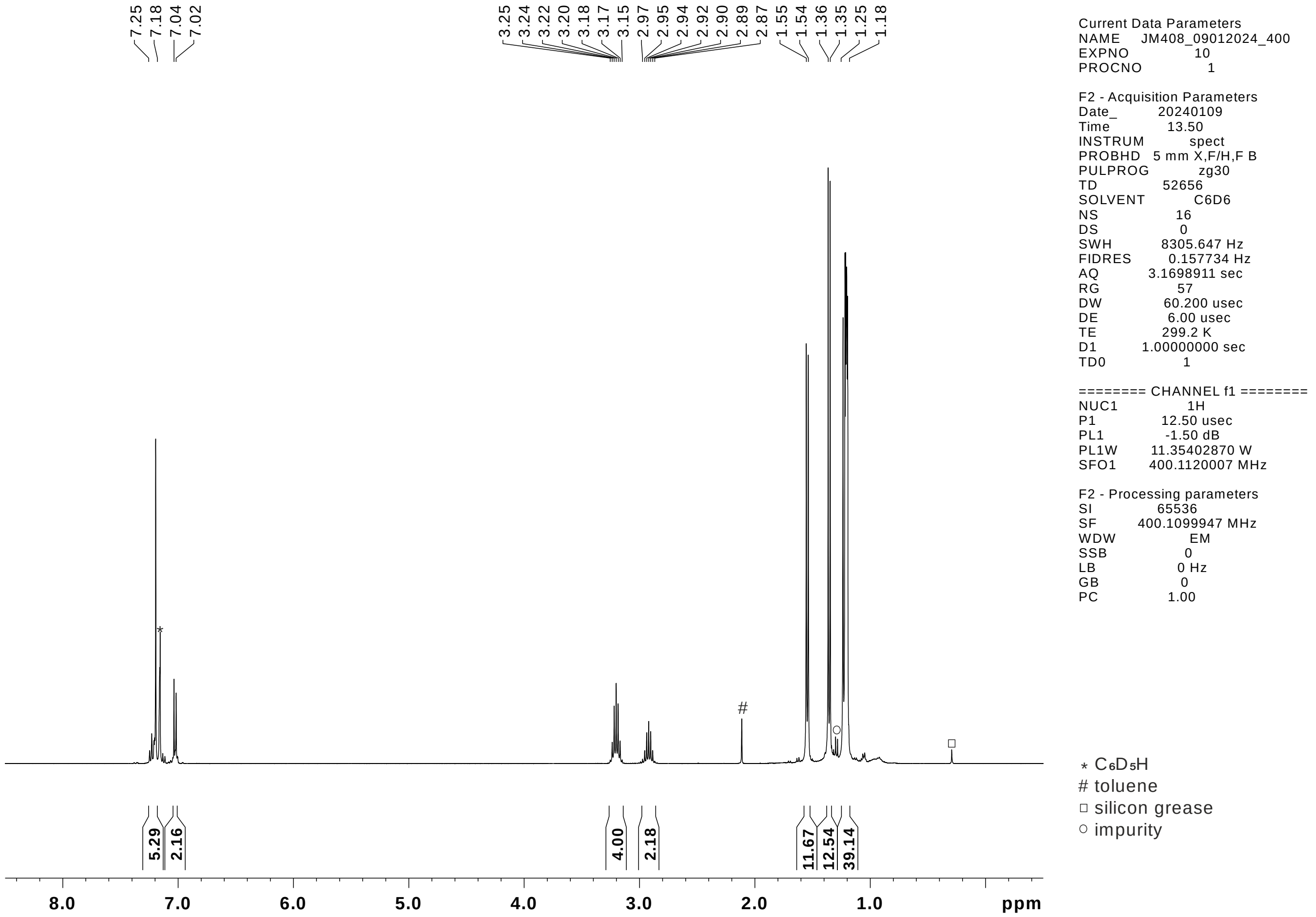

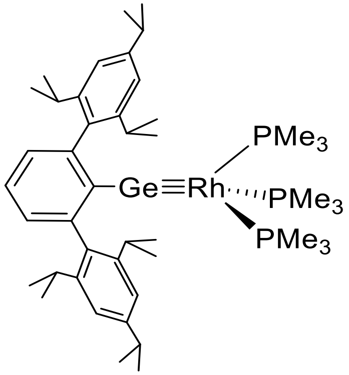

Figure SI15. ^{1}H NMR of compound **2**.

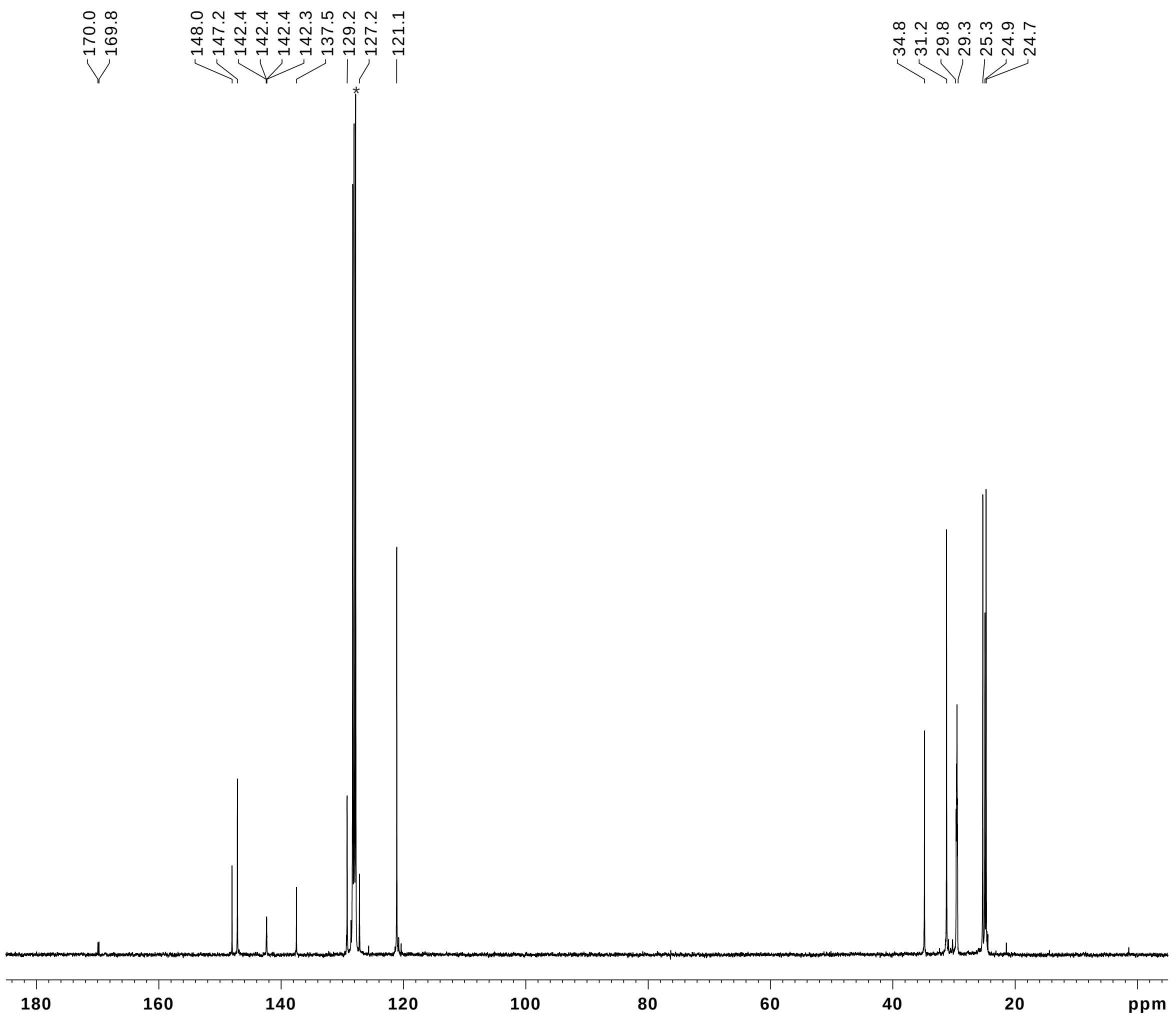

Current Data Parameters
NAME JM408_09012024_400
EXPNO 14
PROCNO 1

F2 - Acquisition Parameters
Date_ 20240110
Time 2.19
INSTRUM spect
PROBHD 5 mm X,F/H,F B
PULPROG udeft
TD 22218
SOLVENT C6D6
NS 5837
DS 0
SWH 30864.197 Hz
FIDRES 1.389153 Hz
AQ 0.3599316 sec
RG 32800
DW 16.200 usec
DE 6.00 usec
TE 299.2 K
D1 3.00000000 sec
D11 0.03000000 sec
D12 0.00002000 sec
D20 100.00000000 sec
TD0 1

======== CHANNEL f1 ========
NUC1 13C
P1 14.40 usec
P13 2000.00 usec
P26 500.00 usec
PL1 -5.90 dB
PL1W 117.26847076 W
SFO1 100.6198135 MHz
SP8 -0.91 dB
SP13 1.39 dB
SPNAM[8] Crp60,0.5,20.1
SPNAM[13 Crp60comp.4
SPOAL8 0.500
SPOAL13 0.500
SPOFFS8 0 Hz
SPOFFS13 0 Hz

======== CHANNEL f2 ========
CPDPRG[2 waltz16
NUC2 1H
PCPD2 90.00 usec
PL2 -1.50 dB
PL12 15.65 dB
PL2W 11.35402870 W
PL12W 0.21885175 W
SFO2 400.1120007 MHz

F2 - Processing parameters
SI 131072
SF 100.6077020 MHz
WDW EM
SSB 0
LB 2.00 Hz
GB 0
PC 1.40

* C_6D_6

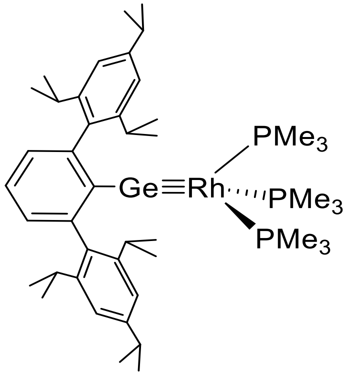

Figure SI16. $^{13}C\{^{1}H\}$ NMR of compound **2**.

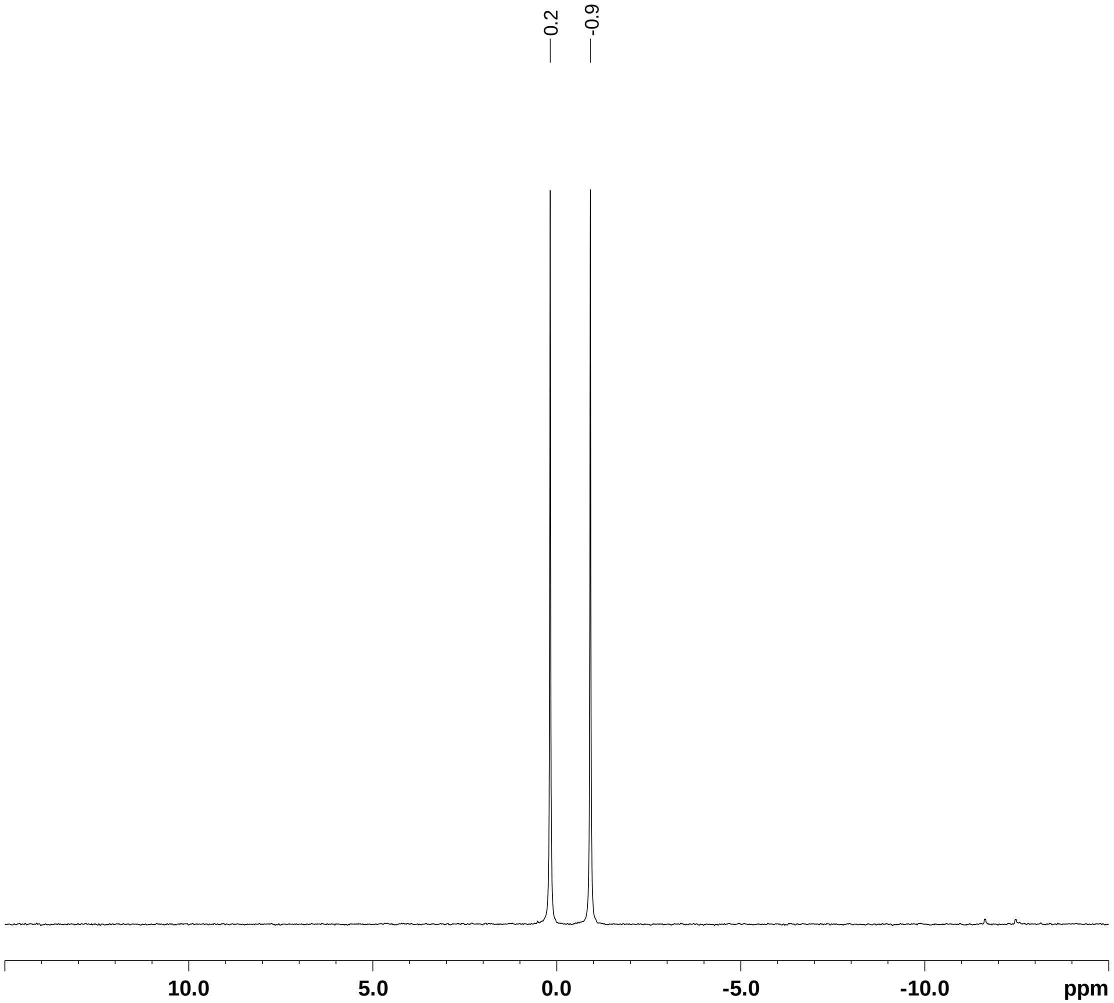

Current Data Parameters
NAME JM408_09012024_400
EXPNO 11
PROCNO 1

F2 - Acquisition Parameters
Date_ 20240109
Time 13.54
INSTRUM spect
PROBHD 5 mm X,F/H,F B
PULPROG zgig30b
TD 88150
SOLVENT C6D6
NS 128
DS 0
SWH 65789.477 Hz
FIDRES 0.746336 Hz
AQ 0.6699400 sec
RG 23100
DW 7.600 usec
DE 6.00 usec
TE 299.2 K
D1 1.00000000 sec
D11 0.03000000 sec
TD0 1

======== CHANNEL f1 ========
NUC1 31P
P1 14.50 usec
PL1 -4.00 dB
PL1W 56.78615952 W
SFO1 161.9674970 MHz

======== CHANNEL f2 ========
CPDPRG[2 waltz16
NUC2 1H
PCPD2 90.00 usec
PL2 -1.50 dB
PL12 15.65 dB
PL2W 11.35402870 W
PL12W 0.21885175 W
SFO2 400.1120007 MHz

F2 - Processing parameters
SI 131072
SF 161.9674970 MHz
WDW EM
SSB 0
LB 3.00 Hz
GB 0
PC 1.40

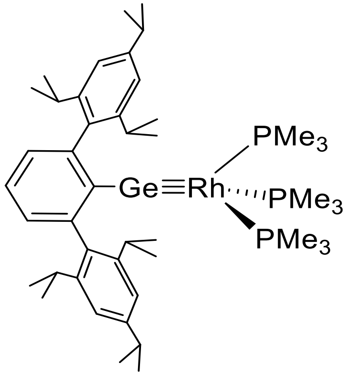

Figure SI17. $^{31}P\{^1H\}$ NMR of compound **2**.

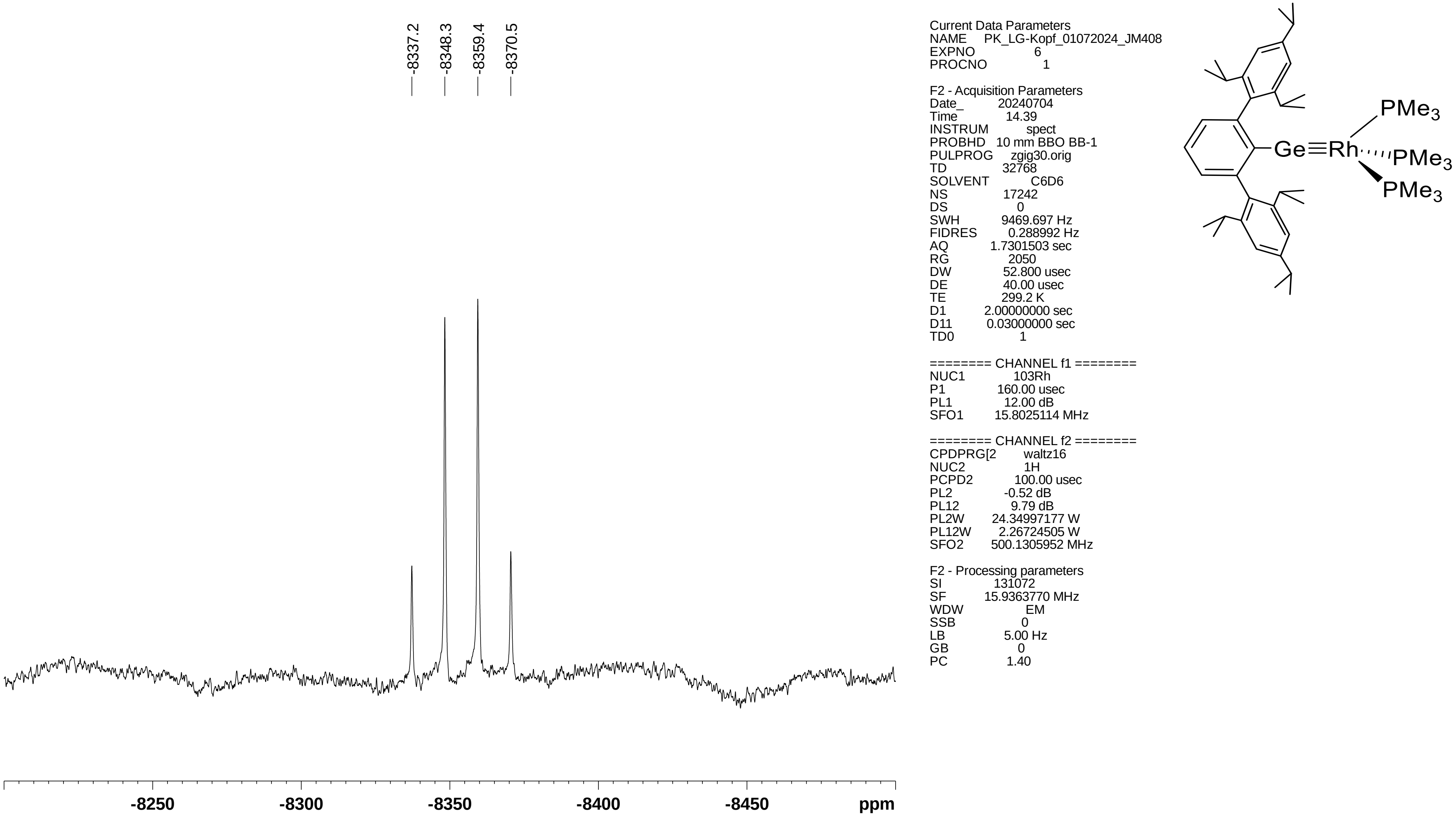

Figure SI18. ^{103}Rh{^{1}H} NMR of compound **2**.

NMR spectra of Ar*Ge≡Ir(PMe_3)$_3$ (**3**)

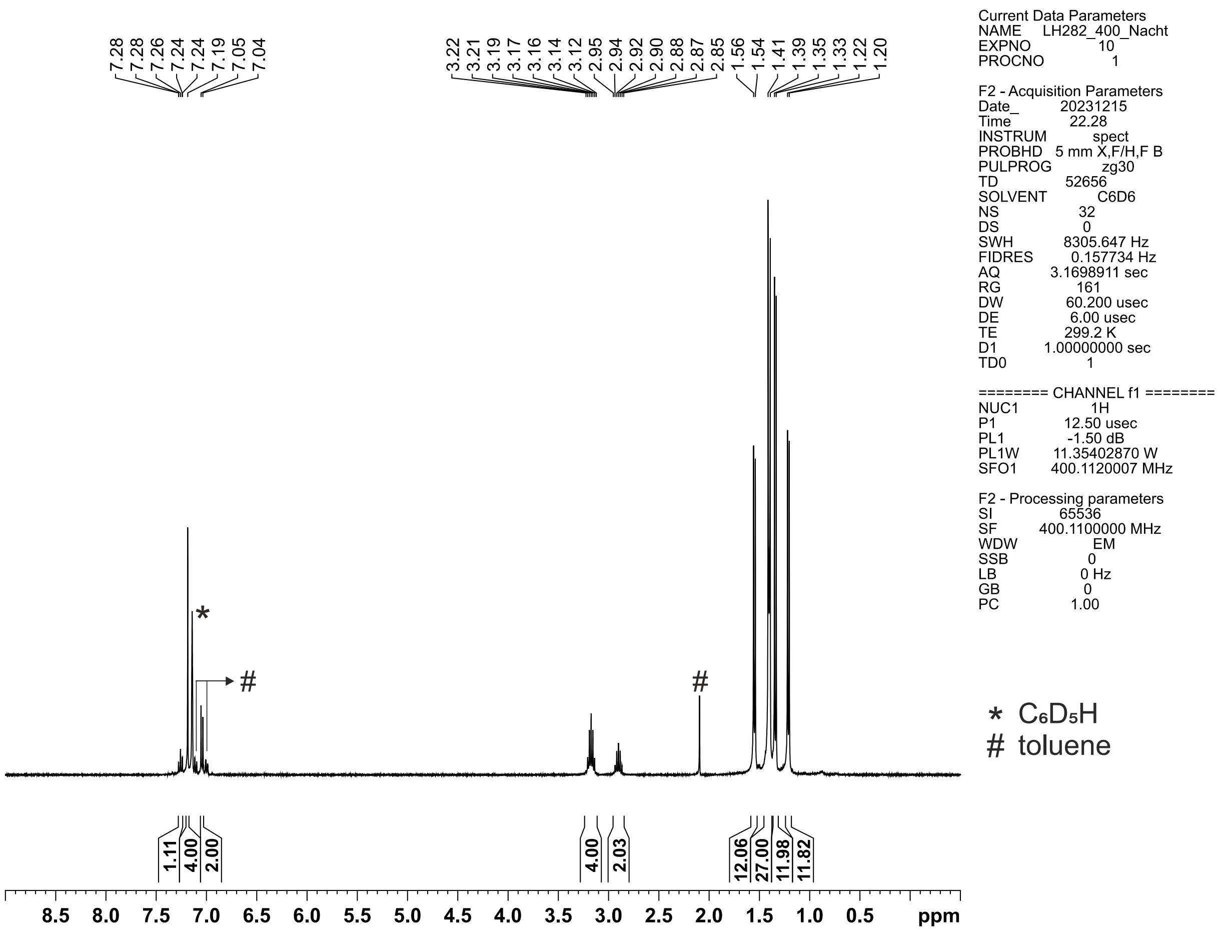

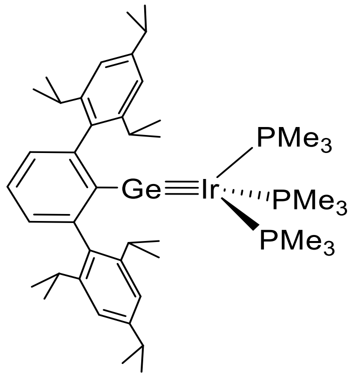

Figure SI19. ^{1}H NMR of compound **3**.

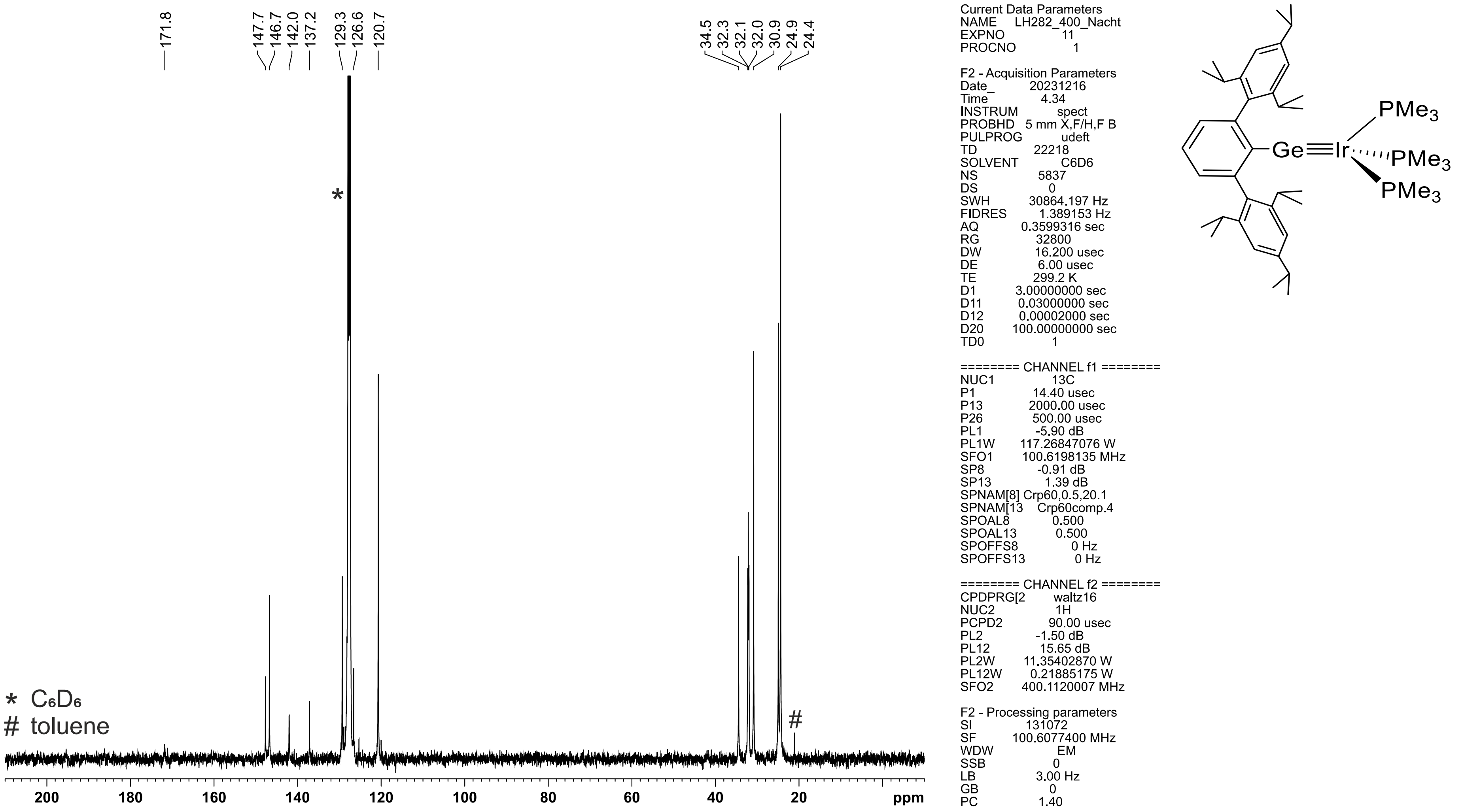

Figure SI20. $^{13}C\{^{1}H\}$ NMR of compound **3**.

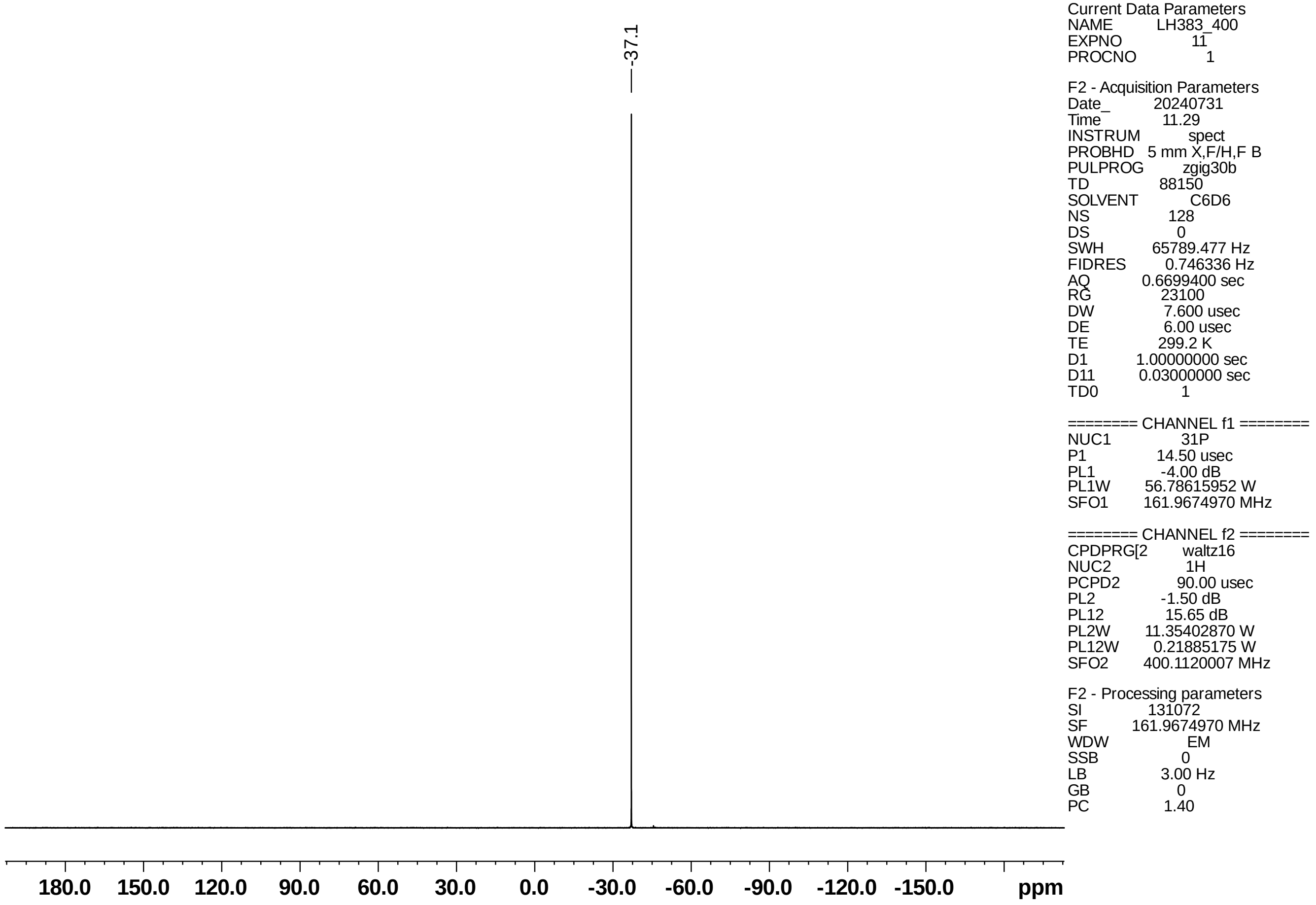

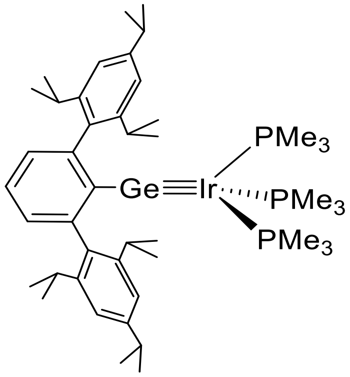

Figure SI21. $^{31}P\{^{1}H\}$ NMR of compound **3**.

NMR spectra of Ar*Sn≡Rh(PMe₃)₃ (**4**)

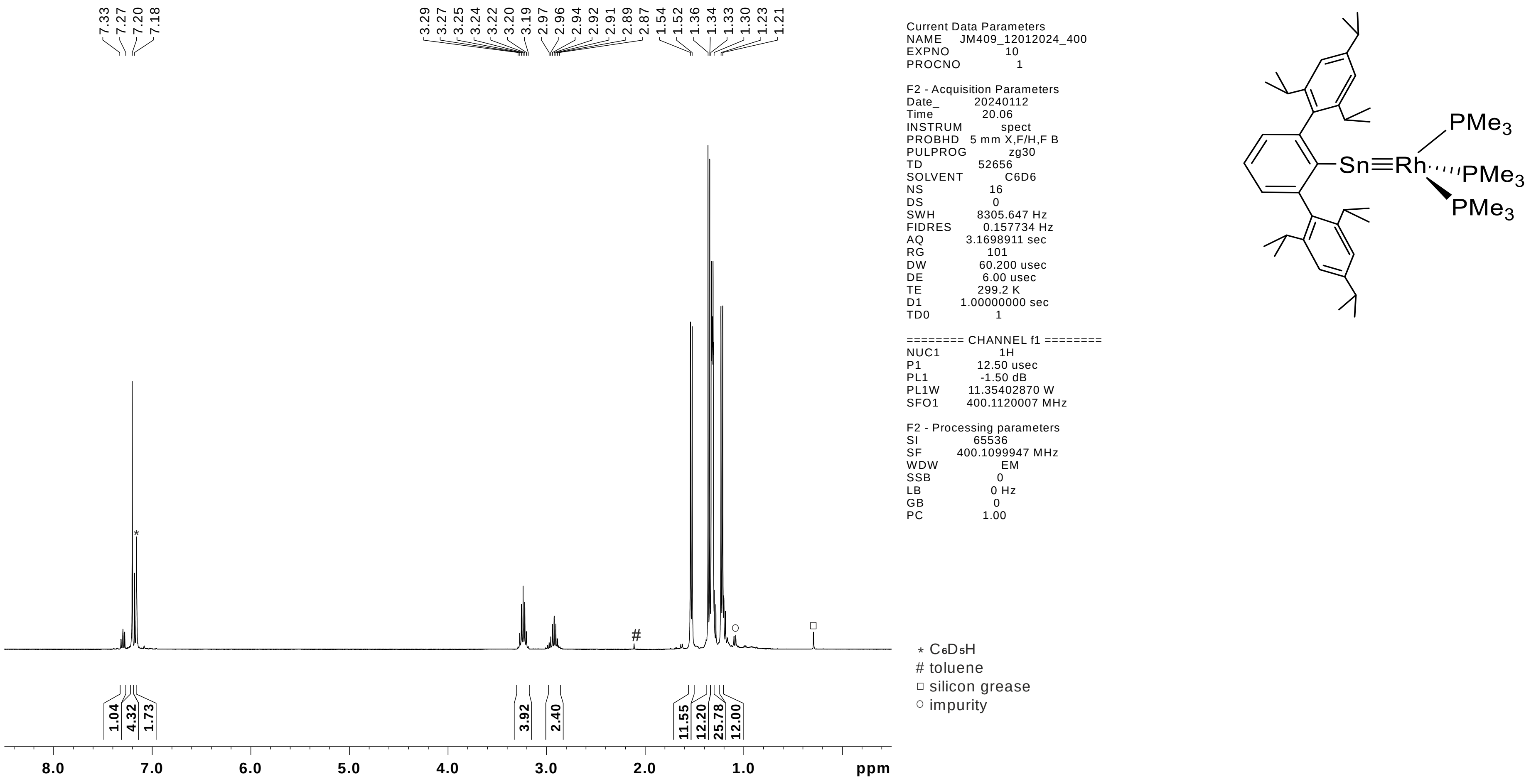

Figure SI22. ^{1}H NMR of compound **4**.

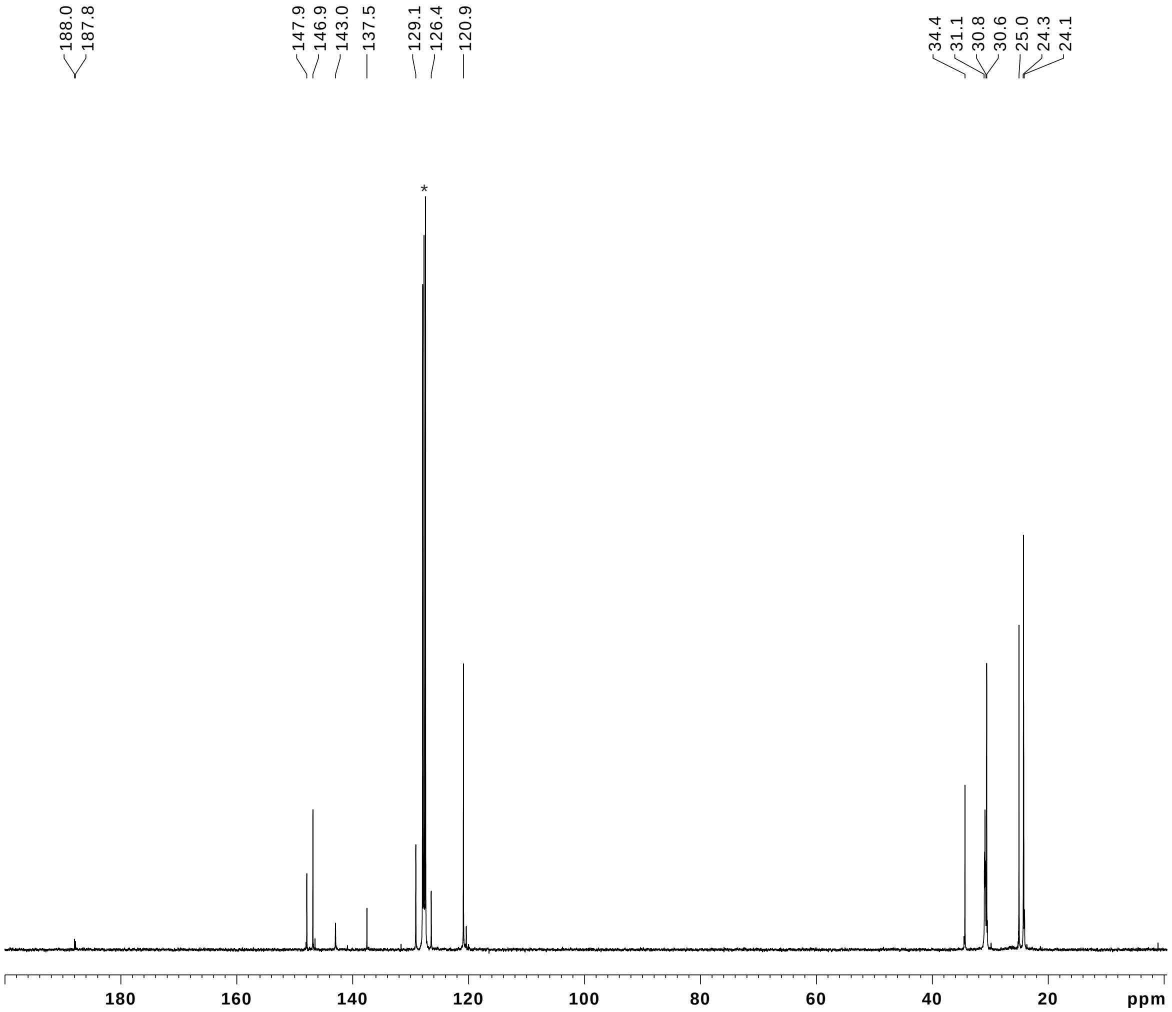

Current Data Parameters
NAME JM409_12012024_400
EXPNO 12
PROCNO 1

F2 - Acquisition Parameters
Date_ 20240113
Time 2.19
INSTRUM spect
PROBHD 5 mm X,F/H,F B
PULPROG udeft
TD 22218
SOLVENT C6D6
NS 5837
DS 0
SWH 30864.197 Hz
FIDRES 1.389153 Hz
AQ 0.3599316 sec
RG 32800
DW 16.200 usec
DE 6.00 usec
TE 299.2 K
D1 3.00000000 sec
D11 0.03000000 sec
D12 0.00002000 sec
D20 100.00000000 sec
TD0 1

======== CHANNEL f1 ========
NUC1 13C
P1 14.40 usec
P13 2000.00 usec
P26 500.00 usec
PL1 -5.90 dB
PL1W 117.26847076 W
SFO1 100.6198135 MHz
SP8 -0.91 dB
SP13 1.39 dB
SPNAM[8] Crp60,0.5,20.1
SPNAM[13 Crp60comp.4
SPOAL8 0.500
SPOAL13 0.500
SPOFFS8 0 Hz
SPOFFS13 0 Hz

======== CHANNEL f2 ========
CPDPRG[2 waltz16
NUC2 1H
PCPD2 90.00 usec
PL2 -1.50 dB
PL12 15.65 dB
PL2W 11.35402870 W
PL12W 0.21885175 W
SFO2 400.1120007 MHz

F2 - Processing parameters
SI 131072
SF 100.6077400 MHz
WDW EM
SSB 0
LB 2.00 Hz
GB 0
PC 1.40

* C_6D_6

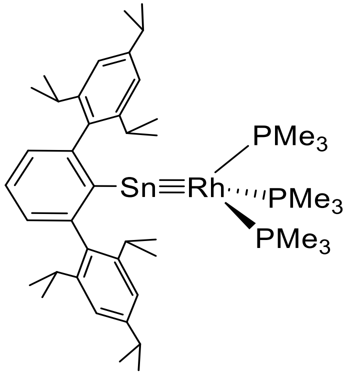

Figure SI23. $^{13}C\{^{1}H\}$ NMR of compound **4**.

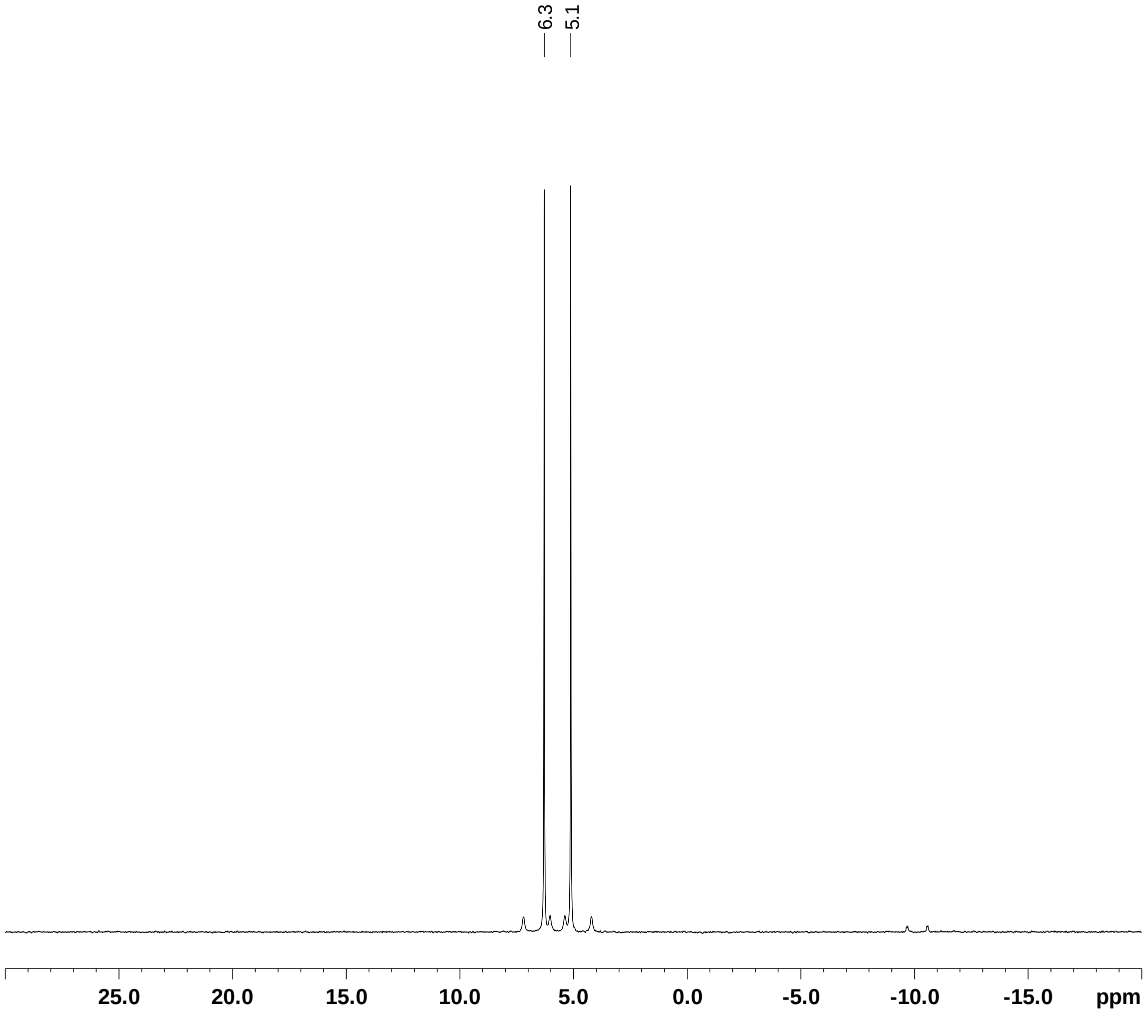

Current Data Parameters
NAME JM409_12012024_400
EXPNO 11
PROCNO 1

F2 - Acquisition Parameters
Date_ 20240112
Time 20.13
INSTRUM spect
PROBHD 5 mm X,F/H,F B
PULPROG zgig30b
TD 88150
SOLVENT C6D6
NS 256
DS 0
SWH 65789.477 Hz
FIDRES 0.746336 Hz
AQ 0.6699400 sec
RG 23100
DW 7.600 usec
DE 6.00 usec
TE 299.2 K
D1 1.00000000 sec
D11 0.03000000 sec
TD0 1

======== CHANNEL f1 ========
NUC1 31P
P1 14.50 usec
PL1 -4.00 dB
PL1W 56.78615952 W
SFO1 161.9674970 MHz

======== CHANNEL f2 ========
CPDPRG[2 waltz16
NUC2 1H
PCPD2 90.00 usec
PL2 -1.50 dB
PL12 15.65 dB
PL2W 11.35402870 W
PL12W 0.21885175 W
SFO2 400.1120007 MHz

F2 - Processing parameters
SI 131072
SF 161.9674970 MHz
WDW EM
SSB 0
LB 3.00 Hz
GB 0
PC 1.40

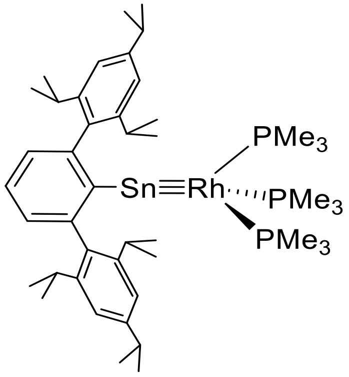

Figure SI24. $^{31}P\{^{1}H\}$ NMR of compound **4**.

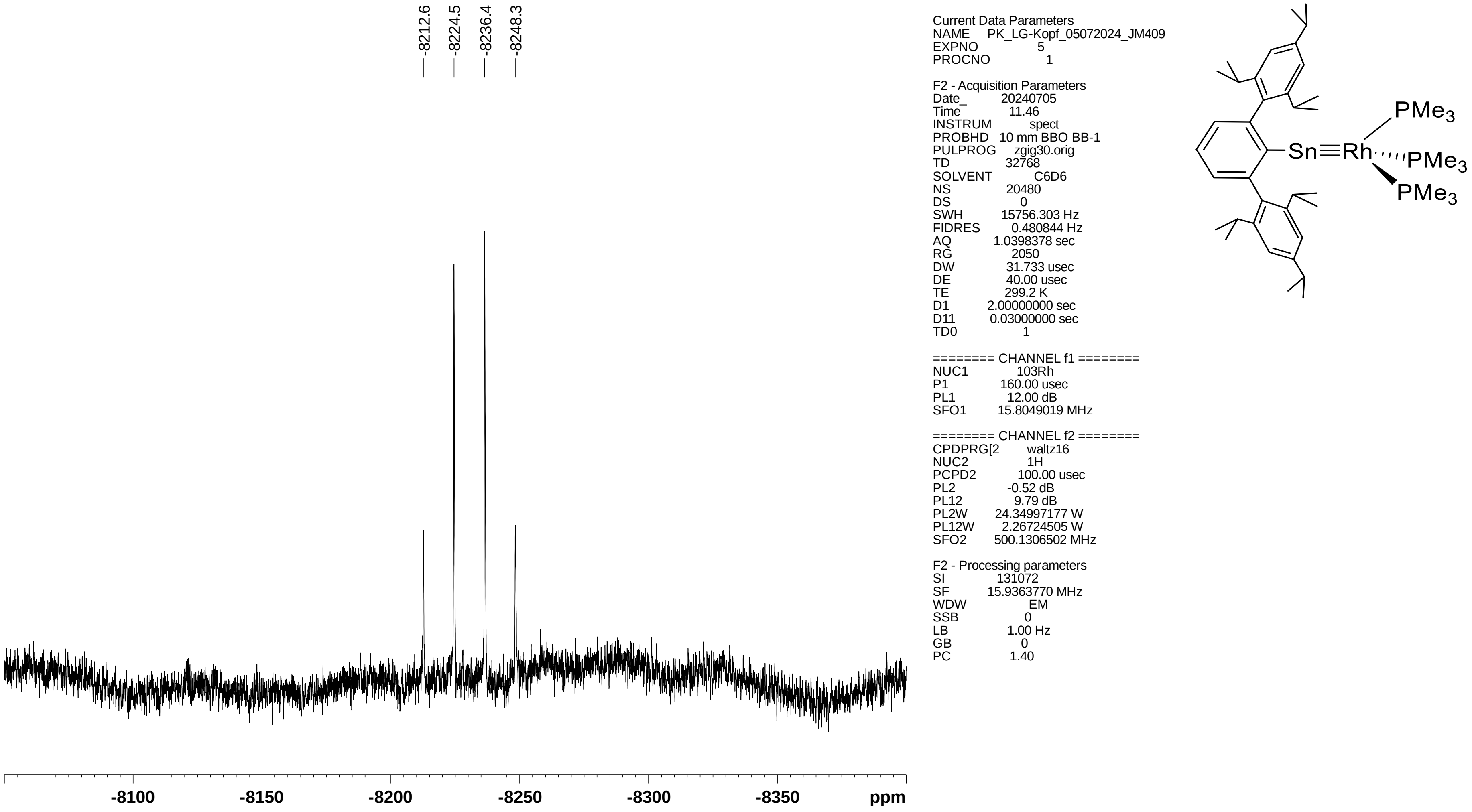

Figure SI25. $^{103}Rh\{^{1}H\}$ NMR of compound **4**.

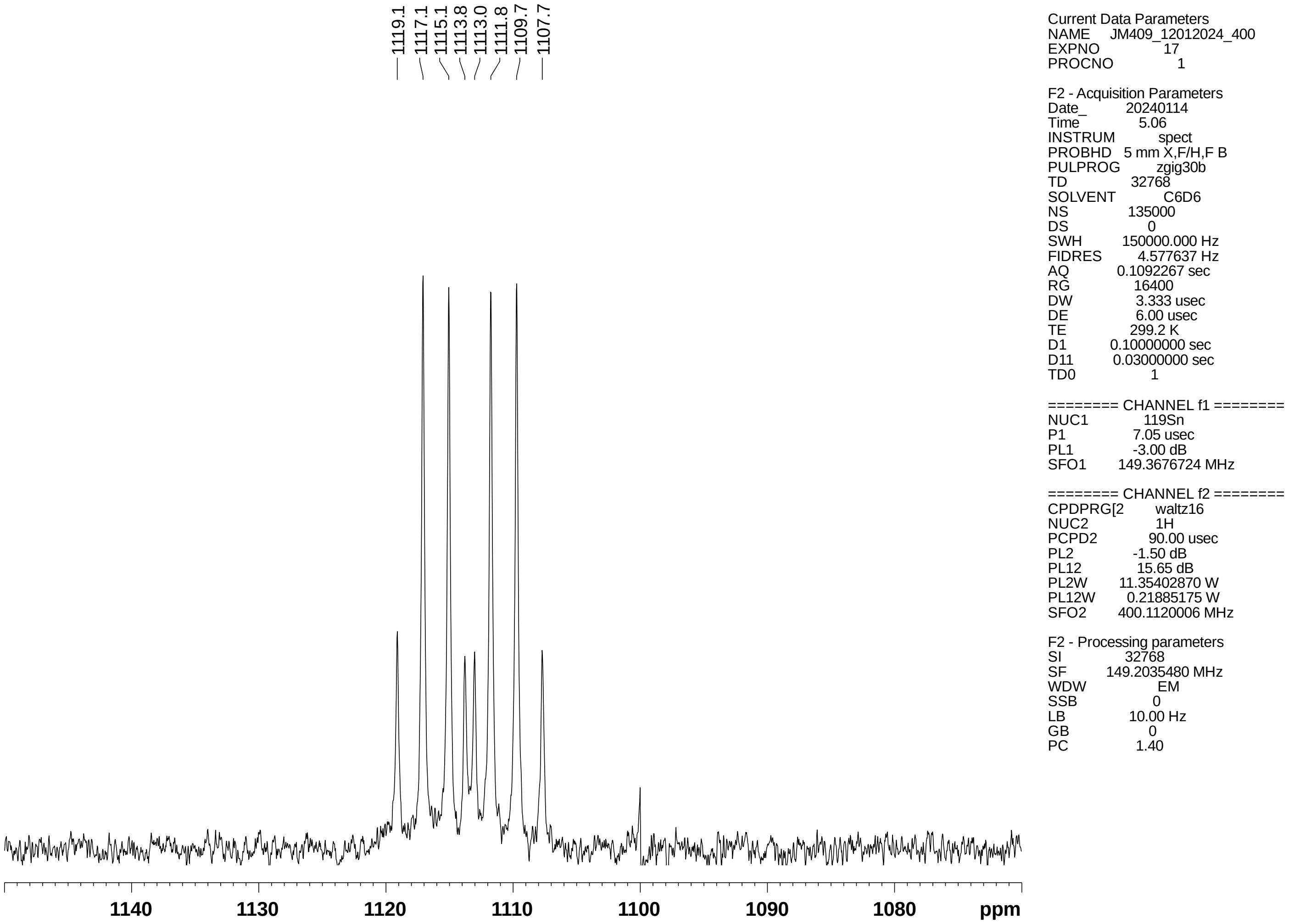

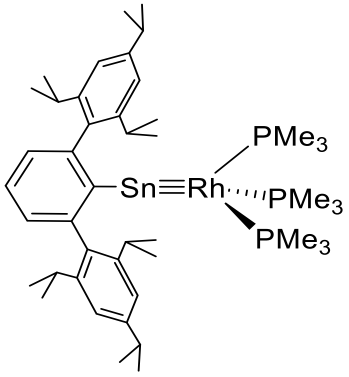

Figure SI26. $^{119}Sn\{^{1}H\}$ NMR of compound **4**.

NMR spectra of Ar*Sn≡Ir(PMe₃)₃ (**5**)

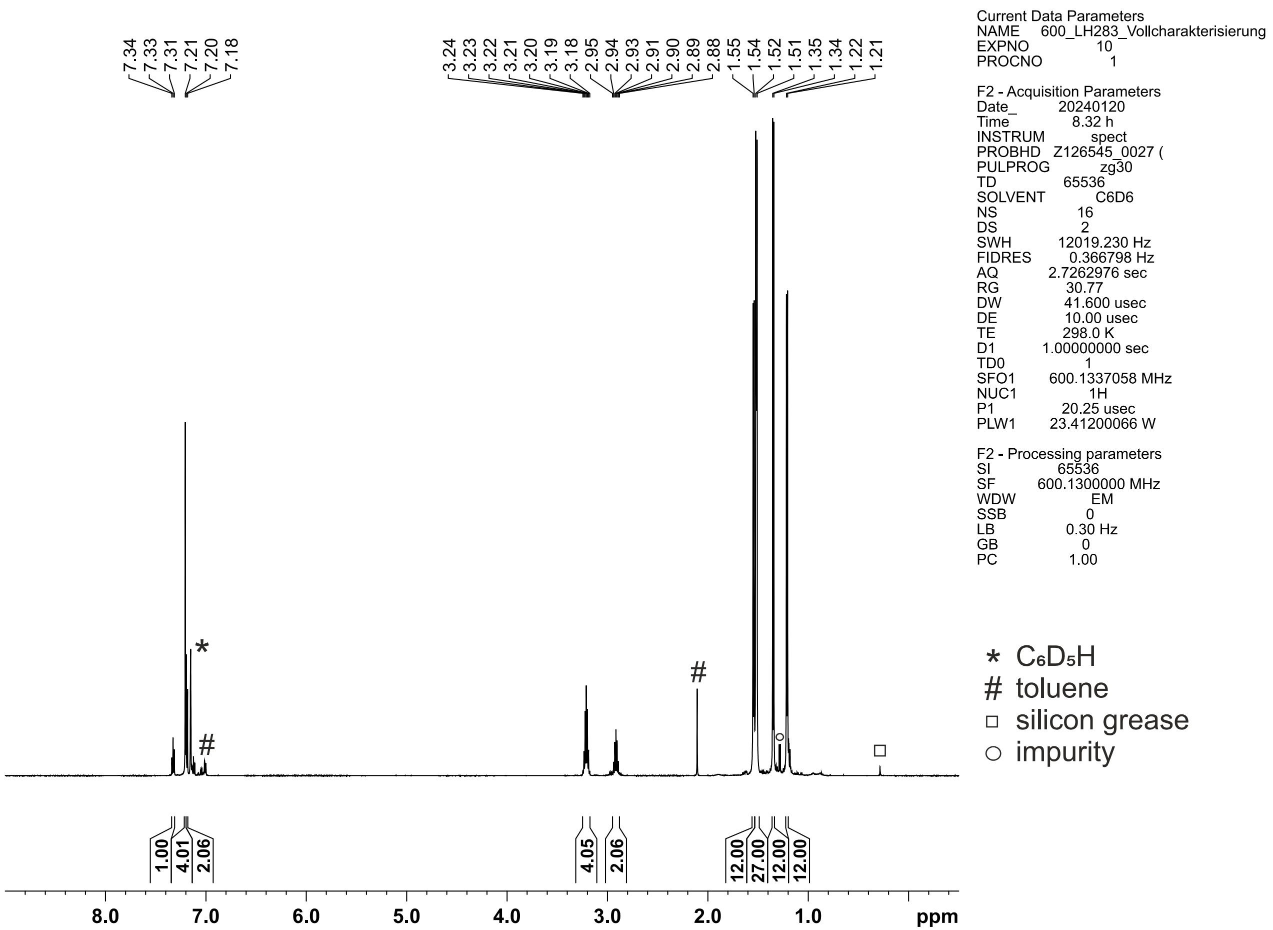

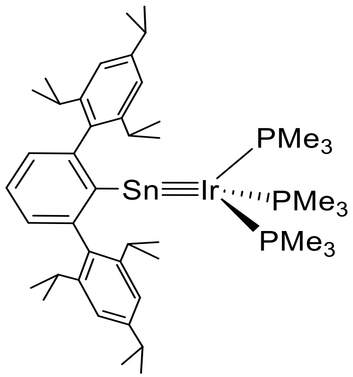

Figure SI27. ^{1}H NMR of compound **5**.

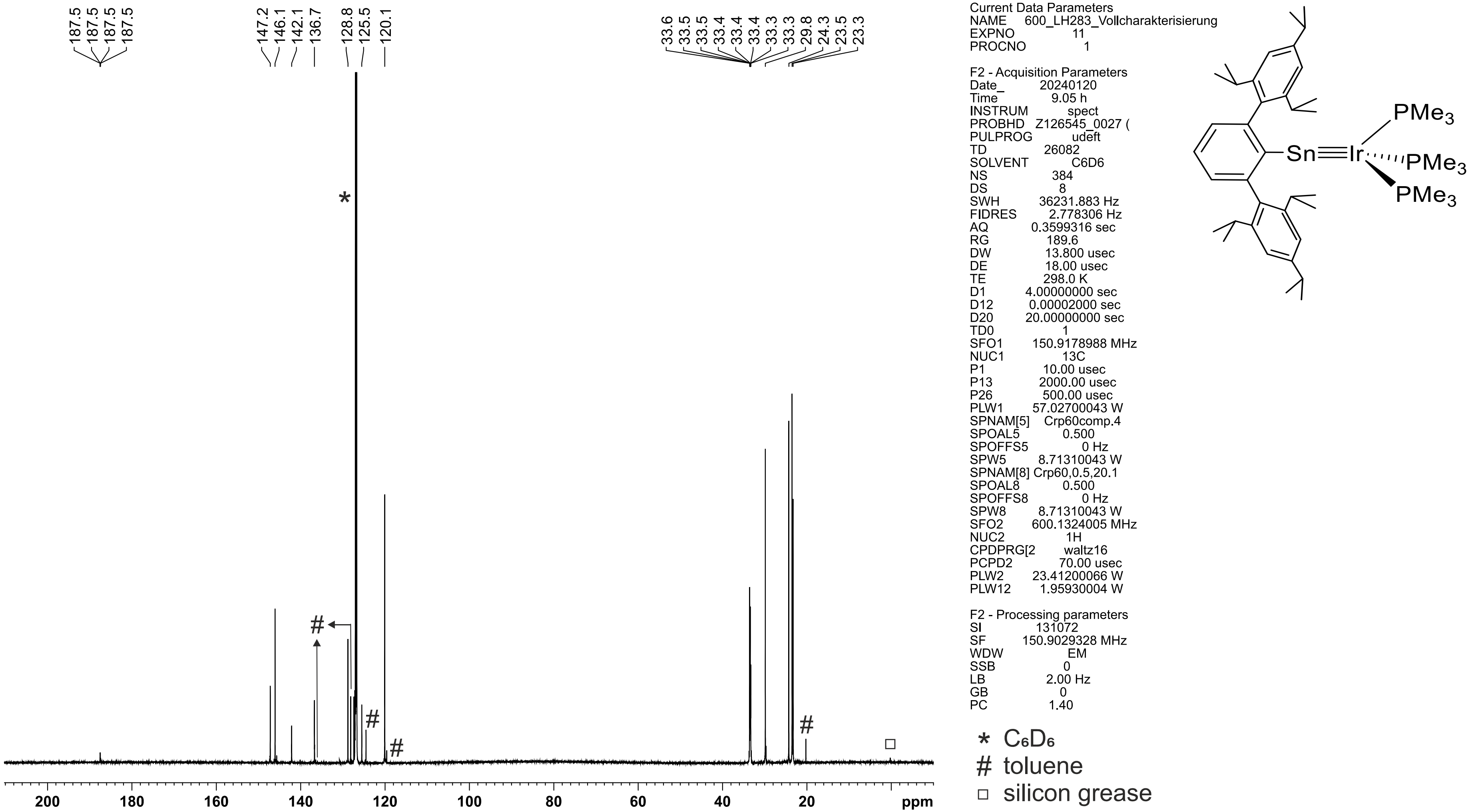

Figure SI28. $^{13}C\{^{1}H\}$ NMR of compound **5**.

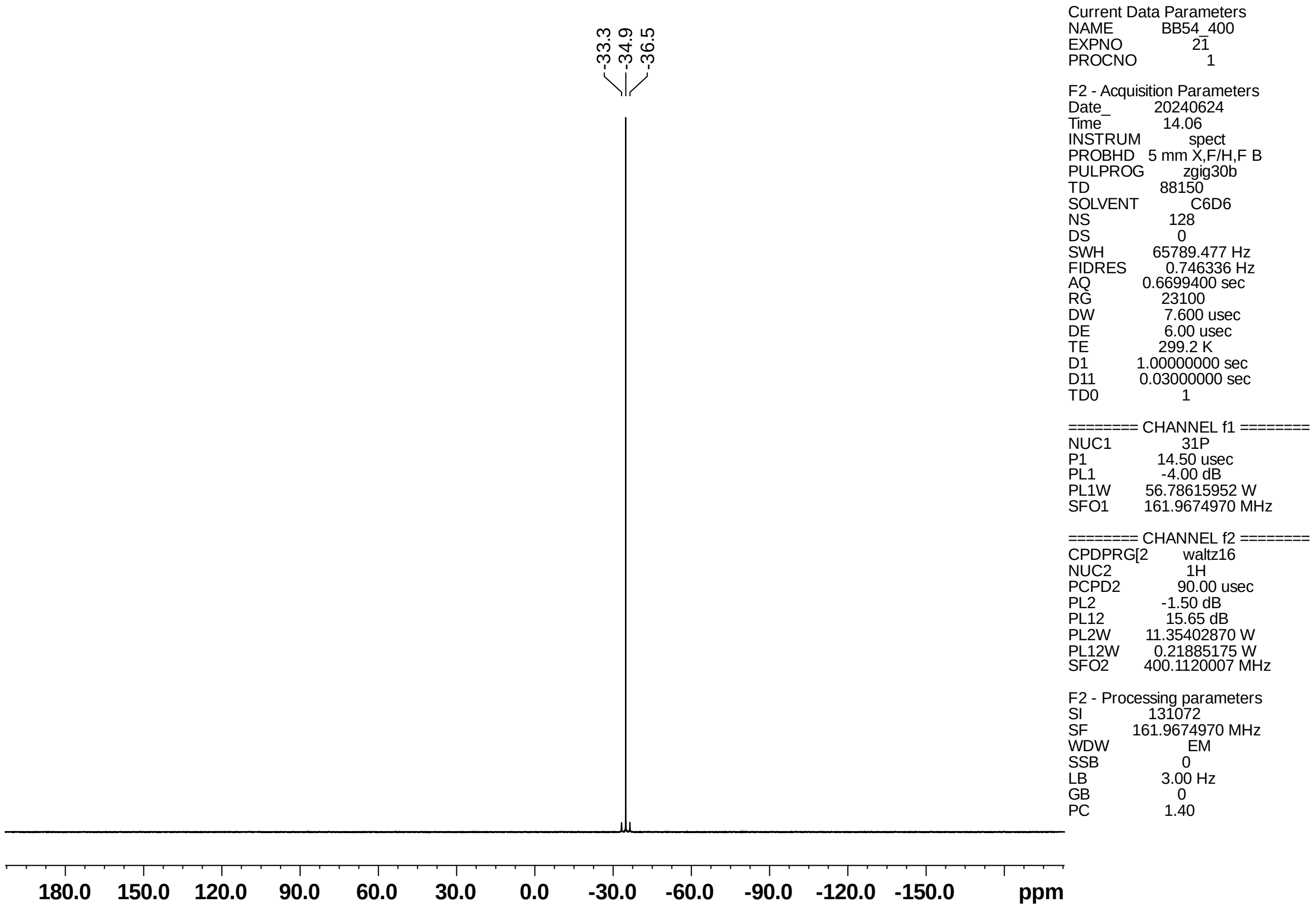

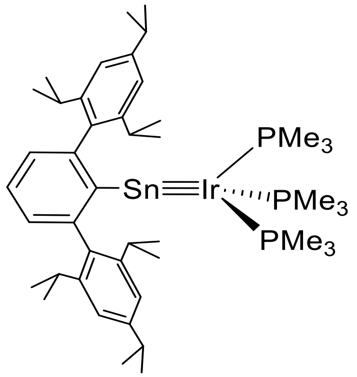

Figure SI29. $^{31}P\{^{1}H\}$ NMR of compound **5**.

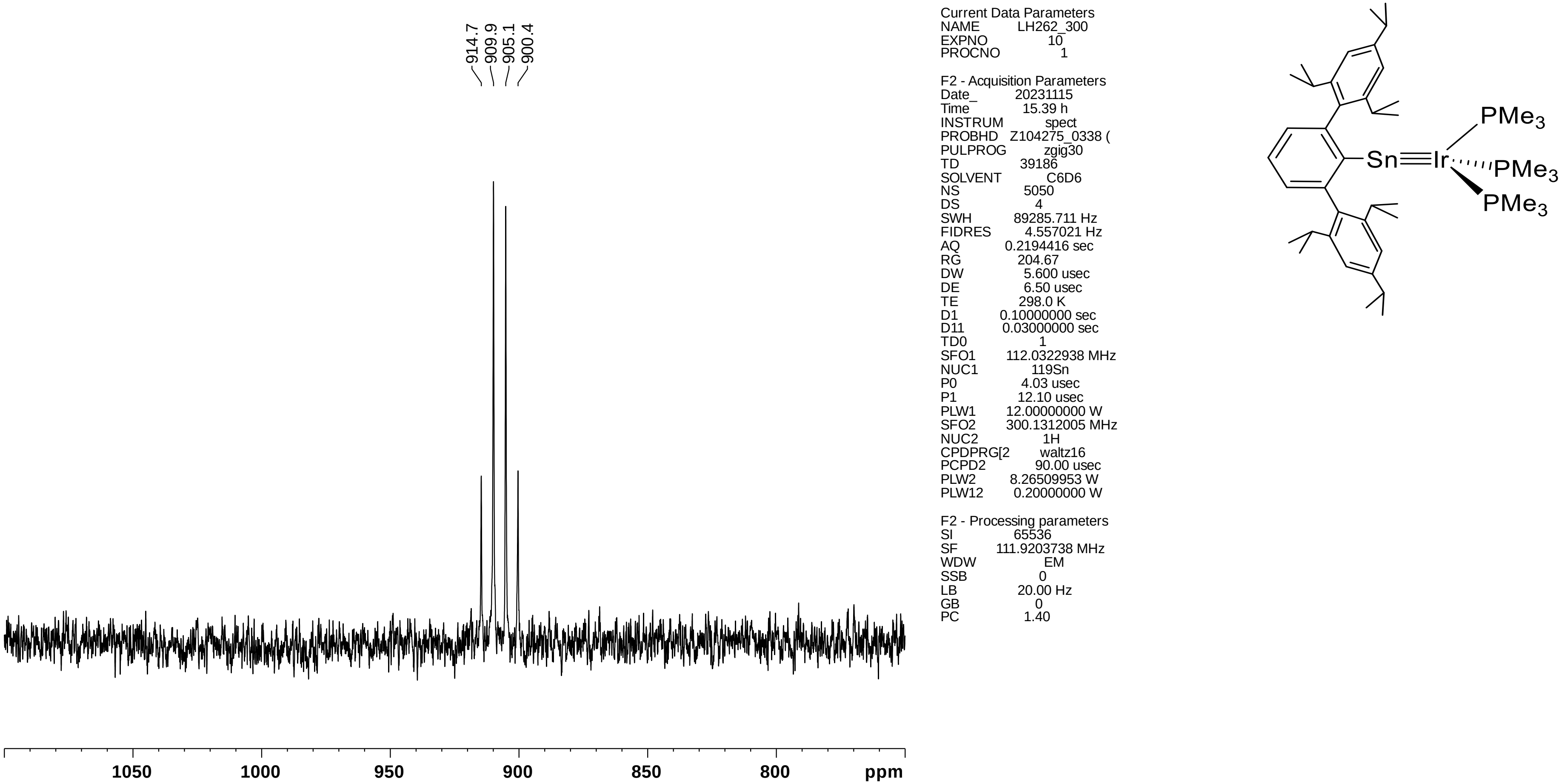

Figure SI30. $^{119}Sn\{^1H\}$ NMR of compound **5**.

NMR spectra of Ar*Pb≡Rh(PMe_3)$_3$ (**6**)

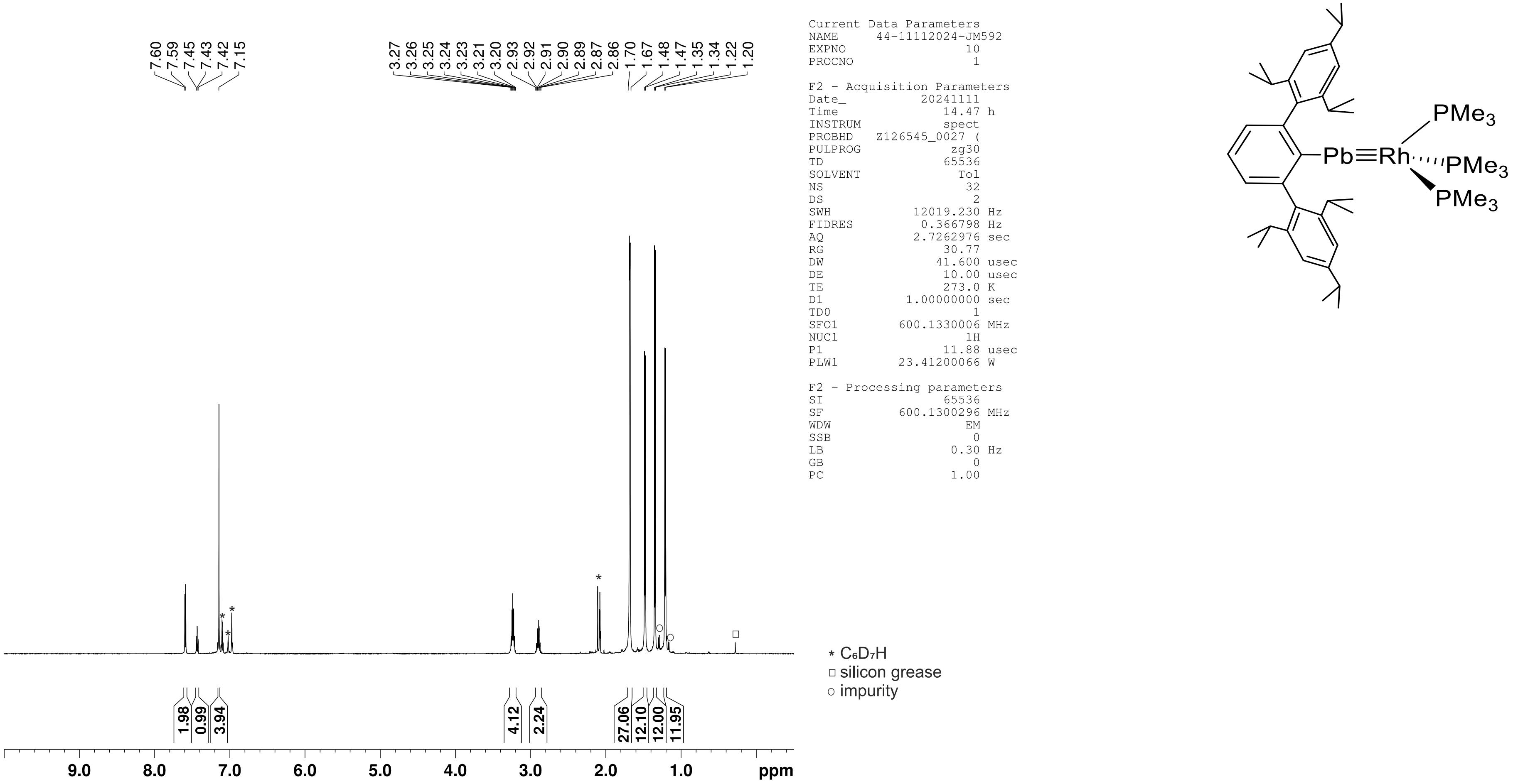

Figure SI31. ^{1}H NMR of compound **6**.

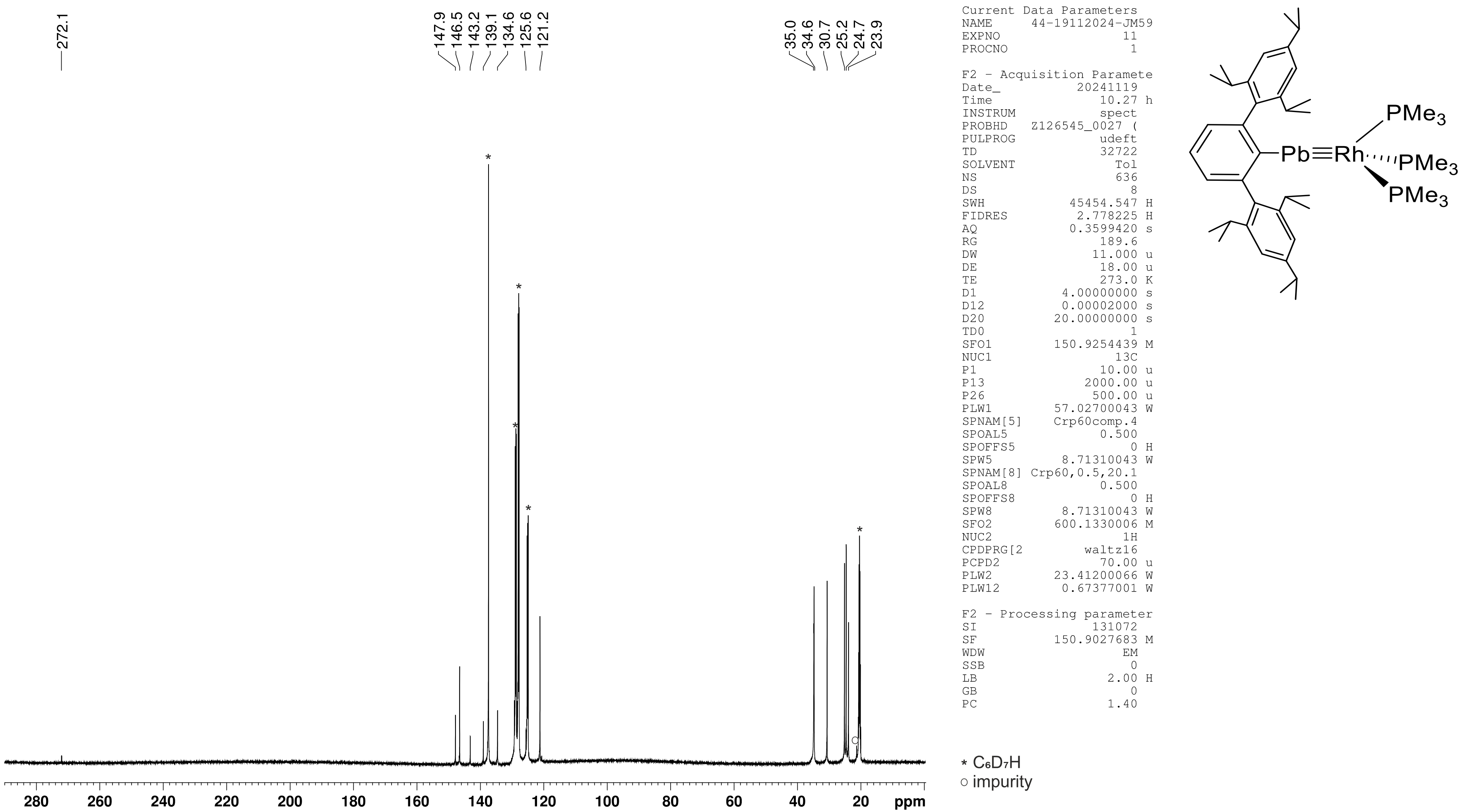

Figure SI32. $^{13}C\{^{1}H\}$ NMR of compound **6**.

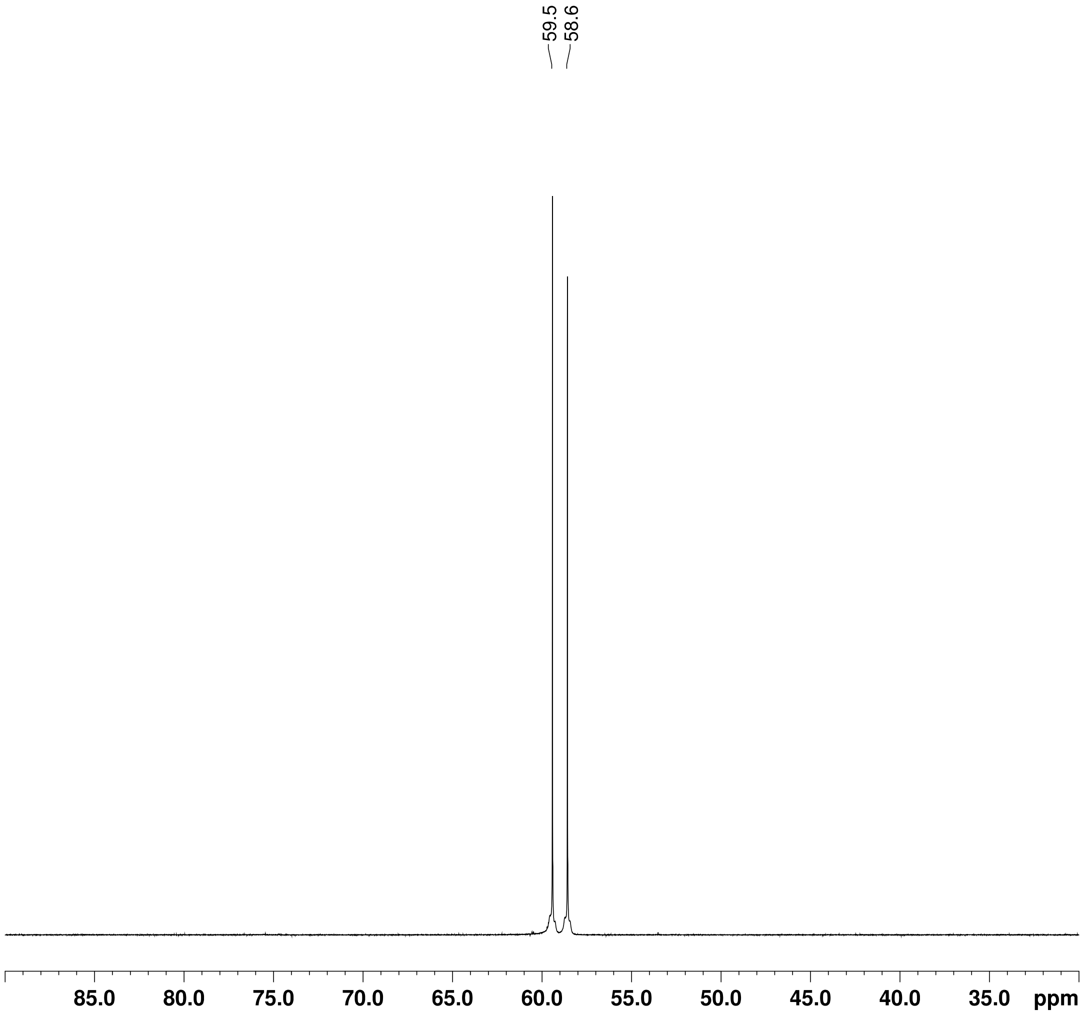


```
Current Data Parameters
NAME     44-19112024-JM595
EXPNO                   13
PROCNO                   1

F2 - Acquisition Parameters
Date_           20241119
Time               11.15 h
INSTRUM            spect
PROBHD   Z126545_0027 (
PULPROG          zgpg30
TD                65536
SOLVENT             Tol
NS                  257
DS                    4
SWH          49019.609 Hz
FIDRES        1.495960 Hz
AQ           0.6684672 sec
RG                189.6
DW               10.200 usec
DE                18.00 usec
TE                298.0 K
D1           2.00000000 sec
D11          0.03000000 sec
TD0                   1
SFO1        242.9443651 MHz
NUC1                31P
P1                12.25 usec
PLW1        52.43000031 W
SFO2        600.1324005 MHz
NUC2                 1H
CPDPRG[2        waltz16
PCPD2             70.00 usec
PLW2        23.41200066 W
PLW12        0.67377001 W
PLW13        0.33890000 W

F2 - Processing parameters
SI                32768
SF          242.9370770 MHz
WDW                  EM
SSB                   0
LB                 1.00 Hz
GB                    0
PC                 1.40
```

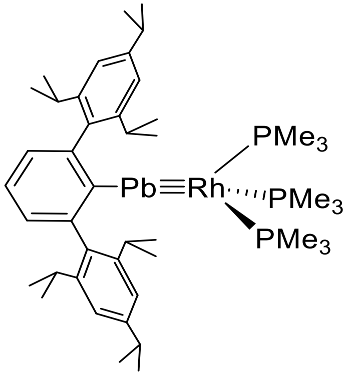

Figure SI33. $^{31}P\{^{1}H\}$ NMR of compound **6**.

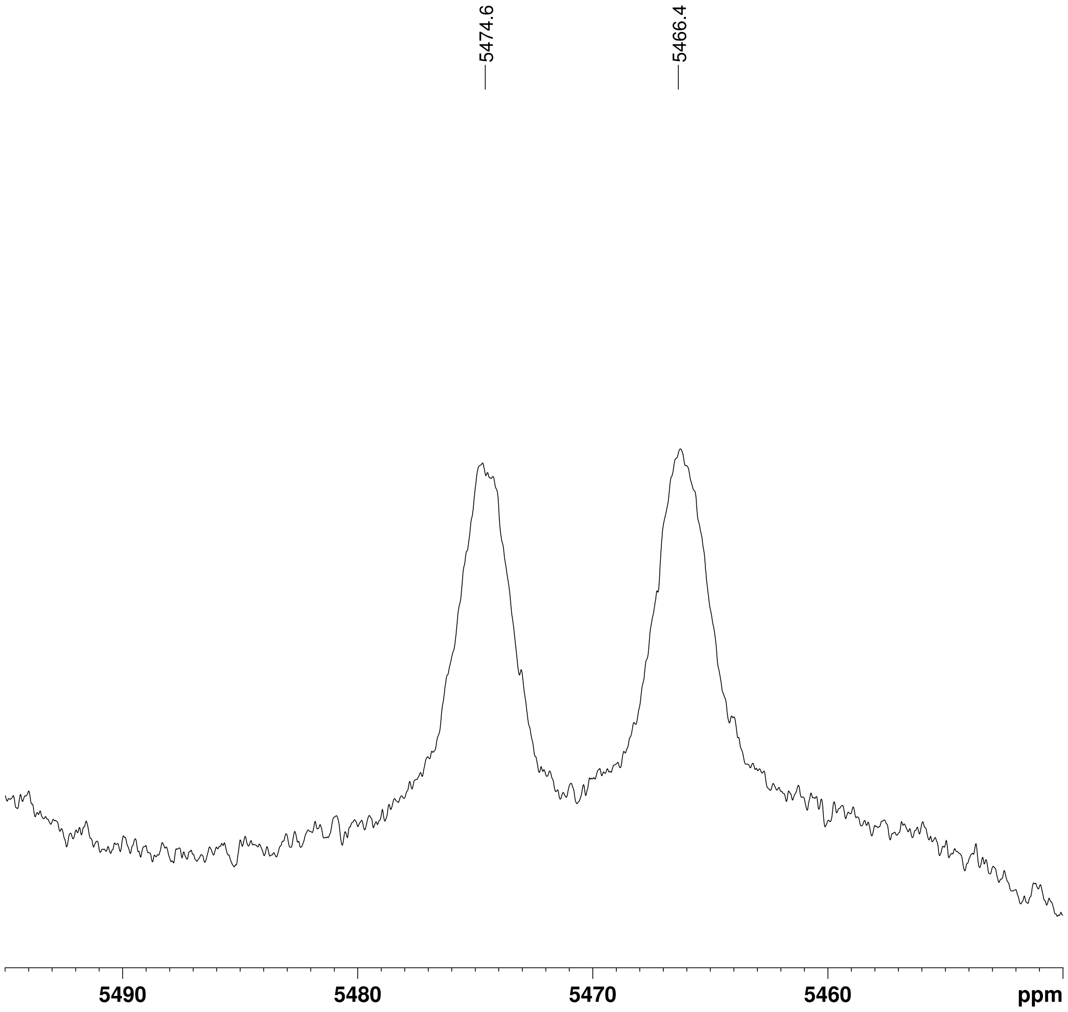


```
Current Data Parameters
NAME      44-19112024-JM595
EXPNO                    16
PROCNO                    1

F2 - Acquisition Parameters
Date_              20241119
Time                  12.27 h
INSTRUM               spect
PROBHD   Z126545_0027 (
PULPROG                zg30
TD                    24986
SOLVENT                 Tol
NS                    20067
DS                        0
SWH              125000.000 Hz
FIDRES            10.005603 Hz
AQ                0.0999440 sec
RG                    189.6
DW                    4.000 usec
DE                    18.00 usec
TE                    298.0 K
D1               0.01000000 sec
TD0                       1
SFO1            126.2664305 MHz
NUC1                  207Pb
P1                    15.13 usec
PLW1            60.00000000 W

F2 - Processing parameters
SI                    65536
SF              125.5507910 MHz
WDW                      EM
SSB                       0
LB                    15.00 Hz
GB                        0
PC                     1.00
```

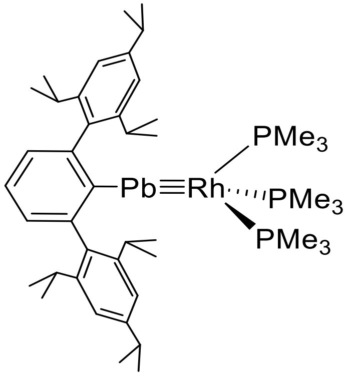

Figure SI34. ^{207}Pb{^{1}H} NMR of compound **6**.

NMR spectra of Ar*Pb≡Ir(PEt_3)$_3$ (**7**)

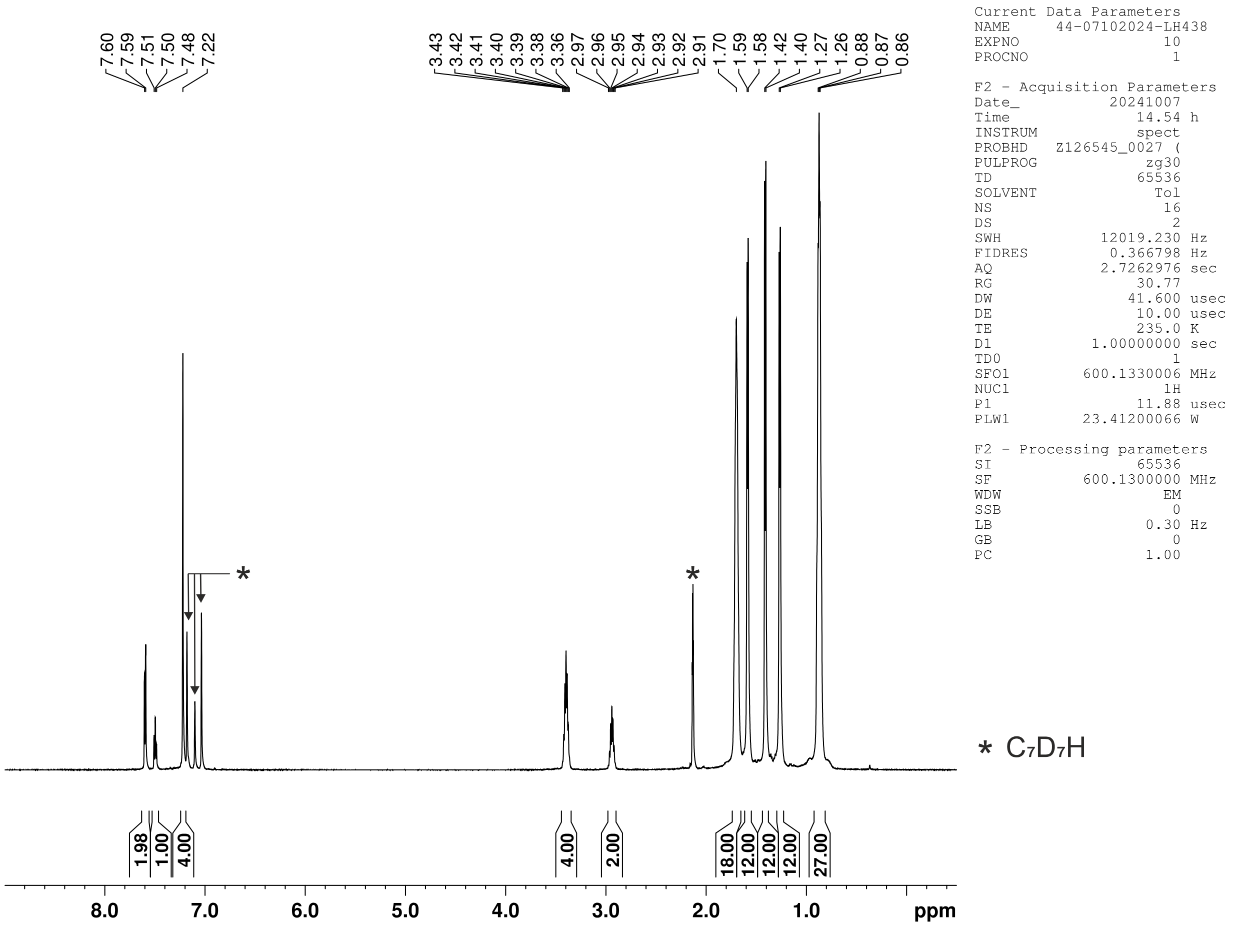

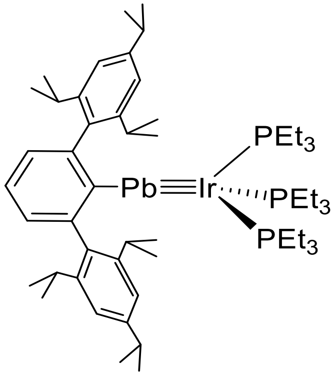

Figure SI35. ^{1}H NMR of compound **7**.

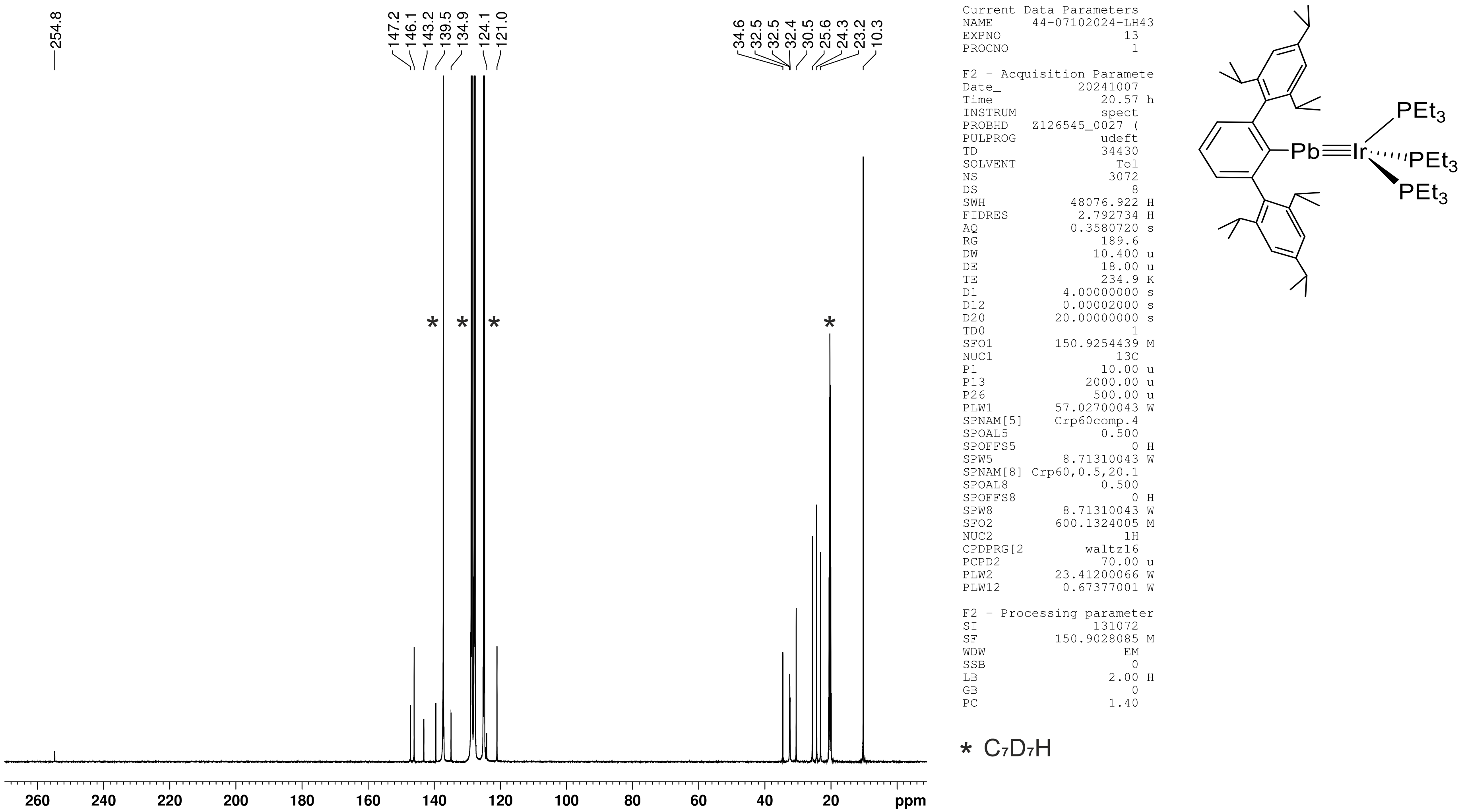

Figure SI36. $^{13}C\{^{1}H\}$ NMR of compound **7**.

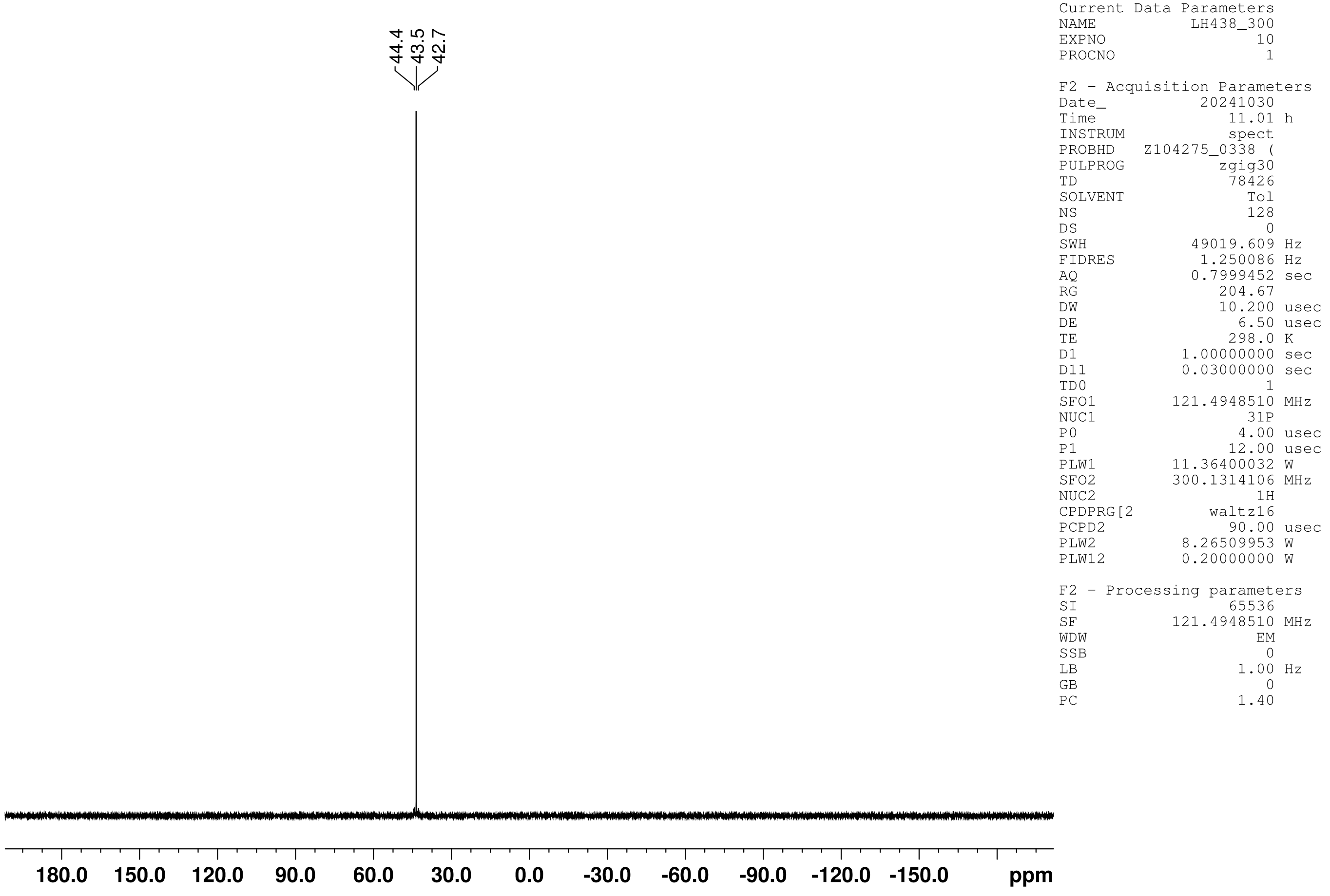

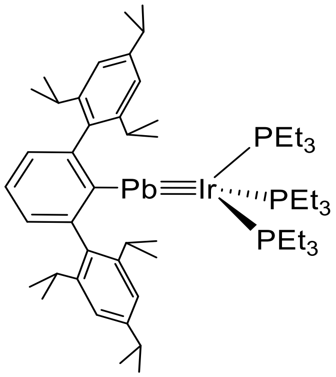

Figure SI37. $^{31}P\{^{1}H\}$ NMR of compound **7**.

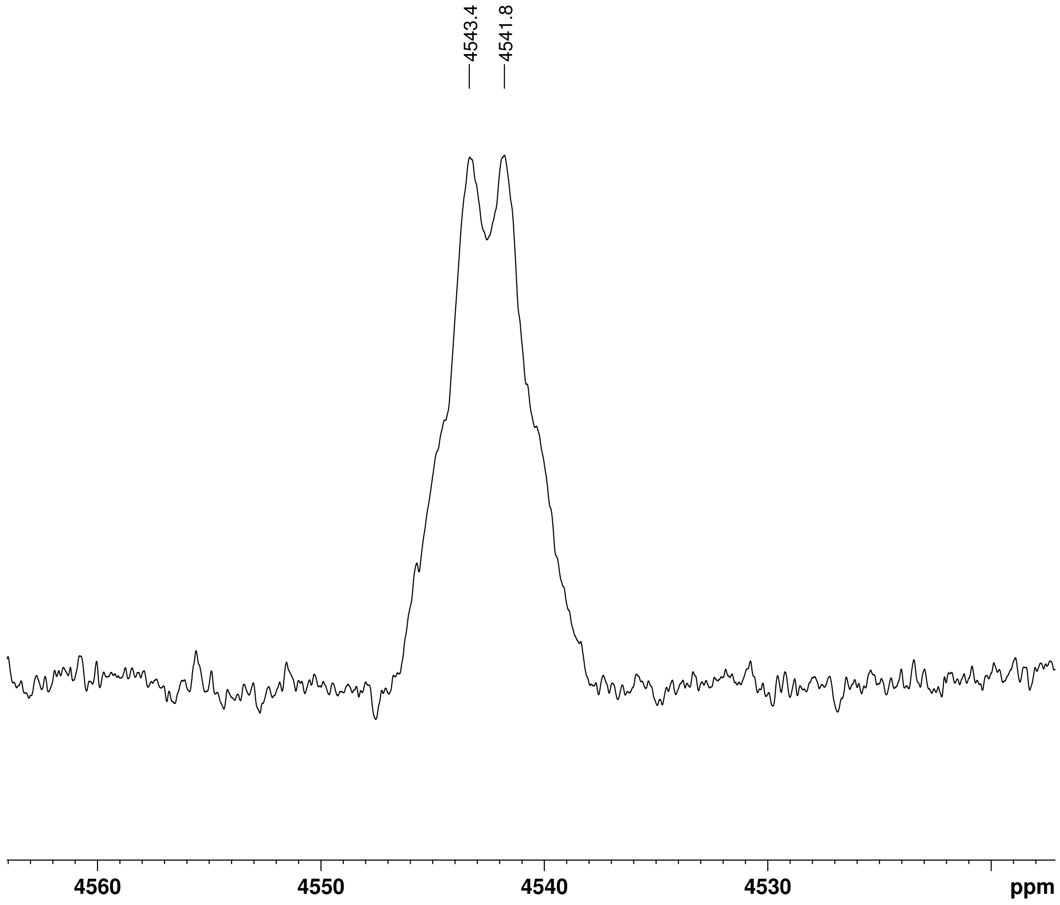


```
Current Data Parameters
NAME      44-31102024-LH438
EXPNO                100013
PROCNO                    1

F2 - Acquisition Parameters
Date_              20241031
Time                  15.02 h
INSTRUM               spect
PROBHD   Z126545_0027 (
PULPROG                zg30
TD                     5678
SOLVENT                 Tol
NS                    32768
DS                        0
SWH               28409.092 Hz
FIDRES            10.006724 Hz
AQ                0.0999328 sec
RG                    189.6
DW                   17.600 usec
DE                    50.00 usec
TE                    298.0 K
D1               0.10000000 sec
TD0                       1
SFO1            126.1169102 MHz
NUC1                  207Pb
P1                    15.13 usec
PLW1            60.00000000 W

F2 - Processing parameters
SI                    65536
SF              125.5507910 MHz
WDW                      EM
SSB                       0
LB                    20.00 Hz
GB                        0
PC                     1.40
```

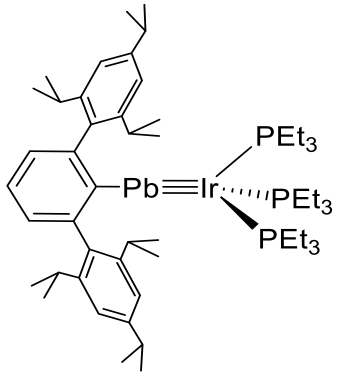

Figure SI38. $^{207}Pb\{^{1}H\}$ NMR of compound **7**.

NMR spectra of [Rh(PMe$_3$)$_4$][Ar*GeCl$_2$] (**8**)

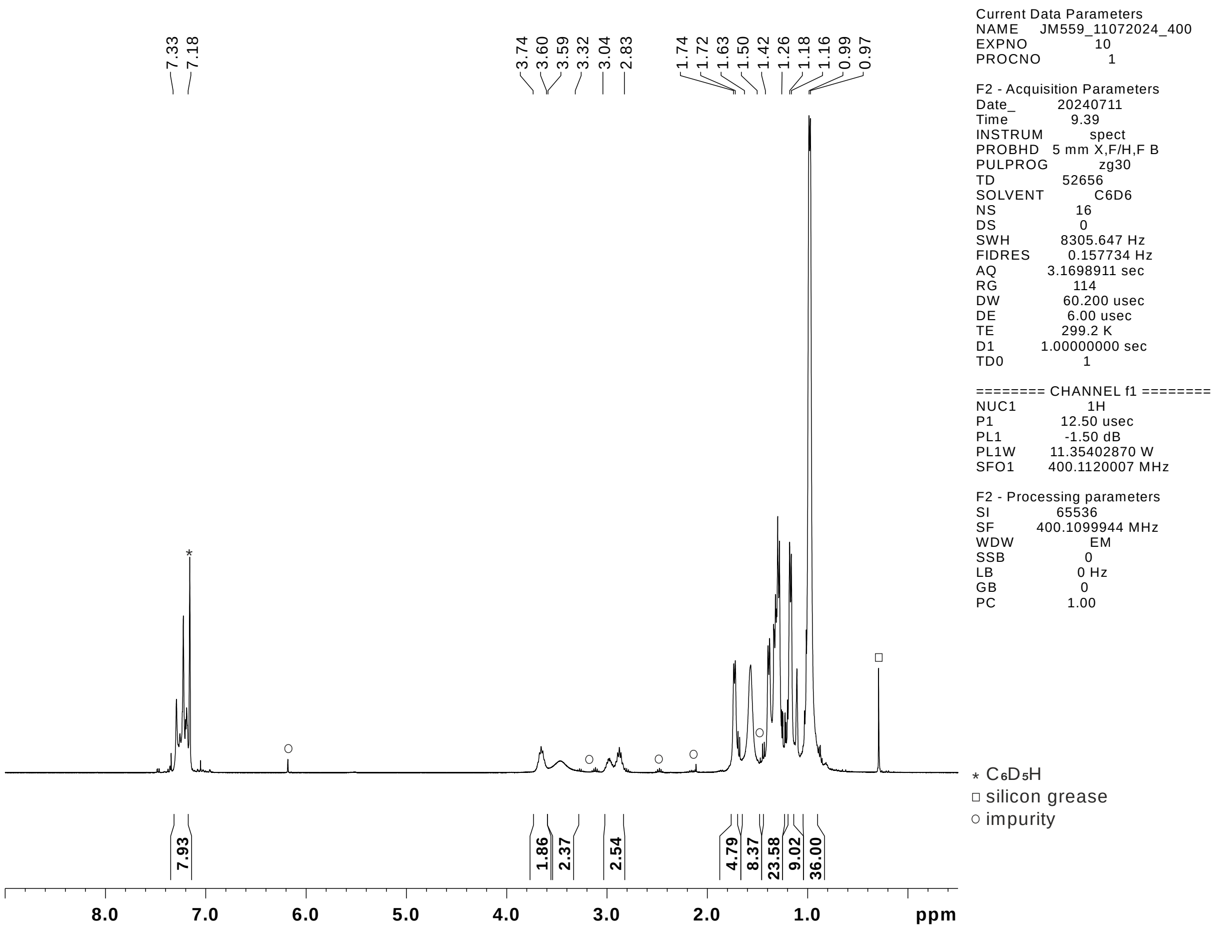

Figure SI39. ^{1}H NMR of compound **8**.

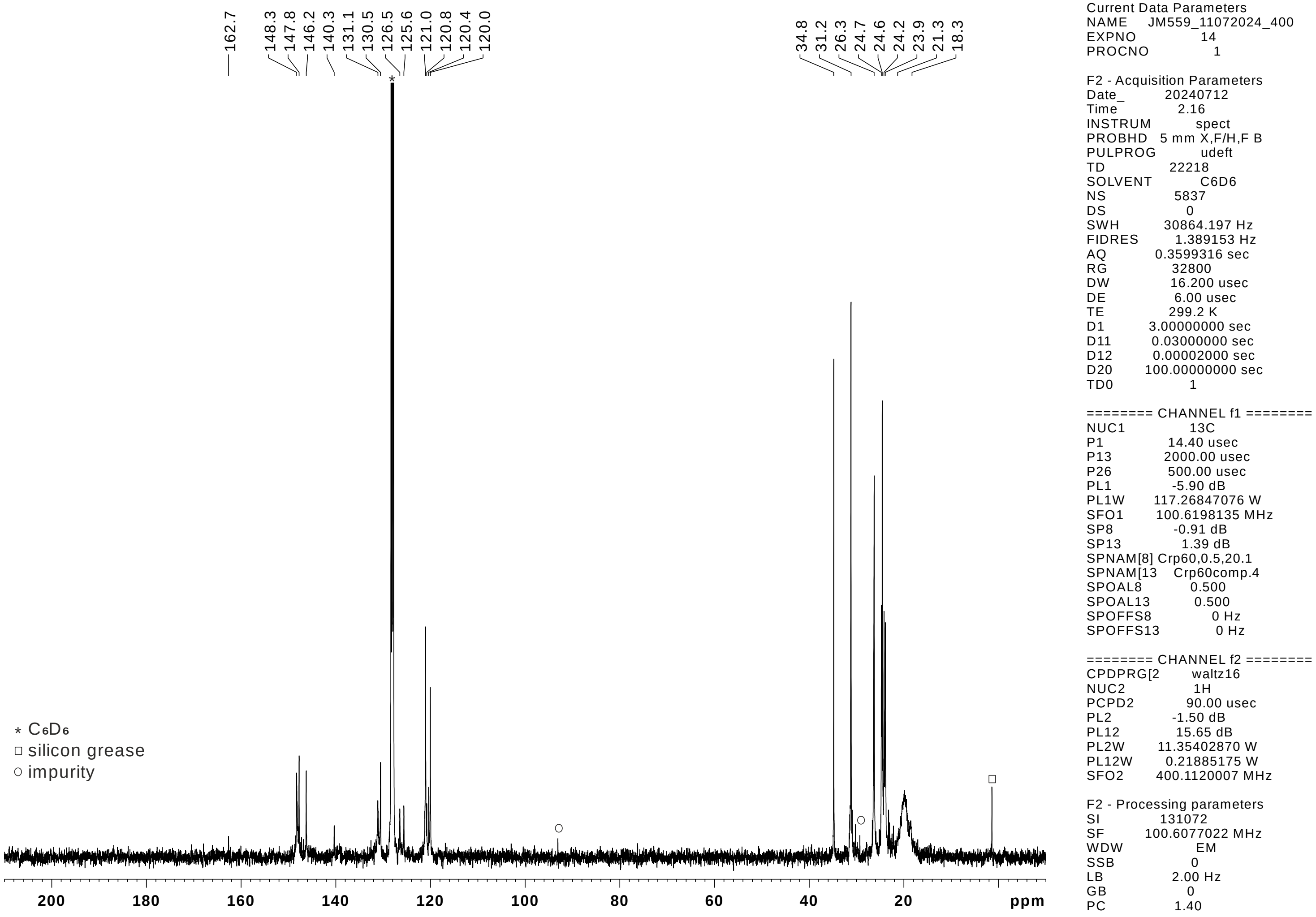

Figure SI40. $^{13}C\{^{1}H\}$ NMR of compound **8**.

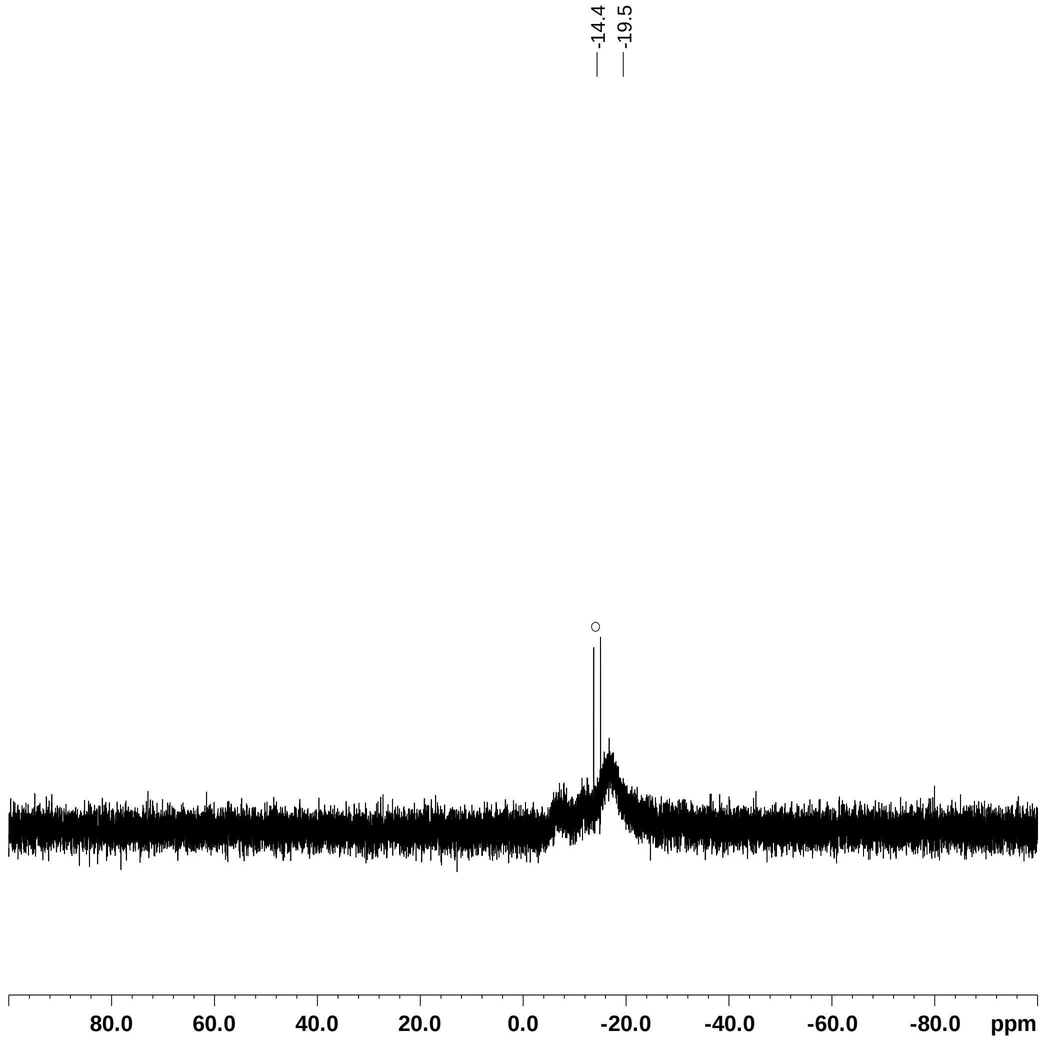

Current Data Parameters
NAME JM559_11072024_400
EXPNO 11
PROCNO 1

F2 - Acquisition Parameters
Date_ 20240711
Time 9.43
INSTRUM spect
PROBHD 5 mm X,F/H,F B
PULPROG zgig30b
TD 88150
SOLVENT C6D6
NS 128
DS 0
SWH 65789.477 Hz
FIDRES 0.746336 Hz
AQ 0.6699400 sec
RG 23100
DW 7.600 usec
DE 6.00 usec
TE 299.2 K
D1 1.00000000 sec
D11 0.03000000 sec
TD0 1

======== CHANNEL f1 ========
NUC1 31P
P1 14.50 usec
PL1 -4.00 dB
PL1W 56.78615952 W
SFO1 161.9674970 MHz

======== CHANNEL f2 ========
CPDPRG[2 waltz16
NUC2 1H
PCPD2 90.00 usec
PL2 -1.50 dB
PL12 15.65 dB
PL2W 11.35402870 W
PL12W 0.21885175 W
SFO2 400.1120007 MHz

F2 - Processing parameters
SI 131072
SF 161.9674970 MHz
WDW EM
SSB 0
LB 1.00 Hz
GB 0
PC 1.40

○ impurity

Figure SI41. $^{31}P\{^{1}H\}$ NMR of compound **8**.

NMR spectra of $[Rh(PMe_3)_4][Ar^*SnCl_2]$ (**9**)

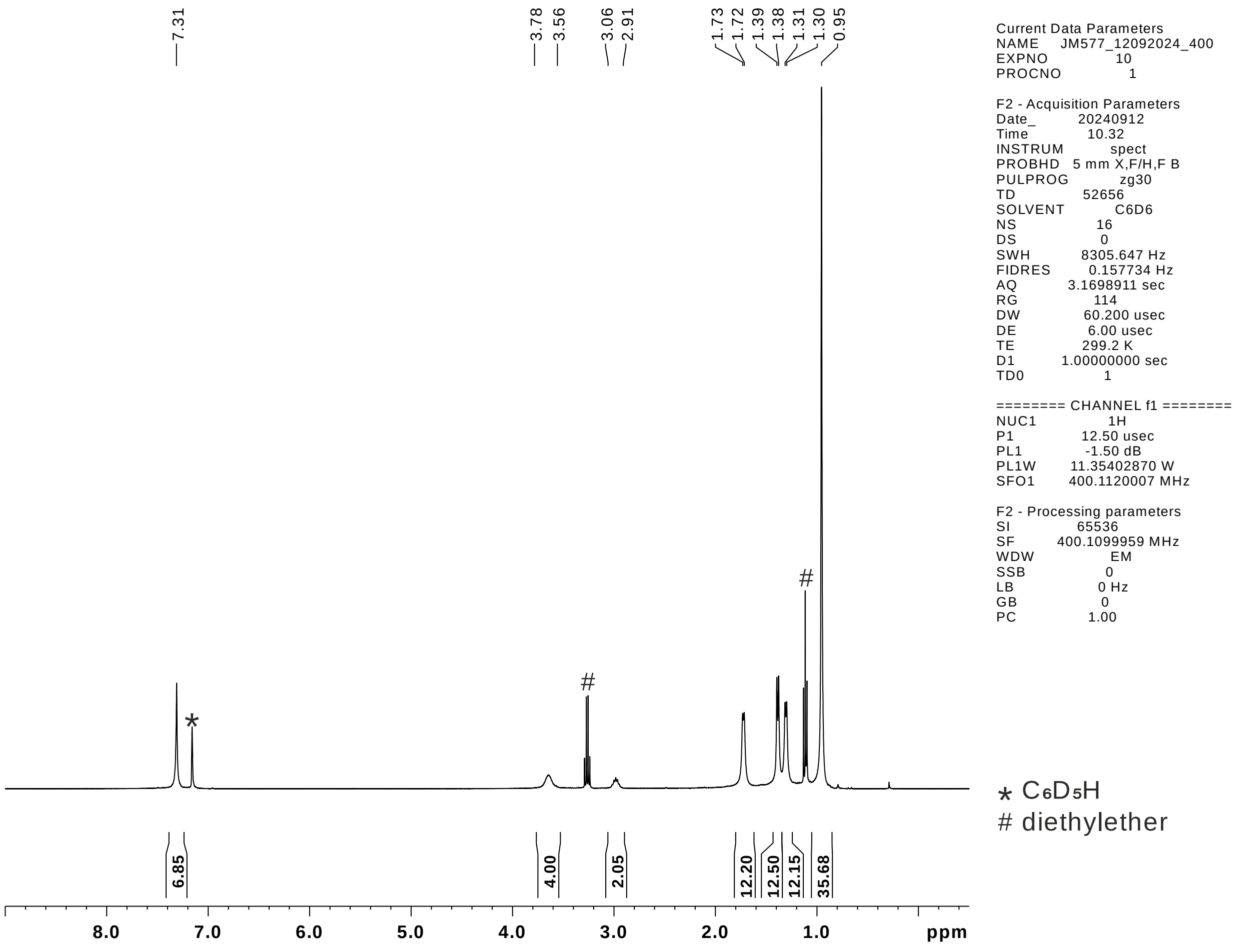

Figure SI42. ^{1}H NMR of compound **9**.

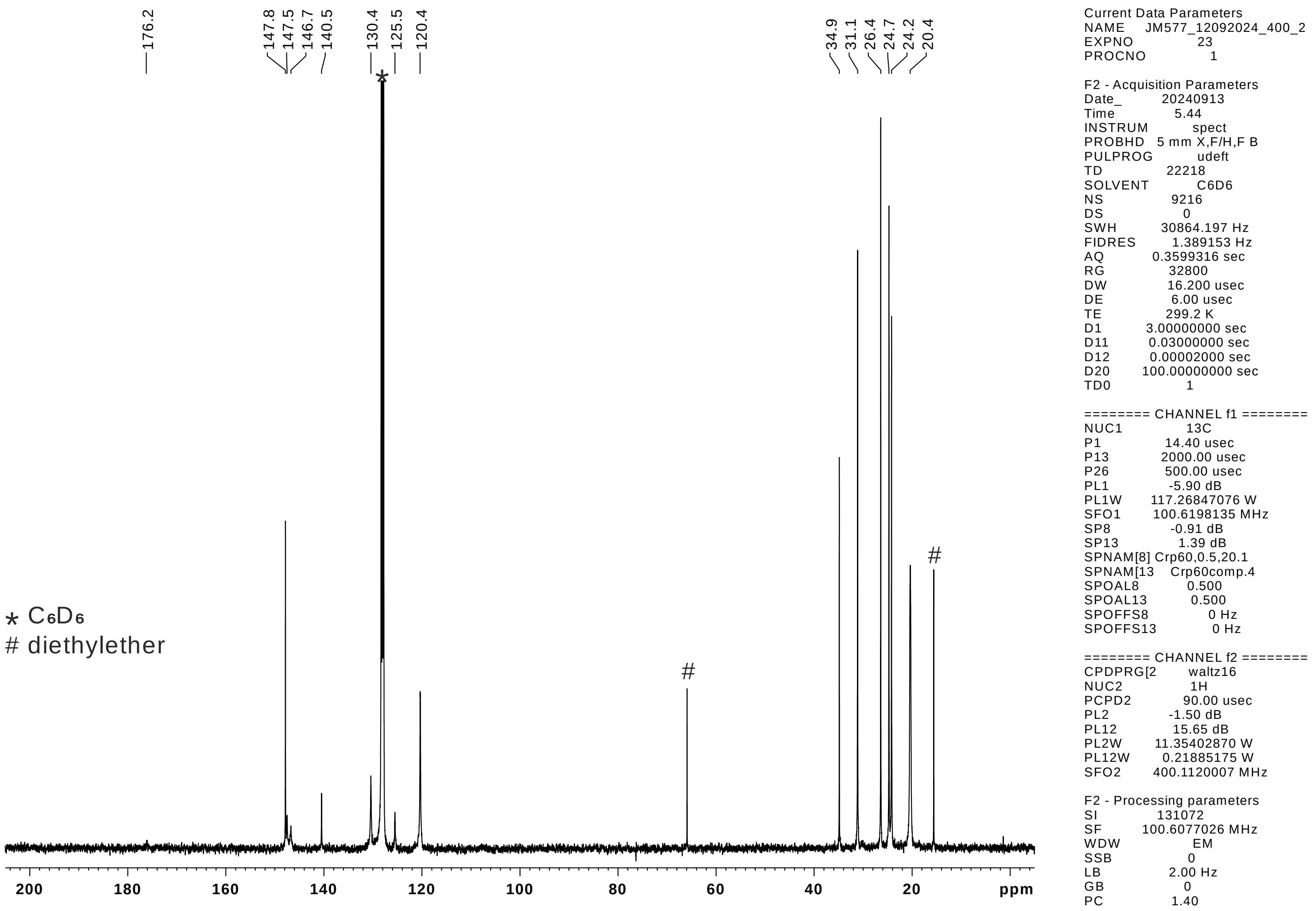

Figure SI43. $^{13}C\{^1H\}$ NMR of compound **9**.

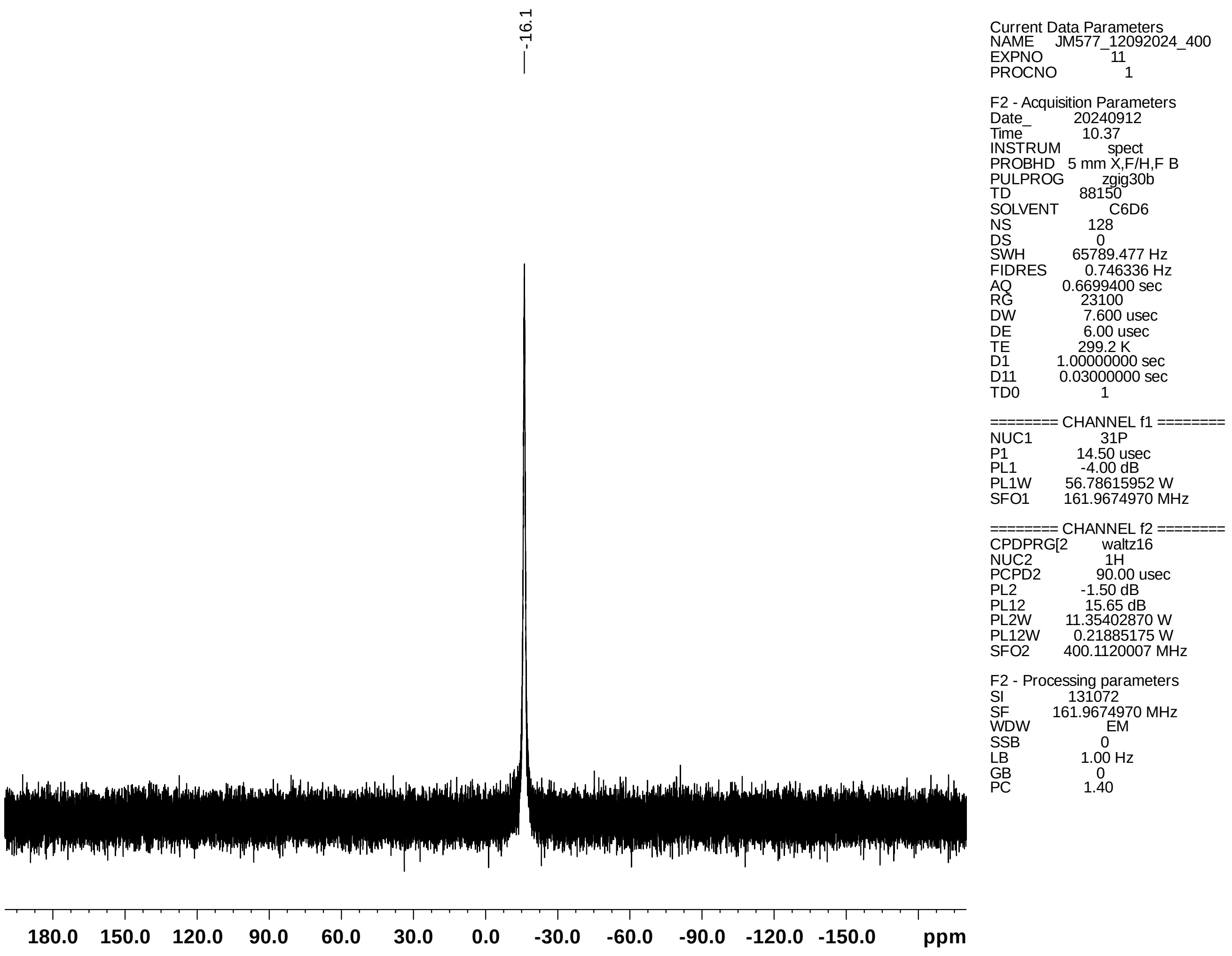

Figure SI44. $^{31}P\{^{1}H\}$ NMR of compound **9**.

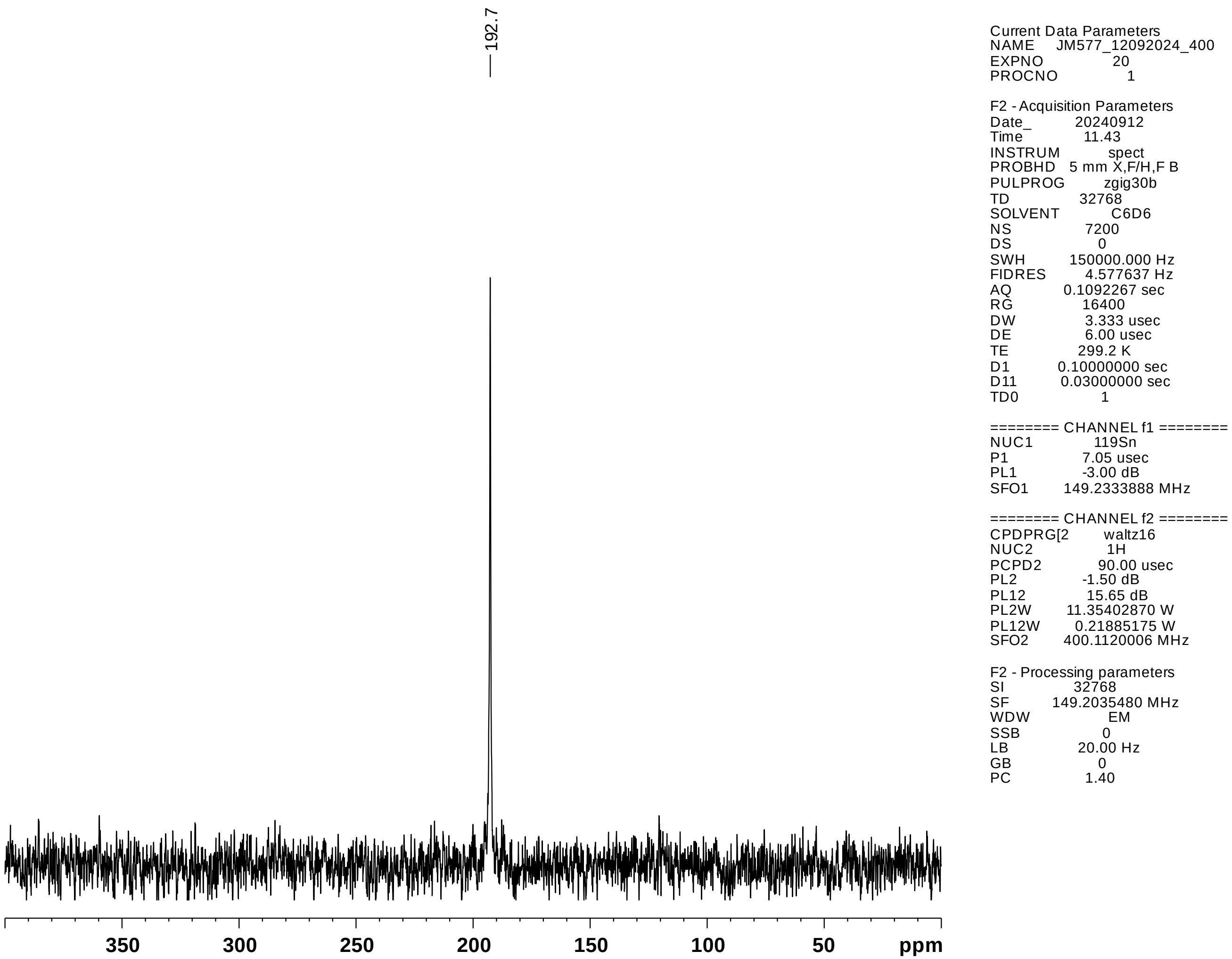

Figure SI45. $^{119}Sn\{^{1}H\}$ NMR of compound **9**.

NMR spectra of $[Ir(PMe_3)_4][Ar^*SnCl_2]$ (**10**)

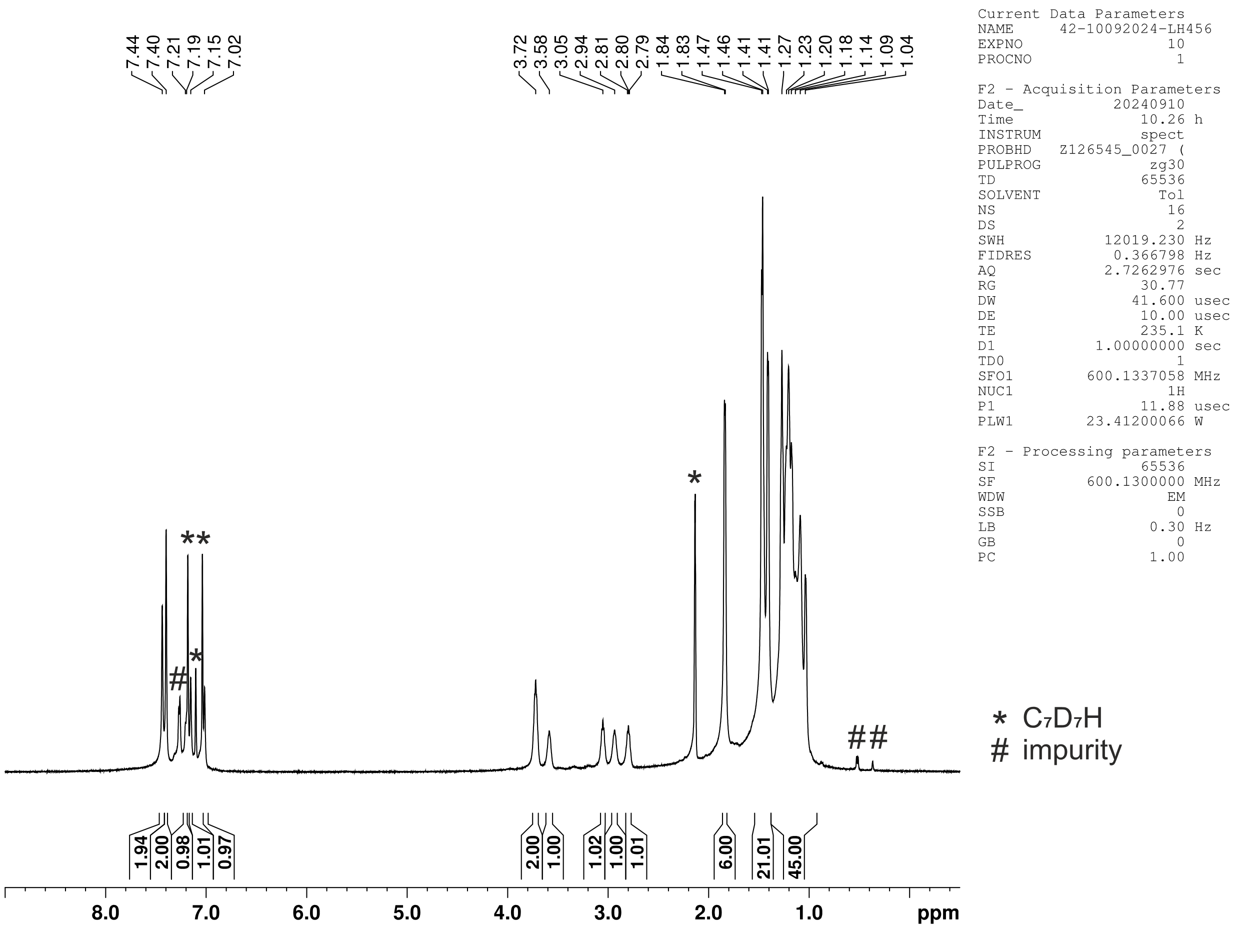

Figure SI46. ^{1}H NMR of compound **10**.

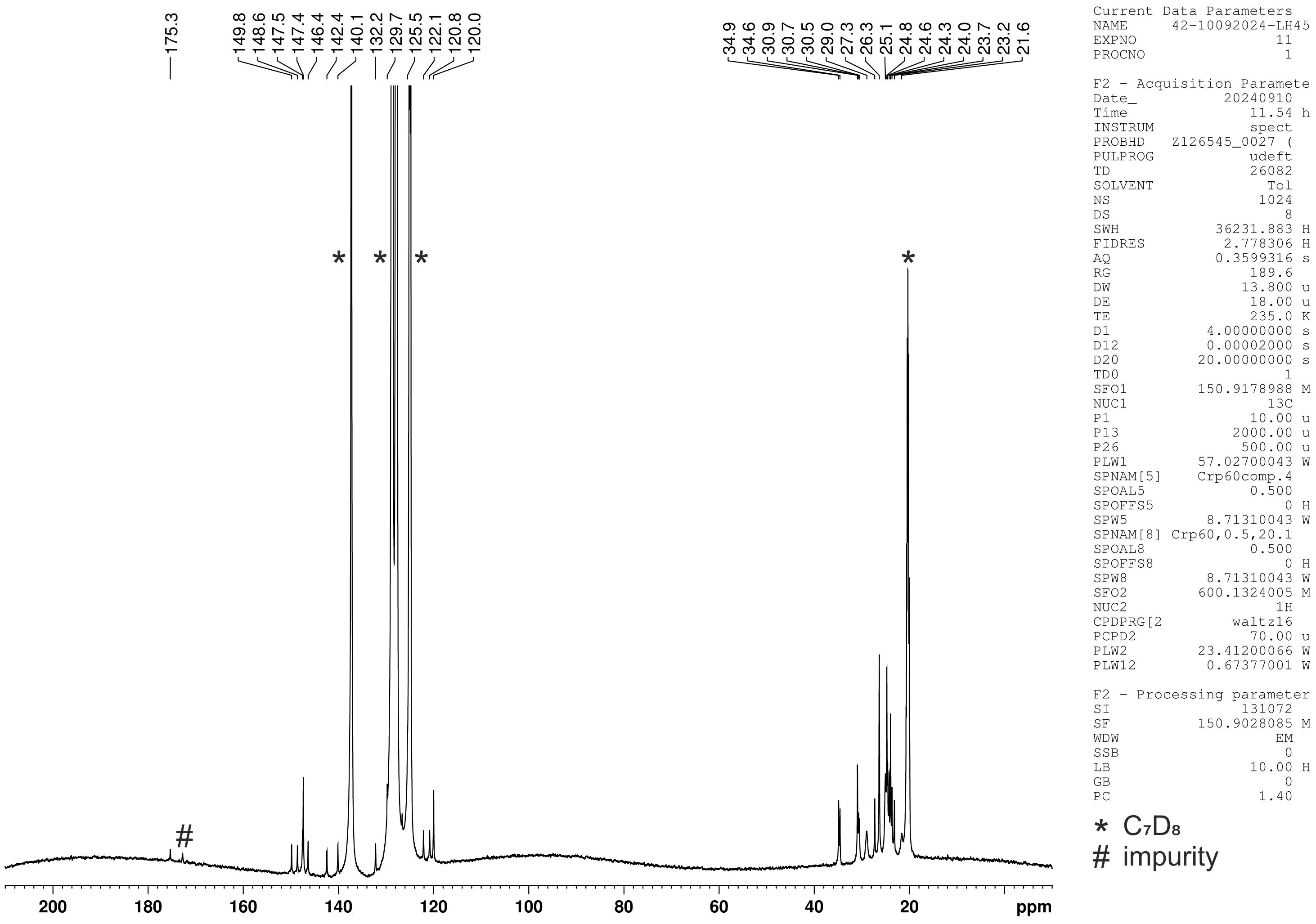

Figure SI47. $^{13}C\{^{1}H\}$ NMR of compound **10**.

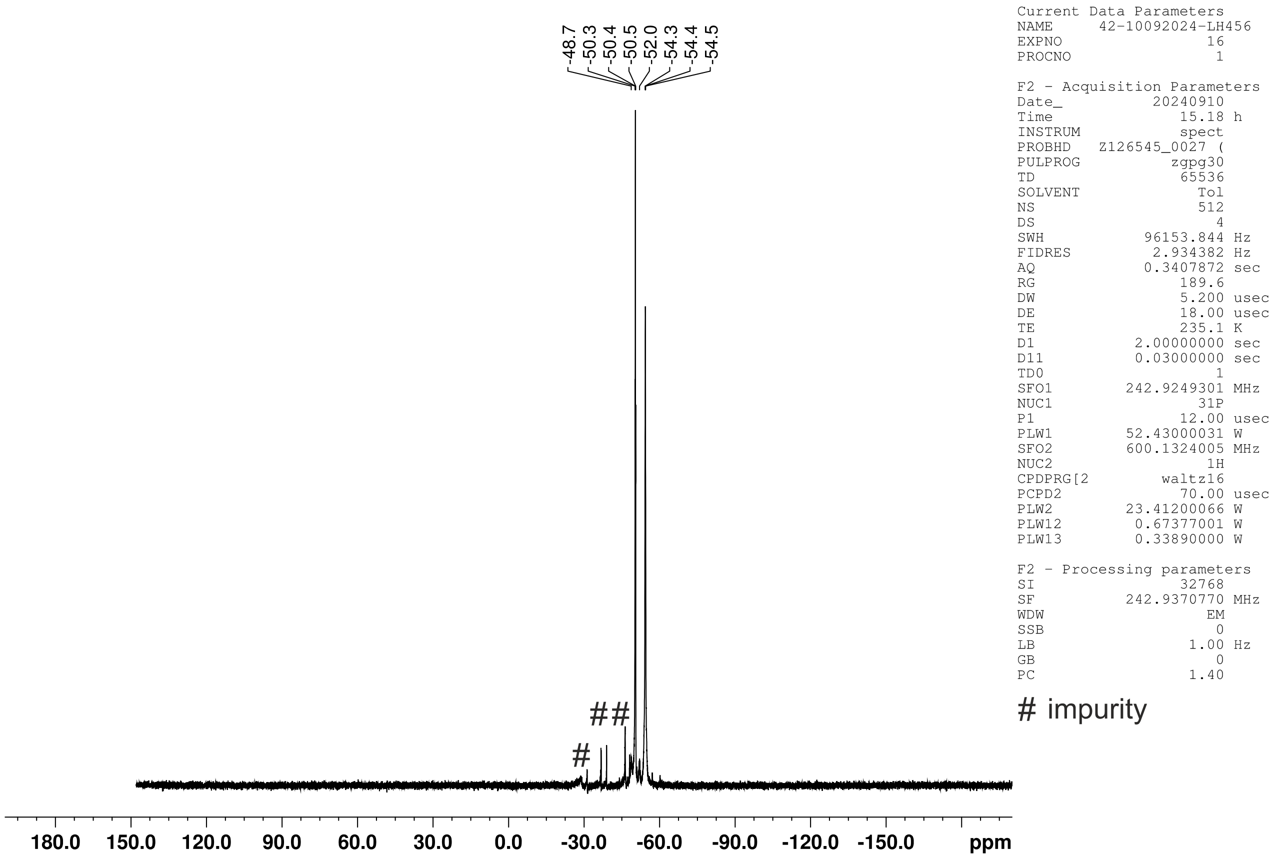

Figure SI48. $^{31}P\{^{1}H\}$ NMR of compound **10**.

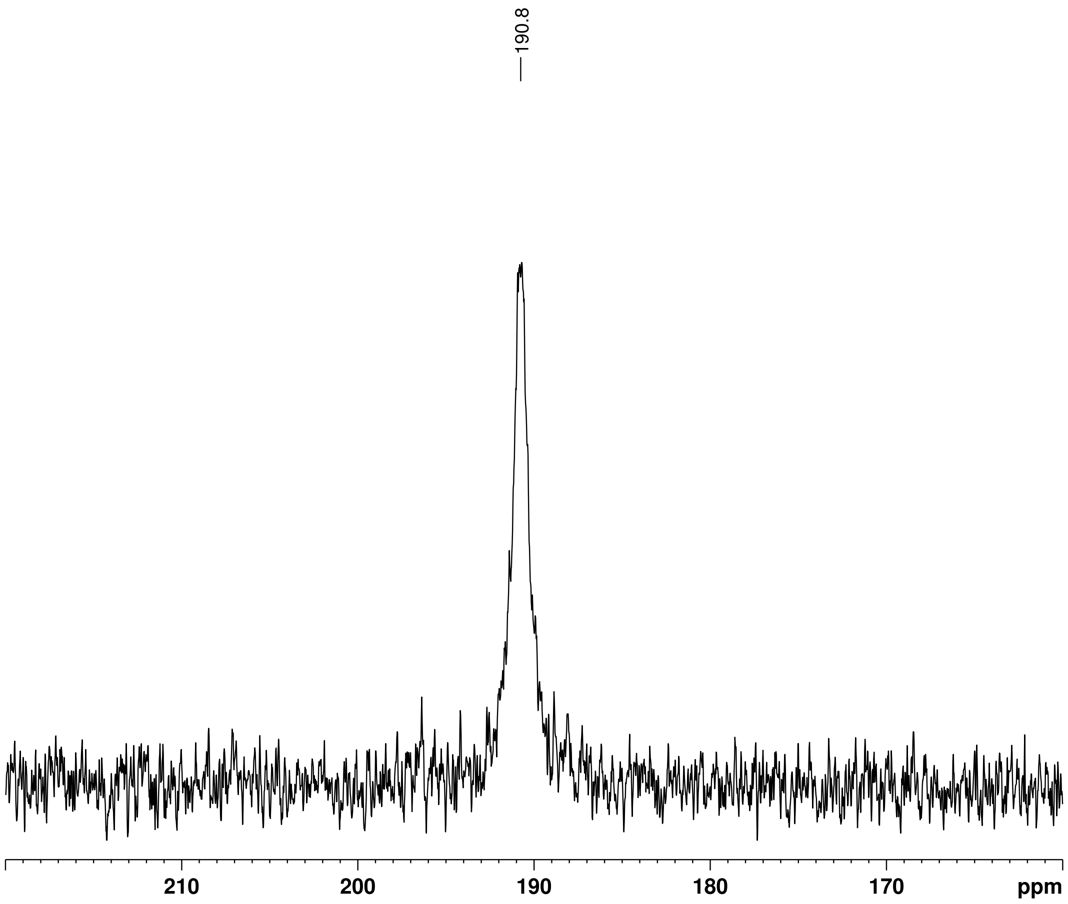


```
Current Data Parameters
NAME     42-10092024-LH456
EXPNO                  18
PROCNO                  1

F2 - Acquisition Parameters
Date_          20240910
Time              17.02 h
INSTRUM           spect
PROBHD   Z126545_0027 (
PULPROG          zgig30
TD                50984
SOLVENT             Tol
NS                 4096
DS                    4
SWH          150000.000 Hz
FIDRES         5.884199 Hz
AQ            0.1699467 sec
RG                189.6
DW                3.333 usec
DE                18.00 usec
TE                235.0 K
D1           1.00000000 sec
D11          0.03000000 sec
TD0                   1
SFO1        223.7922698 MHz
NUC1              119Sn
P1                14.23 usec
PLW1        50.00000000 W
SFO2        600.1324005 MHz
NUC2                 1H
CPDPRG[2        waltz16
PCPD2             70.00 usec
PLW2        23.41200066 W
PLW12        0.67377001 W

F2 - Processing parameters
SI                32768
SF          223.7922698 MHz
WDW                  EM
SSB                   0
LB                10.00 Hz
GB                    0
PC                 1.40
```

Figure SI49. $^{119}Sn\{^{1}H\}$ NMR of compound **10**.

NMR spectra of [Ar*Ge(CO_3)Rh(CO)(PMe_3)$_3$] (**11**)

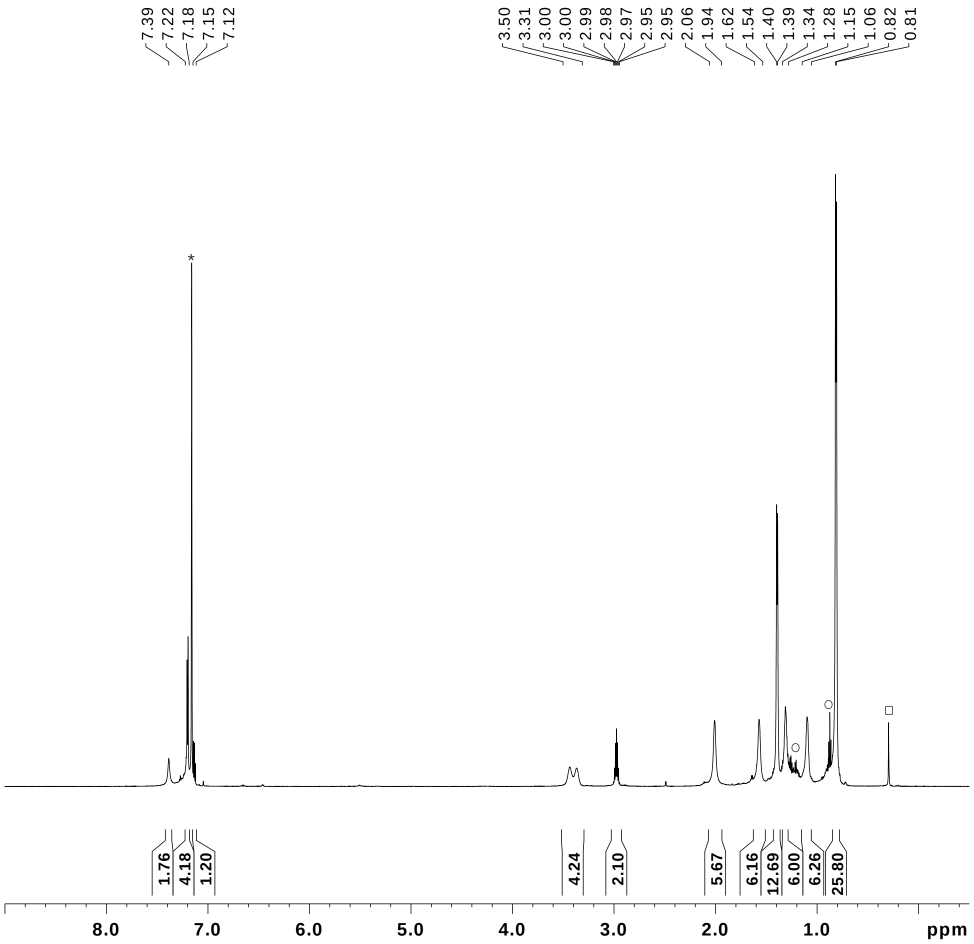

Current Data Parameters
NAME 1-25032024-JM453
EXPNO 10
PROCNO 1

F2 - Acquisition Parameters
Date_ 20240325
Time 15.57 h
INSTRUM spect
PROBHD Z135421_0007 (
PULPROG zg30
TD 65536
SOLVENT C6D6
NS 32
DS 0
SWH 14097.744 Hz
FIDRES 0.430229 Hz
AQ 2.3243434 sec
RG 21.99
DW 35.467 usec
DE 10.00 usec
TE 298.0 K
D1 1.00000000 sec
TD0 1
SFO1 700.2102012 MHz
NUC1 1H
P1 8.00 usec
PLW1 14.67599964 W

F2 - Processing parameters
SI 65536
SF 700.2059943 MHz
WDW EM
SSB 0
LB 0.30 Hz
GB 0
PC 1.00

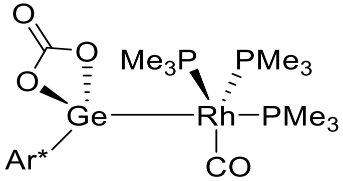

* C_6D_5H
□ silicon grease
○ impurity

Figure SI50. ^{1}H NMR of compound **11**.

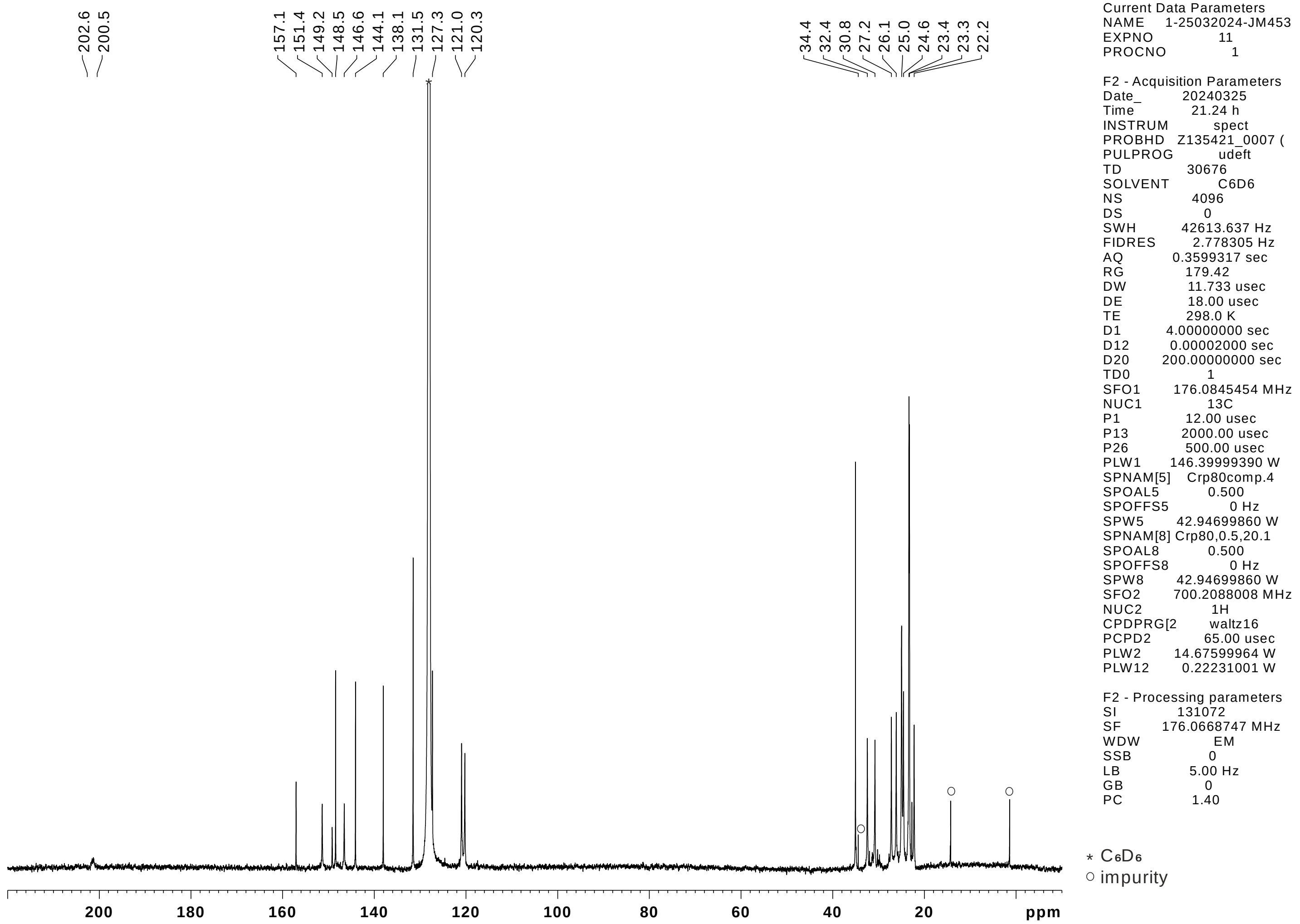

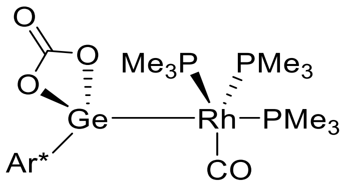

Figure SI51. $^{13}C\{^{1}H\}$ NMR of compound **11**.

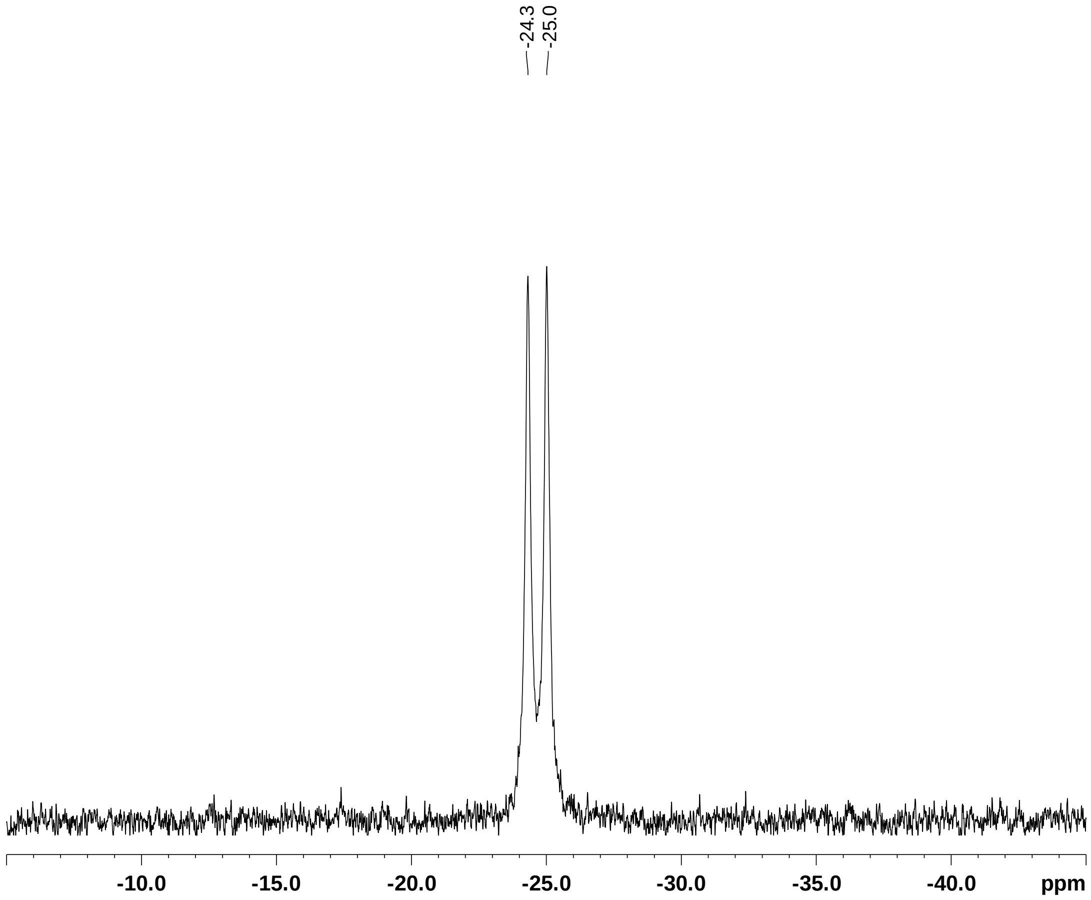

Current Data Parameters
NAME JM453_01032024_400
EXPNO 11
PROCNO 1

F2 - Acquisition Parameters
Date_ 20240301
Time 12.36
INSTRUM spect
PROBHD 5 mm X,F/H,F B
PULPROG zgig30b
TD 88150
SOLVENT C6D6
NS 128
DS 0
SWH 65789.477 Hz
FIDRES 0.746336 Hz
AQ 0.6699400 sec
RG 23100
DW 7.600 usec
DE 6.00 usec
TE 299.2 K
D1 1.00000000 sec
D11 0.03000000 sec
TD0 1

======== CHANNEL f1 ========
NUC1 31P
P1 14.50 usec
PL1 -4.00 dB
PL1W 56.78615952 W
SFO1 161.9674970 MHz

======== CHANNEL f2 ========
CPDPRG[2 waltz16
NUC2 1H
PCPD2 90.00 usec
PL2 -1.50 dB
PL12 15.65 dB
PL2W 11.35402870 W
PL12W 0.21885175 W
SFO2 400.1120007 MHz

F2 - Processing parameters
SI 131072
SF 161.9674970 MHz
WDW EM
SSB 0
LB 3.00 Hz
GB 0
PC 1.40

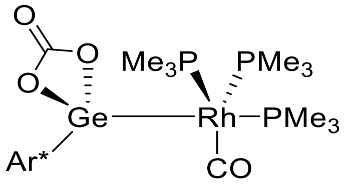

Figure SI52. $^{31}P\{^{1}H\}$ NMR of compound **11**.

NMR spectra of [Ar*Ge(CO_3)Ir(CO)(PMe_3)$_3$] (**12**)

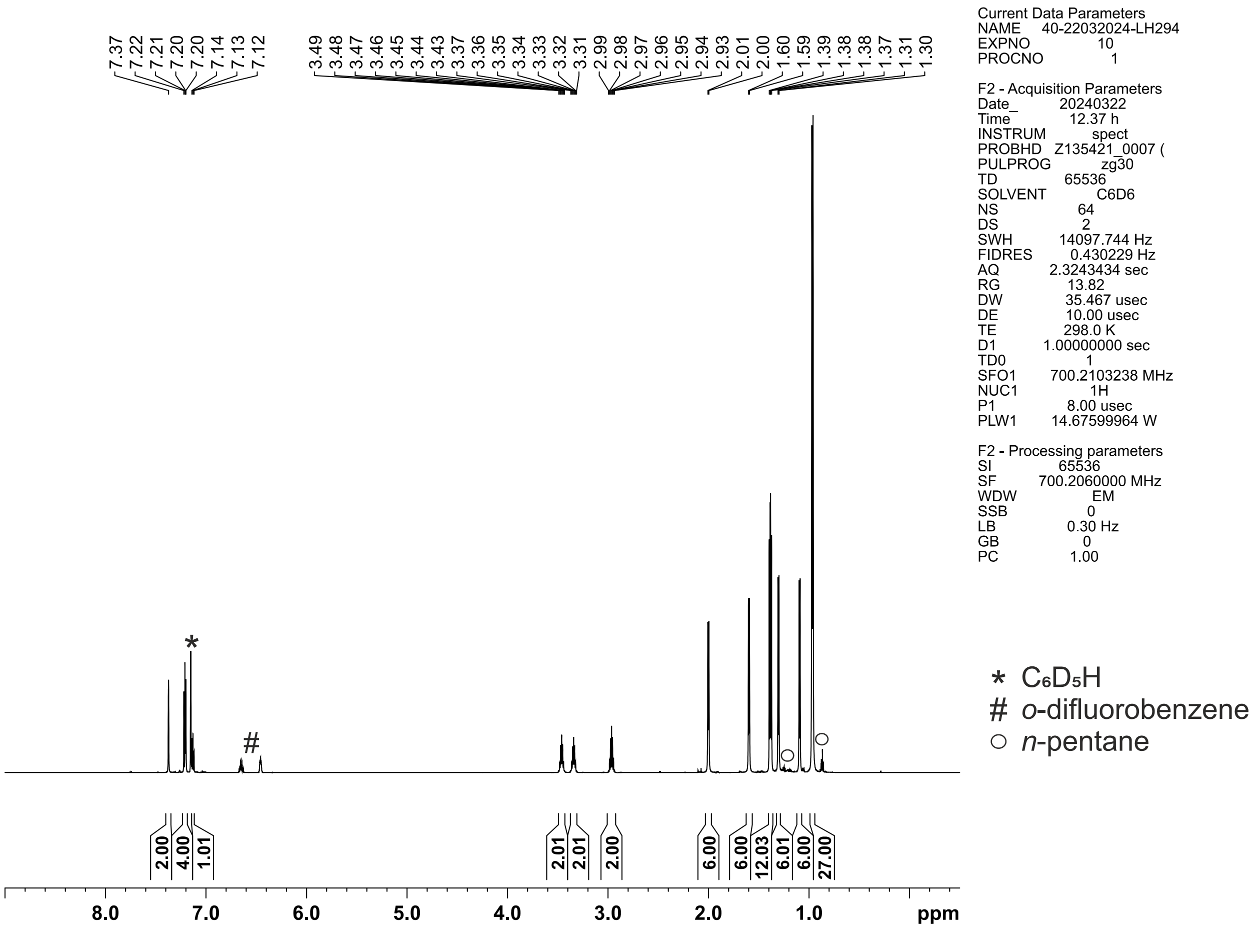

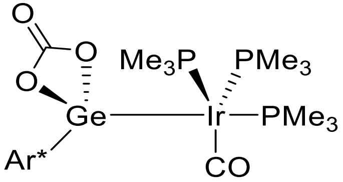

Figure SI53. ^{1}H NMR of compound **12**.

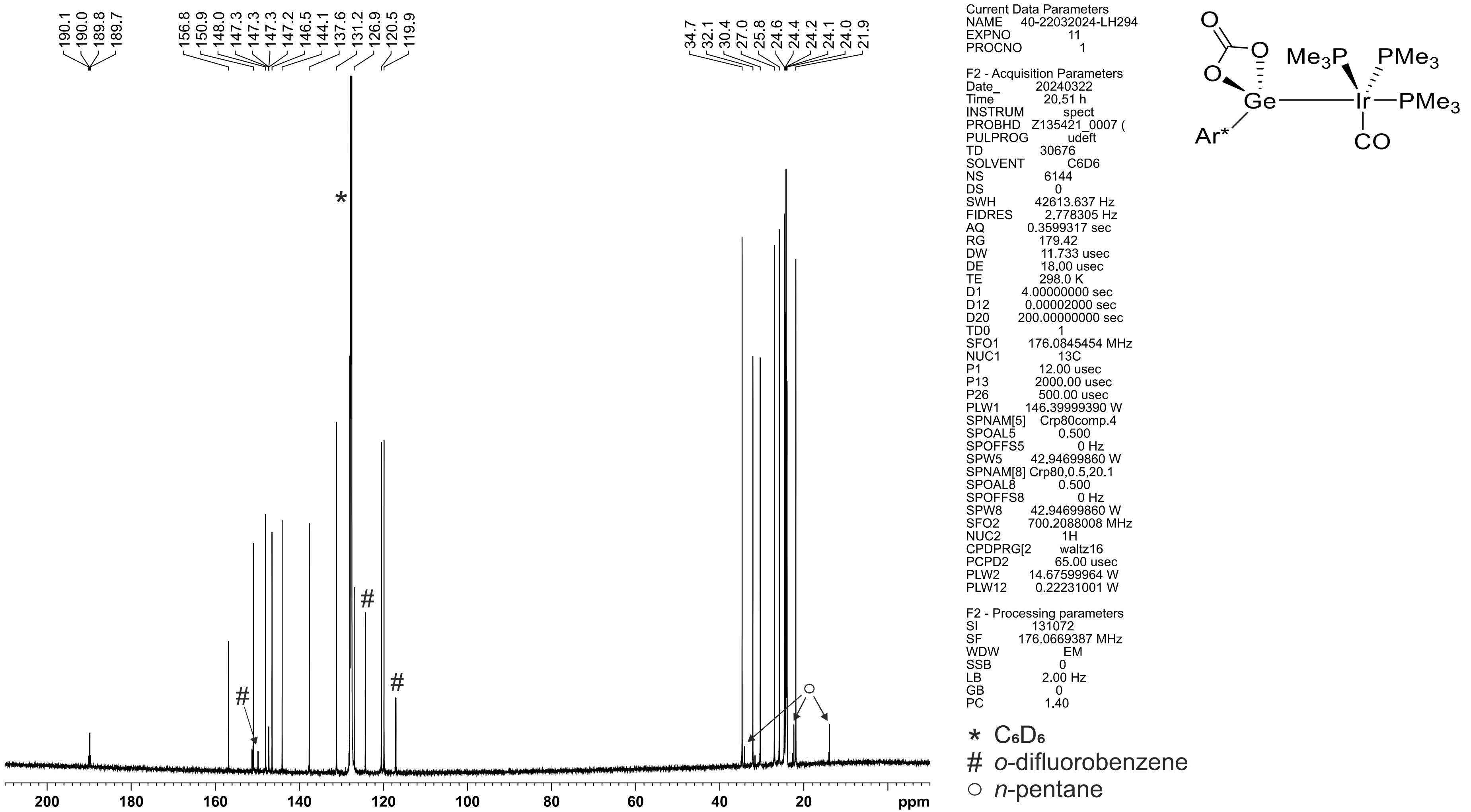

Figure SI54. $^{13}C\{^{1}H\}$ NMR of compound **12**.

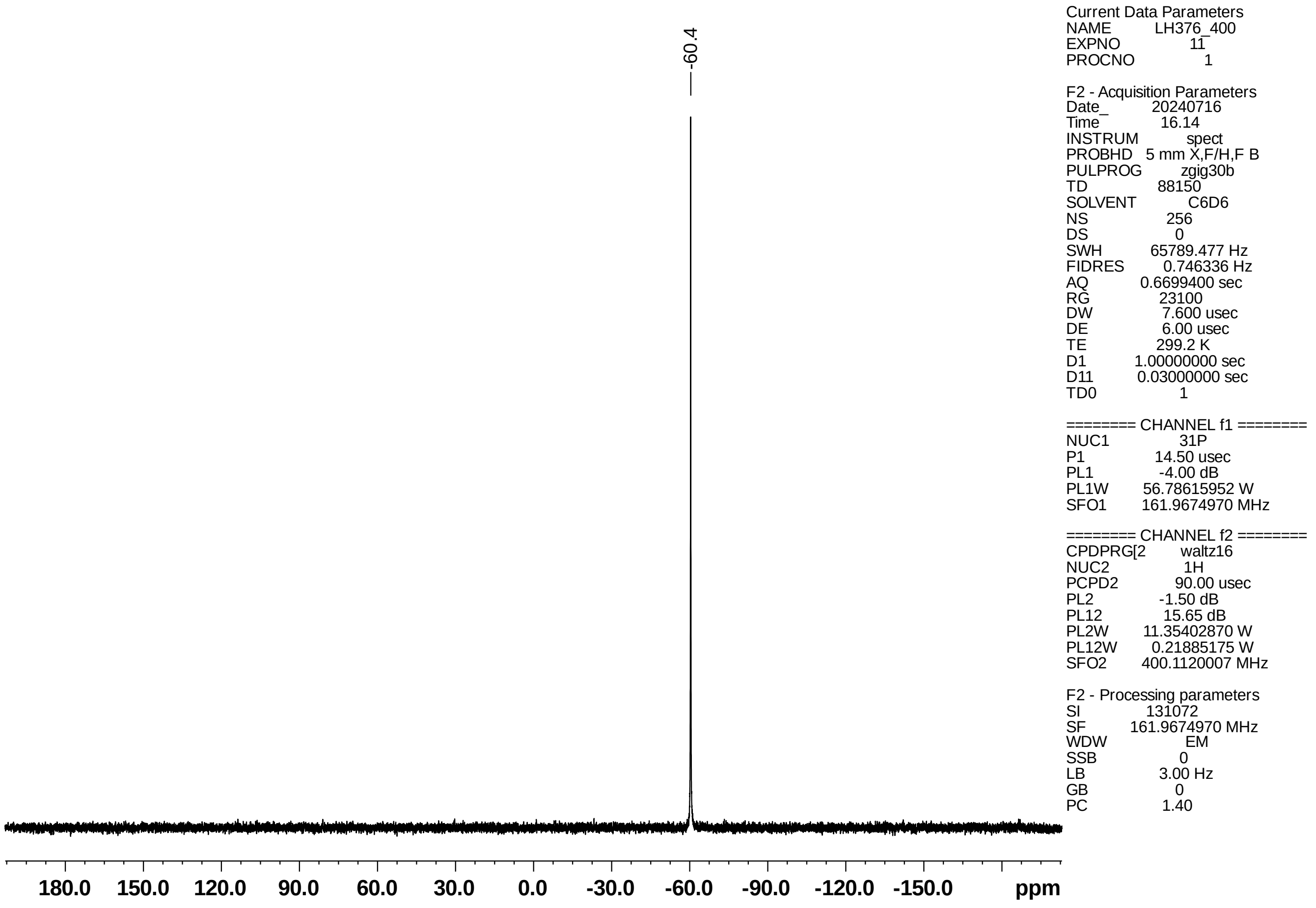

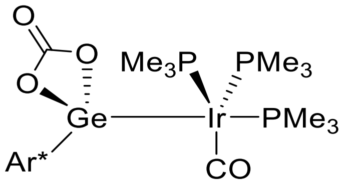

Figure SI55. $^{31}P\{^1H\}$ NMR of compound **12**.

NMR spectra of [Ar*Sn(μ-CO_2)Rh(PMe_3)$_3$] (**13**)

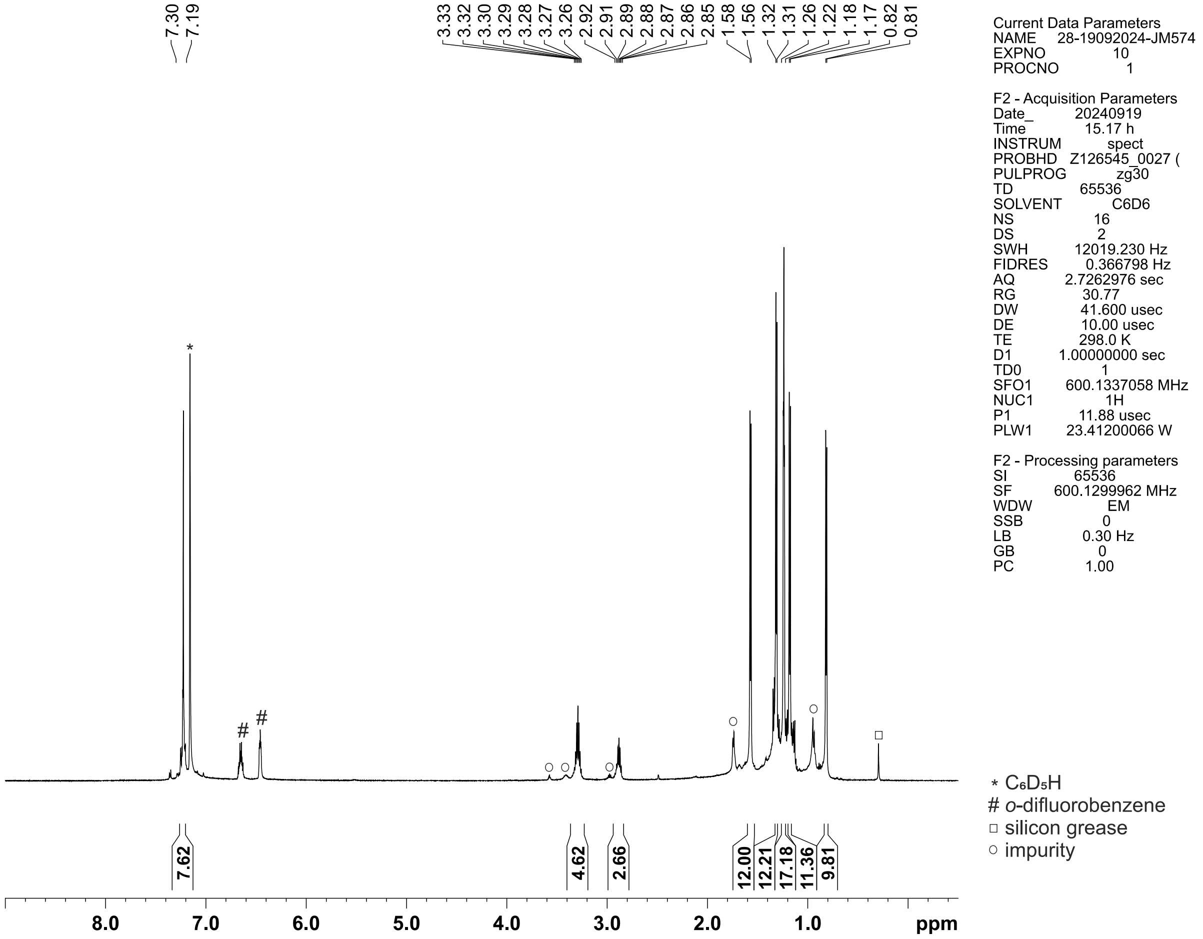

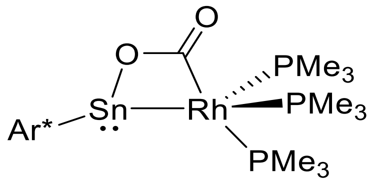

Figure SI56. ^{1}H NMR of compound **13**.

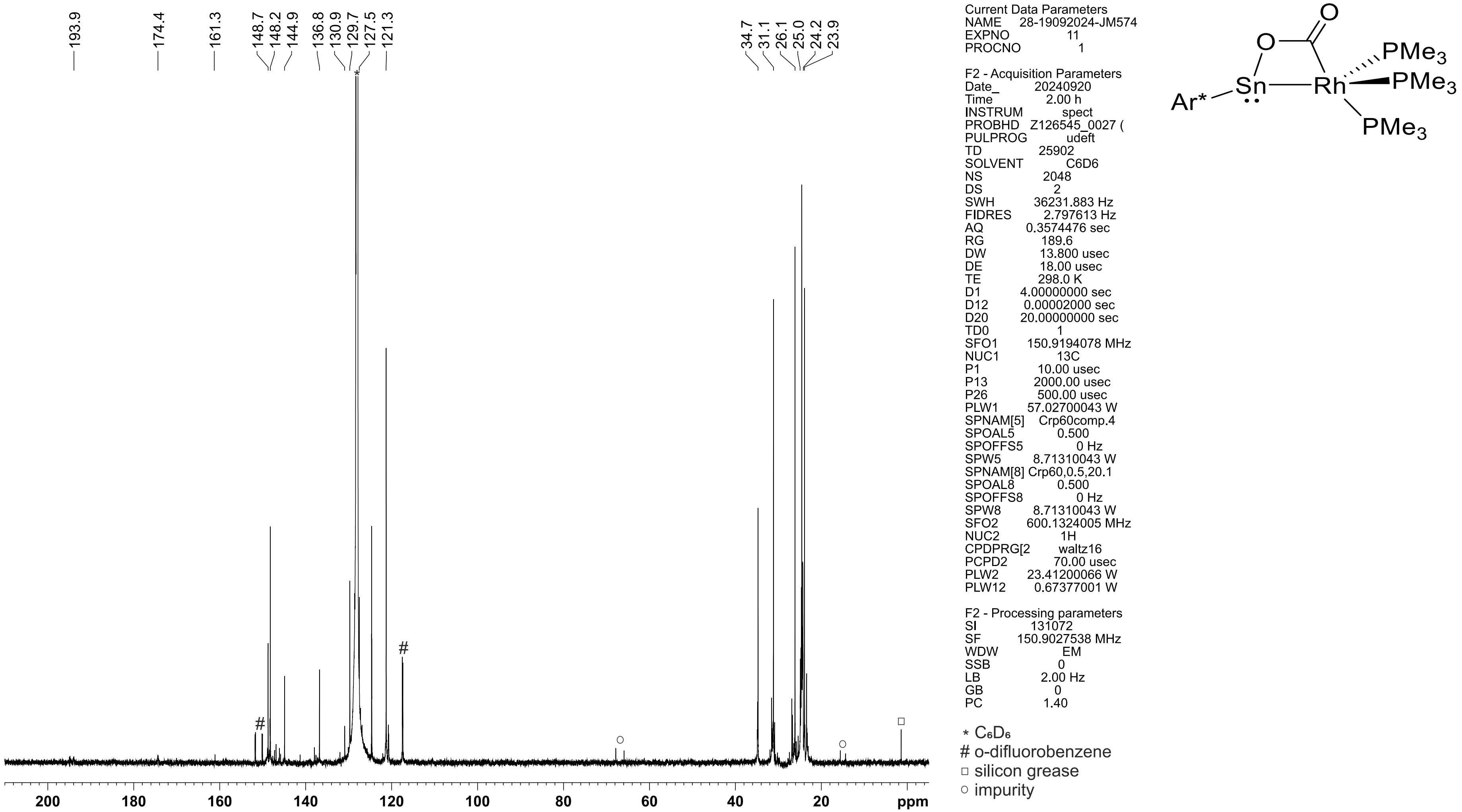

Figure SI57. $^{13}C\{^{1}H\}$ NMR of compound **13**.

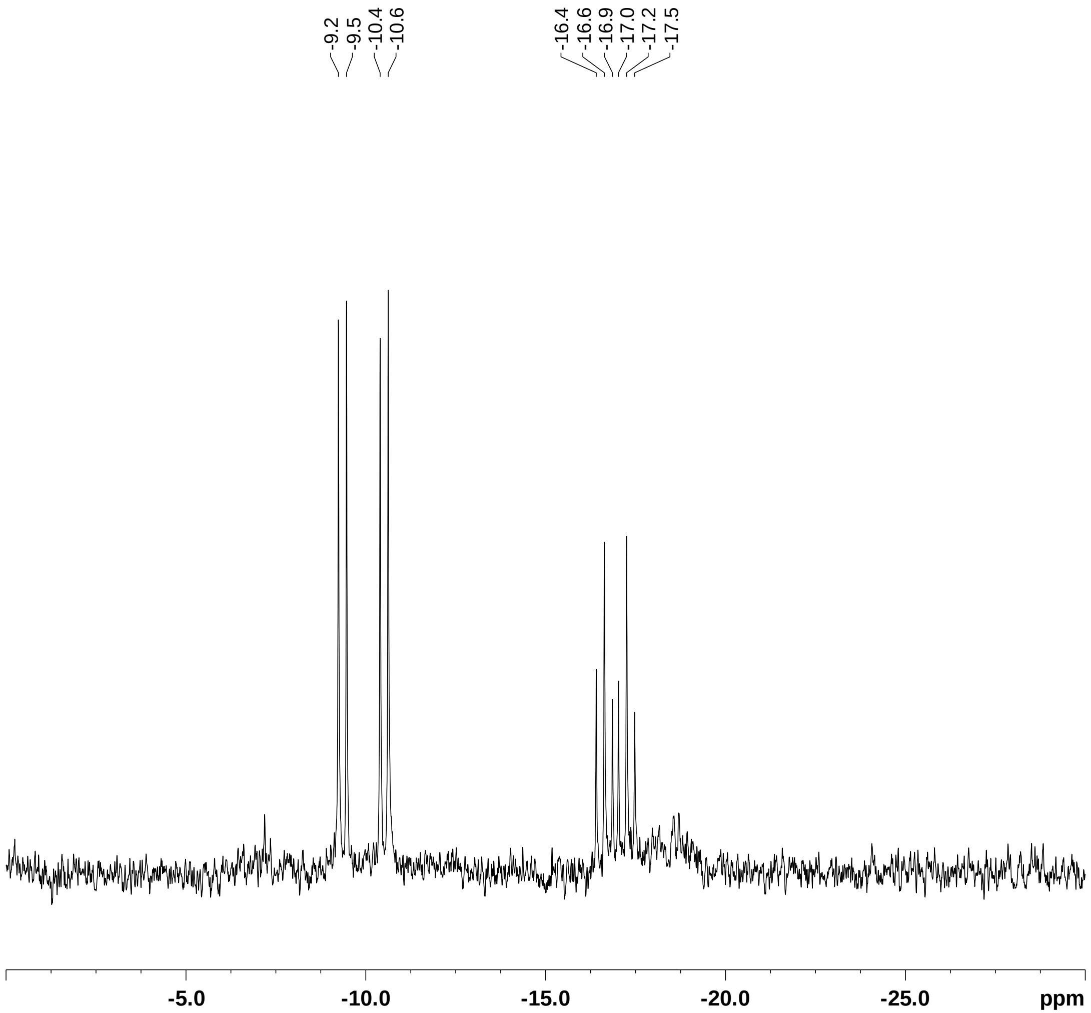

Current Data Parameters
NAME JM574_19092024_400
EXPNO 11
PROCNO 1

F2 - Acquisition Parameters
Date_ 20240919
Time 9.22
INSTRUM spect
PROBHD 5 mm X,F/H,F B
PULPROG zgig30b
TD 88150
SOLVENT C6D6
NS 128
DS 0
SWH 65789.477 Hz
FIDRES 0.746336 Hz
AQ 0.6699400 sec
RG 23100
DW 7.600 usec
DE 6.00 usec
TE 300.2 K
D1 1.00000000 sec
D11 0.03000000 sec
TD0 1

======== CHANNEL f1 ========
NUC1 31P
P1 14.50 usec
PL1 -4.00 dB
PL1W 56.78615952 W
SFO1 161.9674968 MHz

======== CHANNEL f2 ========
CPDPRG[2 waltz16
NUC2 1H
PCPD2 90.00 usec
PL2 -1.50 dB
PL12 15.65 dB
PL2W 11.35402870 W
PL12W 0.21885175 W
SFO2 400.1120007 MHz

F2 - Processing parameters
SI 131072
SF 161.9674970 MHz
WDW EM
SSB 0
LB 3.00 Hz
GB 0
PC 1.40

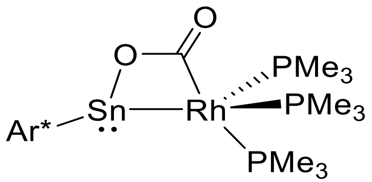

Figure SI58. $^{31}P\{^{1}H\}$ NMR of compound **13**.

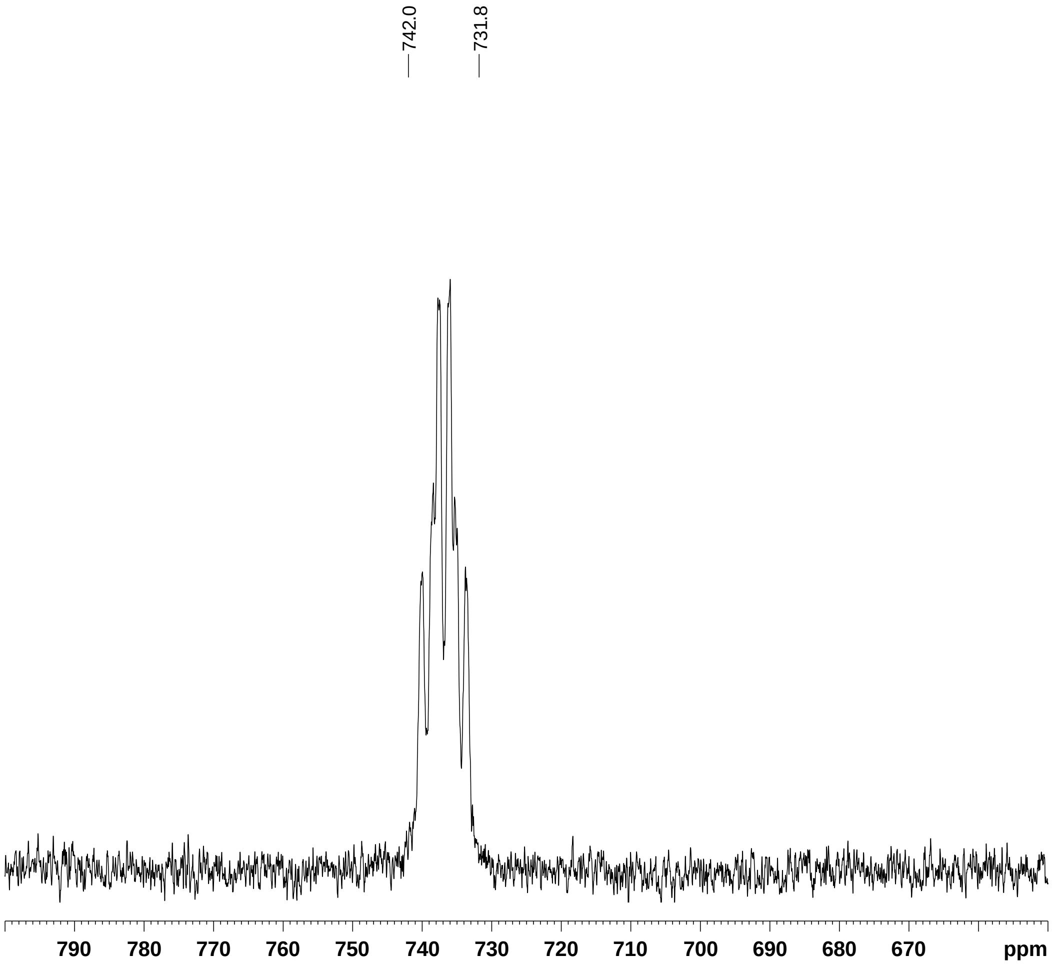

Current Data Parameters
NAME 8-09102024-NA
EXPNO 11
PROCNO 1

F2 - Acquisition Parameters
Date_ 20241008
Time 14.28 h
INSTRUM spect
PROBHD Z126545_0027 (
PULPROG zgig30
TD 65536
SOLVENT C6D6
NS 17591
DS 10
SWH 178571.422 Hz
FIDRES 5.449567 Hz
AQ 0.1835008 sec
RG 189.6
DW 2.800 usec
DE 18.00 usec
TE 298.0 K
D1 0.10000000 sec
D11 0.03000000 sec
TD0 1
SFO1 223.9041660 MHz
NUC1 119Sn
P1 14.25 usec
PLW1 50.00000000 W
SFO2 600.1324005 MHz
NUC2 1H
CPDPRG[2 waltz16
PCPD2 70.00 usec
PLW2 23.41200066 W
PLW12 0.67377001 W

F2 - Processing parameters
SI 65536
SF 223.7922698 MHz
WDW EM
SSB 0
LB 20.00 Hz
GB 0
PC 1.00

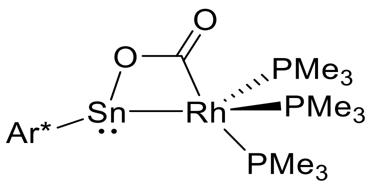

Figure SI59. $^{119}Sn\{^{1}H\}$ NMR of compound **13**.

NMR spectra of [Ar*Sn(CO_3)Rh(CO)(PMe_3)$_3$] (**14**)

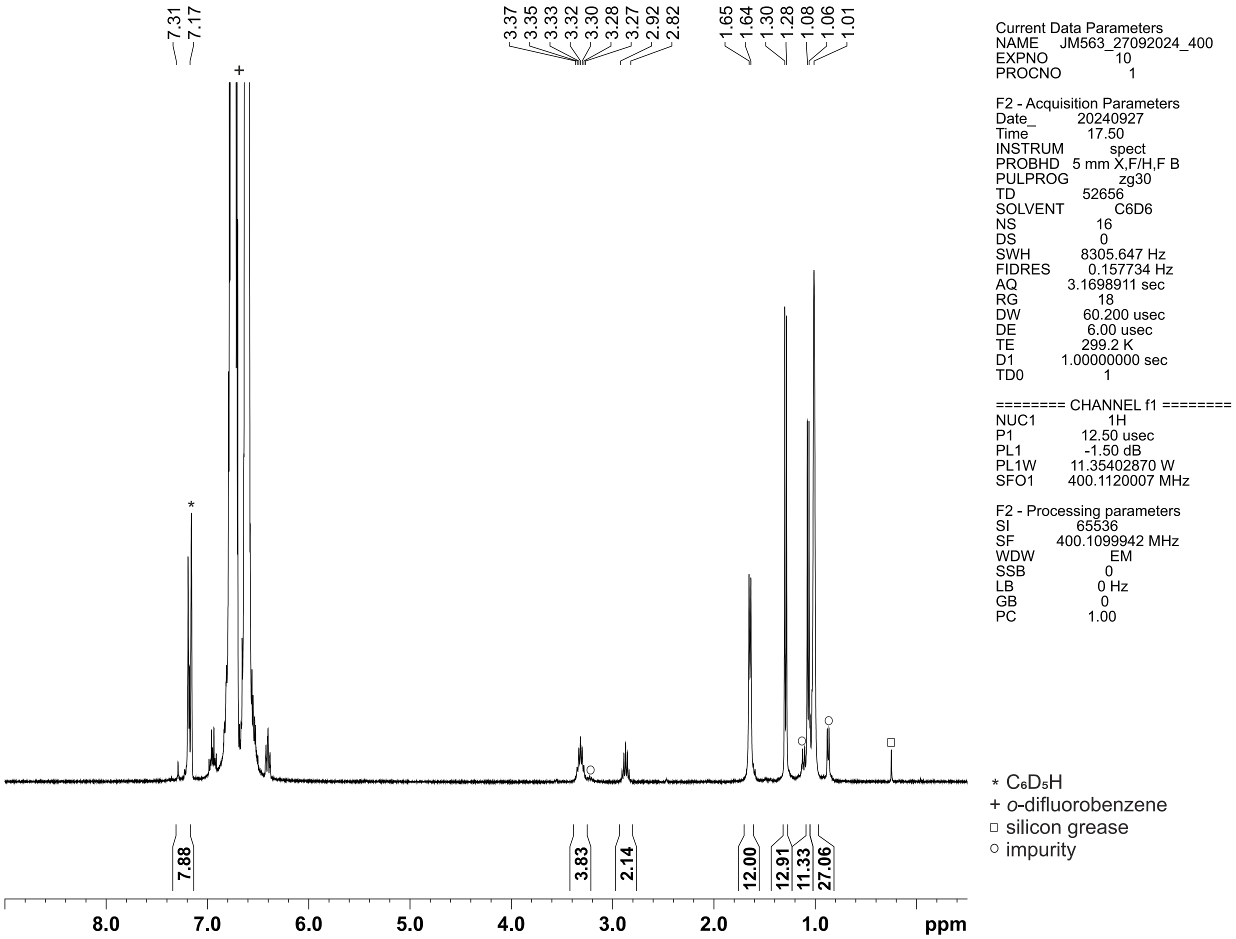

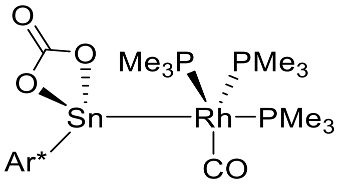

Figure SI60. ^{1}H NMR of compound **14**.

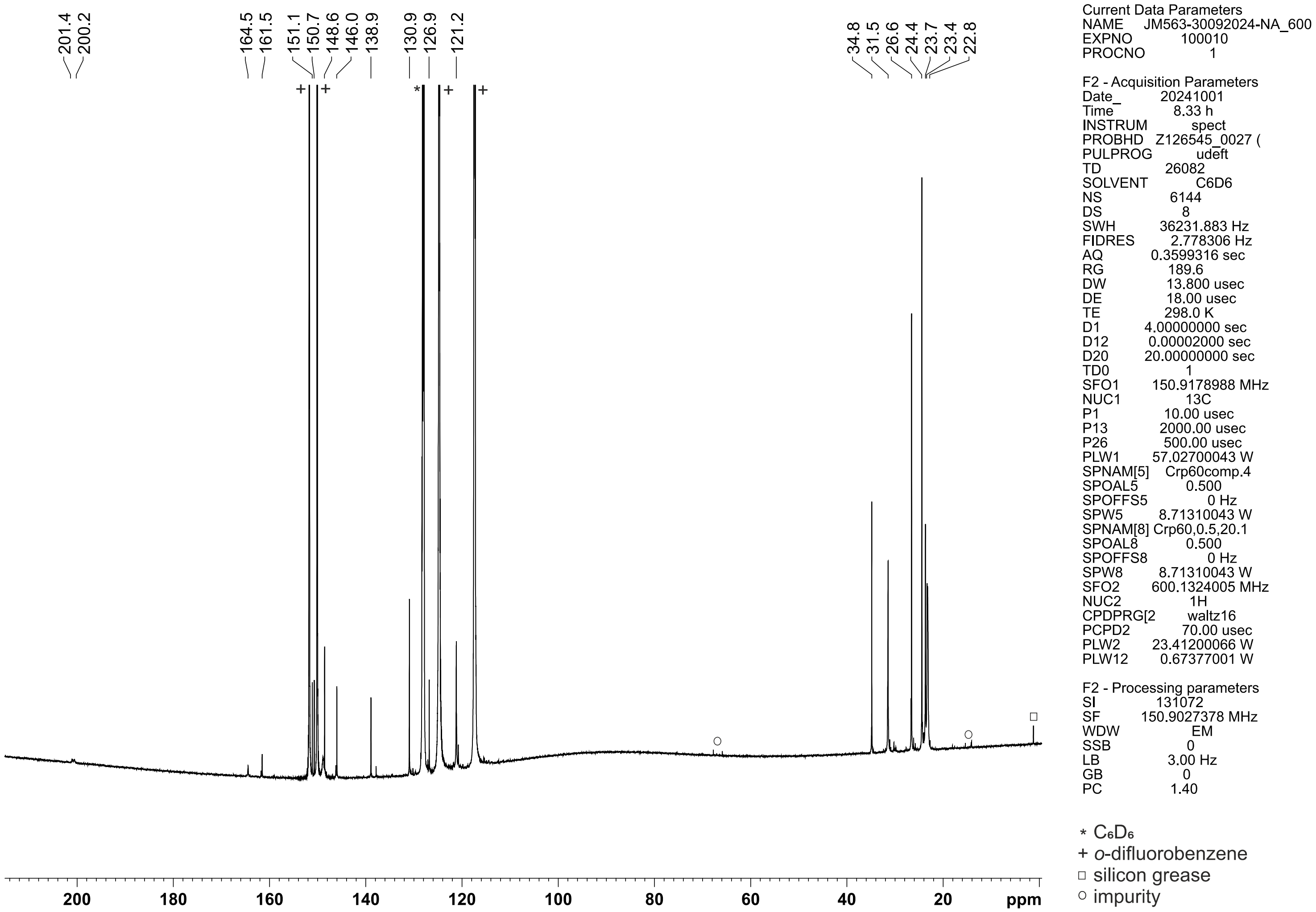

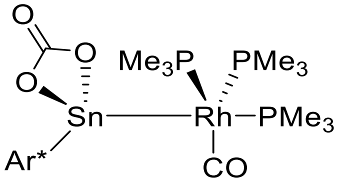

Figure SI61. $^{13}C\{^{1}H\}$ NMR of compound **14**.

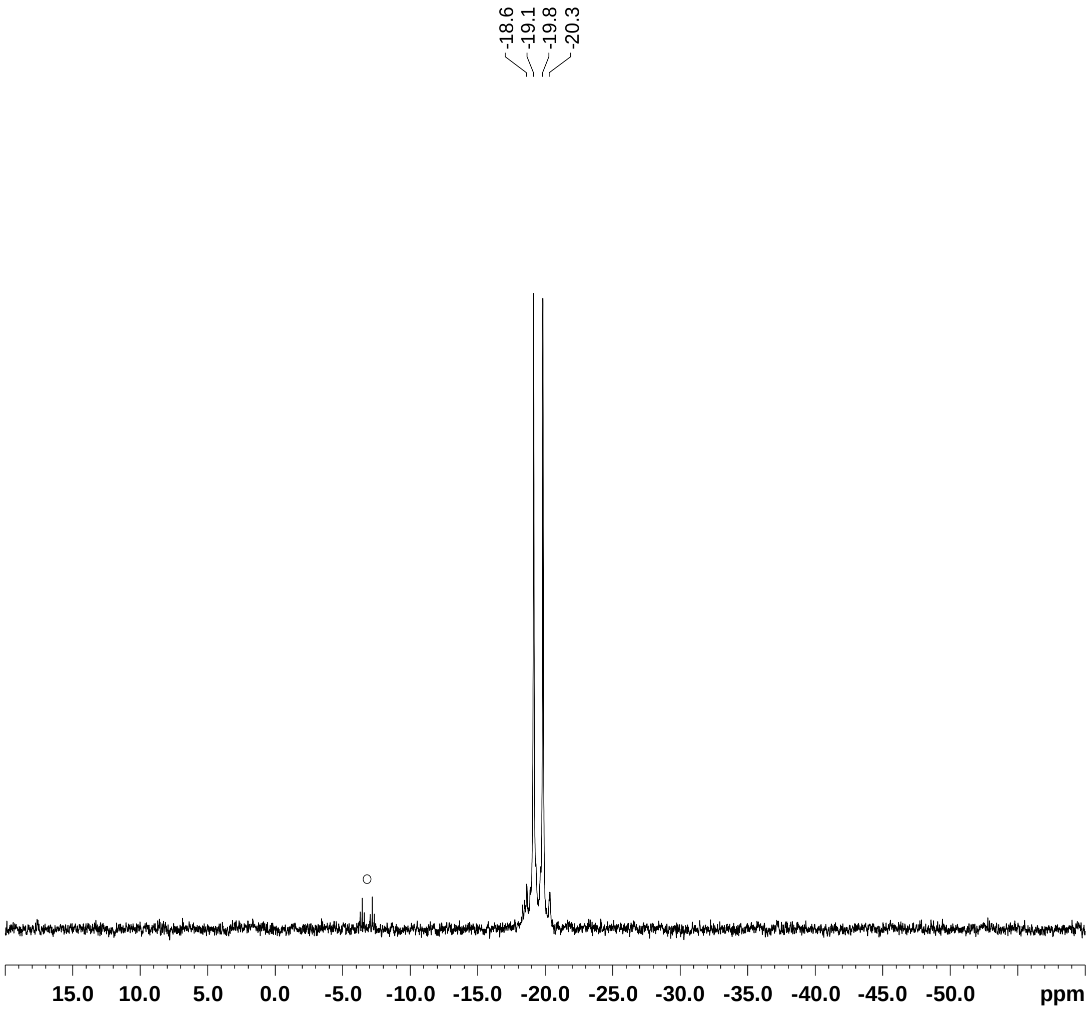

Current Data Parameters
NAME JM563_27092024_400
EXPNO 11
PROCNO 1

F2 - Acquisition Parameters
Date_ 20240927
Time 17.54
INSTRUM spect
PROBHD 5 mm X,F/H,F B
PULPROG zgig30b
TD 88150
SOLVENT C6D6
NS 128
DS 0
SWH 65789.477 Hz
FIDRES 0.746336 Hz
AQ 0.6699400 sec
RG 23100
DW 7.600 usec
DE 6.00 usec
TE 299.2 K
D1 1.00000000 sec
D11 0.03000000 sec
TD0 1

======== CHANNEL f1 ========
NUC1 31P
P1 14.50 usec
PL1 -4.00 dB
PL1W 56.78615952 W
SFO1 161.9674968 MHz

======== CHANNEL f2 ========
CPDPRG[2 waltz16
NUC2 1H
PCPD2 90.00 usec
PL2 -1.50 dB
PL12 15.65 dB
PL2W 11.35402870 W
PL12W 0.21885175 W
SFO2 400.1120007 MHz

F2 - Processing parameters
SI 131072
SF 161.9674970 MHz
WDW EM
SSB 0
LB 3.00 Hz
GB 0
PC 1.40

○ impurity

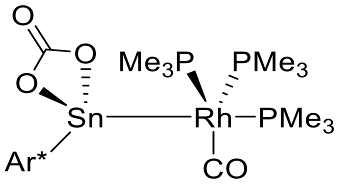

Figure SI62. $^{31}P\{^{1}H\}$ NMR of compound **14**.

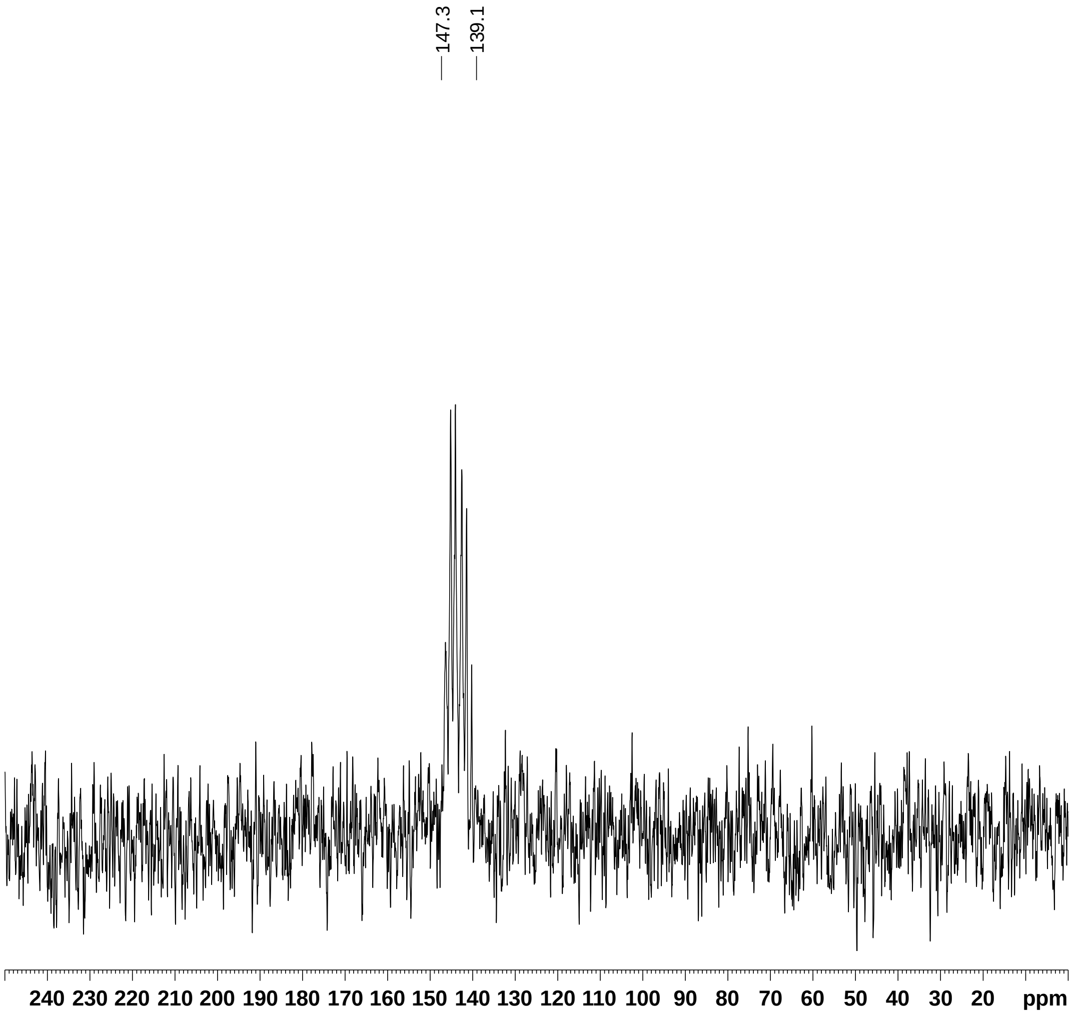

Current Data Parameters
NAME JM563_27092024_400
EXPNO 13
PROCNO 1

F2 - Acquisition Parameters
Date_ 20240928
Time 5.25
INSTRUM spect
PROBHD 5 mm X,F/H,F B
PULPROG zgig30b
TD 32768
SOLVENT C6D6
NS 16000
DS 0
SWH 150000.000 Hz
FIDRES 4.577637 Hz
AQ 0.1092267 sec
RG 16400
DW 3.333 usec
DE 6.00 usec
TE 299.2 K
D1 0.10000000 sec
D11 0.03000000 sec
TD0 1

======== CHANNEL f1 ========
NUC1 119Sn
P1 6.81 usec
PL1 -4.00 dB
SFO1 149.2483092 MHz

======== CHANNEL f2 ========
CPDPRG[2 waltz16
NUC2 1H
PCPD2 90.00 usec
PL2 -1.50 dB
PL12 15.65 dB
PL2W 11.35402870 W
PL12W 0.21885175 W
SFO2 400.1120006 MHz

F2 - Processing parameters
SI 32768
SF 149.2035480 MHz
WDW EM
SSB 0
LB 20.00 Hz
GB 0
PC 1.40

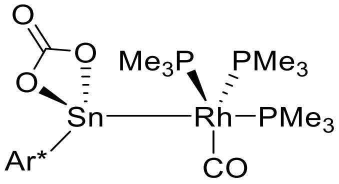

Figure SI63. ^{119}Sn{^{1}H} NMR of compound **14**.

NMR spectra of [Ar*Sn(μ-CO_2)Ir(PMe_3)$_3$] (**15**)

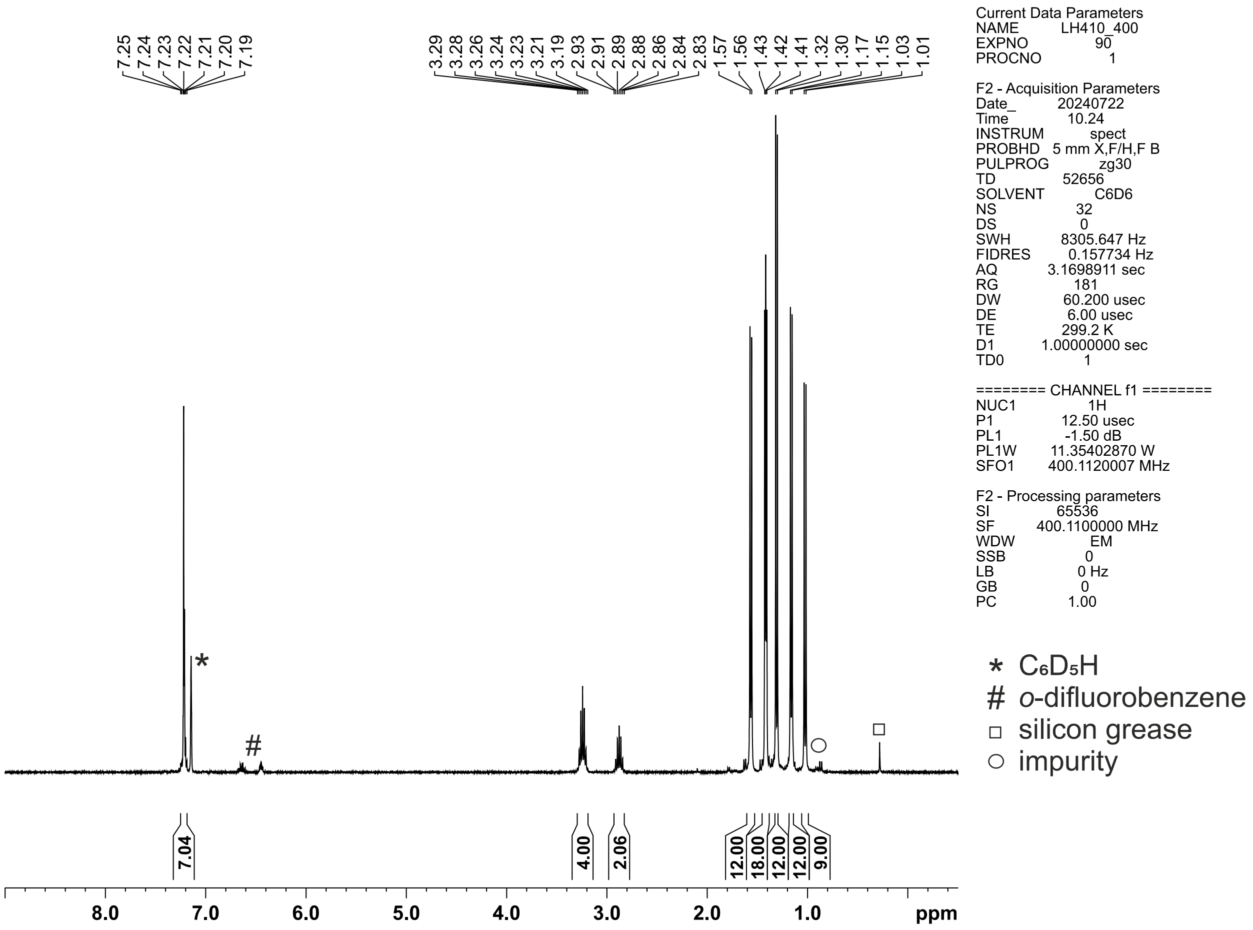

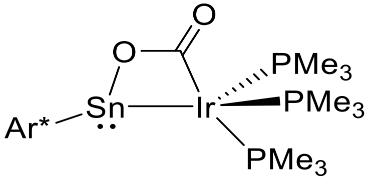

Figure SI64. ^{1}H NMR of compound **15**.

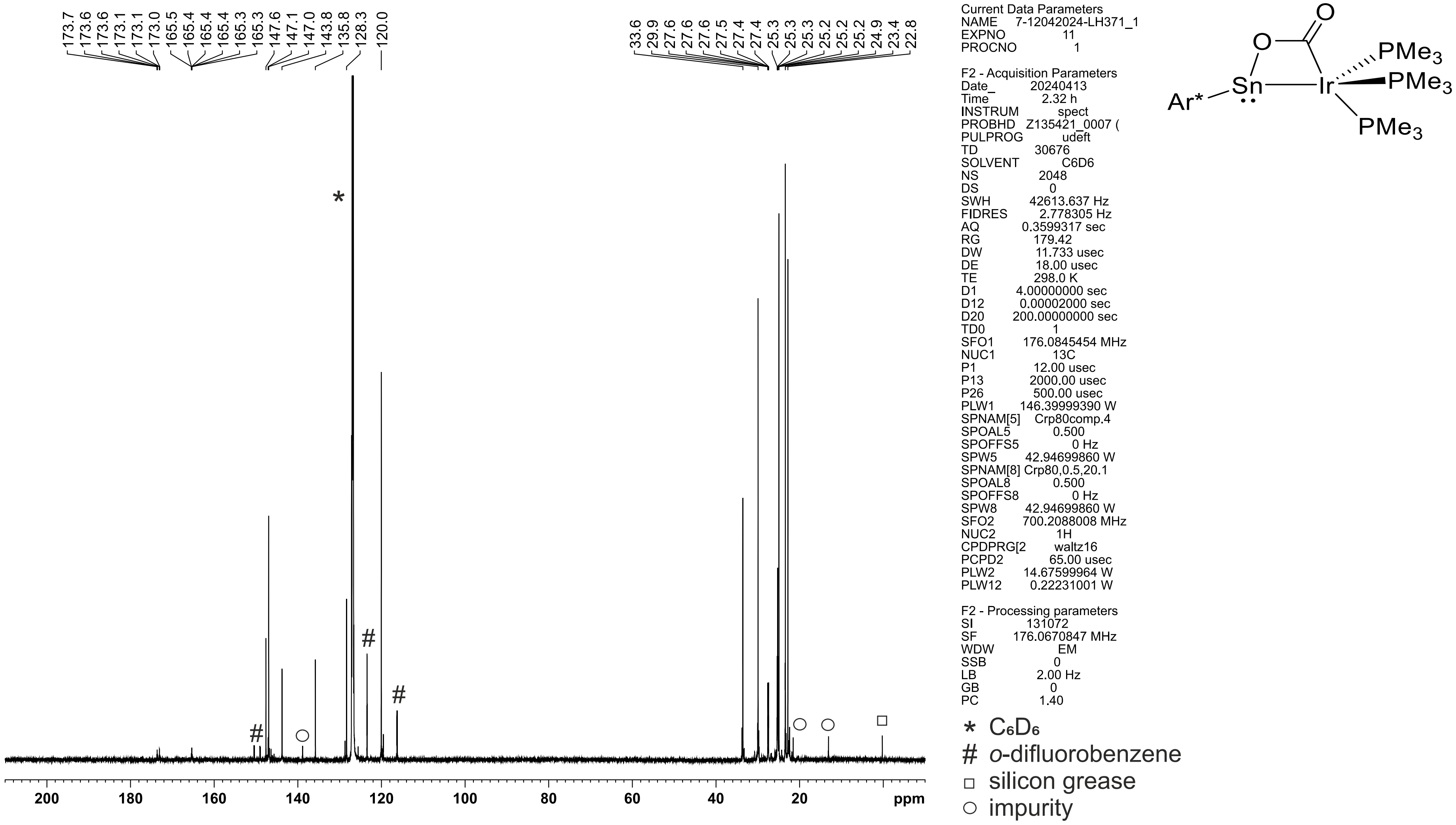

Figure SI65. $^{13}C\{^{1}H\}$ NMR of compound **15**.

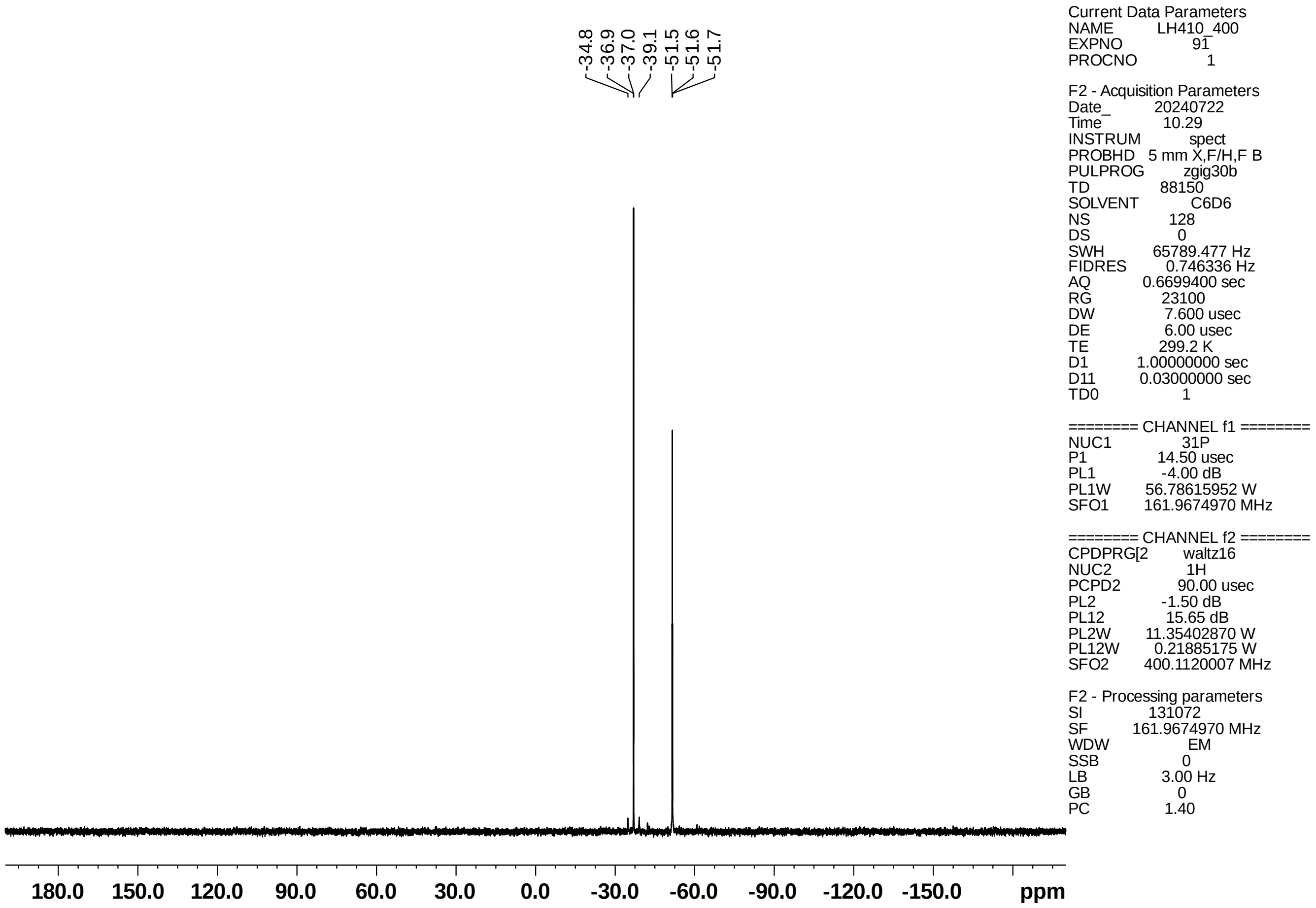

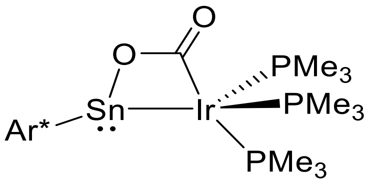

Figure SI66. $^{31}P\{^{1}H\}$ NMR of compound **15**.

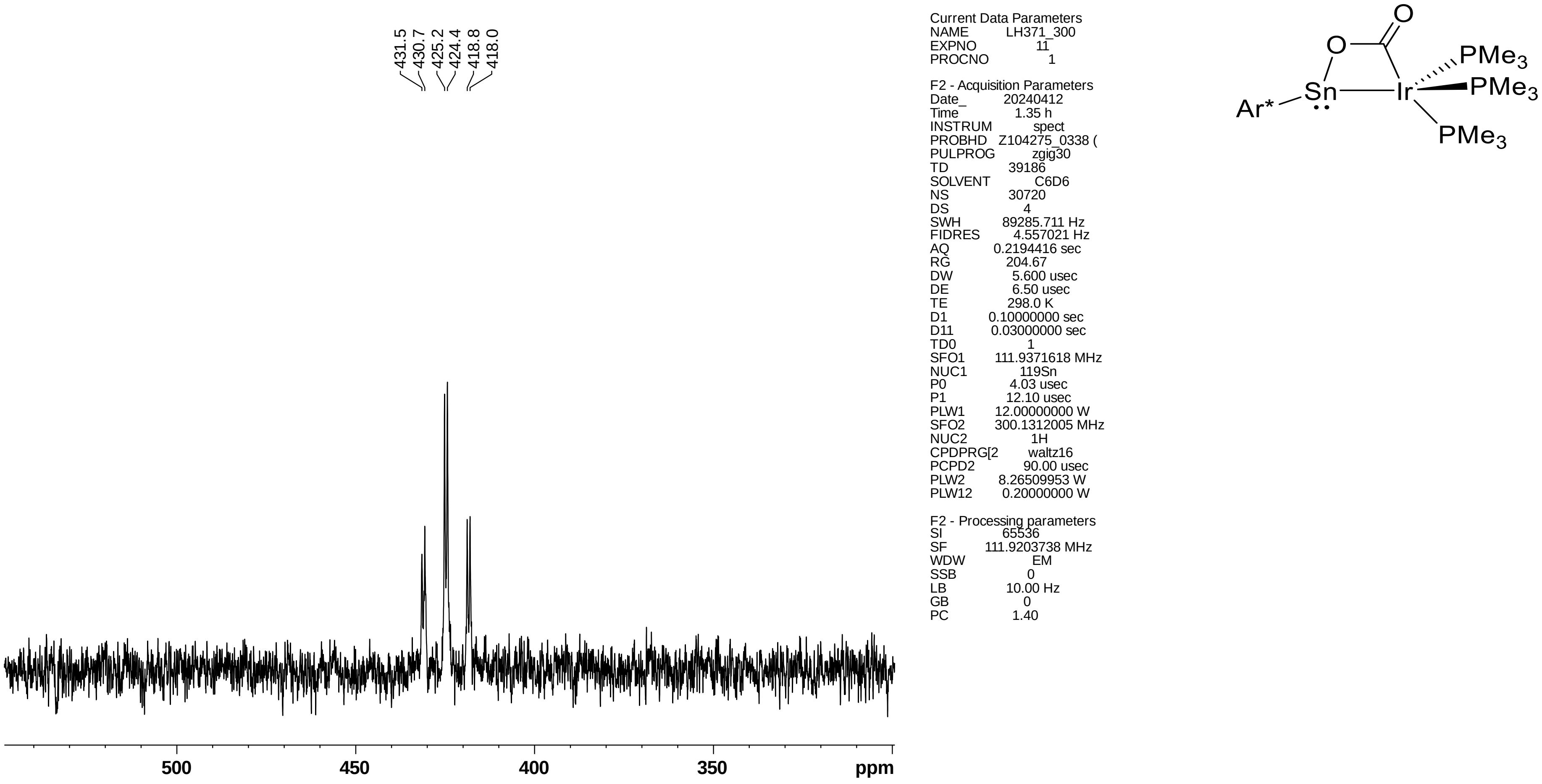

Figure SI67. $^{119}Sn\{^{1}H\}$ NMR of compound **15**.

NMR spectra of [Ar*Sn(CO_3)Ir(CO)(PMe_3)$_3$] (**16**)

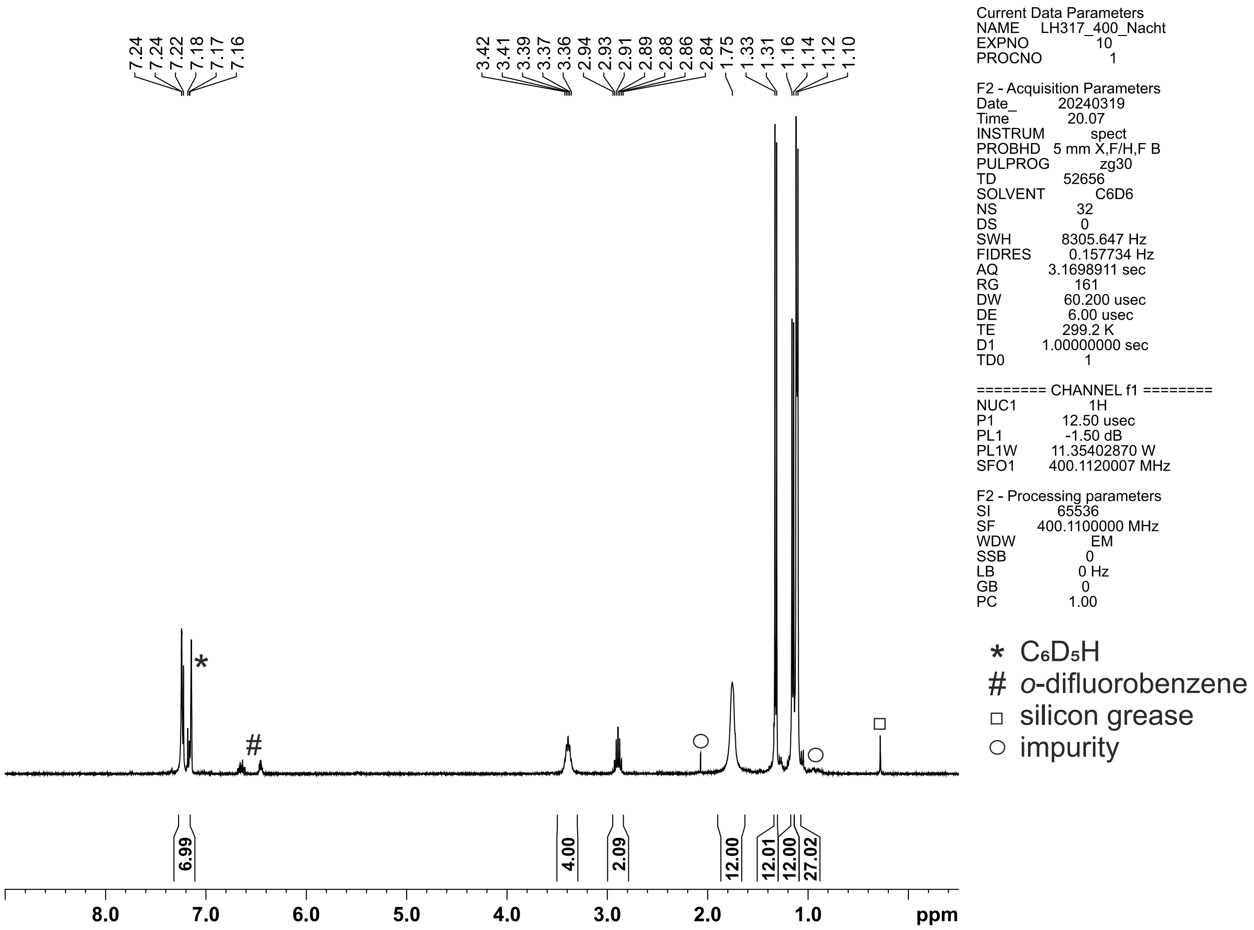

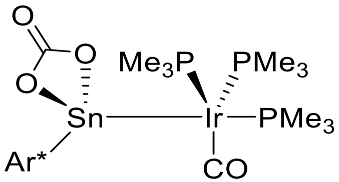

Figure SI68. ^{1}H NMR of compound **16**.

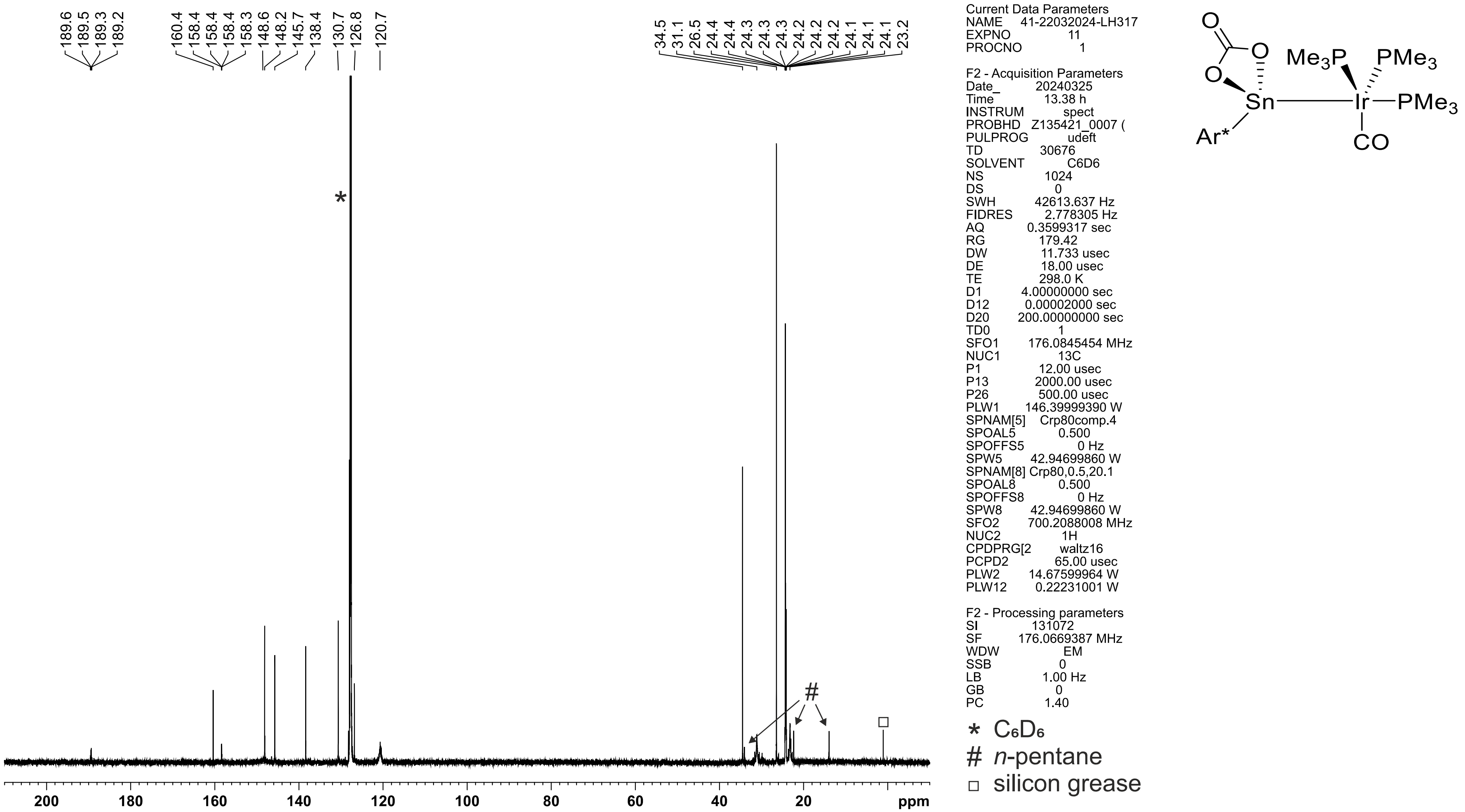

Figure SI69. $^{13}C\{^{1}H\}$ NMR of compound **16**.

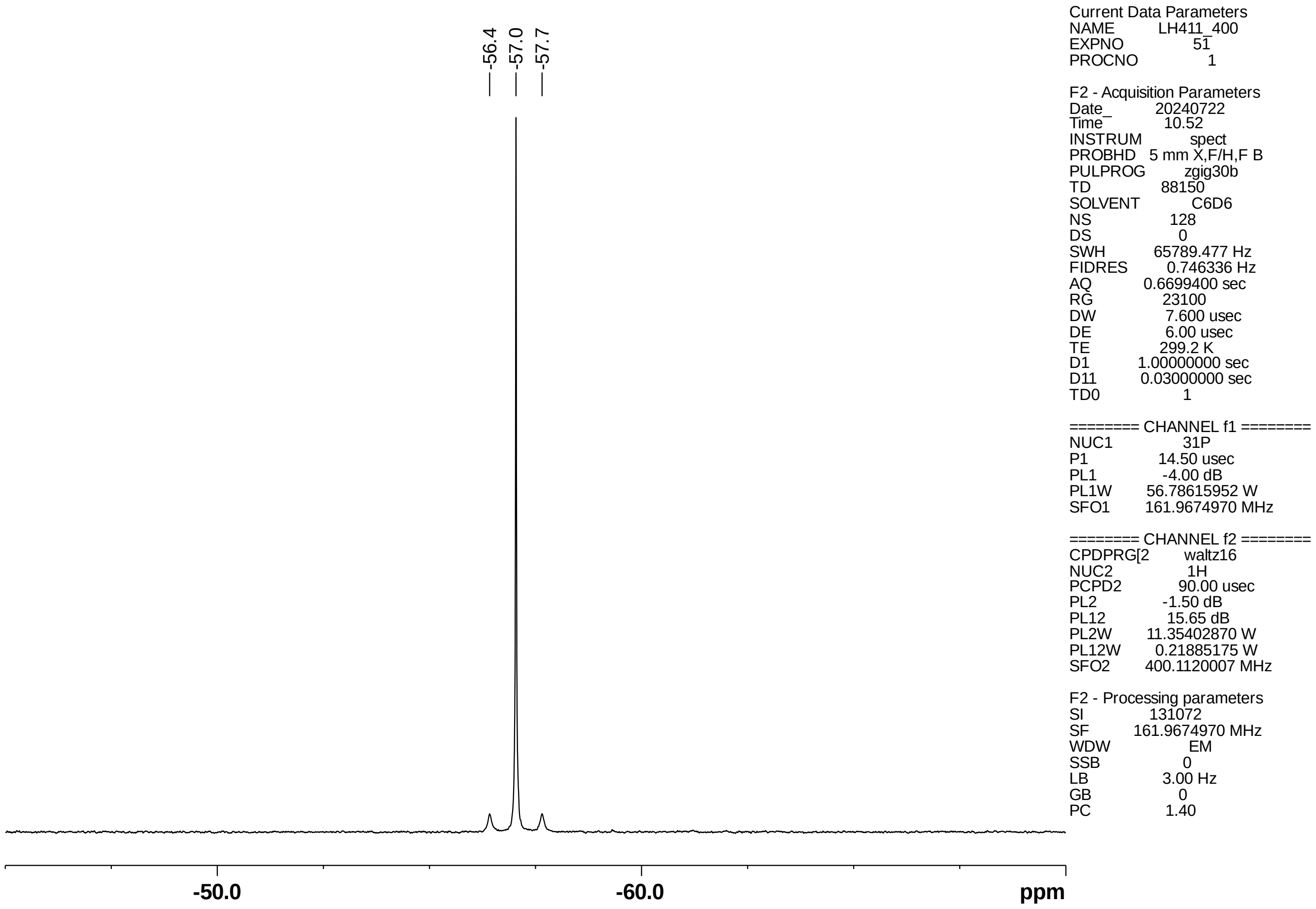

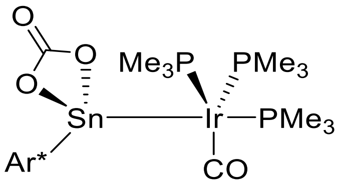

Figure SI70. $^{31}P\{^{1}H\}$ NMR of compound **16**.

NMR spectra of the metathesis reactions

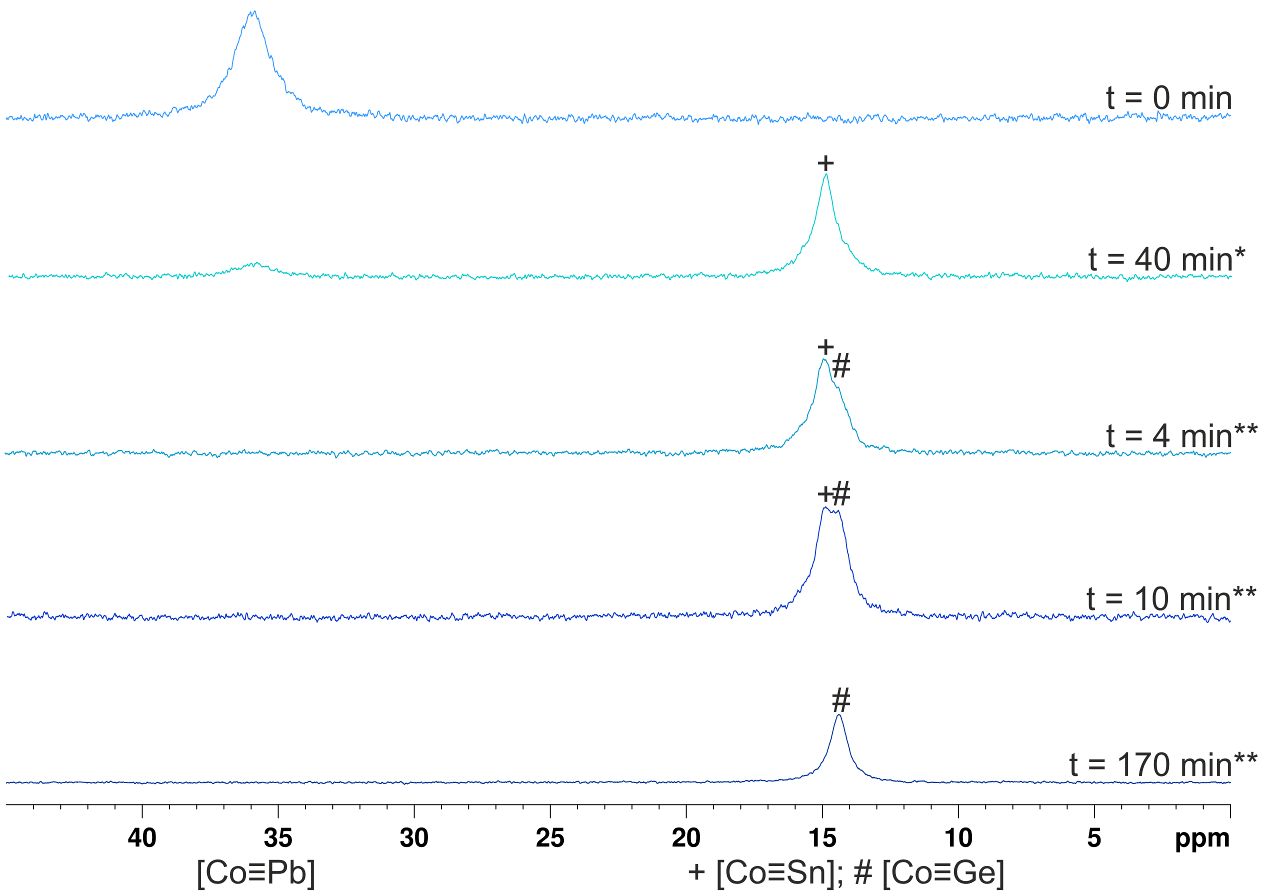

Figure SI71. $^{31}P\{^{1}H\}$ NMR of the metathetical exchange of [Co≡Pb] with [Ar*SnCl] and [Ar*GeCl] to initially yield [Co≡Sn] and finally [Co≡Ge] **1**.
* After addition of one equivalent of [Ar*SnCl]; * After addition of one equivalent of [Ar*GeCl].

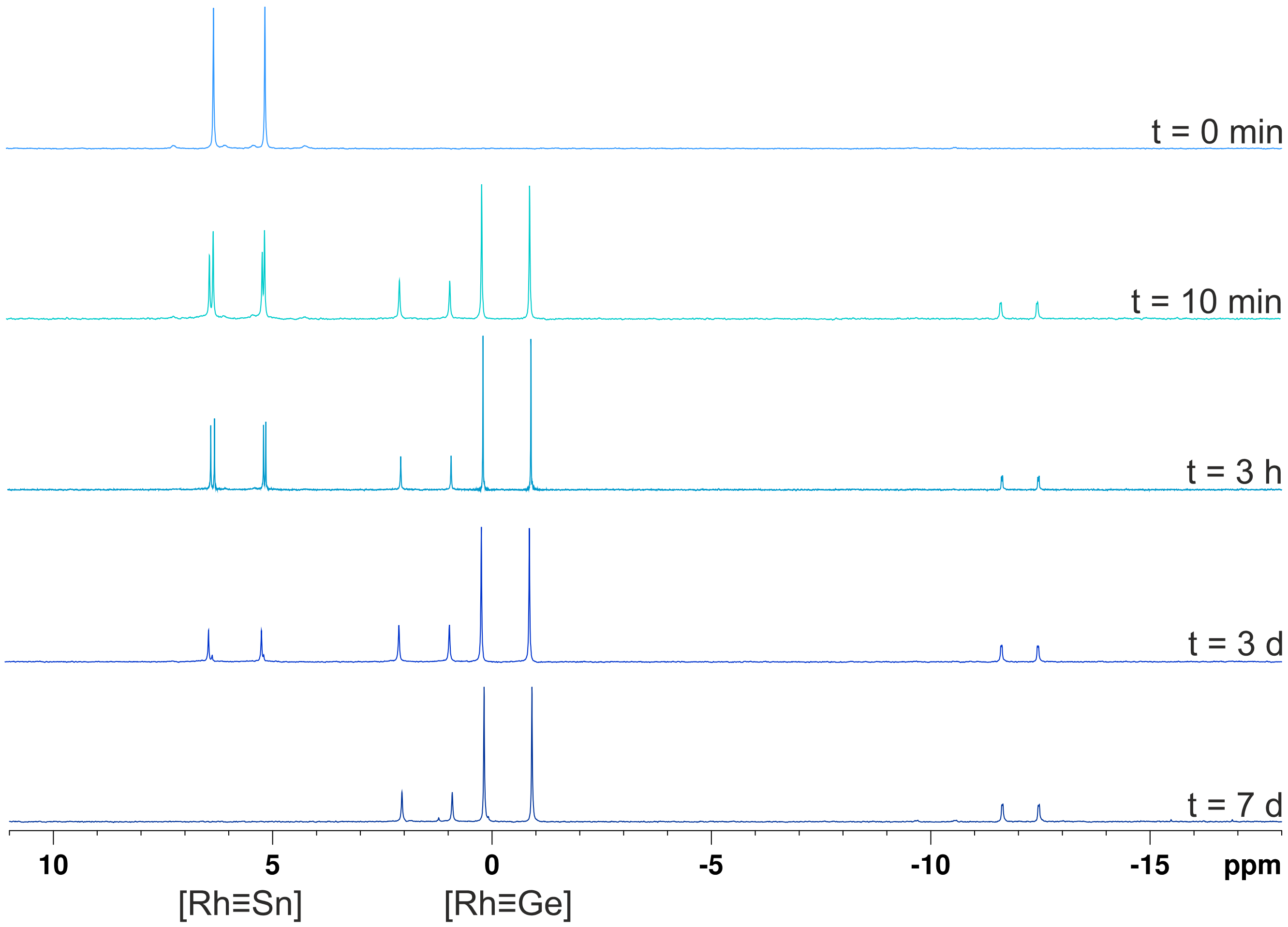

Figure SI72. $^{31}P\{^{1}H\}$ NMR of the metathetical exchange of [Rh≡Sn] **4** with [Ar*GeCl] to yield [Rh≡Ge] **2** and [Ar*SnCl] while several minor side products are formed (–12.0 ppm and 1.5 ppm).

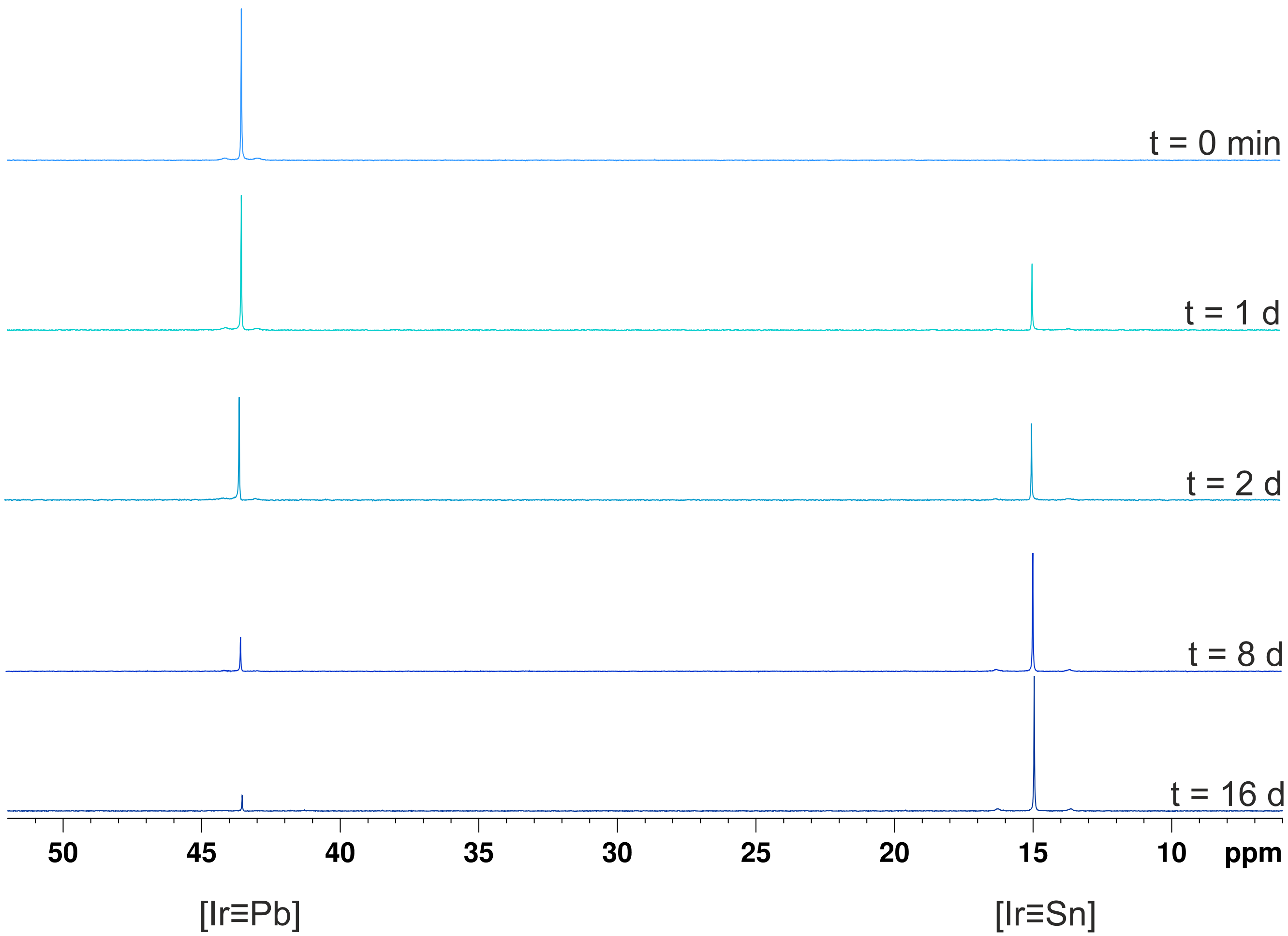

Figure SI73. $^{31}P\{^{1}H\}$ NMR of the metathetical exchange of [Ir≡Pb] **7** with [Ar*SnCl] to yield [Ir≡Sn] **5** and [Ar*PbCl].

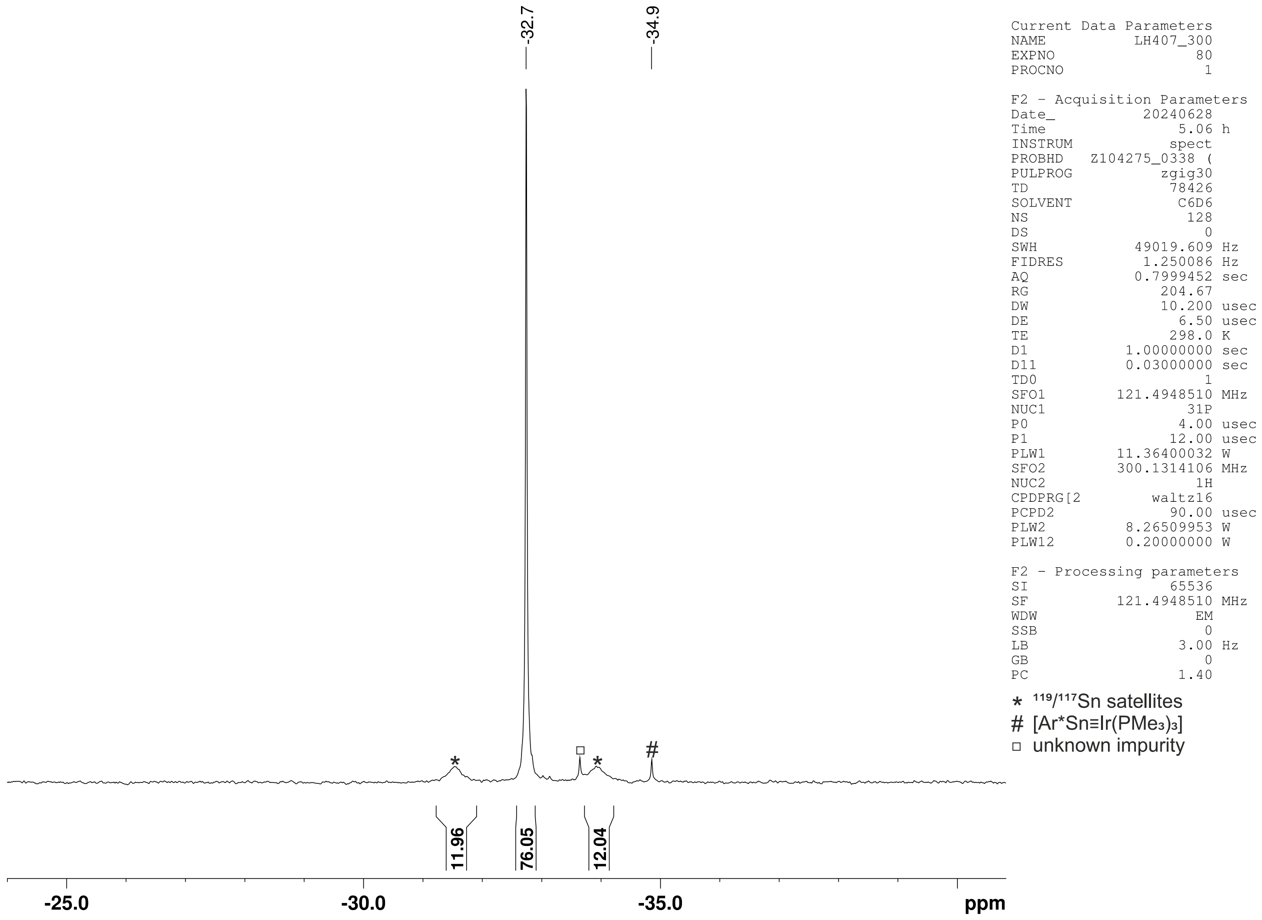

Figure SI74. $^{31}P\{^{1}H\}$ NMR spectrum of a mixture between $[(Me_3P)_3Ir{\equiv}SnAr^*]$ and three equivalents of Ar*SnCl.

IR spectroscopy.

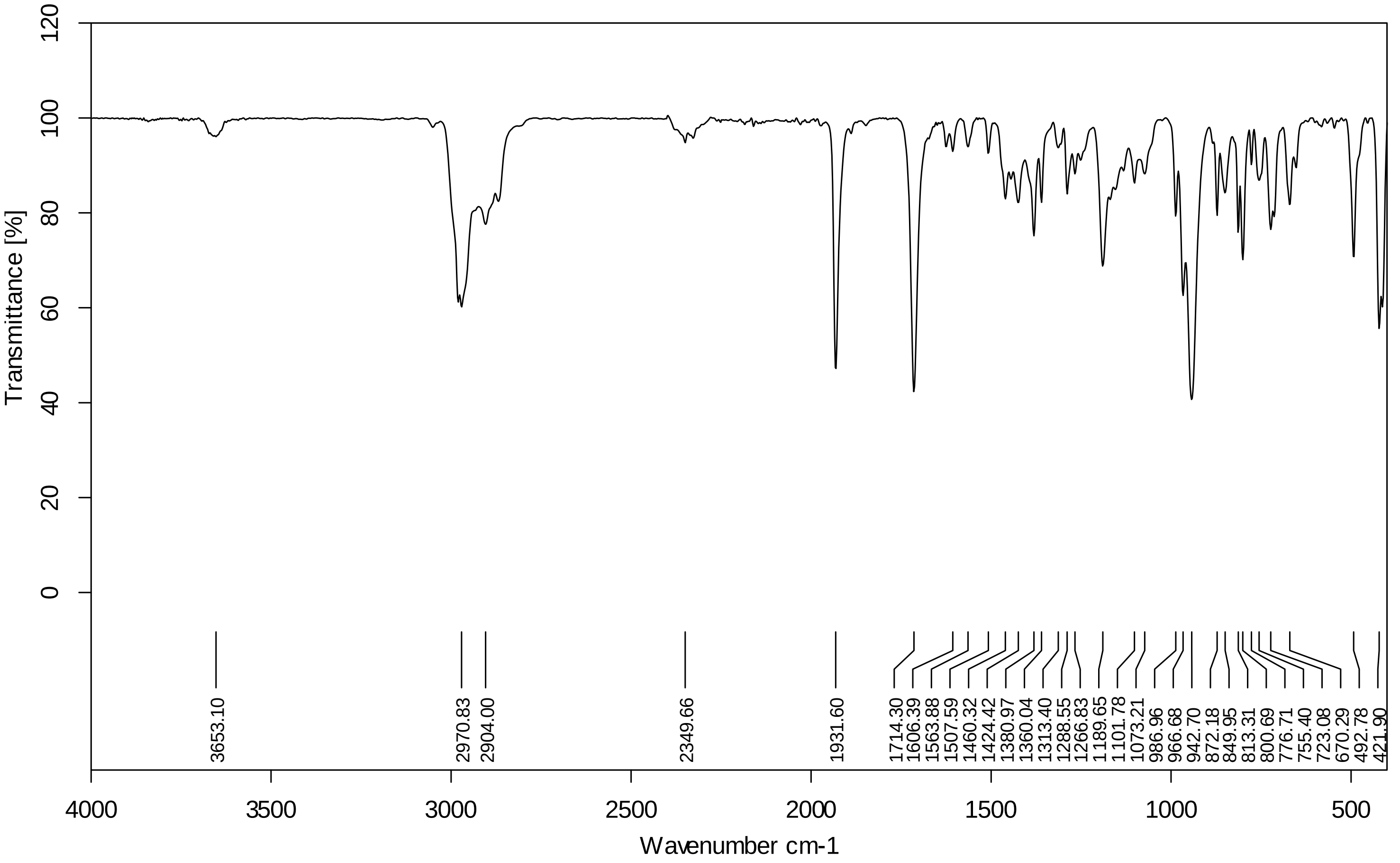

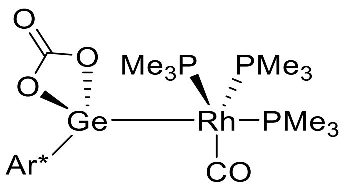

Figure SI75. IR spectrum of compound **11**.

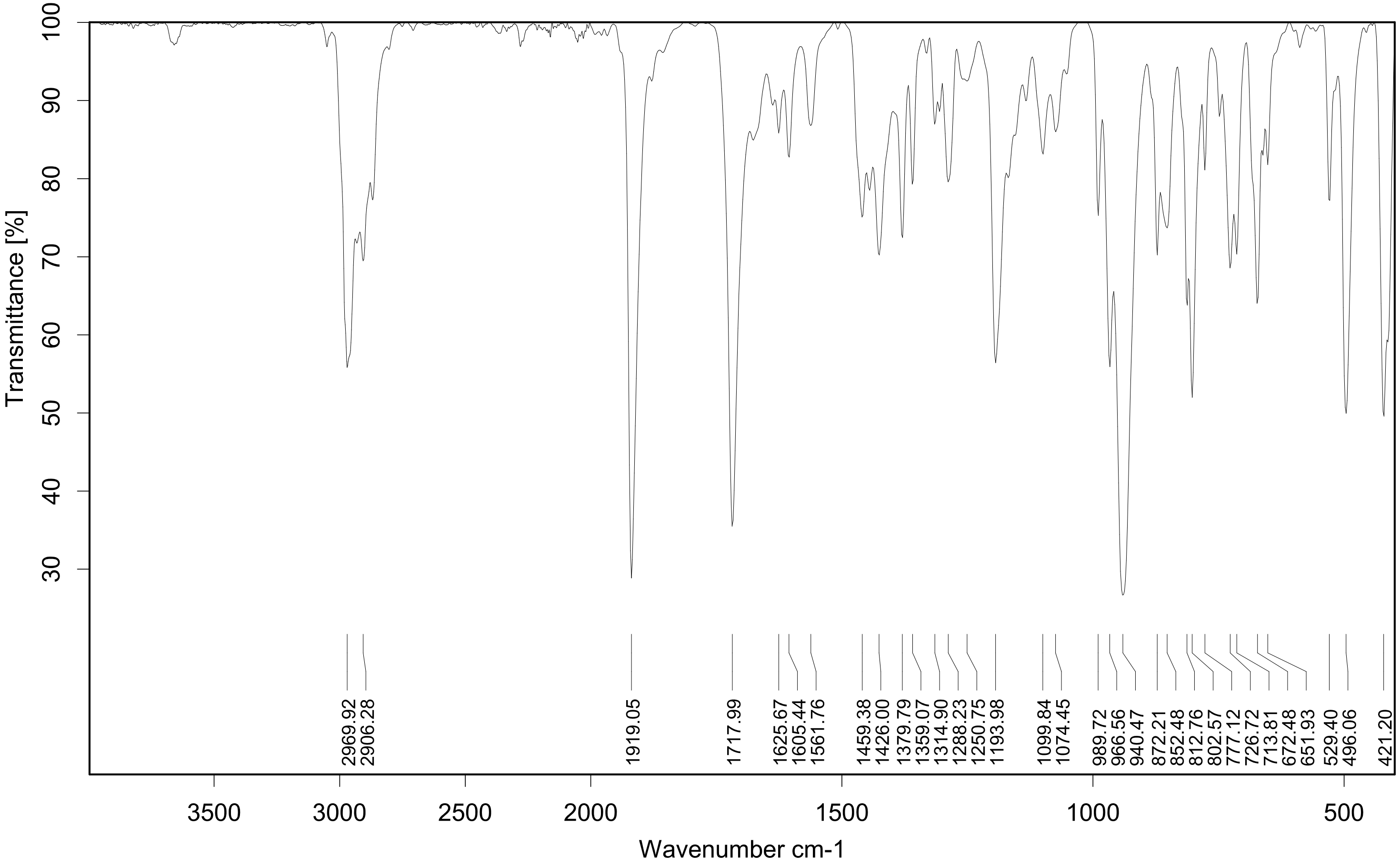

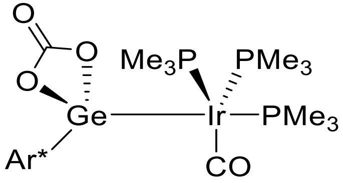

Figure SI76. IR spectrum of compound **12**.

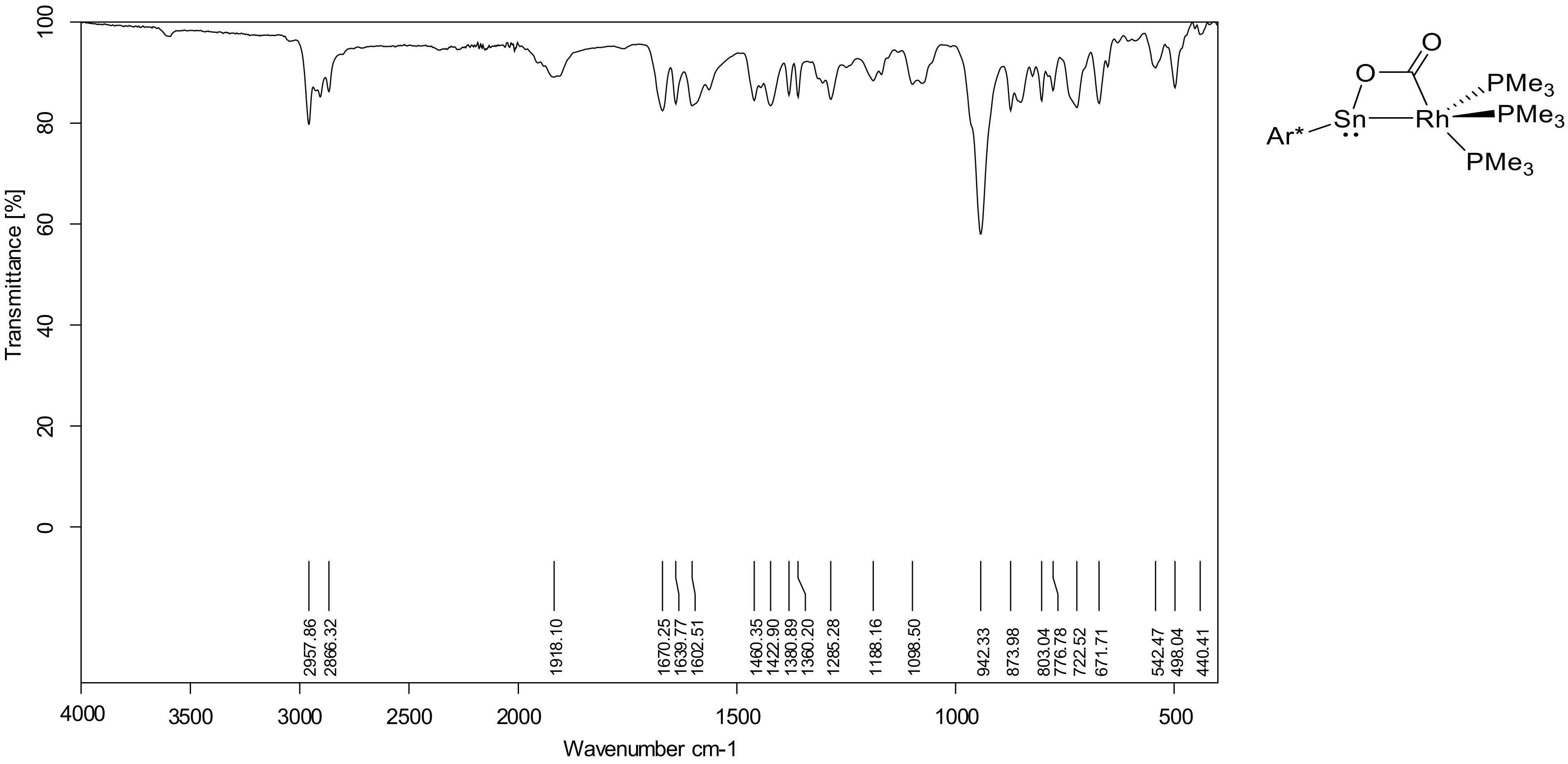

Figure SI77. IR spectrum of compound **13**.

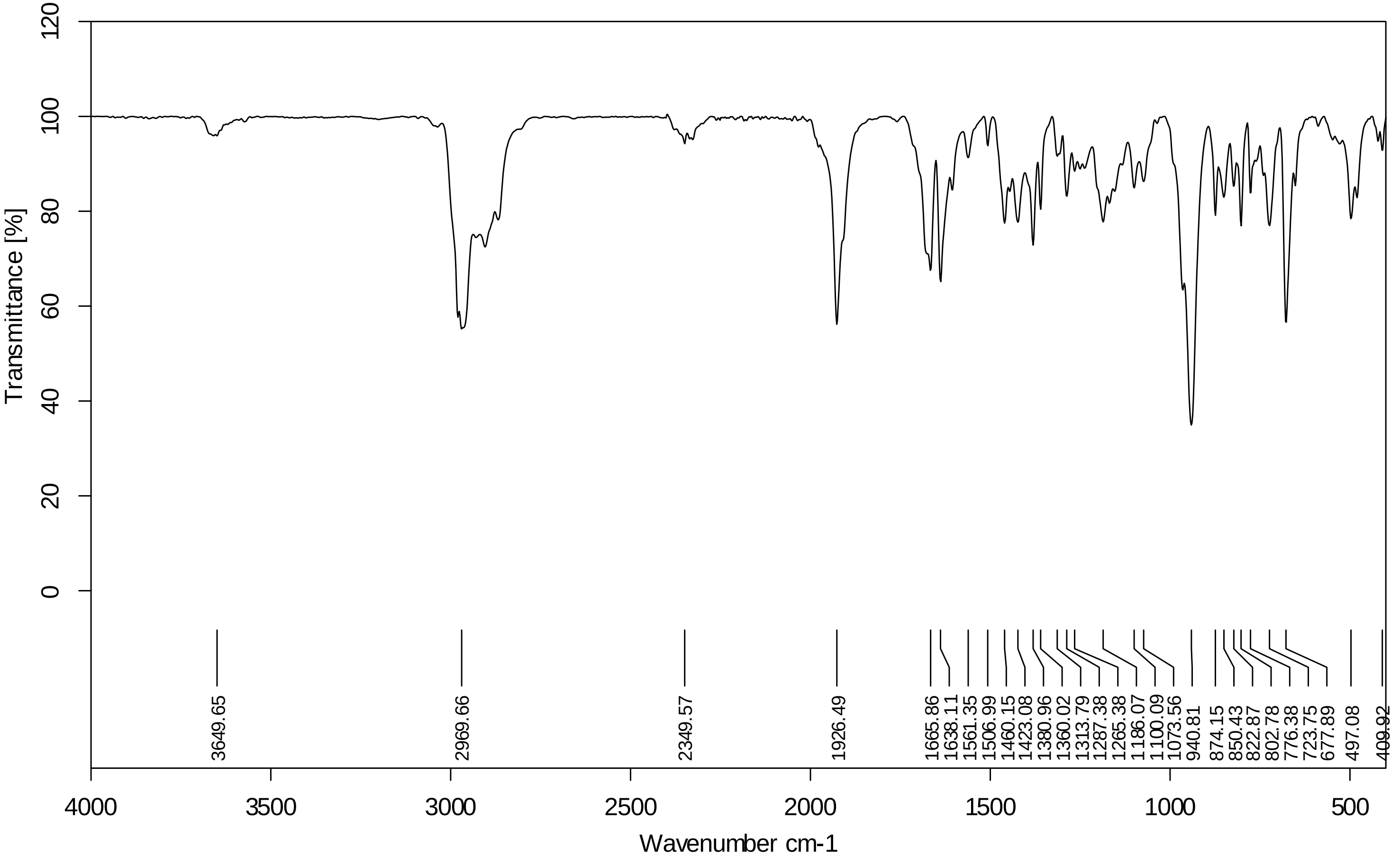

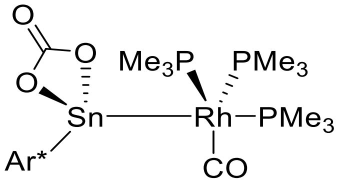

Figure SI78. IR spectrum of compound **14**.

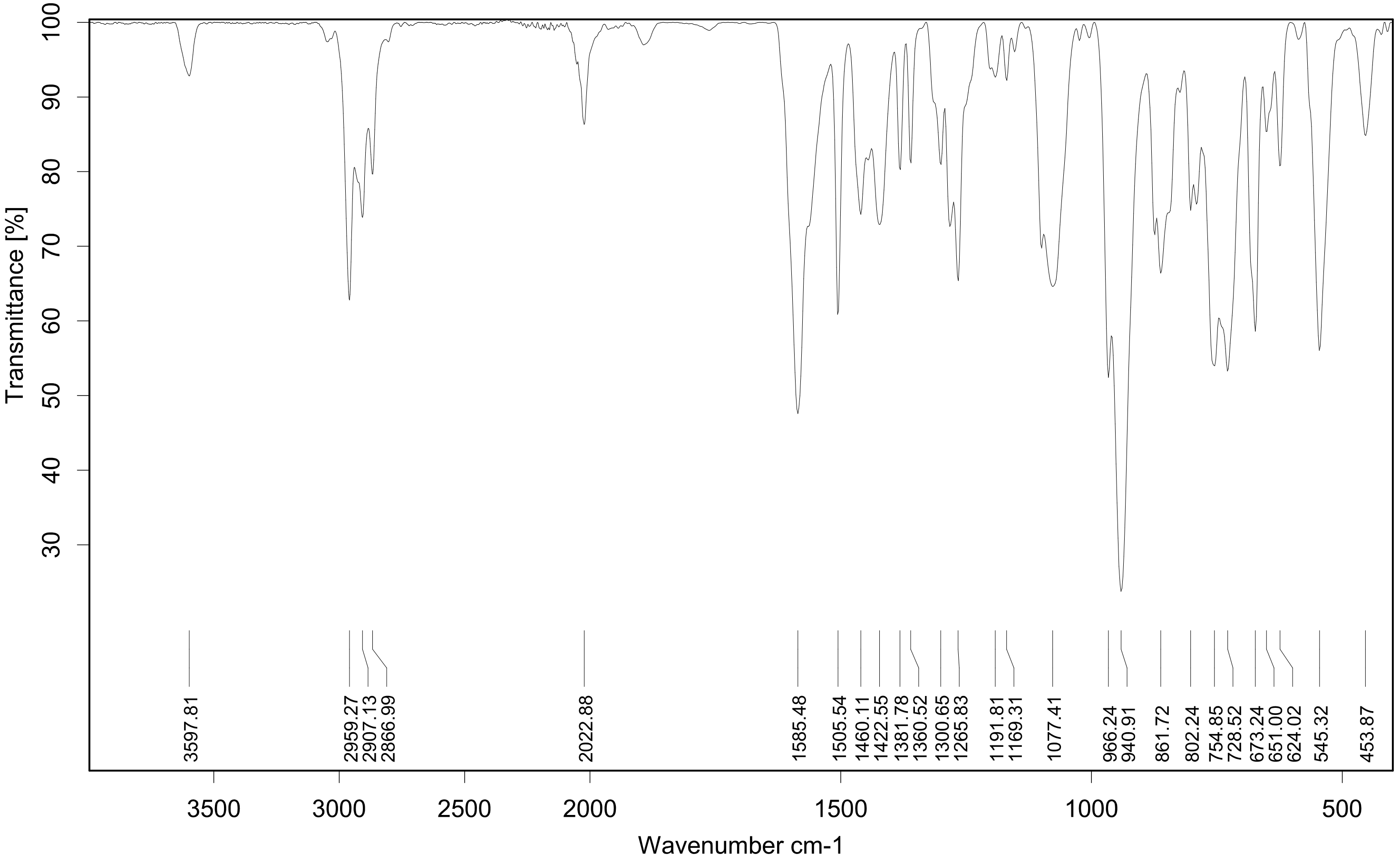

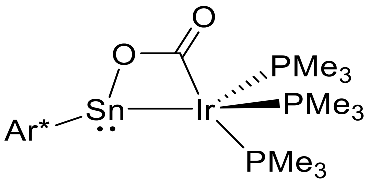

Figure SI79. IR spectrum of compound **15**.

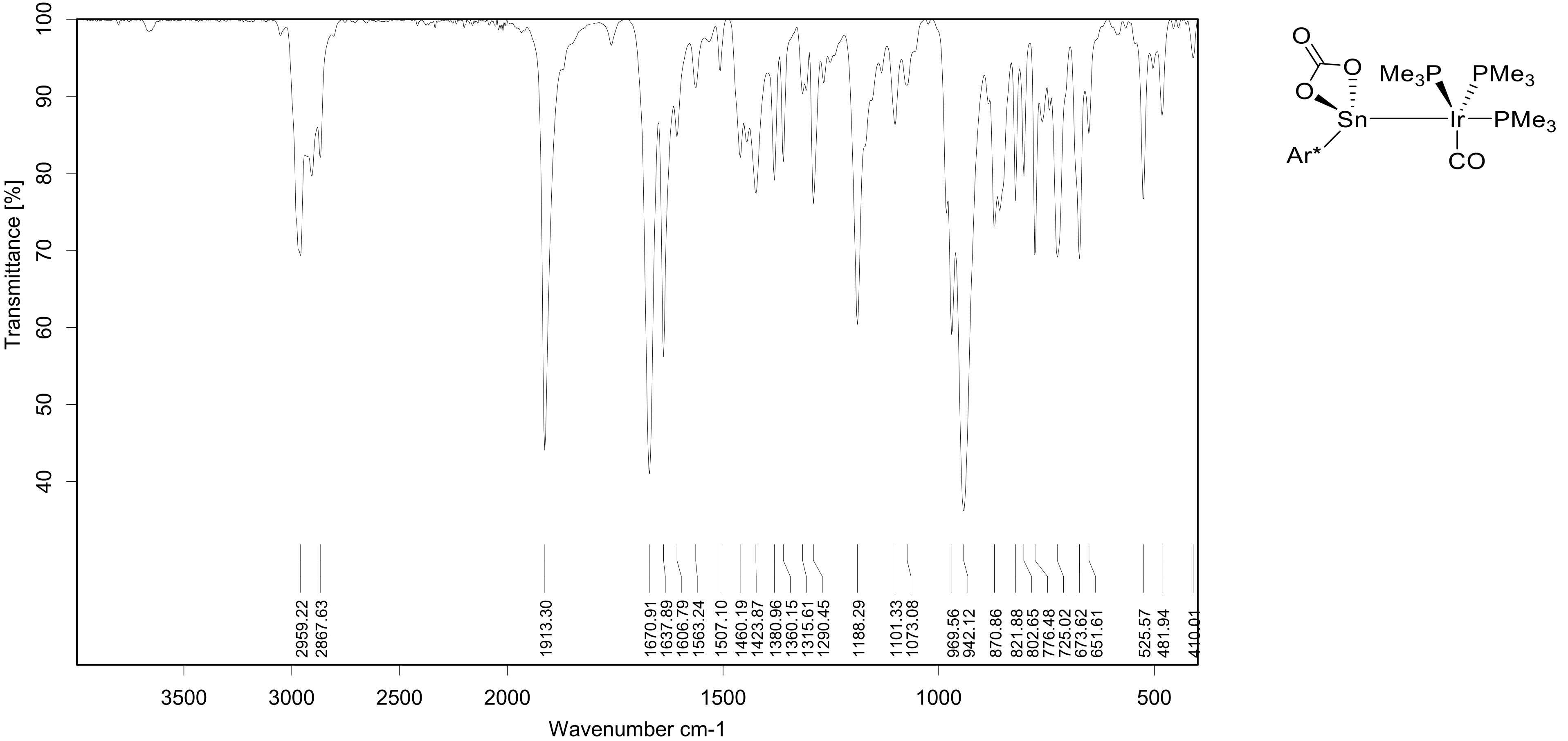

Figure SI80. IR spectrum of compound **16**.

UV-Vis spectroscopy.

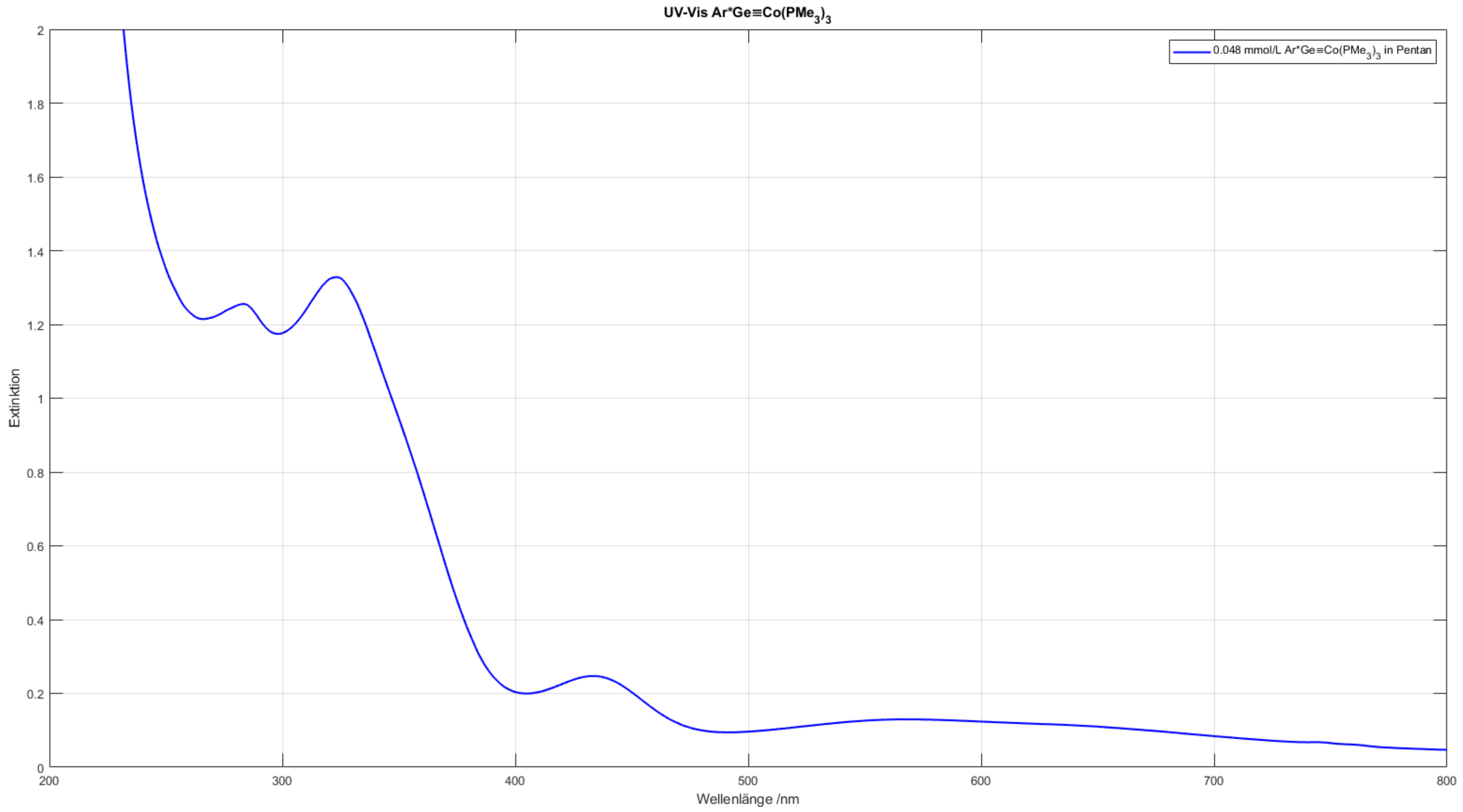

Figure SI81. UV-Vis spectrum of compound [Co≡Ge] (**1**).

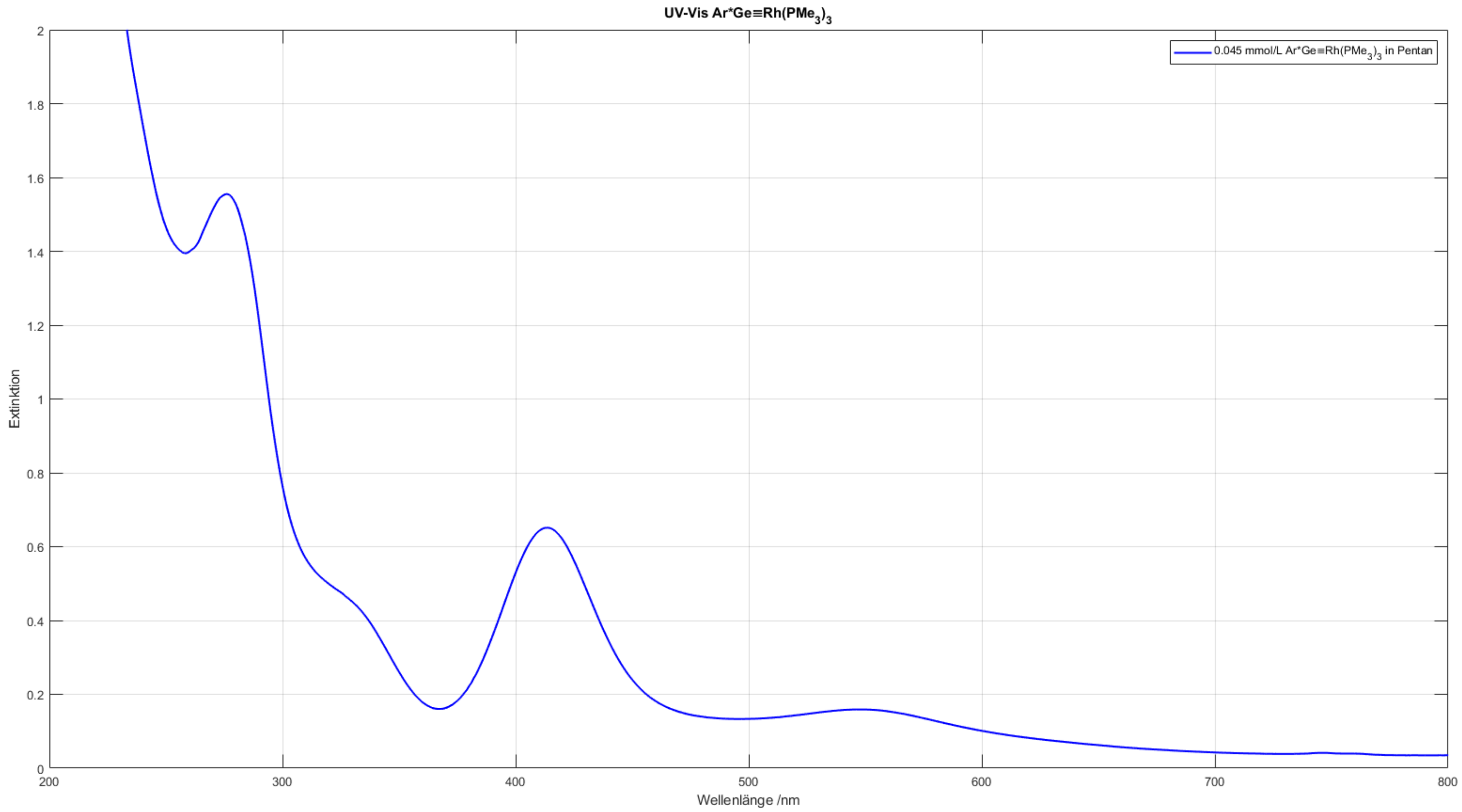

Figure SI82. UV-Vis spectrum of compound [Rh≡Ge] (**2**).

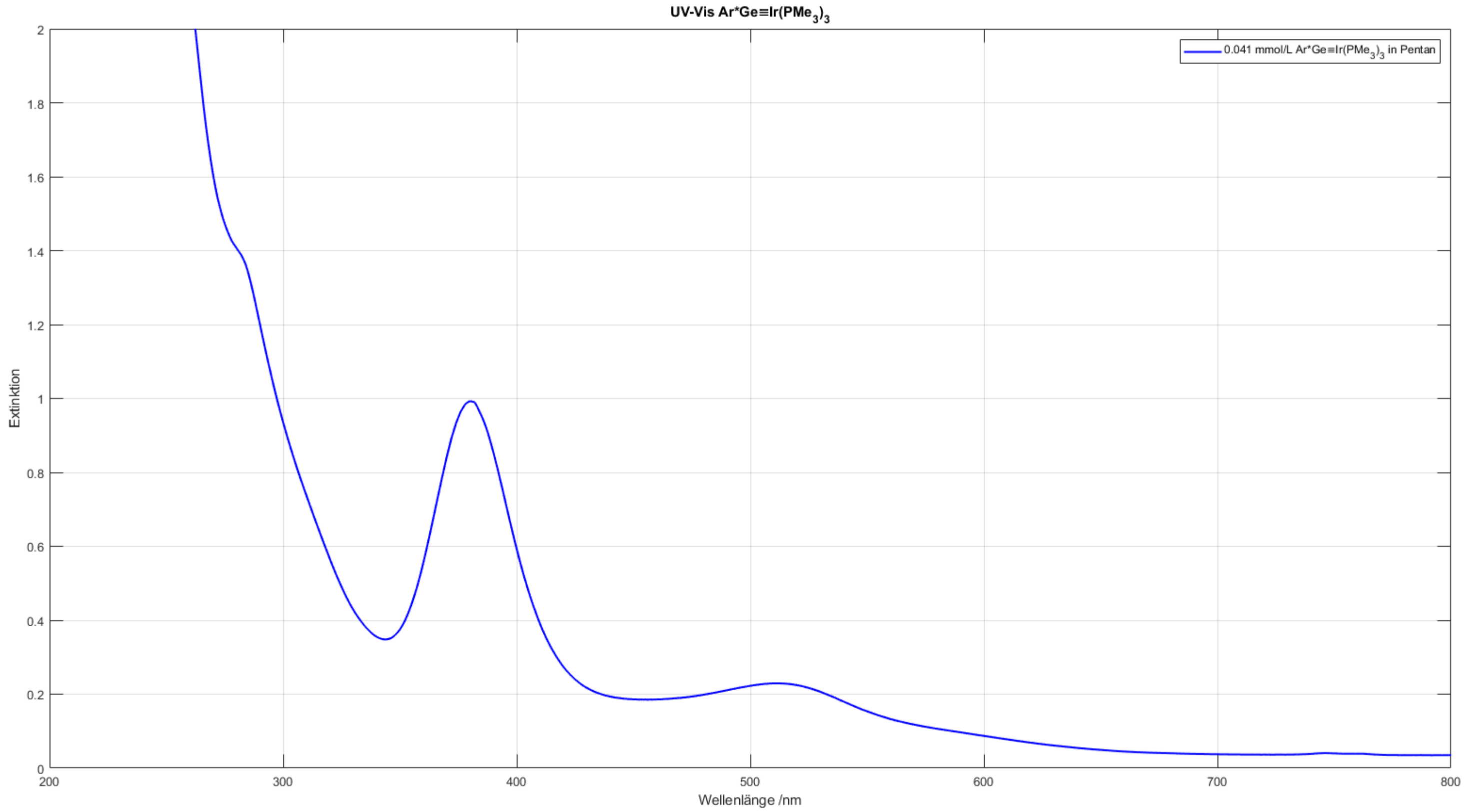

Figure SI83. UV-Vis spectrum of compound [Ir≡Ge] (**3**).

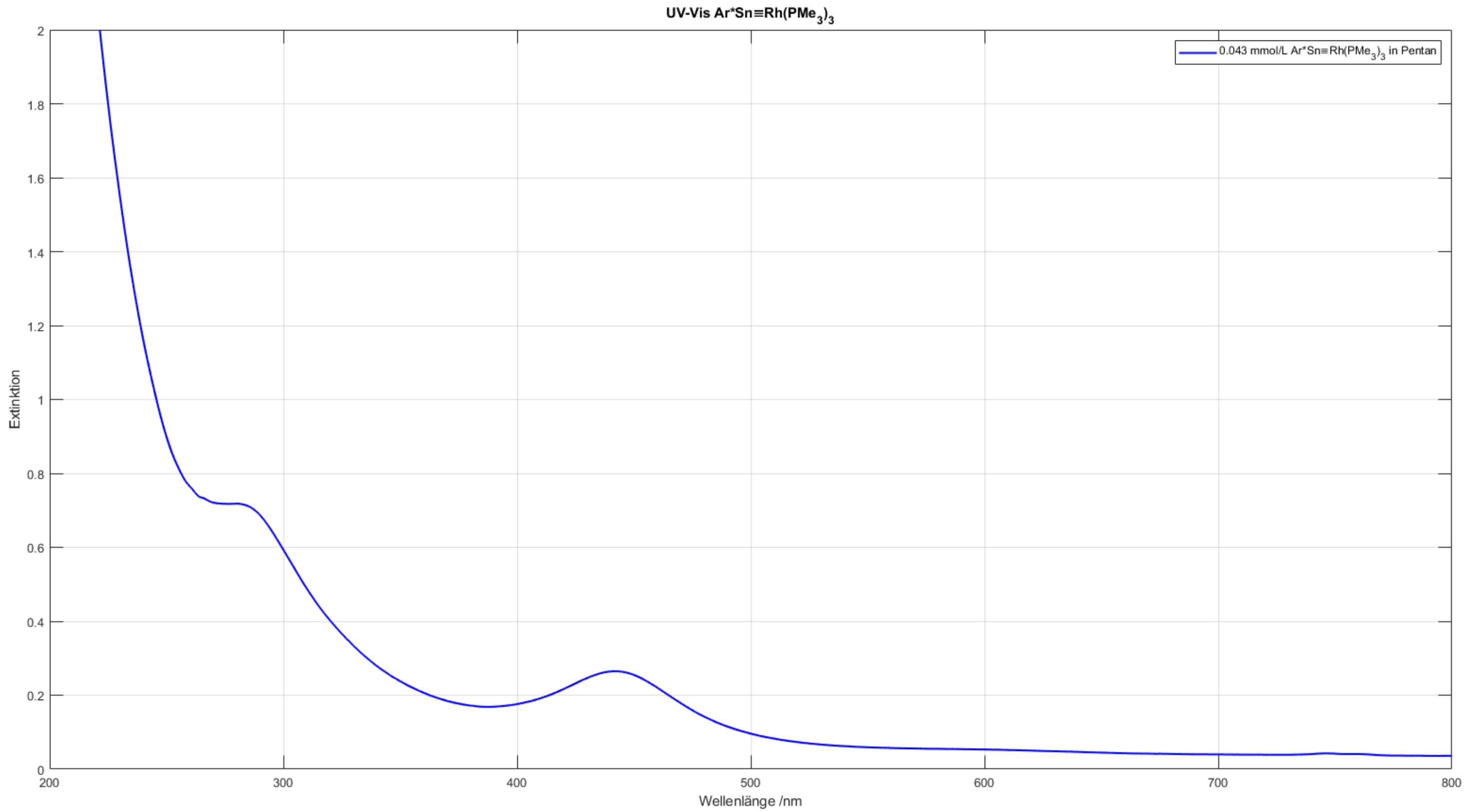

Figure SI84. UV-Vis spectrum of compound [Rh≡Sn] (**4**).

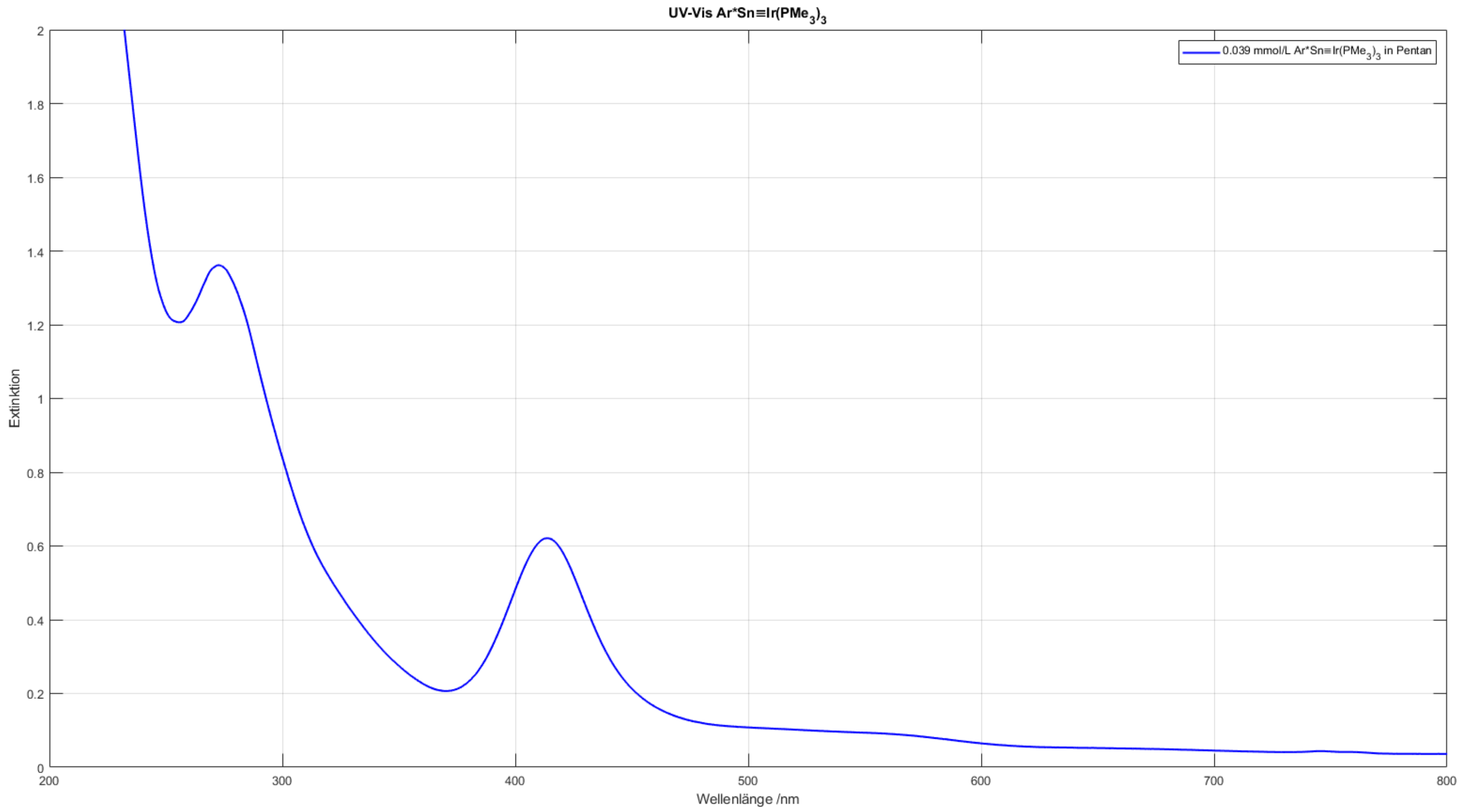

Figure SI85. UV-Vis spectrum of compound [Ir≡Sn] (**5**).

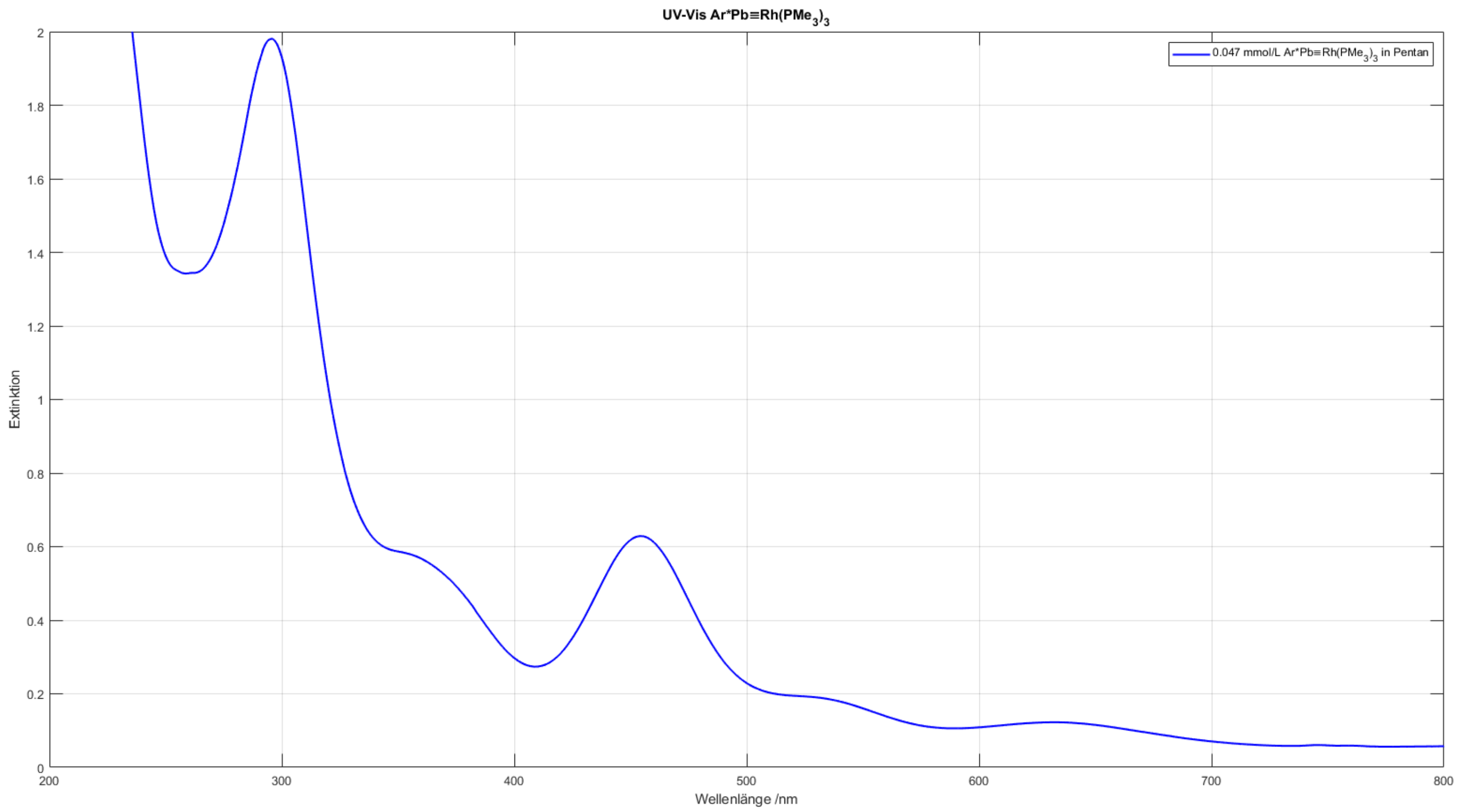

Figure SI86. UV-Vis spectrum of compound [Rh≡Pb] (**6**).

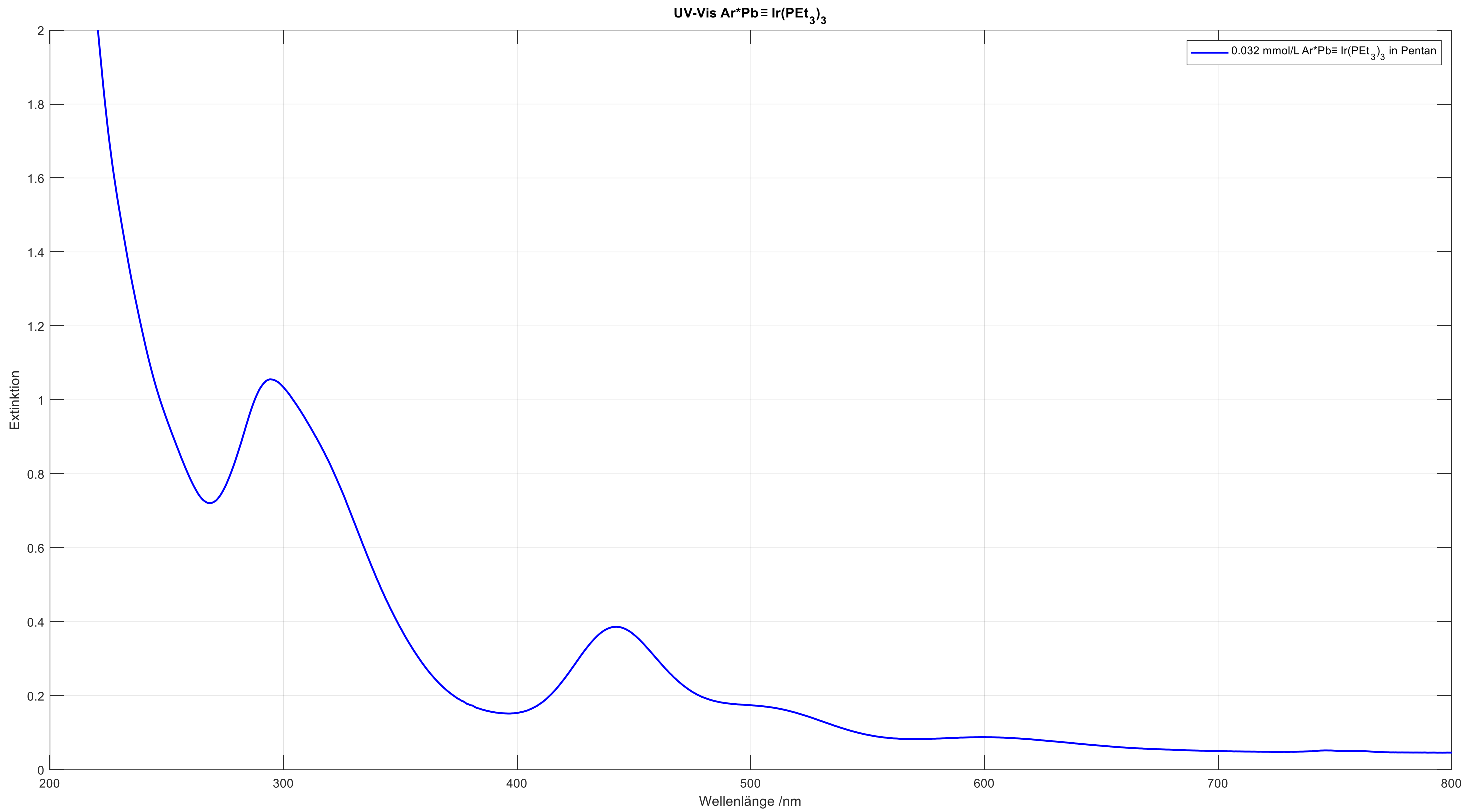

Figure SI87. UV-Vis spectrum of compound [Ir≡Pb] (**7**).

Quantum chemical calculations

On the basis of the molecular structures of compounds **1**–**5**, **7** and **13** determined in the solid state, the structures of the bimetallic complexes were optimized using the programme Orca 5.0.3 and Orca 6.0.1[8] along with BP86,[9] Grimme´s dispersion correction and Becke-Johnson damping (D3BJ) with suitable RI approximations (RI/ RIJCOSX).[10] The basis sets employed were def2-TZVP for Ge, Sn, Pb, Co, Rh and Ir as implemented in ORCA5.0.3, 6.0.1 and def2-SVP for all other elements.[11] Tight or very tight convergence criteria were applied for optimisations and SCF convergence of all calculations. Analyses of the electronic structures were performed using NBO7[12] and respective plots were generated using ChemCraft.[13] Results of DFT calculations and NBO analyses were listed in Table SI3.

Based on the molecular structures of [Ar*Sn≡Co$(PMe_3)_3$] (**1**), Ar*GeCl, and Ar*SnCl obtained from the solid state, Ar* substituents were replaced by Ph-substituents and an initial geometry optimization of reactants and products was performed. The minimum energy path (MEP) was determined using the Nudged Elastic Band (NEB) method as implemented in ORCA.[8a-g, 14] As a result, the existence of an intermediate was identified and optimized. The geometry of the identified intermediate was further optimized by reincluding Trip groups and replacing Co with Rh as well as Ge with Sn and Pb. [8a-g, 10-11, 14]

Bond cleavage energies, which describe the cleavage of a molecule without structural relaxation of the fragments while retaining the spin of the unfragmented molecule,[15] were calculated using the composite DFT method r^2SCAN-3c.[8a, 11c, 11d, 16] The molecules [[$(Me_3P)_3$M≡EAr*] were cleaved into [$(Me_3P)_3M]^-$ anions and $[Ar^*E]^+$ cations (M = Co, Rh, Ir, E = Ge, Sn, Pb). All fragments were treated with a singlet ground state. BCEs were listed in Table SI4.

Calculations of the thermodynamics of the metathesis reactions have been carried out using ORCA (temperature 298.15 K) along with BP86, [9] Grimme's dispersion correction and Becke-Johnson damping (D3BJ) with suitable RI approximations (RI/ RIJCOSX). [8a, 10] The basis sets employed were def2-TZVP for Ge, Sn, Pb, Co, Rh and Ir as implemented in ORCA5.0.3, 6.0.1 and def2-SVP for all other elements.[11] In these computations the tetrylene chlorides were considered in their monomeric form. The structures of the germanium and tin derivatives were optimized using the molecular structures determined in the solid state [Ar*GeCl],[17] [Ar*SnCl].[1b] Due to missing molecular structures of the lead derivative [Ar*PbCl] the DFT optimization was carried out on the basis of the homologous tin structure after replacing the tin atom against a lead atom. Results of thermodynamic calculations were listed in Table SI5.

Table SI3. Selected results of NBO calculations of 1-7 and [Co≡Sn], [Co≡Pb] [BP86-D3(BJ)/def2-SVP (Co, Rh, Ir, Ge, Sn, Pb: def2-TZVP)].

	(**1**) Co≡Ge	(**2**) Rh≡Ge	(**3**) Ir≡Ge	Co≡Sn*	(**4**) Rh≡Sn	(**5**) Ir≡Sn	Co≡Pb*	(**6**) Rh≡Pb	(**7**) Ir≡Pb
M-E [Å]	2.09421	2.20774	2.23079	2.26659	2.39095	2.41429	2.31945	2.45980	2.51246
q [*e*] M, E	–0.45, 0.44	–0.47, 0.36	–0.41, 0.28	–0.63, 0.89	–0.62, 0.70	–0.57, 0.62	–0.64, 0.92	–0.62, 0.73	–0.60, 0.75
Wiberg/Löwdin	0.98/2.15	0.95/2.34	1.01/2.22	0.88/2.06	0.83/2.23	0.88/2.12	0.82/2.05	0.78/2.19	0.74/2.03
σ-bond occ.	1.7258	1.7400	1.6854	1.6866	1.73599	1.6989	1.7498	1.8023	1.7900
M-E % (NLMO)	8, 86	7, 87	9, 85	10, 85	9, 87	8, 85	8, 88	6, 90	5, 90
π-bond occ.	1.6186	1.6485	1.5810	1.7071	1.7131	1.6622	1.7170	1.7154	1.7016
M-E % (NLMO)	82, 13	83, 12	80, 14	85, 9	86, 9	84, 10	86, 9	86, 9	85, 8
π-bond occ.	1.5717	1.5758	1.5296	1.6320	1.6354	1.5879	1.6415	1.6333	1.6029
M-E % (NLMO)	79, 14	79, 14	77, 14	82, 11	82, 11	80, 12	82, 11	82, 11	80, 11

In the case of [Co≡Sn], [Co≡Pb] the [$Me_3P)_3$Co≡SnAr] and [($Me_3P)_3$Co≡PbAr*] derivatives were used.

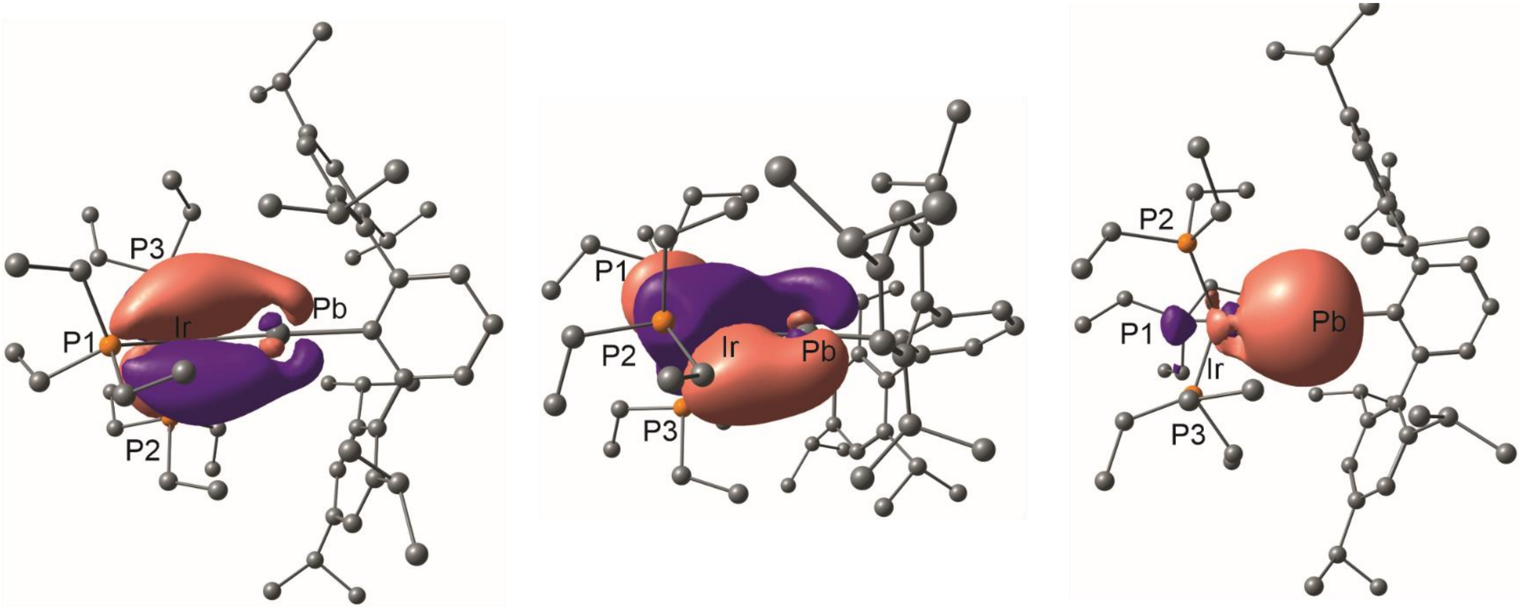

Figure SI88. NLMOs of [($Et_3P)_3$Ir≡PbAr*] (**7**) showing the π- and σ-bonds.

Table SI4. Calculated BCEs [kJ · mol^{-1}] using r^{2}SCAN-3c.

CoGe 991.2	CoSn 894.5	CoPb 839.3
RhGe 928.0	RhSn 835.5	RhPb 771.1
IrGe 991.6	IrSn 879.9	IrPb 807.1

In all cases the same derivative [$(Me_3P)_3$M≡EAr*] was employed in the DFT investigations.

Table SI5. Calculated free enthalpies of metathesis reactions.

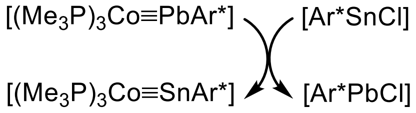

ΔG_R = -21.3 kJ/mol

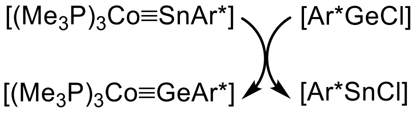

ΔG_R = -42.3 kJ/mol

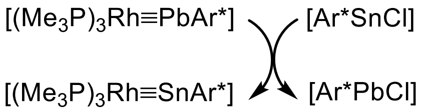

ΔG_R = -25.0 kJ/mol

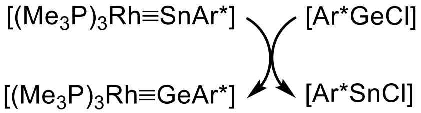

ΔG_R = -43.1 kJ/mol

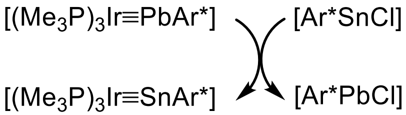

ΔG_R = -32.6 kJ/mol

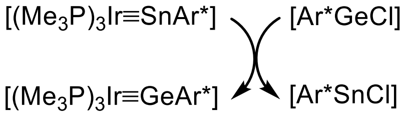

ΔG_R = -49.8 kcal/mol

Optimized structures

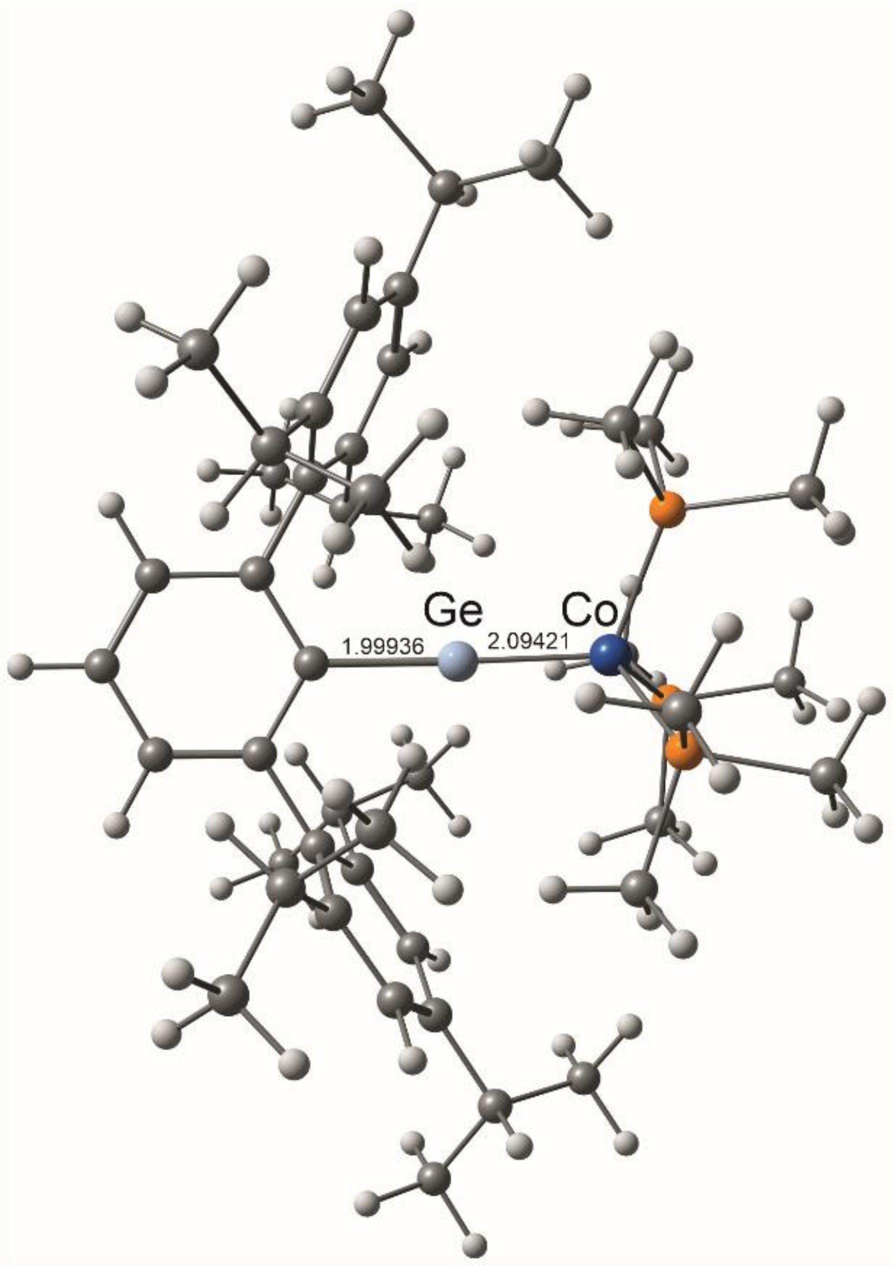

Figure SI89. Optimized structure **1**, distances in Å.

```
126
Coordinates from ORCA-job bp86optfreq_hnumac
 Ge 7.18835456219011    10.52020109030419    5.22165892819067
 Co 5.47454515817964     9.60980317044384    4.43441494419144
 P  3.99692116871414     9.22775103558055    5.91837274850841
 P  4.54592124817390    10.86862562585016    2.99591727267276
 P  5.72294347725516     7.77352400500129    3.38349664858159
 C  9.24874014265616    11.53996921046592    0.91705519067332
 C  9.76709465240205    12.01566806136688    5.19115278596319
 C  10.01920355198542   10.66374610041741    3.08007408668476
 C  9.07778868173440    12.93537090104600    2.93776070423050
 C  9.63011339131993    11.87727164428991    3.70652454693789
 C  11.01724470878866   12.73176535413607    7.16006141769667
 H  11.90222236228603   13.21330545396070    7.60384714875481
 C  8.90170571560821    12.74414306664105    1.55527264454521
 H  8.46748831808772    13.55474558368004    0.94813803886451
 C  10.01480786320572   12.20348665874950    7.98956790387975
 H  10.10551398092500   12.26953079065415    9.08536447526478
 C  10.89286601560377   12.63831466190004    5.76429716630691
 H  11.67662058524686   13.04392410748665    5.10517152209989
 C  8.74593315883854    11.48417637240664    6.02299701175300
 C  6.85326358323143    11.87246662936581    8.91845180609550
 C  7.82376521877266    11.01528832645270    8.33123116066800
 C  5.91852714790808     9.95811236696722   10.14866963467446
```

C	9.07348769171246	11.38587956802395	-0.58852272972657
H	8.42944151086559	12.22880383190136	-0.92397583919901
C	8.87841270637057	11.58338288841910	7.43233027293239
C	6.86319251804854	9.12632422484475	9.52584714455686
H	6.86635971289008	8.05277367238312	9.77494386128709
C	7.81673011185799	9.62553749745689	8.61770380005173
C	5.92016787774381	11.32676421347178	9.81746090338463
H	5.17092156041432	11.99134686783346	10.27510358226399
C	8.62770400119524	14.23077403347363	3.60839978081282
H	9.12000016827622	14.26721262382044	4.60146999024128
C	9.81145066307203	10.51458082047655	1.69712870659746
H	10.11180919498469	9.57143245443192	1.21350640760302
C	6.77905521274014	13.34380236246714	8.51942526261571
H	7.75611545724562	13.60661936931472	8.06579276898897
C	10.69133505686089	9.54727779110894	3.87445880828847
H	10.71362905381954	9.86739286172580	4.93589482971139
C	8.85751097720850	8.68735030338411	8.01316070805425
H	9.35457271493608	9.23568948053900	7.18712564836933
C	4.93766009099242	9.39392718572062	11.16871856280435
H	5.14742483569320	8.30415753080116	11.24583311778336
C	8.21990901069375	7.43232247995968	7.39876964192150
H	7.73942786662040	6.78803915837976	8.16464864914184
H	8.98600398024697	6.81706547172935	6.88373842238191
H	7.45102222800355	7.72101103409696	6.65387729821412
C	3.47458343120010	9.55278211553499	10.71862284429545
H	3.20202410523798	10.62249793657931	10.60246468046991
H	2.78067181250402	9.10810032420687	11.46180769397460
H	3.30208579622138	9.05678526658675	9.74380279371131
C	3.62407550253238	10.57847162319938	7.11069666362485
H	3.39926613519528	11.50397419706095	6.54906190842698
H	4.52803159731875	10.76298468550354	7.71997829131599
H	2.77180816420913	10.33532338680487	7.77837157191567
C	6.55465177123762	14.28770405547514	9.71094548813349
H	5.55766675988429	14.13857327096075	10.17596952462434
H	6.60424241136772	15.34620667028979	9.38240700839197
H	7.32042400373026	14.13502019705457	10.49833526536048
C	3.74556035757283	12.40548990957588	3.63579246281096
H	4.48614039433651	12.97004977112703	4.23182274135844
H	2.91282670906842	12.12366553159051	4.30943109615976
H	3.34958176388592	13.05133016846174	2.82404650140041
C	10.43122952574998	11.52149670990455	-1.30671029151043
H	10.92235402901970	12.48323475109324	-1.05633686216790
H	10.30886583248239	11.47007104747404	-2.40886552200759
H	11.11994593041301	10.70554235152086	-1.00237578552128
C	8.36666857704529	10.07659427837558	-0.97691450195882
H	8.96876240825064	9.18775788480584	-0.69596052740129
H	8.19921618875707	10.02875524151001	-2.07257389809526
H	7.38392489668178	9.98548861556246	-0.47373752583196
C	9.05482779657828	15.48825599270239	2.83453369917703
H	10.14783730549950	15.49697716830754	2.64797747437122
H	8.79507304681286	16.40297988553911	3.40613925131716
H	8.54398181626569	15.56240838768852	1.85176621349259
C	4.27713781644961	7.83241900255944	7.09200617706275
H	5.21387582419596	8.03163403160563	7.64629598681753
H	4.40461411340575	6.89096003085496	6.52331671931964
H	3.44181754299767	7.70824075007763	7.81306757013019
C	7.10858782880607	14.21442618087094	3.86146885675386
H	6.54379858049099	14.23195941152037	2.90652359293408
H	6.79376949247849	15.09384598458742	4.46030556598115
H	6.80793225278991	13.29835371463657	4.41094697638991
C	2.27512387071096	8.84050910985207	5.36047413385890
H	1.60552781219716	8.60464119286671	6.21386194194114
H	2.28365099670646	7.98581217388959	4.65876366703197
H	1.86589351057821	9.71858135398289	4.82356771374970
C	5.16645393013572	10.01256355691723	12.56100696626490
H	6.21440139921476	9.87318621366490	12.89467779861762
H	4.49715636378046	9.55061326460016	13.31637431511872
H	4.96026886359233	11.10330285472821	12.55230137161353
C	5.70769471014937	13.54442434000074	7.42961582614528
H	5.86232252809229	12.84341597430446	6.58295591055207
H	5.73287900196229	14.58031146437573	7.03283689521990
H	4.69166059458799	13.35461180366859	7.83353129339364
C	6.65122288257600	6.39845186226642	4.19532184321056
H	6.70273441051306	5.49485776495050	3.55257416418245
H	6.15092771475968	6.14013761399730	5.14791171581549
H	7.67488291767933	6.73606287963420	4.43437832137746
C	9.94406827323663	8.34451798338008	9.04934846040333
H	10.43349231883055	9.26348818822251	9.43067158036490
H	10.72734968784634	7.69715189280764	8.60262190904177
H	9.51163624411421	7.80655833735844	9.91912520233315
C	3.16064840411424	10.21475872632613	1.95654860922436
H	2.71446783954331	11.00707682428707	1.32020665704254
H	2.37279188655434	9.77878907421714	2.59875132882735
H	3.54899787361460	9.41404946843689	1.29753073340535
C	6.60236168166749	7.86066364095704	1.76112243832610
H	7.54705385372984	8.41937371456075	1.89835243480770
H	5.97591295967787	8.43188558071547	1.04861823912669
H	6.81329066899501	6.86040813671072	1.32806265818170
C	5.60260354957516	11.55789318346249	1.65495551843780
H	6.03146468545279	10.71862946660104	1.07666866702858
H	6.45327104981375	12.09404681307467	2.10944245739425
H	5.04734252251125	12.23227479381810	0.96917868506531
C	12.15082939893062	9.35218778938727	3.42493481260801
H	12.20744698500038	9.02535795059591	2.36535221288340
H	12.65394661746544	8.57975372450671	4.04311134352298
H	12.72636500908606	10.29552932637587	3.51605953088526
C	4.22320193647881	6.81125739755825	2.88280339396858

H 4.48492208107643 5.93302278873793 2.25673758019144
H 3.52109675438576 7.45774614427871 2.32549259495903
H 3.70737287151348 6.45217973679947 3.79480404126213
C 9.88867338422846 8.23926965203016 3.81512504890567
H 8.84700642064103 8.41921395488630 4.15330216039360
H 10.34100108697684 7.46498984030765 4.46862231525332
H 9.84495703142077 7.82558197175506 2.78572641651413

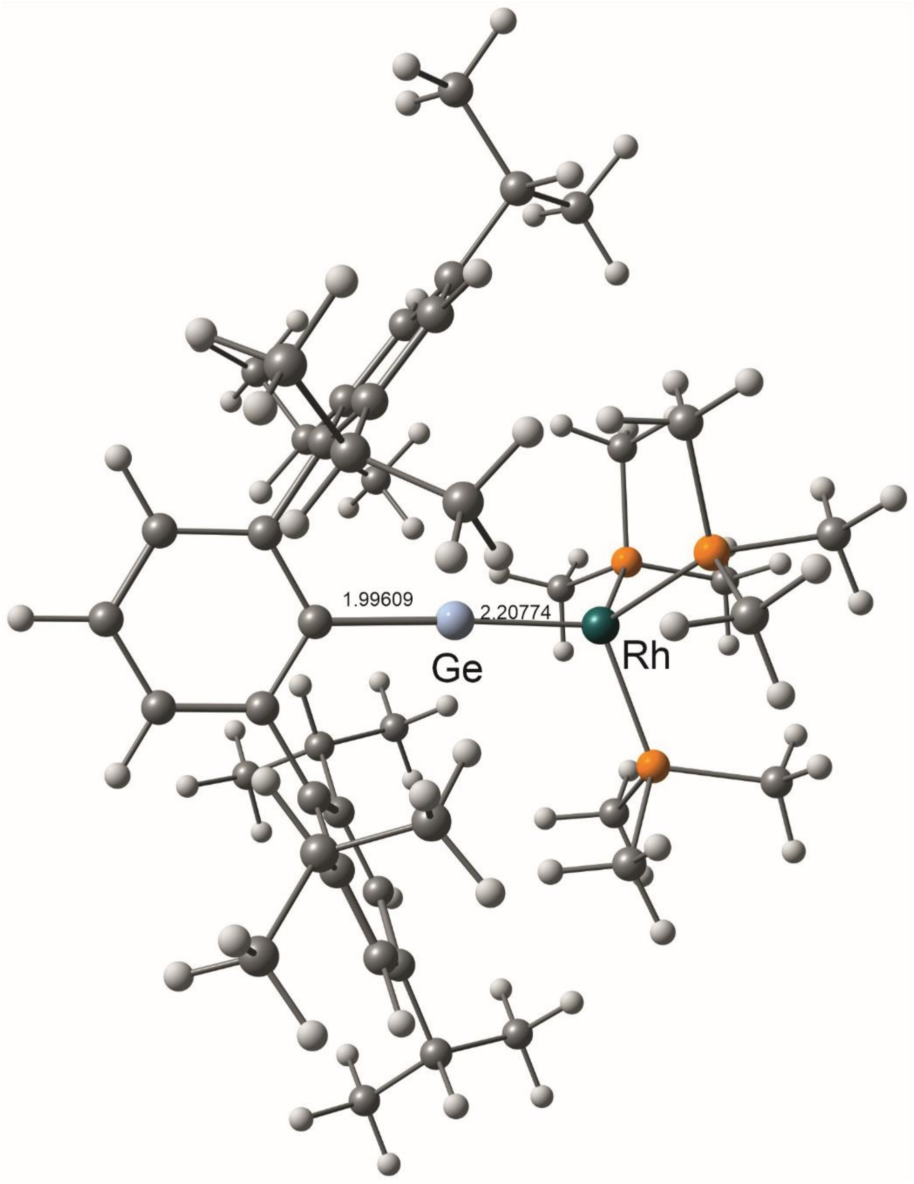

Figure SI90. Optimized structure **2**, distances in Å.

126
Coordinates from ORCA-job bp86optfreq_hnumac
Rh 16.63989931547956 18.59065935143833 22.04242635145808
Ge 14.55612321937700 18.78580747213216 22.74522186570751
P 18.16816208037723 18.67785964190087 23.70530210398518
P 17.17661570536260 16.71630950698026 20.91254134499557
P 17.28485235826335 20.18418201975314 20.57984121792557
C 10.29202171653110 19.06652724699487 23.04641260456055
H 9.45403427820314 19.13959831944972 22.33532869996777
C 10.05132199386990 19.01843318057140 24.43047858738399
H 9.01839587772487 19.05218918991945 24.81008722143677
C 13.09982336540834 19.20475334765922 16.89794921985238
H 13.57589994888368 20.19784872770964 16.73844146594860
C 14.09894593283154 19.99354159951297 26.41843294968022
C 11.12591768835397 18.92930169032035 25.33177683805527
H 10.94149497309667 18.89763248195760 26.41730384983809
C 12.02437837177647 17.85749421791589 20.35262979670137
C 13.60174796878100 21.36528956329789 25.97362654978788
H 12.67594666020407 21.20330381076078 25.38486926858765
C 11.73760982830127 16.50827845878579 21.00518861582662

H 11.37204528154457 16.71301213733970 22.03199822901249
C 12.21879119454122 20.30853188975509 20.47710852886053
C 12.69909610490215 18.92675833180906 23.46352234412176
C 12.15515571930943 21.61553274757809 21.26196624789165
H 11.85506738945839 21.36029383842935 22.29849469135820
C 12.83079800938440 15.44597087707575 26.44939054662292
H 12.43030883057459 14.53387031940244 25.95964032794363
H 11.97282644037722 16.04000634875623 26.82425339001324
H 13.42886854249838 15.12589929233785 27.32857282933087
C 12.59628432917728 20.31908761916404 19.12257807968045
H 12.81861608400085 21.28109316705507 18.63162181595179
C 11.08783223450501 22.56713436072368 20.69332815354711
H 11.32740765845696 22.87089181128846 19.65256498096934
H 11.01742157170707 23.49126455038663 21.30396526820956
H 10.08908093094532 22.08571711399058 20.68065542303400
C 13.24616663159550 22.29040121857351 27.14829948959632
H 12.81899800032171 23.24475830122018 26.77703049159619
H 14.13799898716815 22.54659136573920 27.75774177432347
H 12.50175927281800 21.81907207748534 27.82168644425249
C 11.92689287226911 19.06414181986037 21.09445072043253
C 15.67441028482846 18.64844874577743 27.74666045860250
C 11.61129656013090 19.01854751082228 22.55831266611708
C 12.42605631752528 17.91662748122696 19.00587295250670
H 12.51161651234047 16.97830801147999 18.43577197241374
C 14.83433587619149 15.44626781787008 24.86328476730484
H 14.43907789748887 14.57143374320079 24.30709592099412
H 15.52310653516967 15.05752365749472 25.64280394247123
H 15.42043303521069 16.06992901827644 24.15725518332228
C 19.92550186731970 19.02278419712772 23.25205505632306
H 20.27080391562719 18.27939926322748 22.50846072162046
H 20.60140817918159 18.99951099809028 24.13288125351933
H 19.98488331475045 20.02529053394375 22.78457681028991
C 14.11784525992282 18.13022912601407 16.48512363375150
H 14.43538416142717 18.27371090295454 15.43199179798481
H 13.69060255261629 17.10888581103242 16.56195929062583
H 15.02052999993039 18.16227048974993 17.12637511095913
C 12.45245117119155 18.88798365127265 24.85756144635836
C 13.68482731566661 16.26845006887774 25.46680340839461
H 13.02430503738013 16.56651396802537 24.62711369573016
C 15.12085587102008 19.88727243072260 27.38017744867260
H 15.50523202114593 20.80010323463512 27.86332536071641
C 13.60102959774517 18.80878278035440 25.81628255184810
C 16.71658890118951 18.56351649838764 28.85461632705391
H 16.97759817832041 19.60944352548031 29.12956169030153
C 11.83697958279367 19.14492240293498 16.01464303391489
H 12.09302182089865 19.25105205820001 14.93961887061915
H 11.12221368448138 19.94876334578149 16.28246701120890
H 11.31348518557491 18.17483477929807 16.14756792898067
C 13.53790889500736 22.28590524533850 21.34419060786703
H 13.88986758945633 22.62221603956444 20.34634534765688
H 14.29058345012136 21.57621682644142 21.74759900811083
H 13.50898419360847 23.17507657039586 22.00683038833630
C 16.12334471381021 17.88717871630152 30.10635267637554
H 15.20904891390192 18.41162448975443 30.44970042492452
H 16.85468744622637 17.88052928524090 30.94142740166507
H 15.84436962821822 16.83411598863577 29.89240174438561
C 14.63455288261548 22.01168849832695 25.02894589899099
H 14.87827707107464 21.33630196110986 24.18170003766136
H 15.58370535287427 22.22479630776495 25.56417473120312
H 14.25177972728573 22.96720340803573 24.61428974285830
C 16.70536257880696 15.09682099749105 21.65380178384709
H 15.61170836003539 15.07644550927933 21.80732845196531
H 17.18596818038083 15.00087123316452 22.64586639065605
H 17.00667763485077 14.23996163200082 21.01483952841896
C 14.18405406413481 17.55097323578806 26.12617895646036
C 12.71810832797322 19.13710709695981 18.37137531967624
C 16.45414645239136 16.54554526921426 19.22577116249689
H 16.86594757278934 17.34736784199128 18.58223016224493
H 15.36138222364486 16.70400735335067 19.29950512234598
H 16.66644784830317 15.56143213765825 18.75681817737638
C 18.01034786384162 17.86577338941236 28.40290894367691
H 18.45641032731968 18.37894913987336 27.52925973486332
H 17.82376638476384 16.81113432969364 28.11186737547175
H 18.76071194804498 17.85709198925900 29.22033553617952
C 16.16485290583670 20.51856044775461 19.15927627232117
H 16.51427112474386 21.35890915009227 18.52232947294421
H 15.15488793959468 20.73122817437635 19.55189042484746
H 16.08304636139017 19.60101263520860 18.54661350476069
C 18.38594959639434 17.16906076838830 24.73880106031900
H 18.64824560096829 16.31530290301365 24.08411079768982
H 17.41930773303983 16.94426719775079 25.22807418487828
H 19.17264315977010 17.28784405665818 25.51273744402784
C 15.20952065777347 17.49531364824040 27.08772383656326
H 15.64358870100067 16.51592787333911 27.34439623747066
C 17.47230180119595 21.87765248179360 21.28488182738792
H 17.74118786229682 22.63584946706516 20.51895031447618
H 18.25562350296461 21.85583745203192 22.06743009431869
H 16.51950165900080 22.16116443630398 21.77025295476955
C 18.95558470882378 16.36827529431474 20.56023316210323
H 19.09177991663360 15.45622674862342 19.94198799483532
H 19.48741537882168 16.22827744161243 21.52185489663215
H 19.41194011478726 17.23205092106686 20.04223585073497
C 10.63106922355187 15.73525978504009 20.26705261274894
H 9.69977188351836 16.33341456466005 20.20033349632952
H 10.39540210100551 14.78745919111110 20.79406234637436
H 10.93654216622859 15.47277597941397 19.23240424282030
C 13.02509187210604 15.67690520524283 21.14146225099359

H 12.83883255925950 14.74104721737184 21.70816548424508
H 13.80895919649930 16.25965513950614 21.67007891957290
H 13.43348802682535 15.39572321733896 20.14782111210788
C 18.88899355175385 20.02809126124130 19.67746686577862
H 18.84366864052643 19.14388037416522 19.01198199784204
H 19.71297005722207 19.87233828016393 20.39920872482393
H 19.10838677015746 20.92381157845719 19.05931781296000
C 17.91644919139667 19.95098263115225 25.00768984739983
H 17.83112599553472 20.94350038020951 24.52580108260326
H 18.74132205600632 19.97151612988250 25.75178737988773
H 16.95867734643388 19.74128340806464 25.51980519565413

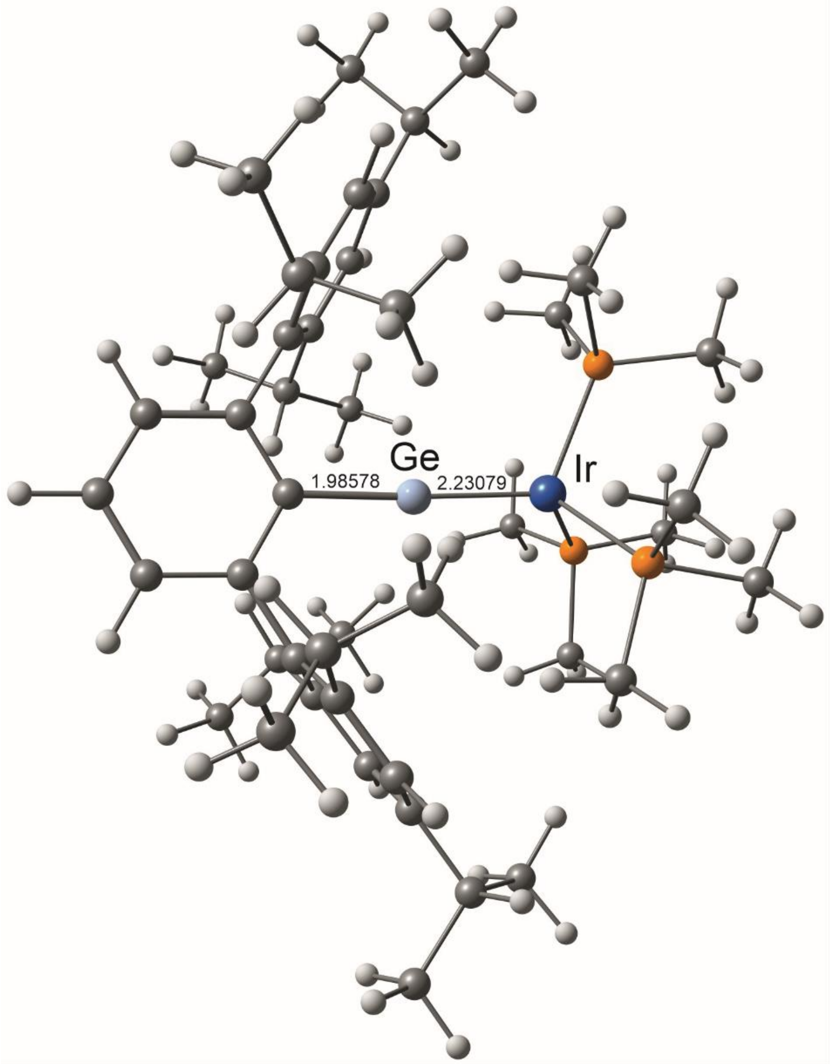

Figure SI91. Optimized structure **3**, distances in Å.

126
Coordinates from ORCA-job bp86optfreq_hnumac
C 2.13554653643643 -2.45883317125680 12.18661593964729
C 1.14771820135173 -2.27533734097278 13.18597562072060
C -0.20383824633510 -2.14423868218337 12.81032164701387
H -0.96731800028076 -2.00095112891579 13.59124592444701
P 7.60829549650198 -1.01985271909956 12.78902978480463
C -0.57398675220746 -2.20074621969993 11.45562835321349
H -1.63271386853444 -2.09841358276362 11.17160280708218
P 7.28864481737548 -4.45109084383179 12.39008287242480
C 0.40413943274428 -2.38271395070730 10.46257613073962
H 0.12027111667609 -2.42397253135910 9.39923396832108
P 6.45503976192222 -2.96814983634656 15.53148784795608
C 1.75961355172836 -2.50852283830912 10.82031031223076
C 2.84477583873251 -2.67961282621350 9.80223468873920
C 3.22870869717670 -3.98136796463055 9.38673347822585
C 4.30809785323122 -4.11553016725591 8.49604320735575
H 4.61712044765301 -5.12276326993660 8.17039425341095
C 5.03006749649550 -3.00498692435157 8.02327134314686
Ir 6.19304954414781 -2.70270881626931 13.30219425017973
Ge 4.06015347738558 -2.54314409366108 12.66838374036909
C 4.63190969252775 -1.72677649185994 8.45043067547463
H 5.18183365680635 -0.84227145473355 8.09393861879327
C 3.55031187021948 -1.54164615754721 9.33062963130635
C 3.16016907977352 -0.13299382809772 9.76841032186901

H	2.35179432596617	-0.23542732506192	10.52082791938977
C	2.59039645825345	0.67791485916963	8.59056525571293
H	3.34875945956327	0.81542858519227	7.79120686500553
H	2.26529265663144	1.68539851313653	8.92421732439680
H	1.71674195602310	0.16579696512554	8.13890120555437
C	4.32963588362919	0.58990440385043	10.45766801760365
H	5.16547406431602	0.78769442379287	9.75382879524677
H	4.72325686845509	-0.03191600066590	11.28881109780923
H	4.00338706638884	1.56670302107221	10.87092533134918
C	6.17673022216255	-3.20653515576157	7.04044776161341
H	6.54050121425193	-4.24805760189909	7.18773586125795
C	7.36278499717141	-2.26099947729570	7.28481654006262
H	8.21454174646711	-2.52103089617477	6.62363137590551
H	7.71138791996165	-2.31185979670326	8.33546084714465
H	7.09608382324235	-1.20482795943821	7.07262218118194
C	5.66412709926488	-3.09288497465224	5.59010166793700
H	6.47562489062732	-3.29403672526712	4.85964441619684
H	5.27222188207572	-2.07252500662034	5.39513442368475
H	4.83964485822145	-3.80873023525315	5.39891802518560
C	2.53618331397902	-5.22218855587235	9.94204229941342
H	1.68313412961993	-4.87206391683718	10.55796066270637
C	1.96699715373018	-6.11792800908313	8.82880345274142
H	1.26482090259380	-5.55524166582817	8.18084109986521
H	1.42080200177778	-6.98222845996266	9.26035968828801
H	2.77096595511263	-6.52420485275033	8.17974812266863
C	3.47997350220375	-6.00057474309819	10.87762914279531
H	4.33782922791005	-6.43146761665964	10.31963239671520
H	2.94694533708468	-6.83664969055969	11.37550272599441
H	3.89077119525156	-5.33286306695204	11.66396035331644
C	1.56551258967026	-2.21407583921790	14.62245035896777
C	1.47703778347731	-3.37071714891117	15.43834808653001
C	1.87257660299110	-3.28781738018615	16.78793560314686
H	1.80512041727086	-4.18915077234209	17.41423078540135
C	2.36303200032871	-2.09455838341528	17.34421063330949
C	2.47160581683759	-0.97019235332628	16.50461065887160
H	2.86806827987971	-0.03245060582876	16.92657994901419
C	2.09058400369031	-1.00432533503314	15.15291059272047
C	2.20838538013061	0.24929443449590	14.29103318112430
H	2.15127107402115	-0.07472960504083	13.23166738098104
C	1.01599671294766	1.19117781418863	14.54309336359168
H	1.07249065032564	2.08693182653852	13.88988138858847
H	0.99926972770597	1.53695529924748	15.59819848703651
H	0.05400401261312	0.67829337370934	14.34091238981322
C	3.55949941969018	0.95721089900887	14.47071465786274
H	4.38895624208066	0.23990404259375	14.30529456439938
H	3.67164645709403	1.39820492258749	15.48348644378925
H	3.66526967438797	1.78475272027734	13.73951199038095
C	1.05518596116682	-4.71172682195873	14.84549046185928
H	0.58629860349803	-4.50164381401827	13.86280589793647
C	2.29831505987157	-5.58309731983456	14.57745040628974
H	2.02347841293242	-6.51299958857586	14.03772783367421
H	2.79330185712757	-5.87195446343040	15.52823285592015
H	3.04772186822008	-5.03446121804082	13.96934639064430
C	0.01720230962648	-5.45202083520221	15.70393173246848
H	-0.87499830727114	-4.82112328217422	15.89296903282639
H	0.43311966157258	-5.75172186448155	16.68869090922129
H	-0.31805182424515	-6.37964328795550	15.19580426490102
C	2.74279405501103	-1.96406452555833	18.81521433282506
H	3.62867621777185	-1.29010895372226	18.85218028358806
C	1.60369634433209	-1.27514505140551	19.59586082979846
H	1.34501703525565	-0.29344185569028	19.15106326354839
H	1.88632223168919	-1.11124299926410	20.65679726686012
H	0.68773655702652	-1.90215418439606	19.57624926414533
C	3.13821787685736	-3.28875443403284	19.48094161123562
H	2.27282659029202	-3.97915868902194	19.56242563435182
H	3.50977104126977	-3.11061866868542	20.51045635886773
H	3.93377180941589	-3.80949926839074	18.91217609417817
C	9.12240887806227	-4.57996494005114	12.55581162072878
H	9.51236096816535	-5.52702334868221	12.12767151732412
H	9.40688973521785	-4.51688040417277	13.62316267611692
H	9.59381186206671	-3.73113339826176	12.02326377299075
C	7.11847610062874	-4.68714798562533	10.57562024328800
H	6.04285121149401	-4.73082460440125	10.32912432733976
H	7.63565603666590	-5.59983437580486	10.21018514964545
H	7.52833875889359	-3.79799352547450	10.06116859098047
C	6.78744185604928	-6.11102347087299	13.01251304271596
H	5.69406197559215	-6.21665858939379	12.88365307912421
H	7.00832979143334	-6.16989382108555	14.09604305322412
H	7.30886831789531	-6.93938386940769	12.48776613340741
C	8.09693773941108	-3.52809292359653	16.16214512846393
H	8.31732104047005	-4.52894380116717	15.74184389214078
H	8.12117371595173	-3.59239893231730	17.27034858562751
H	8.88652613698845	-2.83335237355603	15.81918433881976
C	6.15237943333350	-1.50970253078491	16.61236553379129
H	6.85939907337078	-0.70101006172772	16.34322862908806
H	6.26250476087598	-1.74909108922448	17.69144004651456
H	5.12439747236427	-1.15092453838229	16.41462429203040
C	5.36311160708449	-4.21093122743319	16.32884068985008
H	4.31655002976905	-3.87533353776342	16.20717248852793
H	5.59286551682454	-4.34342805073797	17.40770516511290
H	5.47322017419715	-5.17883274296311	15.80518649613419
C	8.04934057228996	-0.83983597437827	11.00870559462805
H	7.11166004624525	-0.80086585527610	10.42271980125294
H	8.60990628479191	-1.73954173727012	10.68813533385726
H	8.66398922896745	0.06190206499596	10.80310836572924
C	7.05074096817250	0.69046639435882	13.17928181489316
H	7.80138375372681	1.45280402340074	12.88200006690523

```
H  6.85910970126847    0.76576370779301   14.26641387347371
H  6.09779349223294    0.88501186075604   12.65528021123896
C  9.28337680763197   -0.96872045571390   13.56279026838609
H  9.81170887661380   -1.92280113402983   13.38121267095732
H  9.16498609721818   -0.84593483431905   14.65714904771959
H  9.89750595800566   -0.12969189987465   13.17400194308188
```

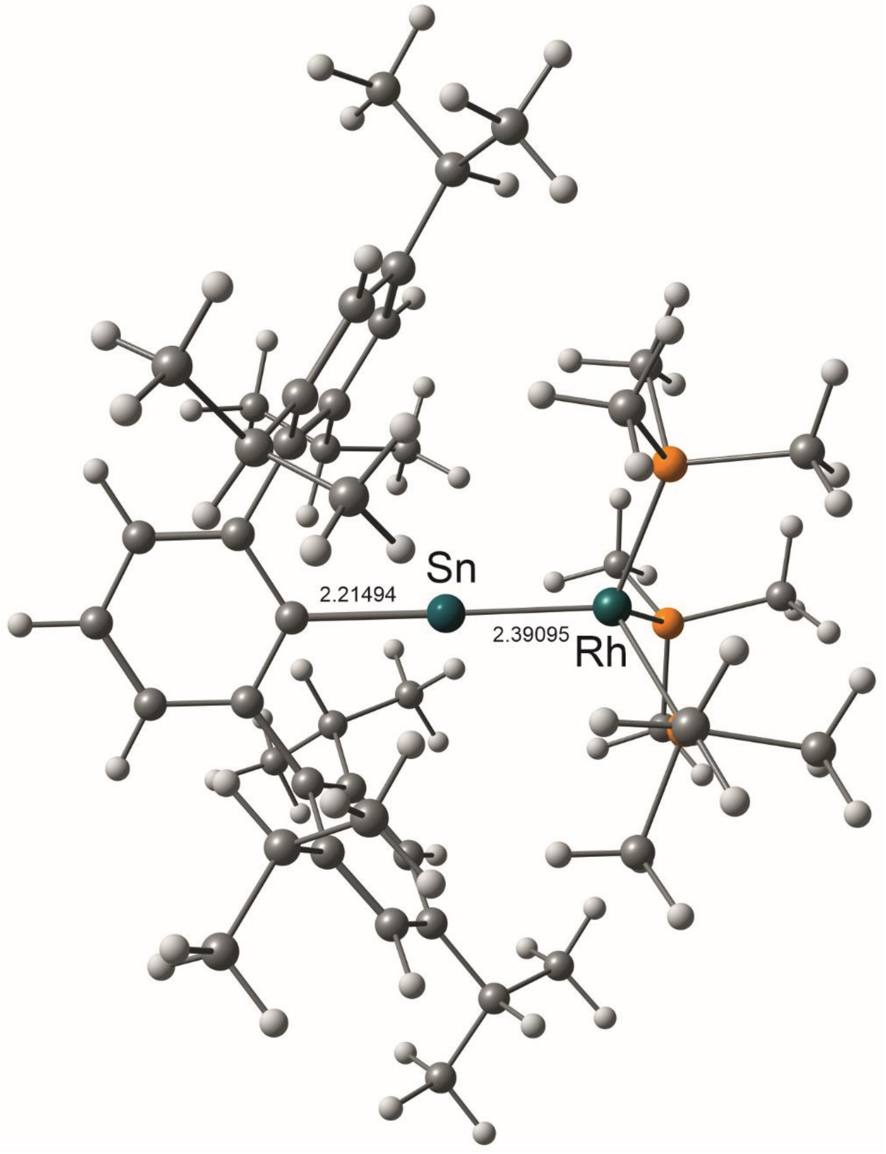

Figure SI92. Optimized structure **4**, distances in Å.

```
126
Coordinates from ORCA-job bp86optfreq_hnumac
 Sn 5.98421434889607    8.16275497532359   11.27505345685402
 Rh 3.70317447341185    7.91511366549079   10.60262095300556
 P  3.55544890427230    7.57443865894988    8.37667318028001
 P  2.72716413074441    6.17008183066667   11.62717499538137
 P  2.31501841701039    9.61972134868256   11.07172319879938
 C  10.82634775728737    8.51082666426293   12.58307685407281
 H  11.88230571675421    8.61701730250110   12.87622218708451
 C  7.67994611662311    9.79899566820103    7.58951559731643
 H  7.27059730001411   10.74320344064236    7.19580728083899
 C  9.84293121046664    8.31680011359090   13.56973105563259
 H  10.12285461469723    8.27150977617725   14.63429351194271
 C  10.46813309979860    8.57873376608989   11.22486079306983
 H  11.23868497322032    8.73663891296052   10.45340649812525
 C  8.49091878591359    8.18222530179464   13.19804143154648
 C  8.22103032799051    7.47290531415856    7.26341624494014
 H  8.23916060969535    6.57375086167140    6.62974167607193
 C  4.42121335446898    7.43127654749333   18.28865063138704
 H  5.20625542801383    6.66416930173436   18.44352100870529
 H  3.58176058719394    7.21380498406522   18.98177112624127
 H  4.85433924231296    8.41383691342900   18.57079714119622
 C  7.72806438104843    8.67887760571492    6.73796698396944
 C  7.29429672342953    8.83018441126370    5.28404886256222
 H  6.43987690085110    9.54406645273202    5.28200123180565
 C  7.75508771904877   11.28966245378506   15.26003244620716
 H  7.11738624213241   11.41218854535569   16.16080296137409
 H  8.06849646112139   12.29951310896881   14.92223750058257
```

H	8.66313333468390	10.73103037469607	15.56395832239842
C	8.24713009183893	4.57259745244992	15.22436890868876
H	7.42092947287232	4.16110372376061	15.84138015202404
H	8.91965540211520	5.13975633811570	15.89922773351679
H	8.81544666614828	3.71169000052265	14.81534837307826
C	6.99926401044083	6.70488357080842	14.61246052441297
C	6.80583268286445	4.68073781320916	13.12549561900699
H	7.35101693266123	3.83475636437150	12.65839842372761
H	6.42775458104307	5.33709777685684	12.31301041844243
H	5.92163688705943	4.26554822657663	13.65346629215923
C	3.94284861496022	7.45425867605972	16.82210608504692
H	3.53442973599553	6.44578610939699	16.58677842717747
C	5.51071657999850	8.94527610159645	15.44339322936953
H	4.92334040385779	9.82261807712274	15.75070487521538
C	5.88660874180205	6.56512131444391	15.46040844595377
H	5.57952707413728	5.55663102090480	15.78413796040494
C	6.99772500216391	10.54468017153038	14.14498960164531
H	7.69700803273831	10.43505996777832	13.29067414006796
C	6.62109238312472	9.13682758354273	14.59970993366700
C	9.30416635881603	11.89163446965143	9.54720967417884
H	9.32512384037597	12.25032834583953	8.49652837557430
H	10.24472111231186	11.33525000639779	9.73334028640183
H	9.29195320680623	12.77945758626837	10.21334922738298
C	8.12703002262885	8.23592578707317	11.83087460193516
C	8.13394055318859	9.75081660087935	8.91823399792641
C	7.38195200174387	8.00669181186178	14.19239981797937
C	6.81266379974889	7.52716773579803	4.63314854544720
H	6.41095496046392	7.72527525011209	3.61875006140760
H	6.01607190429331	7.04372458882004	5.23143535172541
H	7.64080330838286	6.79699522634595	4.51784194857156
C	8.68715113442485	7.37530624541163	8.58987705222937
C	9.11829911197413	8.44244111920698	10.84274421223774
C	7.71732432632933	5.46484140853656	14.08910785410389
H	8.59066698470422	5.81542697825293	13.50251860597487
C	5.12568232948989	7.66904056240135	15.88579473471184
C	5.78544328896310	11.34366130811654	13.63810487131006
H	5.06652407496401	11.57665622589126	14.45106287943098
H	5.24247598707806	10.77076324078805	12.85637770675958
H	6.10978046352346	12.30877144563880	13.19796178795952
C	9.12653562270857	6.02344143148049	9.14370566562015
H	9.53578846714753	6.20392022632351	10.15831938153787
C	8.43307783759670	9.46856853706605	4.46088670529318
H	9.31973344908448	8.80066929131536	4.44439135504421
H	8.75302260989869	10.43637457072056	4.89627097498660
H	8.11729020653669	9.64766417695974	3.41184947211602
C	8.67282805441036	8.52950691760373	9.41377720182129
C	8.07546997879843	10.99707174550384	9.79807905439417
H	8.13364454902944	10.65297988991995	10.85161193560008
C	3.91212523761194	8.98486236619775	7.24399186881022
H	4.94299476796876	9.33096076267807	7.45230490358782
H	3.21849037510356	9.81796261122353	7.47093669200364
H	3.81854278100526	8.71217440250085	6.17097289828046
C	2.92940705422474	5.98795296980606	13.44867196343557
H	2.40676105858537	5.09574555435432	13.85577759969838
H	2.54454860418900	6.89954919142779	13.94343589672896
H	4.01046798908527	5.93257677624431	13.67477056031640
C	6.75629382753724	11.77091902497211	9.64730047735624
H	6.65864799641017	12.24469839958398	8.64820519404812
H	6.69507243809132	12.58204113931737	10.40138705428195
H	5.88747739155027	11.09609622824114	9.79494565378788
C	10.24006554961666	5.37661831797221	8.30300382248475
H	9.89448877812156	5.14246377430111	7.27421119137813
H	10.57655710715314	4.42448290293036	8.76304807680722
H	11.11912439426337	6.04706987981806	8.21751619445552
C	0.89260044709498	6.01029066627457	11.48967945517566
H	0.51183735111616	5.08112262128782	11.96368447524308
H	0.60114280931807	6.02249022508766	10.42189873937913
H	0.41737012176205	6.88093469374926	11.98262158703043
C	2.80941214976651	8.47148979148691	16.62641609288591
H	3.11408496825382	9.49105564619076	16.94193497696958
H	1.92559196818710	8.19358374778705	17.23591033349831
H	2.49350911139145	8.53002579298669	15.56551623727783
C	7.91327800407750	5.08724297521286	9.30337681206939
H	7.47277013564968	4.82528148931536	8.31862852685200
H	7.11328754601875	5.56909733958734	9.90397200668309
H	8.20379442418417	4.14291317959777	9.80862775366393
C	0.65600737289436	9.66855956414181	10.26361235165625
H	0.11817978270166	8.72248370621818	10.46342561918166
H	0.79756515652361	9.75487166956395	9.16840096081170
H	0.03877901504670	10.52259762277944	10.61364908165688
C	3.24372251767009	4.49032055125838	11.06571916895962
H	4.33782331336991	4.39872089101666	11.20402578226451
H	3.02966092044154	4.39067313999085	9.98364389242384
H	2.72776038324740	3.67475762082878	11.61615701587887
C	2.91127470324699	11.31759537460787	10.66791280351001
H	2.16217514239195	12.10271264873037	10.90636799593810
H	3.16420372778432	11.36174014193504	9.59135742063698
H	3.84072628595102	11.50730725841583	11.23706700892262
C	4.64961282007220	6.28684902207626	7.64404103574237
H	4.43415677833376	6.10273661880816	6.56948855784044
H	4.52973996632695	5.34395348414885	8.21019663461255
H	5.69759414452623	6.62177988766640	7.76320915031659
C	1.92187332904136	7.01436026221877	7.72172351866825
H	1.13703581198449	7.73851429916535	8.01163248675452
H	1.66974207625112	6.03908663109839	8.18282078638359
H	1.92445828709528	6.89916787218950	6.61706710828049
C	1.83593551329490	9.85362168452499	12.83885333675321

H 1.19628413198765 10.74698182227662 13.00230511100926
H 2.76263952256788 9.94168002908809 13.43794026689568
H 1.29397590756224 8.95276640747985 13.18751124604803

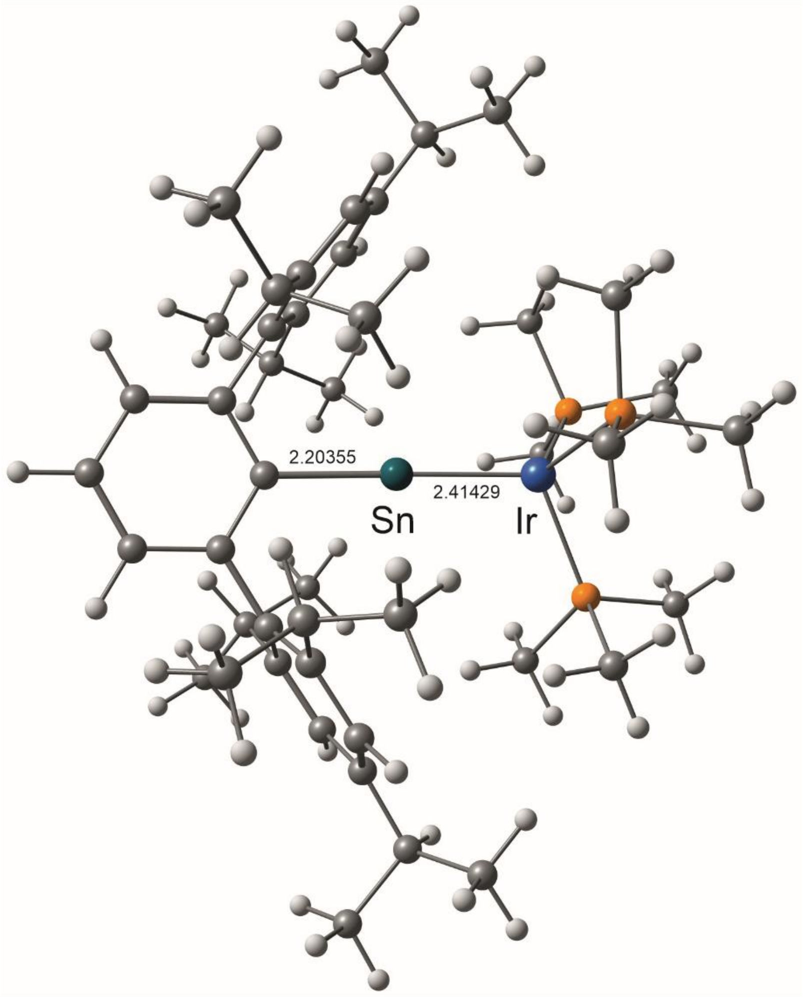

Figure SI93. Optimized structure **5**, distances in Å.

126
Coordinates from ORCA-job bp86optfreq_hnumac
Ir 3.22679944578539 18.52576138326069 9.82886848064965
Sn 5.49167489846836 18.78270914186036 10.62455295360748
P 1.72301641255946 18.56745865218913 11.50961751343917
P 2.82193475642594 16.61224483446589 8.72677636100886
P 2.62031208435459 20.09334695163005 8.32769120885969
C 9.95165929183349 19.04344923257894 10.91891966047780
H 10.77805776847591 19.12404647344884 10.19503457335979
C 8.47909088843256 16.51053940725078 8.87436928209416
H 8.82166666242036 16.70167854399956 9.91159744343526
C 8.62220000504373 19.00582702205397 10.45567148546206
C 7.69975265642165 17.92891320073483 6.91910156605840
H 7.61105678973785 16.99439839131885 6.34398386258828
C 7.07593074409040 22.20279969674733 15.15837062449358
H 7.57293870566610 23.14698070191917 14.85391241841208
H 7.74920971900399 21.67024596197356 15.86028850664920
H 6.15814981495116 22.48096835951451 15.71755971713893
C 6.75619636496681 21.33854382484332 13.92767989317436
H 7.71055031279359 21.14878372507365 13.39591764516651
C 10.21738736258897 18.97072894129533 12.29808706471773
H 11.25785134631185 18.99446467847623 12.65734214604465
C 8.15628885590264 17.86468574395279 8.24915977024585
C 4.41187832537311 18.68878312471170 15.44364646336972
C 3.80417072455425 18.73285111504178 17.88110400976360
H 4.40101388534730 17.82354396071020 18.10352857635690
H 2.97860763340520 18.79214107663814 18.62084732690559
H 4.46541703783445 19.60998131133072 18.02914523625963
C 9.16159285413242 18.87161063322463 13.22120295583253
H 9.36981287413826 18.82470397848329 14.30198993992622
C 3.37644519032532 15.03972536648870 9.50968485928114
H 3.10028574135657 14.14841701874908 8.90702554496233
H 2.92554700423778 14.95939999313198 10.51667575934011
H 4.47399578848818 15.07765385270678 9.63562890719021
C 7.55685445565283 18.90597503485981 11.38315661816261
C 7.93113684094308 21.60395331292336 9.21883993403063

H	8.10172522570201	21.32876447593413	10.27974868546281
C	6.16445002244561	19.98072684974133	14.29441212073199
C	5.82461010858105	22.07169972633721	12.94311404212868
H	6.27237189692705	23.03012765179574	12.60748331211439
H	4.84290075978950	22.29476046415219	13.41133947966905
H	5.62394884014772	21.45121651861789	12.04472319378109
C	5.37396444280731	15.37536555711322	12.78075053833216
H	4.73111356410460	15.95780341983252	12.08796458900390
H	4.73319249422507	14.96729396319361	13.59034990025666
H	5.78551422229230	14.50988680985351	12.22211506528832
C	4.89677222258357	17.52335519395793	14.82671419831902
H	4.40008443328247	16.56225864857102	15.02166151262474
C	6.02841439173658	18.05786075102080	4.40898189772343
H	5.64842999795824	18.22327376828386	3.38018804485473
H	5.15893981085043	17.92441813741425	5.08232367192449
H	6.59252549864778	17.10208057239083	4.39768271648470
C	8.26053798154692	19.06448525871660	9.00165720938933
C	7.45092130754607	20.32048729386350	7.08114046773285
H	7.16684617295465	21.27947178239073	6.61766893537133
C	7.88231450224811	20.30509652542471	8.41897002881617
C	5.05172184885507	19.90714445924937	15.14884653554436
H	4.65330364164202	20.83345669099904	15.59451713135935
C	9.11781263666962	22.47860314503400	8.77426919280789
H	9.01831602995646	22.77727784278846	7.70938979717637
H	10.07632479218399	21.93180489750403	8.88148931505916
H	9.17934612013843	23.40474712748881	9.38301696328144
C	3.25700633742818	18.67736447929479	16.43916607490808
H	2.68128573009421	19.61445217896546	16.26409843632472
C	-0.02984912748722	18.95222333919432	11.07743842868755
H	-0.07441167104267	19.96604853835322	10.63371482852371
H	-0.70310132910396	18.91507748901387	11.95990210017636
H	-0.38511831395466	18.23133253118162	10.31618896203687
C	6.66471802718852	18.78264123655739	13.71600302039619
C	6.51166068873936	16.25287028098127	13.32634025756531
H	7.14619532353123	16.53880732752738	12.46180868827933
C	6.91002609359253	19.23294793390721	4.85643392189167
H	6.30525252550614	20.16244611196561	4.76214789383561
C	2.29107097915502	17.49889738773590	16.26460537834594
H	1.91692072317405	17.42971048592825	15.22503898536214
H	1.41794718146699	17.60837995323969	16.93938064248099
H	2.77596970357543	16.53214522046811	16.51469838727303
C	8.14217425564502	19.38437056593968	3.94067767442629
H	7.84105215545511	19.50836500744188	2.87934689071584
H	8.79131635417893	18.48632159972311	4.01058888580533
H	8.75416964635581	20.26134494492185	4.23251551063290
C	6.00670388286781	17.54723425761628	13.95918318469814
C	7.82587971648282	18.84631838828378	12.77061251695992
C	7.34695221478435	19.14696238635186	6.31350636028163
C	3.78232000418069	20.44331731675778	6.94246269097458
H	3.94292016781769	19.51251667095558	6.36670180554417
H	4.75978693231399	20.72655577127457	7.37460027143466
H	3.41659313592750	21.24354127108614	6.26392115123494
C	6.59762650390712	22.36893862604269	9.15705321645530
H	6.37603416896230	22.73718218515548	8.13353039550717
H	6.61857639420253	23.25018787245340	9.82997908750195
H	5.75605217261971	21.71542581892066	9.47127050535116
C	7.41085461913277	15.48664256661407	14.31511071350204
H	7.81793044916898	14.56454968492987	13.85007351073630
H	6.84133288676903	15.19121273598280	15.22144138870681
H	8.26523294699083	16.11264841521519	14.64266794813699
C	3.55669580552824	16.42401412715650	7.04728322415305
H	3.14924575037766	17.21559263345616	6.38868380159138
H	3.35179866999029	15.43167895960028	6.59218754974818
H	4.64841836282611	16.58450835679077	7.13187325939477
C	1.48885691017854	17.01007964610817	12.46778211576161
H	1.18296916028918	16.20041827857970	11.77708129101795
H	0.72514667135901	17.10877050038696	13.26827278548383
H	2.46317328754447	16.73095484285515	12.91220784935695
C	2.36533376099313	21.79463328631068	8.99088761677924
H	2.08305175078729	22.52557313502937	8.20327399347569
H	3.30279234260875	22.11762133444556	9.48228070106153
H	1.57409388690982	21.76370020852423	9.76492422699421
C	2.00306110951368	19.76481285159737	12.87978110357572
H	2.07434424921126	20.78339957293473	12.45309069911237
H	2.97757716800952	19.52914167817251	13.34800521328963
H	1.19835986890762	19.73474980443043	13.64577997004786
C	1.06041976557528	16.18496240821741	8.38112528053872
H	0.58073478347473	17.01156847252485	7.82461287084252
H	0.52686559202107	16.07061322940269	9.34491221198201
H	0.96023964834169	15.24386100994728	7.80068563211197
C	7.22000203533555	15.62919451401099	8.96750851816829
H	6.83220231384793	15.36300675640506	7.96169716149698
H	6.40636825773785	16.16241219546925	9.50405949236743
H	7.43377352250589	14.68418260162917	9.50853871076735
C	9.62349701297098	15.79690113172630	8.13365559301129
H	9.88049267514945	14.83983010348696	8.63328538030674
H	10.53558512069341	16.42676175612527	8.10558229558855
H	9.34544057555082	15.56200972751089	7.08471476263880
C	1.05106582193683	19.86340863443032	7.38502910855147
H	0.82548808555649	20.73044690283306	6.72937754483776
H	0.21432474446197	19.71143904450189	8.09304067368321
H	1.13826060233103	18.95588453615445	6.75628979386161

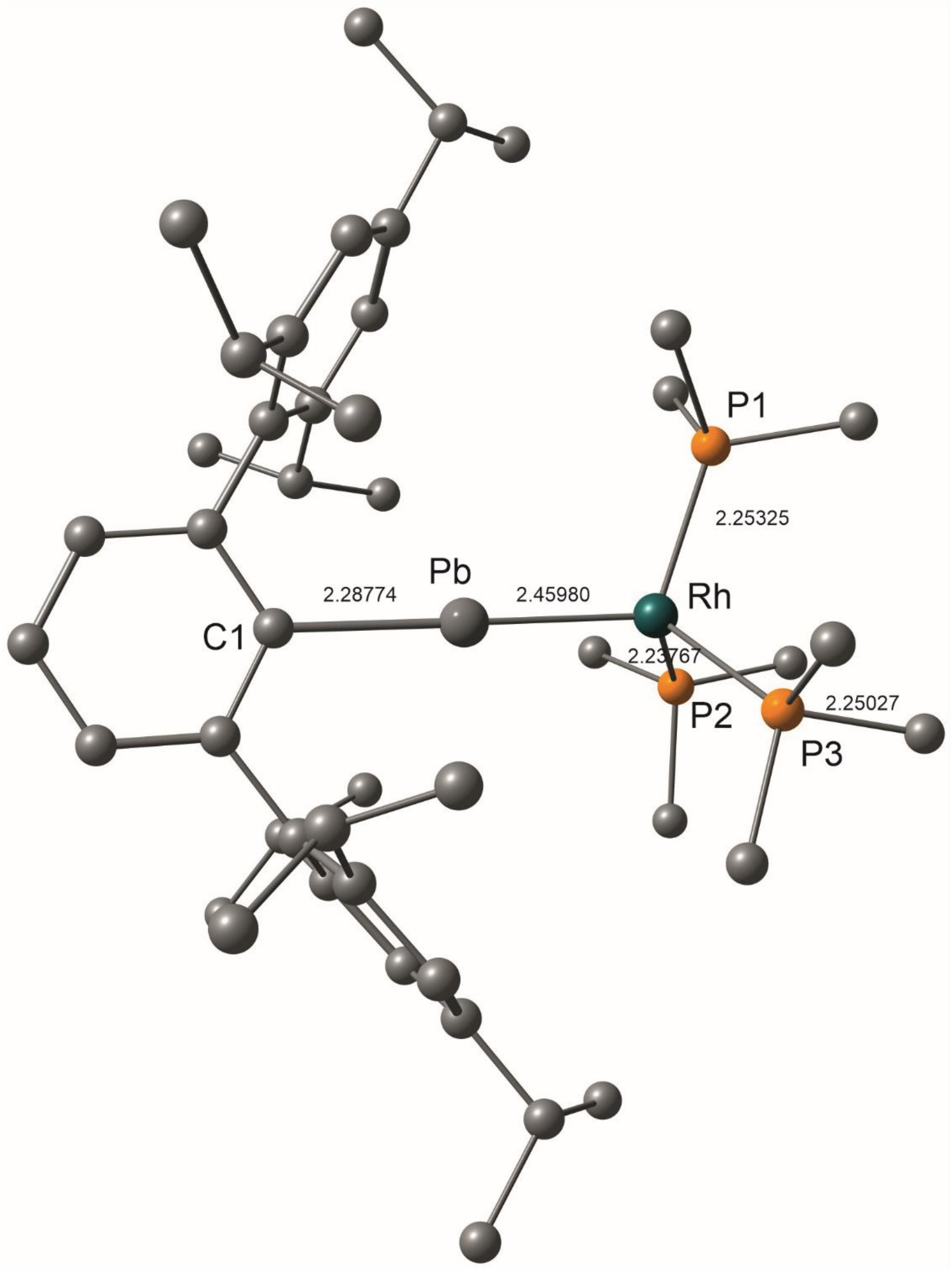

Figure SI94. Optimized structure **6**, distances in Å.

126
Coordinates from ORCA-job bp86optfreq_hnumac

Rh	3.16210021585639	18.51305386553007	9.79941060846171
Pb	5.46113117774654	18.80414809594027	10.62423826936503
P	1.68420077322966	18.55385686058872	11.49978261288523
P	2.79637878342245	16.58685990759407	8.72090579437766
P	2.58188912974876	20.07832399377273	8.29044295308002
C	9.98899232197307	19.07316845484875	10.92010891064058
H	10.80927216109534	19.14949652414151	10.18853531942642
C	8.45277063330071	16.52194975904624	8.93161123357967
H	8.77655311714111	16.72209840198286	9.97309380931586
C	8.65298690869815	19.03622854434517	10.47398182266345
C	7.65981714504407	17.91957685705918	6.96691864711414
H	7.55617866887417	16.97784355039441	6.40650534616653
C	7.00378149416905	22.25305577109599	15.29616778588158
H	7.57258646256324	23.17155807127667	15.04427273444450
H	7.52796874521790	21.74083747418165	16.12833705097239
H	6.01148475136919	22.57958809526014	15.67161055007429
C	6.88297427051782	21.34275908043380	14.06397221538532

H 7.90903757681494 21.14553913406888 13.69199216086432
C 10.26805092847508 19.00172735930208 12.29734549069188
H 11.31241782357027 19.02338342901157 12.64535608260981
C 8.13309634495777 17.87030108538091 8.29242353319960
C 4.43848067299935 18.69779274516733 15.43077734037361
C 3.79763701345651 18.69370320072662 17.86054525221147
H 4.38170615677894 17.77331083849574 18.07092821676057
H 2.96229543199515 18.74540391309794 18.58976389371783
H 4.46566403622215 19.56018174040678 18.03780162990198
C 9.22299924186176 18.90261655986820 13.23339394100814
H 9.44598590872653 18.85087000308776 14.31131535975117
C 3.33851737616569 15.01062260139912 9.51131046854632
H 3.05894894054388 14.11740752023396 8.91276377318338
H 2.88415033258263 14.93639864418154 10.51744759057022
H 4.43624662670303 15.03883751092360 9.64086969308819
C 7.60385481527738 18.94168762816470 11.41390136649133
C 7.93974245753779 21.62139162700510 9.21645229524873
H 8.11908448275683 21.35740791097623 10.27871907886145
C 6.24478241796072 19.98998815799575 14.36728332371482
C 6.11370377376614 22.03921531368821 12.92402497656435
H 6.58636166568809 23.00619277149646 12.65330371041574
H 5.06132882276738 22.23594520877684 13.21744115773125
H 6.08903471051345 21.40572896520380 12.01397600067358
C 5.37427762607986 15.40862456360540 12.73759415855031
H 4.69537059411286 15.98766501371734 12.07684703327076
H 4.76981831339349 14.97669674671178 13.56244567146654
H 5.77907147520786 14.55895783699507 12.15088037491642
C 4.90160527459659 17.54770759133573 14.77087688232802
H 4.37827504332856 16.59283630898043 14.91923437564287
C 6.02268638251292 18.00660756302257 4.44100293216817
H 5.63503066862809 18.16582908366619 3.41418485067592
H 5.15952149605979 17.83980441808259 5.11484758690705
H 6.61875066680800 17.07050286114572 4.42178472409983
C 8.26252182746486 19.07943675446981 9.02639345721718
C 7.42780209053249 20.31394263170010 7.10140338998778
H 7.14143751532455 21.26848359693531 6.63023523398201
C 7.87807924944863 20.31343081428365 8.43309496446401
C 5.11657899916293 19.90973868455130 15.20015875890864
H 4.73810164937780 20.82483167356474 15.68335024293635
C 9.12578338178307 22.48629792972700 8.75162129038947
H 9.01871587294110 22.77266192724785 7.68410106946261
H 10.08279154559934 21.93682066155159 8.85784195927702
H 9.19600313734193 23.41916425078146 9.34900267562606
C 3.26992575154568 18.67566537352929 16.41035315465065
H 2.70531674502538 19.62196587508592 16.24860380173420
C -0.06495858987914 18.97436852543719 11.07965143187300
H -0.09443411325545 19.99519030991198 10.65067239156632
H -0.73853582991875 18.93483692657405 11.96210535202203
H -0.43337165294166 18.27127664365831 10.30769782764121
C 6.71912561682798 18.80592679117932 13.73812800160636
C 6.51729845281142 16.29543544756860 13.25452044859309
H 7.12152127895850 16.59662584912623 12.37349214140380
C 6.86498412900425 19.20768132462424 4.89327316236360
H 6.22938176350100 20.11701950811743 4.80468210091420
C 2.29548798531843 17.51083550967277 16.19781655718348
H 1.93205996870291 17.46909943189172 15.15323029965497
H 1.41571696450863 17.61327698837267 16.86493853449698
H 2.76880004284890 16.53418481043487 16.43117549745498
C 8.08939303943314 19.40497172162580 3.97549770853128
H 7.78153407026418 19.52439916055555 2.91560708281665
H 8.76799183710657 18.52856411158780 4.03955726793736
H 8.67266795260182 20.30026595613474 4.27042813957249
C 6.02516773983715 17.57890440134440 13.92039254730152
C 7.88191878897490 18.87909269473569 12.79550157840326
C 7.31052303244533 19.13190152697319 6.34838813743082
C 3.74806031283355 20.42676668673098 6.90477697046815
H 3.91488728283339 19.49393408376835 6.33353951192293
H 4.72374400171146 20.71864571256742 7.33635965303753
H 3.38270825488556 21.22115481794063 6.21894872378243
C 6.60906300736224 22.39159294324489 9.15568541076789
H 6.38122296070288 22.74911817223332 8.12980663754546
H 6.63773649691861 23.27988873410010 9.81882053062369
H 5.76432835389070 21.74760320262326 9.48158457803466
C 7.45688767628680 15.52857376045344 14.20489084410854
H 7.85517995473271 14.61407463264642 13.71777364622750
H 6.92066002917639 15.22147753579598 15.12744602174292
H 8.31692026165227 16.15917018309224 14.50736965528934

C	3.53781979045793	16.37819880070487	7.04473444981490
H	3.13944224258940	17.16776100854213	6.37804427935177
H	3.32904245621479	15.38416000837707	6.59484028667317
H	4.63079188268755	16.53070271380332	7.13056363916145
C	1.41631210902108	16.98459341195587	12.43429816045860
H	1.09020864923732	16.19453141621634	11.72999144298316
H	0.65714953523157	17.08345605553114	13.23923315094362
H	2.38371927804639	16.67208190675381	12.87195449395612
C	2.32129631336235	21.78928514029412	8.93313628116278
H	2.03181755478955	22.51082304548066	8.13918358473552
H	3.25928255591917	22.12439836363010	9.41603161973138
H	1.53401593051831	21.76483499016721	9.71158801608538
C	1.97750226350847	19.72149214778701	12.89718257156984
H	2.07068989158850	20.74624045337918	12.48903730627853
H	2.94457407652165	19.46100217259439	13.36880311836988
H	1.16923600397863	19.69573260530724	13.65977446402600
C	1.03531854887879	16.16010337562086	8.36577028872289
H	0.56223495762105	16.98449299132418	7.80005007733598
H	0.49359430739917	16.05549007129859	9.32622690249783
H	0.93477711117652	15.21486510973881	7.79175907303450
C	7.19910152069502	15.63171980812101	9.00869385004491
H	6.83285652559324	15.34927250512283	7.99936872674353
H	6.36977132972248	16.16336136082638	9.52245904551484
H	7.40848682149904	14.69529591102456	9.56588290614565
C	9.61483780269995	15.81288108848608	8.21421604516456
H	9.86779096020213	14.85792678735985	8.71995473532050
H	10.52348986503211	16.44808285526821	8.20324426088763
H	9.35763646733865	15.57638701258943	7.16029256418716
C	1.01740171701356	19.84502585050825	7.33832359144878
H	0.79452518004798	20.70761623318140	6.67552396703311
H	0.17647541206812	19.69718160718375	8.04264438905658
H	1.10711724483657	18.93331075025047	6.71576073056212

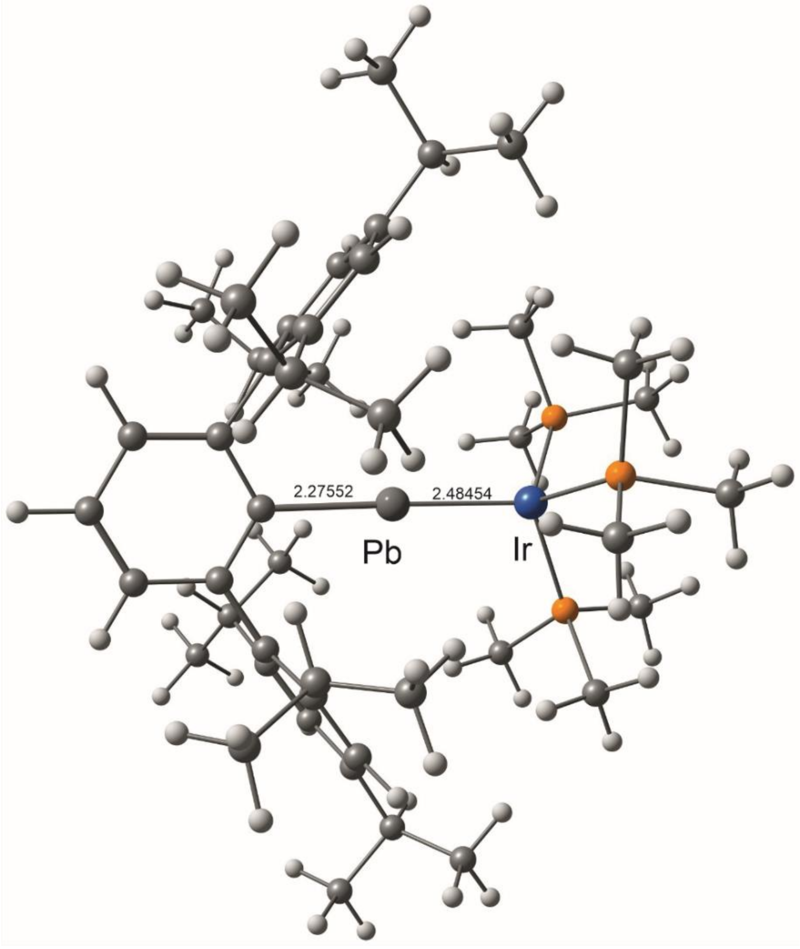

Figure SI95. Optimized structure **7** (PMe_3), distances in Å.

126
Coordinates from ORCA-job bp86optfreq_hnumac
Ir 3.15400072330153 18.51190077011052 9.80211581739861
Pb 5.47708768614071 18.80333446910543 10.63353823826511
P 1.67075946950188 18.55390362034426 11.49589939704149
P 2.77720149213441 16.58736976416697 8.71902509480820
P 2.57831205076367 20.07367521529216 8.28749066464698
C 9.99190561459026 19.07366573011158 10.92025785961240
H 10.81183811907254 19.14985119232091 10.18840781762489
C 8.45756508595753 16.52389435268394 8.93075675242013
H 8.77531000614152 16.72282890805693 9.97438152248007
C 8.65623313683086 19.03841226912710 10.47298831525497
C 7.66268814500525 17.92166768238134 6.96726313100764
H 7.55797441256201 16.97982487251127 6.40729688841759
C 7.01260619306191 22.24816437081200 15.30409625076101
H 7.58245753336161 23.16669986691974 15.05466892648037
H 7.53691125012881 21.73238212267650 16.13400320386989
H 6.02115627520854 22.57480886048796 15.68168512003154
C 6.88888005530956 21.34229121583185 14.06886955491042
H 7.91436895012730 21.14424170010760 13.69570974292224
C 10.27118668626888 19.00058013112307 12.29728438130509
H 11.31561948521581 19.02104267872479 12.64502766181636
C 8.13698730242812 17.87242345306915 8.29227726080503
C 4.43994103413387 18.69806183678415 15.42888530386346
C 3.79083486050755 18.69037259481809 17.85659479389583
H 4.37257031923485 17.76852424578491 18.06707159604346
H 2.95312310803820 18.74211556408121 18.58309743216804
H 4.45980250639665 19.55532472688343 18.03769838237156
C 9.22634224657506 18.90111386071803 13.23339606908174
H 9.44918971838015 18.84780469983540 14.31119170024671
C 3.33021496512613 15.02062886642603 9.51695175040033
H 3.05514080126582 14.12357041188116 8.92221171614754
H 2.87783698346879 14.94949008788611 10.52396678856410
H 4.42786172028778 15.05874780218270 9.64384936821464
C 7.60945431099458 18.94445435097303 11.41526887039996
C 7.94402034085662 21.62377802415172 9.21621451464327
H 8.12357549252717 21.36001139476537 10.27855061591085
C 6.24859537359397 19.98982373664450 14.36923745135474
C 6.12068143673765 22.04429410454389 12.93162536016021
H 6.59645035908594 23.01054303165219 12.66366340652322
H 5.06924928171553 22.24362237753757 13.22659678851145
H 6.09229417691132 21.41360104615589 12.01989517376267
C 5.38087202430493 15.40201169901360 12.74858707878838
H 4.69695635926070 15.97717294981040 12.08965531611154
H 4.78294936353098 14.96922857266357 13.57778575681884
H 5.78674629909056 14.55272733685950 12.16198916002448
C 4.90487751637778 17.54744036806792 14.77114950730095
H 4.38109081741365 16.59275159721724 14.91860254743935
C 6.01950239452298 18.00716757315308 4.44637396973586
H 5.62750728053639 18.16607649861034 3.42114041099732
H 5.15931503560647 17.83963024346928 5.12388365339830
H 6.61623540036755 17.07154276665064 4.42473842328058
C 8.26673208348423 19.08180206105841 9.02551446220241
C 7.43094308832873 20.31618752326400 7.10154848501825
H 7.14352238921326 21.27055546207569 6.63068060823063
C 7.88252741431929 20.31590467738015 8.43267400431096
C 5.11871332264467 19.90980731111414 15.19991522646368
H 4.73958810513349 20.82505323074099 15.68232204386117
C 9.12981430263808 22.48892064986434 8.75117452855914
H 9.02209553075195 22.77534201337313 7.68372879845131
H 10.08703661120208 21.93966344689026 8.85677166904400
H 9.20002312379425 23.42174811832432 9.34862123823429
C 3.26813104326604 18.67611840001896 16.40455611313774
H 2.70567565094857 19.62366416788714 16.24265213646676
C -0.07350604544878 18.98648905933746 11.07446799967211
H -0.09495469269648 20.00706772391199 10.64486918990104
H -0.74596127090491 18.95182817771449 11.95780868140193
H -0.44573734544051 18.28472441419568 10.30339934905153
C 6.72287519686843 18.80564203673595 13.74053992457251
C 6.52288672122984 16.29468763916273 13.25754923368246
H 7.12167739260870 16.59531919641078 12.37256160541273
C 6.86272991731355 19.20886210091356 4.89534410227923
H 6.22648454216046 20.11791660756619 4.80830595256775
C 2.29251235619410 17.51330127538841 16.18621017451361
H 1.93343236816700 17.47366375739599 15.14001688653439
H 1.41021551404743 17.61615847126036 16.84995129423215
H 2.76317339308587 16.53547157514790 16.41996263524448
C 8.08420498398460 19.40618299144724 3.97372081717670
H 7.77314460391618 19.52487345417802 2.91467566461585
H 8.76338757464424 18.53011477918112 4.03625655534672
H 8.66791788370324 20.30191476710892 4.26645628464861
C 6.02965831620850 17.57819915531676 13.92260323601380
C 7.88468600227561 18.87925525959530 12.79714006961512
C 7.31226416097081 19.13384654113725 6.34925125682150
C 3.75347478422861 20.42093530445611 6.91075840557117
H 3.92783728397099 19.48666256190777 6.34464330894876
H 4.72399014077483 20.71700604710199 7.35081377740333
H 3.38958704873778 21.21266064522133 6.22111381630024
C 6.61307929902150 22.39370261059813 9.15605106365741
H 6.38563319373959 22.75215310613165 8.13036634490408
H 6.64203369497124 23.28164991540455 9.81968370922733
H 5.76894294027152 21.74898511218616 9.48186265233649
C 7.47008259553606 15.53398079843581 14.20547376945795
H 7.86887620444260 14.61931698313106 13.71909475051106
H 6.93939534388711 15.22796616434542 15.13157322324190
H 8.32958368006707 16.16822478666052 14.50179863102061
C 3.51931590459508 16.37957453658232 7.04435298671851

H	3.12343174200867	17.17037138553824	6.37785079941618
H	3.30776550329494	15.38584189555248	6.59515713091422
H	4.61226561054830	16.52919405952620	7.13334759304386
C	1.39719674851230	16.98298888441478	12.42379678071181
H	1.06783416273938	16.19687145137926	11.71683447758752
H	0.63850257881074	17.08406057024012	13.22878914838515
H	2.36411842345009	16.66659806404453	12.85941148096021
C	2.31349040077611	21.78199981399785	8.93080084342207
H	2.02827739391169	22.50255248814110	8.13451936928141
H	3.24919576307601	22.11573193692528	9.41865281608724
H	1.52236971102705	21.75621578377957	9.70507267114526
C	1.97665761038193	19.71522586555338	12.89383301032750
H	2.07113673613581	20.74060053938069	12.48839951473500
H	2.94502780416474	19.44815130931864	13.35867206819542
H	1.17088516219062	19.68785607023929	13.65896708528142
C	1.01787025246115	16.15604919113702	8.36790437276980
H	0.54440399950853	16.97518687077091	7.79519692040301
H	0.47663455626713	16.05886565060696	9.32921647810651
H	0.92044150518990	15.20611958739180	7.80140474891002
C	7.20737530650759	15.62790055224922	8.99940186766767
H	6.84700228314975	15.34715840691049	7.98745185326087
H	6.37401245670624	16.15419548479416	9.51188280160132
H	7.41941390811015	14.69036627236518	9.55377154923672
C	9.62660224920892	15.82161400879778	8.21785669975321
H	9.88079206645816	14.86627293203215	8.72228407157861
H	10.53269221091066	16.46058521628734	8.21339814799909
H	9.37586192490257	15.58717948299372	7.16191382621445
C	1.01971794445943	19.83294773242958	7.33055953577537
H	0.80124775577094	20.69227601389517	6.66231999229699
H	0.17633583116244	19.68964398601603	8.03255823734597
H	1.11307984537727	18.91757326083806	6.71423793295931

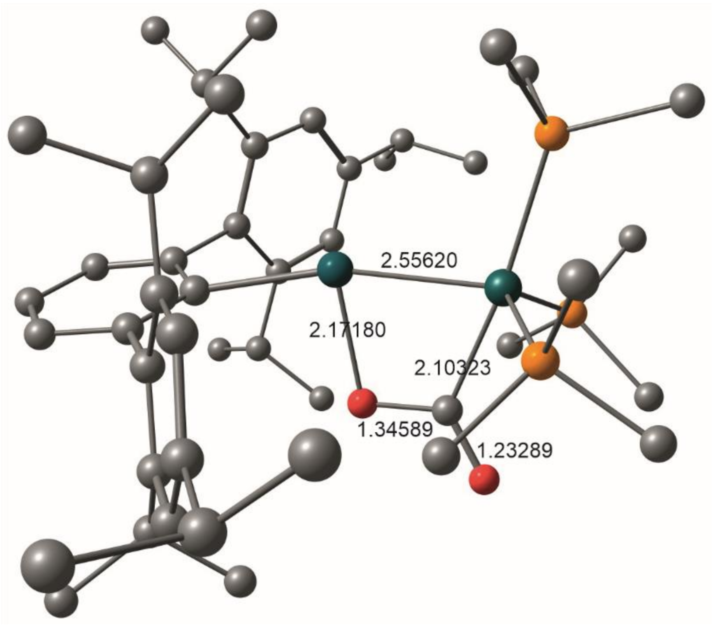

Figure SI96. Optimized structure **13**, distances in Å.

Coordinates from ORCA-job bp86optfreq_hnumac E -3297.617391397625

Sn	2.99830822953468	17.66420211274047	15.87280673947978
Rh	4.40787350688736	15.99727214497533	14.54291005129836
P	5.83660877107301	14.60489759674573	15.66251882413344
P	2.78533147393612	14.45867509214031	13.93402455338455
P	4.85593137468956	16.98821848183819	12.54198448526645
O	5.04380668131316	18.37904600865985	16.01991049846333
O	6.91703793439722	17.61594381107955	15.06236795508797
C	1.91312750437518	17.89341323372807	18.72020462667575
C	2.40495323827551	20.96646551420565	14.82160442707148
C	3.56975950654067	21.70432977181787	14.49177064087206
C	0.90390311634902	16.92980632051515	18.45106688544356
C	1.10271112331768	15.60517158286467	18.87894388329974
H	0.32067216566396	14.85183381246826	18.68972138318557
C	3.11011565684911	17.51475036065937	19.38689124146595
C	-0.38839459175129	17.31896613001340	17.73959993387921
H	-0.26838940087584	18.36490888573591	17.39152765000129
C	2.27042685272866	15.20798110773135	19.55042489973121
C	2.19236402516014	19.49431464091887	16.85856846780514
C	3.26607233828566	16.17450688582420	19.78311219228014
H	4.19008257341886	15.88278674522595	20.30587163110913
C	3.01483139100817	21.50381535838928	12.10308787816475

C	2.02750300406006	20.75429163236701	16.25243073848819
C	3.33162127922124	21.85048104662939	10.65445719286610
H	4.42227890597322	22.06656346670801	10.61069103162095
C	1.75947116806201	19.28330998054168	18.18463613809921
C	4.16952656463565	18.55545576841460	19.73150752437486
H	3.93804046827024	19.46383904413315	19.13899514904300
C	1.89034189938795	20.73563086939886	12.45139635276096
H	1.23908728139294	20.34794264355043	11.65571958407996
C	3.84367188813298	21.96633883924584	13.13728514464996
H	4.75050188215698	22.53487370956265	12.87418043380182
C	1.19315083656645	20.33752308520311	18.92507042473976
H	0.86101196819247	20.16516878909612	19.96083998435739
C	1.45773258898222	21.80767706910792	17.00144302262334
H	1.33179019065412	22.79542419200037	16.53043897681855
C	2.44512168830241	13.76375077394058	20.00011640745279
H	1.46863571200281	13.25782766462489	19.83307950685730
C	1.56937814309229	20.45245563495023	13.79090701219336
C	5.70817922863715	17.48407470438358	15.26555262147227
C	4.53417198770042	22.19371557924037	15.56582740326699
H	4.13810713568374	21.85277396205899	16.54366069955743
C	3.49288717639763	13.04162651539809	19.13520373023917
H	3.23390081284752	13.12441051836153	18.06164493671244
H	3.56149269379165	11.96548772453680	19.39727678949586
H	4.49940686199773	13.48767629235857	19.26983769749891
C	0.29760752117009	19.67649779094668	14.12563808337699
H	0.47472351013270	19.15509564849532	15.08944613824475
C	7.48358672949566	14.53887211837108	14.85824914631821
H	7.38437781416781	14.05786420640949	13.86628008404411
H	8.21959418025492	13.97797250924738	15.47043518009104
H	7.81203701402827	15.58755046135064	14.72348425086792
C	5.59365558220873	18.12357597825153	19.35431368613383
H	5.67098306607267	17.95897423826361	18.26220814624779
H	5.90642918605503	17.19966905101006	19.88442345513831
H	6.31737293694392	18.91891922661777	19.62581525194224
C	5.92628565852458	21.55810843277077	15.39880082241523
H	5.86540795665311	20.45464718967884	15.48549921102414
H	6.61425736716766	21.92390902624806	16.18927573709155
H	6.37918355340139	21.81687529920518	14.41845777455396
C	2.00550402759644	13.53619403183457	15.32394164473707
H	1.16487395347341	12.89610262085686	14.98591711075451
H	1.64129233955970	14.27251931069004	16.06642656490130
H	2.76419490598858	12.90474724049789	15.81944302161365
C	1.05271854305654	21.60201423779420	18.32984544537831
H	0.61225432803732	22.43287872209959	18.90221290115093
C	6.28190816958602	15.09462869900493	17.37562960987092
H	6.97215985318182	14.36797616262929	17.85145556819741
H	5.35696436476979	15.18876524079155	17.97457314116636
H	6.76533073879335	16.08773702985697	17.33552164514581
C	-0.65301044425381	16.45272188017843	16.49713290682383
H	-1.55145399599156	16.80767496367319	15.95209630904505
H	-0.81858310137268	15.38692368339534	16.75884905980967
H	0.20916021122836	16.49319310350286	15.79887375072043
C	2.77780378606858	13.65503721863194	21.49818628356178
H	2.82584811898883	12.59296462669113	21.81557476591951
H	2.01526673052847	14.16759196145299	22.11830880072039
H	3.76197359854532	14.11365928367702	21.72770049563524
C	-0.04695350623385	18.60376932526764	13.08471184695964
H	-0.37833040524540	19.04413428735302	12.12149512934616
H	-0.87354592189222	17.96043585971871	13.44876668527315
H	0.82808962600695	17.95538943739086	12.87983906021365
C	3.04411073999751	20.70148863056916	9.67521042779373
H	3.40305889958153	20.95537148658299	8.65695237301707
H	1.95670444403180	20.49464460431626	9.59392701087182
H	3.54145880104964	19.76383522714473	9.99366126810871
C	2.58592475346692	23.13495048454148	10.23876376109340
H	2.84821257543596	23.43498997492871	9.20261610200986
H	2.83064093759281	23.97722285259284	10.91639697158769
H	1.48758423517538	22.97851256658844	10.28309719288493
C	-1.57734774987962	17.29663042466962	18.71723498892868
H	-2.50970957617248	17.62823065521604	18.21487672780099
H	-1.39335951906490	17.96831308026156	19.57961673968702
H	-1.75080965938626	16.27554237018081	19.11711121347277
C	1.26164654548299	15.07945476830280	13.11034147987680
H	1.52342317392308	15.60106063513986	12.17282335630135
H	0.77616898763873	15.81166155074847	13.78174889472821
H	0.54732740644060	14.26044341064857	12.88811090180691
C	4.05390268022336	18.93479182235012	21.22118370941228
H	4.77499025351586	19.73756508075967	21.47980760931059
H	4.26742327857240	18.06129758614496	21.87287748050788
H	3.03441975035665	19.29503363839784	21.46749821051443
C	5.51552603296131	12.80494744679475	15.89801496185231
H	5.29544486183374	12.32670337949253	14.92491840051085
H	4.65028340449562	12.65434947821250	16.56827945785991
H	6.39888093772182	12.30648999458757	16.34701085213037
C	4.59685186026243	23.73151178870930	15.60078177196989
H	4.99975357581484	24.14318203142251	14.65142516632244
H	5.25793691697705	24.07845492321771	16.42174533875491
H	3.59226998319337	24.17535126012360	15.75752450199370
C	3.74512966924582	16.77438526046907	11.08863672319360
H	4.18405018286857	17.23048108902211	10.17738071994543
H	2.77761677926973	17.26864055773256	11.30224758050374
H	3.55982628506994	15.70033152059283	10.89579860419342
C	3.24763979845253	13.09061373541799	12.78855829167190
H	3.58550950045656	13.52834743056435	11.82914673966952
H	2.40747198883995	12.39288629630033	12.59360548016226
H	4.09810853390665	12.52581590398880	13.21449138145241
C	5.05030088911742	18.80796250070603	12.52458325019945

H	5.33685006363369	19.18124900872261	11.52023434861999
H	5.81989461387780	19.10294779439478	13.25949001373467
H	4.09196672591422	19.26826864522646	12.82341583566408
C	-0.87981266674140	20.64325522166250	14.35467836453700
H	-1.09400733573955	21.22436276361855	13.43333081043221
H	-0.65122557322834	21.36211495560906	15.16596919229331
H	-1.79970778012860	20.08911161148104	14.63585561224766
C	6.47040610618860	16.43809345951572	11.86592518715961
H	6.73371793928528	16.96902448908036	10.92733244288570
H	6.44441387074007	15.34691190291820	11.68172887575231
H	7.23031904371236	16.65012449237700	12.64193560896077

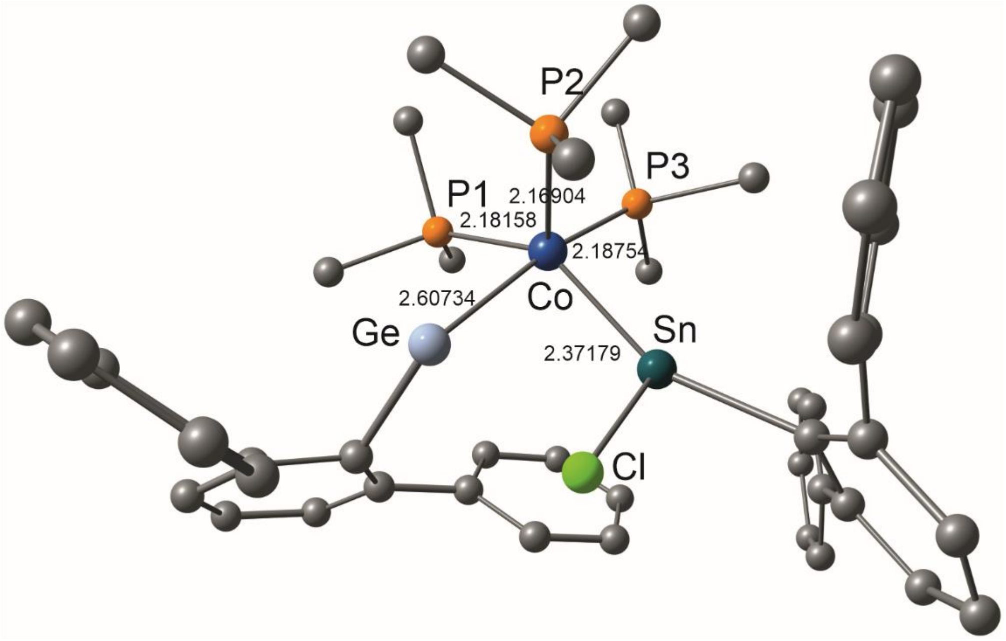

Figure SI97. Optimized structure of intermediate (H atoms have been omitted), distances in Å, Sn-Co-Ge 81.8°.

105
Coordinates from ORCA-job Zwischenstufe_CoSnGe_BP86

C	-0.40885769086536	3.60685054113368	-2.06816056197461
C	1.76237265784140	3.93930281811698	-0.31838439725246
C	-0.84555040560003	4.54851945426192	0.49414113229716
C	1.11529696456111	-5.93830603225405	1.07164859862378
C	2.26919862774555	-5.14930403945498	1.09977590440216
C	-0.12947480908777	-5.33906916057266	1.29678593497502
C	-3.43032984559453	2.41023425832105	0.74840436812813
C	2.19179257176294	-3.74158687820620	1.24109974233354
C	-0.23358121464697	-3.94019646981094	1.46183502546755
C	0.91539869963257	-3.11023273390973	1.32374210019230
C	-2.54756504769749	1.22270867758624	3.13148641641657
C	2.80166350686623	1.63578980797611	2.12816150934709
C	0.78471447992080	3.19000337932975	3.31471658854269
C	-3.30806507497473	-0.35232322233375	0.91332067005260
C	0.86753660235429	0.44906347828821	3.80165204097620
P	0.07803253384033	3.16871808709356	-0.33917873210116
P	-2.25485277434572	1.09851145795906	1.31364630048055
P	0.97145986405522	1.61100996755517	2.36167402278301
Co	-0.21970458248654	1.20880206946751	0.57151428913323
Sn	0.53409247344813	-1.03933492174348	0.51641650172542
H	-0.38271872551213	4.70554136005322	-2.21658191333869
H	0.26537680144323	3.12246070661423	-2.79606591328527
H	-1.43823165739630	3.24610539942292	-2.27112938562871
H	1.78239976296971	4.87524570008038	-0.91293570752144
H	-0.38969042627425	5.52824927462676	0.24413361028883
H	2.50170482994865	3.22695172402177	-0.72177897356898
H	-1.88850285211354	4.55042509726485	0.13162636121128
H	2.04566608574111	4.18197659762603	0.72310978486784
H	1.19281826594789	-7.02926404237386	0.94877393136012
H	-3.16766396728027	3.38718760988450	1.19039078876308
H	-0.85894973041332	4.42131554759071	1.59034397165592
H	3.25651193180645	-5.63321499705954	1.06036131705372
H	-1.03171613496581	-5.96076963344766	1.40254240144333
H	-3.38346227533358	2.48060167240591	-0.35681765876840
H	-4.46835908697394	2.15296404780518	1.04181928283313
H	-2.08440200766843	2.14722404014521	3.52643689979354
H	3.07056932390566	2.42872864390089	1.40922902979201
H	1.05677997733824	4.05445934928156	2.68125376536580
H	-3.63135143709797	1.23337721303623	3.36600862815233

H	3.12024772856724	0.67686212571602	1.68083816134837
H	-0.26677026173676	3.31721150924742	3.63587069416151
H	3.34370758984156	1.80691844691646	3.08174354090279
H	-2.08917838600148	0.35140251281906	3.63158324649950
H	-3.49012092947058	-0.36143875338079	-0.17934497568781
H	1.43531743643544	3.19354173601481	4.21326732009603
H	-4.27821306789607	-0.30861838694769	1.44635889720997
H	-2.78011771079940	-1.28239441669725	1.17125469423815
H	1.01367113768409	-0.59290759996361	3.45735149491954
H	-0.14165726032427	0.50838118939482	4.24728651600160
H	1.62145831955227	0.68311414335694	4.58126791057623
C	-1.53967487948061	-3.41980328609741	1.94694465776246
C	-2.76980089308121	-3.85764338668546	1.40488773432765
C	-1.56446206321122	-2.61038736530060	3.10689591559770
C	-3.98314992960314	-3.52271886606992	2.02217230468496
C	-2.77933074292761	-2.28718809980755	3.73146812900508
C	-3.99260096740951	-2.74769115274512	3.19549767137719
H	-2.76365367653840	-4.45487587284928	0.48146035254629
H	-0.60918027539408	-2.29155247937625	3.54921431360915
H	-4.93172532874863	-3.86482082303714	1.58098280956830
H	-2.77726557985069	-1.68931379702809	4.65557453849349
H	-4.94525852467455	-2.49846742573636	3.68701445959071
C	3.46995425637847	-3.00761743044946	1.43315174747167
C	3.53428437749331	-1.91692642506598	2.32884084759407
C	4.67831375930756	-3.44225536172301	0.83533975294510
C	4.75316368039418	-1.28629579829525	2.62221281603341
C	5.89781572577874	-2.81744875554192	1.12962729370538
C	5.94282147629379	-1.73531417554062	2.02771165420422
H	2.61497195277287	-1.60058950494451	2.84041949218487
H	4.65758173947317	-4.27093914139228	0.11212805151917
H	4.77460371601468	-0.44817125436033	3.33447024659964
H	6.82080545444376	-3.16993509045081	0.64444110471551
H	6.90081364255591	-1.24727878171871	2.26226102122877
C	0.55748130375684	2.06283677367445	-5.75062482502970
C	1.47241756791159	1.61576459511337	-4.78652295520048
C	-0.80018821780446	1.74790899310020	-5.61279147717884
C	1.02676201478117	0.89051407925402	-3.65904425566785
C	-1.26101618074187	1.03306919194490	-4.48411128260604
C	-0.35630492621929	0.63908575112704	-3.44922575756373
Ge	-1.38889886111047	0.48069689741634	-1.64232148968276
H	0.91011395096726	2.62071043749516	-6.63191785780862
H	2.55030958311924	1.80023151716705	-4.91952245248453
H	-1.51625788943846	2.03146236281628	-6.40017690754880
Cl	-1.01578838871029	-2.17482573652172	-1.29123847105089
C	-2.70911003767785	0.69788793478490	-4.38986420276097
C	-3.70898587896843	1.64796820916536	-4.69064970146221
C	-3.10840320947596	-0.59858920017736	-3.98340737286634
C	-5.06919231795339	1.32246516454658	-4.56260666422231
C	-4.46622822388115	-0.92594972667109	-3.86439684463553
C	-5.45201669169495	0.03635996269112	-4.14630588377236
H	-3.41199136470767	2.66543209085798	-4.98913181813279
H	-2.33557486390969	-1.35465388731013	-3.77603726867516
H	-5.83587814710118	2.08136471884671	-4.78381000192161
H	-4.75481682992954	-1.93843690244891	-3.54322759373449
H	-6.51870124469836	-0.21735324133578	-4.04604082301216
C	2.03919445682274	0.37025471163726	-2.71129389157855
C	2.04467207707926	-0.99975645347780	-2.37723398184603
C	3.03544956745303	1.20629202450523	-2.16860367950619
C	3.01047259116985	-1.51123124106837	-1.49608478313668
C	3.99490038288166	0.69849837725366	-1.27937103609148
C	3.98606490876112	-0.66111294974672	-0.93982116676615
H	1.27841628355610	-1.65792455917912	-2.80960276487657
H	3.05150869485444	2.26780445076044	-2.45726897906231
H	3.01496078612918	-2.58492049261899	-1.25885435411885
H	4.75819366216915	1.36673282626633	-0.85145034386314
H	4.74071471197068	-1.06214676181130	-0.25265520218476

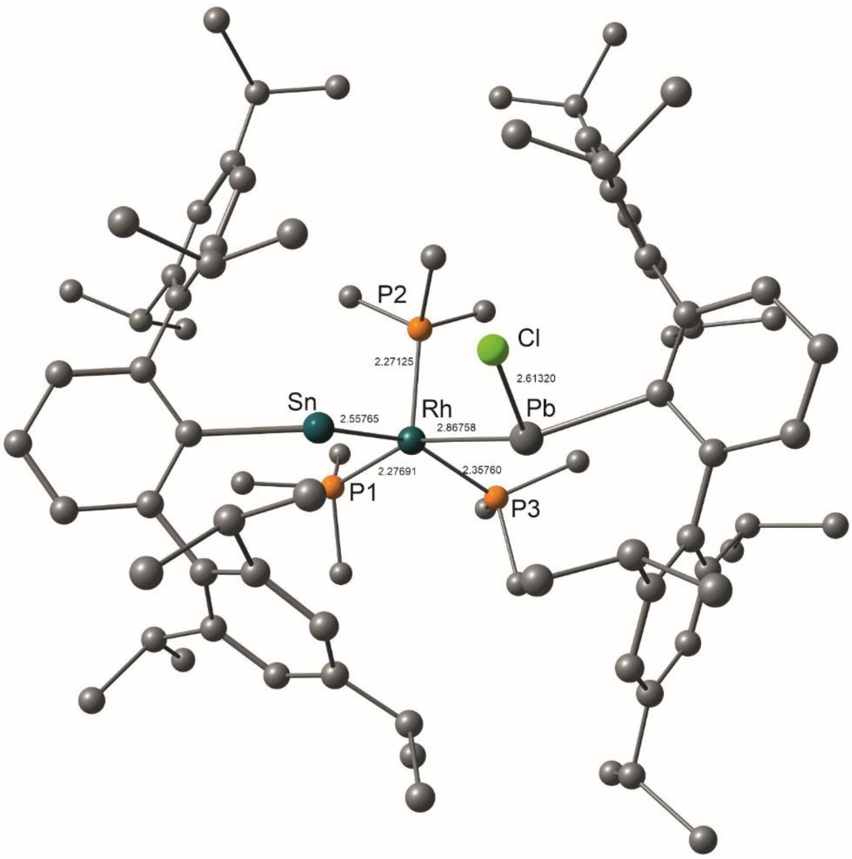

Figure SI98. Optimized structure of intermediate, distances in Å, Pb-Rh-Sn 85.4°.

```
213
Coordinates from ORCA-job Zwischenstufe_RhSnPb_BP86
 C  -0.55515690768455   3.54412721066108  -2.47189755260219
 C   1.72399440960068   3.72294300129200  -0.85744353427159
 C  -0.91697072370726   4.05799542380832   0.22536335340620
 C   1.65987179195919  -6.15324761279544   3.13829560118014
 C   2.72235862957595  -5.32273268862671   2.76174064678817
 C   0.35924205331932  -5.63358699082770   3.15184077869085
 C  -3.45301945028606   1.34441042719583   0.05096442295144
 C   2.47369324434316  -4.00155011275058   2.32926320461036
 C   0.08840186089473  -4.31361544875819   2.72060480338161
 C   1.14825637008995  -3.49794695707531   2.24446835487004
 C  -2.36963392912139   0.34072931850519   2.46076866620268
 C   3.19729229865659   1.15060916936260   1.47041801957233
 C   1.26089950203733   2.88600589153943   2.64229533595351
 C  -2.83530169368007  -1.38701110776127   0.28094576556033
 C   1.23461906360332   0.20470291719123   3.27210735104861
 P   0.06322885682254   2.92622390860344  -0.85664748451761
 P  -2.10743680987848   0.24903973485327   0.64431165753877
 P   1.38321594146725   1.25672752343356   1.77452130867726
 Rh -0.01904517596619   0.79359530915428  -0.06326029212038
 Pb  1.04593659120316  -1.81062317012129   0.49067705332564
 H  -0.55400201023763   4.65346779215577  -2.50323331920418
 H   0.07158623219280   3.14847910738802  -3.28796610082331
 H  -1.57936531110199   3.16858073405275  -2.63870913940317
 H   1.67150741873286   4.80538213597466  -1.09471850769520
 H  -0.64158004200040   5.11862637322454   0.05016732019493
 H   2.33868503339369   3.20418085515458  -1.61726524458102
 H  -1.98560647830300   3.92954897341848  -0.02994592828903
 H   2.21823939189836   3.59619184104439   0.12105933889920
 H   1.84746922016416  -7.18949497983928   3.45857727730244
 H  -3.25708793200143   2.38531655705615   0.36652709135728
 H  -0.78830287701755   3.80981446173373   1.29154135501683
 H   3.75910974164011  -5.69335365012884   2.79861259675580
 H  -0.47030886167307  -6.24797244197124   3.53375043298559
 H  -3.48408173425358   1.30857706066032  -1.05268367915697
 H  -4.43988828881577   1.02831751007518   0.44746181892321
 H  -2.00135142688909   1.31103046845236   2.84609626263054
 H   3.48037208011931   1.82136117369515   0.63834501807270
 H   1.41658085682708   3.73954768579178   1.95949552608572
 H  -3.44029363156088   0.21986106713810   2.72558170996687
 H   3.44856023916113   0.11630493850922   1.16271901698980
 H   0.25073305346997   2.97770278506841   3.08675578498672
 H   3.79198683340394   1.40074418995194   2.37272352088365
 H  -1.80587264875296  -0.48030734170100   2.94043554943969
 H  -2.90238753445085  -1.51164718061951  -0.81659540570055
 H   2.01035001387775   2.94064139070869   3.45793926998466
 H  -3.84241697715026  -1.49983430393234   0.72747427762243
```

H	-2.17783713133588	-2.18017980658323	0.67890713636092
H	1.55202036937329	-0.83342550741161	3.05567117207544
H	0.17413884179180	0.17299049158365	3.57096068767011
H	1.83817688368576	0.59031607255895	4.11633068509777
C	-1.30286432630943	-3.80442640145178	2.96405163875687
C	-2.41969833671152	-4.29973020367738	2.23382229549734
C	-1.51570884490088	-2.92875315199042	4.07314476793558
C	-3.71551234506647	-3.89309727613631	2.60774551874742
C	-2.83186529726638	-2.56255274187554	4.40681286452162
C	-3.94571035387879	-3.03315282232463	3.69275198703412
H	-4.57350385724136	-4.27872623683652	2.03503331855066
H	-3.00858723751458	-1.89854845933725	5.26615009439246
C	3.64868199516363	-3.12617062522260	1.99133056667369
C	4.20854535823838	-2.27525054847191	2.98840388920990
C	4.21716867268165	-3.15991727525760	0.68805477164916
C	5.30884954929847	-1.46642230503257	2.65037861359108
C	5.30658753165693	-2.31627058607933	0.39415102490862
C	5.86857992344567	-1.46524585225243	1.36014391121421
H	5.74479290112372	-0.81404538274193	3.41923683739679
H	5.74352476862751	-2.33302161784841	-0.61661785579312
C	-1.53160984263857	1.81125789786989	-7.03812514007059
C	-0.19891690175474	1.71292639197488	-6.60723194396836
C	-2.56930801636688	1.48049680553797	-6.15706665405217
C	0.09286448705351	1.29836181905862	-5.29404907402709
C	-2.29246490334679	1.06857854441555	-4.83274657682290
C	-0.95409830606102	0.98720384166542	-4.37815209137263
Sn	-0.32925316959690	-0.06928605272000	-2.45089166007230
H	-1.75871650299499	2.12617427021786	-8.06804869411170
H	0.62148867667948	1.94347438441078	-7.30343124999287
H	-3.61577652411181	1.52243849867531	-6.49766732756072
Cl	-0.01288063747659	-3.63005657343093	-1.05766962934194
C	-3.43742359500545	0.66070175095061	-3.95872535418322
C	-4.36098322221009	1.62670685247703	-3.47559953578006
C	-3.65302415678289	-0.72502900860633	-3.69297775926622
C	-5.49056843096585	1.18946324774492	-2.75722463690364
C	-4.77484804216019	-1.10476949376938	-2.93464409500525
C	-5.71880822410227	-0.16651673117343	-2.47807458151456
H	-6.22230735591177	1.93119730671092	-2.39770330576567
H	-4.94128349276216	-2.17439859521827	-2.73667308306128
C	1.48999458881634	1.13062284904283	-4.77444506456776
C	1.93415345330016	-0.19567203917443	-4.46786465046269
C	2.31528355009240	2.23969749828251	-4.45257188247466
C	3.08293468293060	-0.35696725821787	-3.67234025096328
C	3.45553788185671	2.02420720766968	-3.65062846728674
C	3.81967431656385	0.74406093306447	-3.19561870264982
H	3.40212206150183	-1.37146807379510	-3.39954991491232
H	4.07524187784882	2.88930511585063	-3.37803199276476
C	-2.80280725415719	-1.79752512872056	-4.37536954426157
H	-1.80673811114663	-1.35544646942144	-4.58408761786997
C	-4.18629083145047	3.11786748842848	-3.75842644479534
H	-3.17148611427685	3.25376495230398	-4.18633980332842
C	-6.97488254807612	-0.61073299359554	-1.74213240353245
H	-7.55444607868621	0.30994347884358	-1.50920660055633
C	-2.26317414688583	-5.31491337785618	1.10253739457546
H	-1.19492066851293	-5.31811449940324	0.80487906443551
C	-5.35533162318305	-2.63935890211392	4.11306107927196
H	-5.25252322150243	-1.95818716040573	4.98677328038478
C	-0.35476078783159	-2.46453377436849	4.95577867802373
H	0.48909656298990	-2.24015693944578	4.27141476242762
C	3.67022513315896	-2.28044947076022	4.41917332721942
H	2.56856727505306	-2.40652733395153	4.34894680378578
C	3.71490257846988	-4.15412258972617	-0.35386694833164
H	2.69082026603050	-4.46543787577471	-0.05955552014755
C	7.09938546629595	-0.63406816708229	1.02221581439022
H	7.07301645131980	-0.46280311583818	-0.07637683335044
C	4.99543494177567	0.50816728395347	-2.25171548877754
H	4.60058388913278	-0.10642648063844	-1.40937826784828
C	2.05368803903980	3.60697517447559	-5.08381024217142
H	0.96614397049980	3.67178745420800	-5.30049145990632
C	1.27232728425654	-1.38773496070385	-5.16620833285598
H	0.18201569613101	-1.18476337683337	-5.21349597776128
C	-6.16946422179795	-3.86494427811552	4.56840337922515
H	-7.17543546443650	-3.56481927320396	4.92919111323223
H	-6.31216629321990	-4.58102683828475	3.73229766383207
H	-5.65411220626709	-4.40600300023967	5.38719747117955
C	-2.64308480464367	-6.72880403767955	1.58888554216566
H	-2.48299171665427	-7.47490909583262	0.78302386422051
H	-2.04450033711309	-7.04307745828976	2.46580949428774
H	-3.71302850531396	-6.77130271548088	1.88433879019422
C	-0.65650071455433	-1.20113840423414	5.77560526816834
H	-1.09752157507011	-0.38987389921894	5.16282857505914
H	-1.36337875861311	-1.41033182248044	6.60565367545054
H	0.27624872899585	-0.81326356420388	6.23284301987926
C	-6.08291261368807	-1.86913076949450	2.99959530071263
H	-5.50878360727332	-0.97229611425619	2.69013459205918
H	-6.21548749931503	-2.50460677869183	2.10100241548946
H	-7.08980143189952	-1.53664453889421	3.32736225512018
C	0.12037058722045	-3.58533743361094	5.90338973277771
H	0.96810314021309	-3.23119423059049	6.52668597511005
H	-0.70054349821176	-3.89407742295279	6.58409534849677
H	0.46008085453193	-4.47723910598308	5.34560867454598
C	-3.08165800927844	-4.95872045310300	-0.15048933513631
H	-2.87096869479385	-5.68706277372343	-0.95946098171915
H	-4.17497964169659	-4.98710138380915	0.04427726527601
H	-2.81619789132046	-3.95846489165829	-0.53560597425359
C	3.96175773736283	-0.98715109128466	5.19570795119922
H	3.37394376221386	-0.96364940141947	6.13568904847581

```
H 5.03210582803654 -0.91458749406784 5.48099438751166
H 3.71102072011286 -0.08110998290412 4.61132355812155
C 8.38155718852964 -1.43316879041334 1.33254428756840
H 8.45047411815969 -1.65195060698742 2.41883080471448
H 8.38888436951749 -2.40300671312142 0.79605408625558
H 9.28930423258292 -0.86603326075050 1.03794816220506
C 4.59873780477746 -5.41710331740109 -0.34954384660838
H 4.21825523806821 -6.16238166876033 -1.07812641002136
H 5.64537509568264 -5.16798993986647 -0.62495807728196
H 4.61680134156950 -5.89454423056005 0.64951028367074
C 7.12346085275199 0.73978218628103 1.70850239085403
H 7.97241076934926 1.34827862289587 1.33512428994686
H 6.18749507050181 1.30339949219984 1.52322378852331
H 7.24997420467685 0.64683443921485 2.80721628688944
C 3.61738859902983 -3.55692993143977 -1.76118327227957
H 4.61438672052029 -3.31432562919578 -2.18603909611679
H 3.12072034396646 -4.27040146682201 -2.44606081303028
H 3.00797547823929 -2.63122950053187 -1.75570082759709
C 4.21441750547401 -3.48585090351461 5.21427918035587
H 3.81898467003274 -3.47810655874761 6.25128626675644
H 3.92365119881356 -4.44538586340079 4.74945318360292
H 5.32260746779411 -3.44657685763387 5.26760794811564
C -6.64216134713305 -1.30017944902350 -0.40968444959755
H -7.56588275206688 -1.58697514157958 0.13331473937216
H -6.04309230981562 -2.22124659469985 -0.56778158753442
H -6.05253446404245 -0.63263958860207 0.24821594967942
C -2.57108182067469 -3.05401396470546 -3.52951034564015
H -1.89578511217702 -3.75307776898522 -4.06153730162277
H -2.07924749350687 -2.82121794574428 -2.56364442425496
H -3.51156974669466 -3.60414603206578 -3.31988186841788
C -4.27641593910166 3.97736146688084 -2.48490663777145
H -5.28629861558769 3.93306142021150 -2.02739916988222
H -3.55123467692136 3.64347588676636 -1.71821023749901
H -4.06359494008503 5.04110203967659 -2.71723611652070
C -7.85841102593224 -1.50498946811775 -2.63261938292403
H -7.34000533993057 -2.45292790111977 -2.88675628363577
H -8.80416731064877 -1.76893147354668 -2.11533295663234
H -8.11354147984895 -0.99700064180836 -3.58433616919548
C -3.42709315681827 -2.13874333663673 -5.74385130705106
H -3.51909732638802 -1.23498981052079 -6.37872250086038
H -2.80214891686244 -2.87854020207612 -6.28570197361530
H -4.44164717188097 -2.57117632429992 -5.61537956145508
C -5.20120663025250 3.60363604873196 -4.81159138455711
H -5.11294390485639 3.02915138723690 -5.75437817070220
H -6.24199207941705 3.48478060944977 -4.44405398848320
H -5.04430044381125 4.67667873014671 -5.04787037004394
C 1.77902155102134 -1.44187355802971 -6.62391045144746
H 2.87570044297136 -1.61184034533562 -6.65027151642345
H 1.28818537450458 -2.26941536314357 -7.17658337179669
H 1.56744614311442 -0.49633070496735 -7.16113640070761
C 5.57285504727108 1.79581717066699 -1.65274796906550
H 6.35053693991722 1.55980095107505 -0.90128352760096
H 6.04669222867516 2.43184362613582 -2.42956992776610
H 4.79223376180677 2.40217222842491 -1.15053160650378
C 2.43561822225267 4.81920164608817 -4.22409898295243
H 1.89665489549541 4.82825414190863 -3.26066341674671
H 3.52340991781883 4.84916332799298 -4.00591587352021
H 2.18827743680745 5.75779463288082 -4.76031528714605
C 1.46404379133885 -2.73507901003165 -4.46674527227634
H 0.88910032620541 -3.52083338559982 -4.99669553315291
H 2.52692381214616 -3.05251331752688 -4.47044717252255
H 1.10655094277061 -2.71577209708701 -3.41707160234209
C 6.10149811015939 -0.31446532449834 -2.94183630372529
H 6.93051791470614 -0.53477707299513 -2.23741624321713
H 5.71684311371922 -1.28079154348624 -3.32324657816672
H 6.52427951741443 0.24319459251539 -3.80350300121051
C 2.80203859348746 3.67172574099563 -6.43456220563995
H 3.89930517986651 3.64276948984778 -6.26876765834580
H 2.54796627848959 2.81342901622980 -7.08653308321642
H 2.56013854930779 4.60796264599021 -6.97943783191250
```

References

[1] a) M. Stender, L. Pu, P. P. Power, *Organometallics* **2001**, *20*, 1820-1824; b) B. E. Eichler, L. Pu, M. Stender, P. P. Power, *Polyhedron* **2001**, *20*, 551-556; c) L. Pu, B. Twamley, P. P. Power, *Organometallics* **2000**, *19*, 2874-2881.

[2] H.-F. Klein, H. H. Karsch, *Chem. Ber.* **1975**, *108*, 944-955.

[3] R. A. Jones, F. M. Real, G. Wilkinson, A. M. R. Galas, M. B. Hursthouse, K. M. A. Malik, *Dalton Trans.* **1980**, 511-518.

[4] T. Herskovitz, C. Kampe, H. D. Kaesz, W. M. Seidel, in *Inorganic Syntheses*, **1982**, pp. 99-103.

[5] R. S. Simons, J. C. Gallucci, C. A. Tessier, W. J. Youngs, *J. Organomet. Chem.* **2002**, *654*, 224-228.

[6] R. K. Harris, E. D. Becker, S. M. C. d. Menezes, R. Goodfellow, P. Granger, *Pure Appl. Chem.* **2001**, *73*, 1795-1818.
[7] a) L. J. Farrugia, *J. Appl. Crystallogr.* **1997**, *30*, 565; b) L. J. Farrugia, *J. Appl. Crystallogr.* **1999**, *32*, 837-838; c) L. J. Farrugia, *J. Appl. Crystallogr.* **2012**, *45*, 849-854; d) C. B. Hübschle, G. M. Sheldrick, B. Dittrich, *J. Appl. Crystallogr.* **2011**, *44*, 1281-1284; e) G. M. Sheldrick, Göttingen, Germany, **1997**, p. Program for the Solution of Crystal Structures; f) G. Sheldrick, *Acta Cryst., Sect. A* **2008**, *64*, 112-122; g) G. M. Sheldrick, University of Göttingen, Germany Program for Empirical Absorption Correction of Area Detector Data, **1996**.
[8] a) F. Neese, *WIREs Comput Mol Sci.* **2022**, *12*, e1606; b) F. Neese, *J. Comput. Chem.* **2003**, *24*, 1740-1747; c) D. Bykov, T. Petrenko, R. Izsák, S. Kossmann, U. Becker, E. Valeev, F. Neese, *Mol. Phys.* **2015**, *113*, 1961-1977; d) F. Neese, *J. Comput. Chem.* **2023**, *44*, 381-396; e) F. Neese, *Wiley Interdiscip. Rev. Comput. Mol. Sci.* **2012**, *2*, 73-78; f) F. Neese, *Wiley Interdiscip. Rev. Comput. Mol. Sci.* **2018**, *8*, e1327; g) F. Neese, F. Wennmohs, U. Becker, C. Riplinger, *J. Chem. Phys.* **2020**, *152*, 224108; h) E. Caldeweyher, C. Bannwarth, S. Grimme, *J. Chem. Phys.* **2017**, *147*, 034112; i) E. Caldeweyher, S. Ehlert, A. Hansen, H. Neugebauer, S. Spicher, C. Bannwarth, S. Grimme, *J. Chem. Phys.* **2019**, *150*, 154122; j) E. Caldeweyher, J.-M. Mewes, S. Ehlert, S. Grimme, *Phys. Chem. Chem. Phys.* **2020**, *22*, 8499-8512.
[9] a) A. D. Becke, *Phys. Rev. A* **1988**, *38*, 3098-3100; b) J. P. Perdew, W. Yue, *Phys. Rev. B* **1986**, *33*, 8800-8802.
[10] a) S. Grimme, S. Ehrlich, L. Goerigk, *J. Comput. Chem.* **2011**, *32*, 1456-1465; b) S. Grimme, J. Antony, S. Ehrlich, H. Krieg, *J. Chem. Phys.* **2010**, *132*, 154104-154119.
[11] a) F. Weigend, *Phys. Chem. Chem. Phys.* **2006**, *8*, 1057-1065; b) F. Weigend, R. Ahlrichs, *Phys. Chem. Chem. Phys.* **2005**, *7*, 3297-3305; c) B. Metz, H. Stoll, M. Dolg, *J. Chem. Phys.* **2000**, *113*, 2563-2569; d) D. Andrae, U. Häussermann, M. Dolg, H. Stoll, H. Preuß, *Theoret. Chim. Acta* **1990**, *77*, 123-141.
[12] E. D. Glendening, J. K. Badenhoop, A. E. Reed, J. E. Carpenter, J. A. Bohmann, C. M. Morales, P. Karafiloglou, C. R. Landis, F. Weinhold, Theoretical Chemical Institute, University of Wisconsin, Madison, **2018**.
[13] G. A. Zhurko, CHEMCRAFT (http://www.chemcraftprog.com).
[14] V. Ásgeirsson, B. O. Birgisson, R. Bjornsson, U. Becker, F. Neese, C. Riplinger, H. Jónsson, *J. Chem. Theo. Comp.* **2021**, *17*, 4929-4945.
[15] L. R. Maurer, J. Rump, A. C. Filippou, *Inorganics* **2023**, *11*, 129.
[16] a) H. Kruse, S. Grimme, *J. Chem. Phys.* **2012**, *136*, 154101; b) S. Grimme, A. Hansen, S. Ehlert, J.-M. Mewes, *J. Chem. Phys.* **2021**, *154*, 064103; c) S. Lehtola, M. A. L. Marques, *J. Chem. Phys.* **2023**, *159*; d) S. Lehtola, C. Steigemann, M. J. T. Oliveira, M. A. L. Marques, *SoftwareX* **2018**, *7*, 1-5; e) M. A. L. Marques, M. J. T. Oliveira, T. Burnus, *Comput. Phys. Commun.* **2012**, *183*, 2272-2281.
[17] L. Pu, M. M. Olmstead, P. P. Power, B. Schiemenz, *Organometallics* **1998**, *17*, 5602-5606.
